# Synthesis and hypoglycemic activity of quinoxaline derivatives

**DOI:** 10.3389/fchem.2023.1197124

**Published:** 2023-07-06

**Authors:** Weidong Jia, Jingjing Wang, Chengxi Wei, Ming Bian, Shuyin Bao, Lijun Yu

**Affiliations:** Medical College, Inner Mongolia Minzu University, Tongliao, China

**Keywords:** diabetes, quinoxalinone derivatives, hypoglycemic activity, molecular docking, quinoxalinone, Pioglitazone

## Abstract

In this study, a new series of quinoxalinone derivatives (5a–5p, 6a–6n) was designed and its hypoglycemic activity was evaluated. The results showed that compounds 5i and 6b exhibited stronger hypoglycemic effects than the lead compounds and were comparable to the positive control Pioglitazone. 5i and 6b may exert hypoglycemic effects by alleviating cellular OS and modulating the interactions among GLUT4, SGLT2, and GLUT1 proteins. The alleviating cellular OS of compound 6b was better than that of 5i, and 6b was found to bind better than 5i for most of the screening targets. In summary, compound 6b is a potential lead compound with hypoglycaemic activity.3

## 1 Introduction

Diabetes is a group of metabolic diseases characterized by high blood glucose levels. Diabetes is known as a “silent disease” because it remains asymptomatic until target organs are severely affected (Zimmet et al., 2018). Currently, 537 million adults have diabetes worldwide, which represents 10.5% of the global population or 1 in 10 individuals (Greenhill, 2021).

Diabetes can be categorized into type 1 diabetes mellitus (T1DM) and type 2 diabetes mellitus (T2DM) depending on the pathogenesis. The most prevalent form of diabetes in adults is T2DM, which accounts for 90%–95% of patients with the disease. It is characterized by hyperglycemia caused by defective insulin secretion and signaling cascade, of which defective signaling is primarily manifested as insulin resistance (IR). Complications resulting from T2DM are a serious health threat and include heart disease, stroke, neuropathy, kidney disease, visual impairment, peripheral vascular disease, ulcers and amputations, infections, digestive disorders, oral complications, and depression (Gross et al., 2017). These complications could be attributed to various factors, such as the polyol pathway, protein kinase C, advanced glycation end products, and inflammation (Forbes and Cooper, 2013). Most of the underlying causes are related to the overproduction of reactive oxygen species (ROS) in cells, and oxidative stress (OS) is a key factor that triggers IR. The high-glucose environment can lead to a surge in ROS, which aggravates islet β-cell damage and ultimately affects the body’s sensitivity and response to insulin (Yang et al., 2022).

As of today, diabetes remains a lifelong metabolic disease that requires long-term medication. Drugs used to treat diabetes are classified based on the route of administration into oral drugs and non-oral drugs. Oral medications are broadly categorized into sulfonylureas, non-sulfonylurea insulin promoters, biguanide hypoglycemic agents, alpha-glucosidase inhibitors, insulin sensitizers, dipeptidyl peptidase-4 inhibitors, and sodium-glucose cotransporter inhibitors. Non-oral drugs are mostly insulin and its analogs. However, these drugs have disadvantages such as toxic side effects, high price, and slow onset of action owing to differences in the route of administration or their inherent properties (Heppner and Perez-Tilve, 2015; Xu et al., 2018).

Hence, the development of a new glucose-lowering drug that has no toxic side effects and can regulate blood glucose levels effectively in the long term is the need of the hour. Upon reviewing the literature for lead compounds with hypoglycemic activity, we found a structure with a benzopyrazine backbone called quinoxaline. It was first reported in 1884 as an intermediate for the antituberculosis drug pyrazinamide. Subsequent studies on the pharmacological activities of quinoxaline and its derivatives revealed that they have a wide range of biological activities, such as antimicrobial (Patidar et al., 2011), antidiabetic (Kojima et al., 2020), anti-inflammatory (Indalkar et al., 2017), anti-Alzheimer’s (Sagar et al., 2019), and anticancer (Tariq et al., 2018). Moreover, quinoxaline and its derivatives possess multiple modification sites in their chemical structures.

Positions 1 and 4 in the quinoxaline backbone are the main modification positions, and the introduction of various pharmacodynamic groups has a profound effect on its activity. The heterocyclic structure of triazoles exhibits a wide range of pharmacological activities (Xia et al., 2010; Kamboj et al., 2015; Gao et al., 2019), including inhibitory effects on diabetes-related targets (Fuh et al., 2021). Furthermore, the triazole ring can act as an acceptor-ligand linker. There are CH-π interactions between 1,2,3-triazole and the enzyme, and the appropriate side ring (on the ring of the triazole hybrid system) can bind to the enzyme or amino acid (Bozorov et al., 2019). The triazole ring acts as a connector to link the corresponding drug active group, thereby increasing the drug activity of the lead compound. In addition, the number of methylene groups between the benzene ring and triazole can affect the activity of the compounds (Bistrović et al., 2018).

In this study, quinoxalinones were selected as lead compounds. Quinoxalinone derivatives 5a–5p and 6a–4n containing different substituents were designed and synthesized, and all 30 new compounds were screened for hypoglycemic activity.

## 2 Results and discussion

### 2.1 Chemistry

**Scheme 1:**
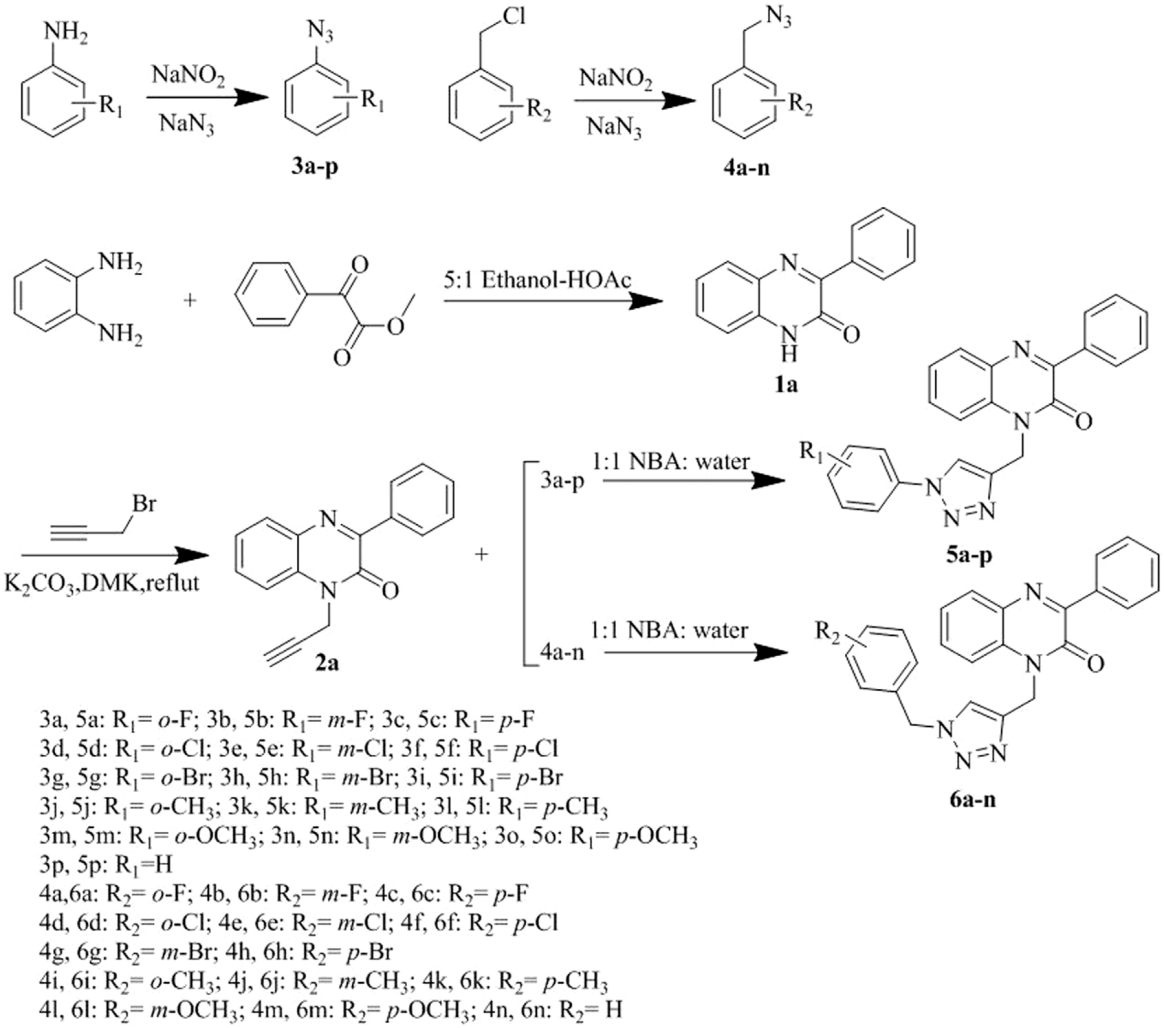
Synthetic pathways for the compounds 5a-p and 6a-n.

Strategies used to synthesize the target compounds analyzed in this study are outlined in Scheme 1. Overall, synthesis reactions involved three main steps. First, using benzene-1,2-diamine and methyl benzoylformate as starting materials, the lactam product 3-phenyl-1*H*-quinoxalin-2(1*H*)-one (1a) was formed via the cyclization reaction in a 5:1 system of anhydrous ethanol and glacial acetic acid. Subsequently, 1-propargyl-3-phenylquinolin-2(1*H*)-one (2a) was obtained with a simple nucleophilic substitution. Then, compound 2a was cyclized with intermediates 3a–p and 4a–n of different substituents to obtain the target compounds 5a–p and 6a–n, respectively. Proton nuclear magnetic resonance (^1^H-NMR), carbon-13 nuclear magnetic resonance (^13^C-NMR), and high-resolution mass spectrometry (HRMS) were used to structurally characterize all prepared compounds.

### 2.2 Structure-activity relationships

Results of the MTT assay of the compounds with LO2 cells showed that all synthesized compounds exhibited either no toxicity or low toxicity toward LO2 cells at concentrations of <40 μM. Some compounds did not show significant toxicity at concentrations of up to 80 μM. Overall, the toxicity of the compounds toward LO2 cells was reduced when the substituent on the benzene ring attached to the triazole was a methyl group. The compounds did not show significant toxicity even at concentrations of up to 80 μM. However, the introduction of methylene-based groups on triazoles increased the toxicity of most of the compound. Therefore, most of the derivatives in the 5a–p series were less toxic to LO2 cells than those in the 6a–n series. In the synthesized 5a–p series derivatives, interposition substitution on the benzene ring was often accompanied by an increase in cytotoxicity.

The results of *in vitro* hypoglycemic activity indicated that the hypoglycemic activity of the 5a–p series derivatives increased with the introduction of halogen atoms into the benzene ring. The following order of activity was observed: Br > Cl > F. Highest hypoglycemic activity was noted in compound 5i with para-substituted Br atoms probably because of its spatial conformation. No clear conformational relationships were observed for 6a–n; nevertheless, the hypoglycemic activity was greatly enhanced when the triazole-linked benzene ring interposition was substituted with an F atom (compound 6b).

### 2.3 Assssment of activity

**FIGURE 1:**
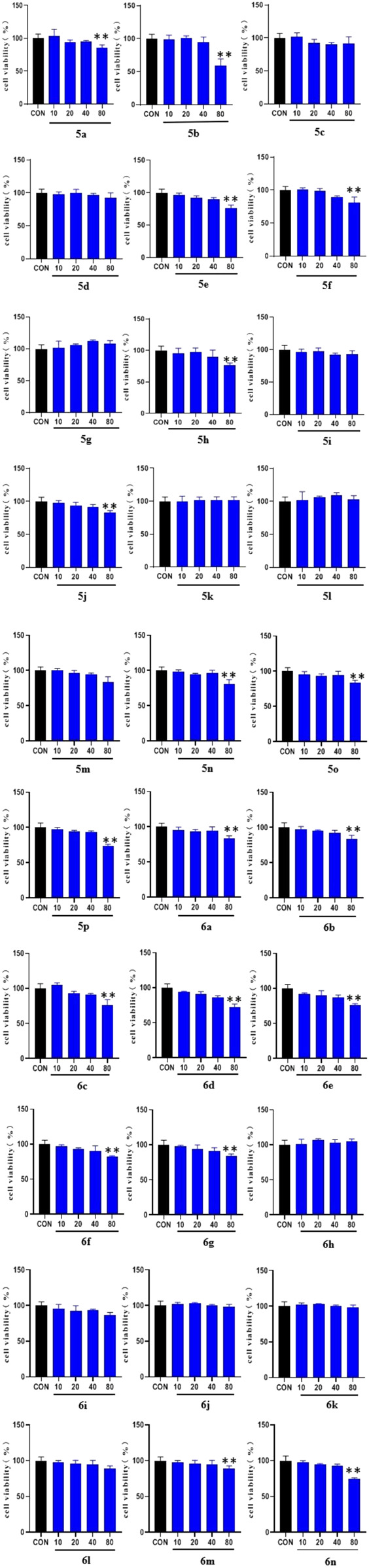
MTT results of the derivatives 5a-p and 6a-n on LO2 cells. LO2 cell cultures were added to 96-well plates (1 × 10^4^ cells/well in 100 µl/well) and treated for 48 h with the 30 synthesized compounds dissolved in DMSO (10, 20, 40, and 80 μg/ml) for 1 day. (* *vs*. CON, *p < 0.05, **p < 0.01).

To determine the hypoglycemic activity of the 30 new derivatives (5a–p and 6a–n), their toxicity toward LO2 cells was first examined using the MTT assay. The experimental results (Figure 1) indicated that the derivatives did not exhibit significant toxicity at concentrations of <40 µM. Therefore, they were administered at this concentration.

### 2.4 Analysis of the effects of derivatives 5a–p and 6a–n on glucose production in LO2 cells

**FIGURE 2:**
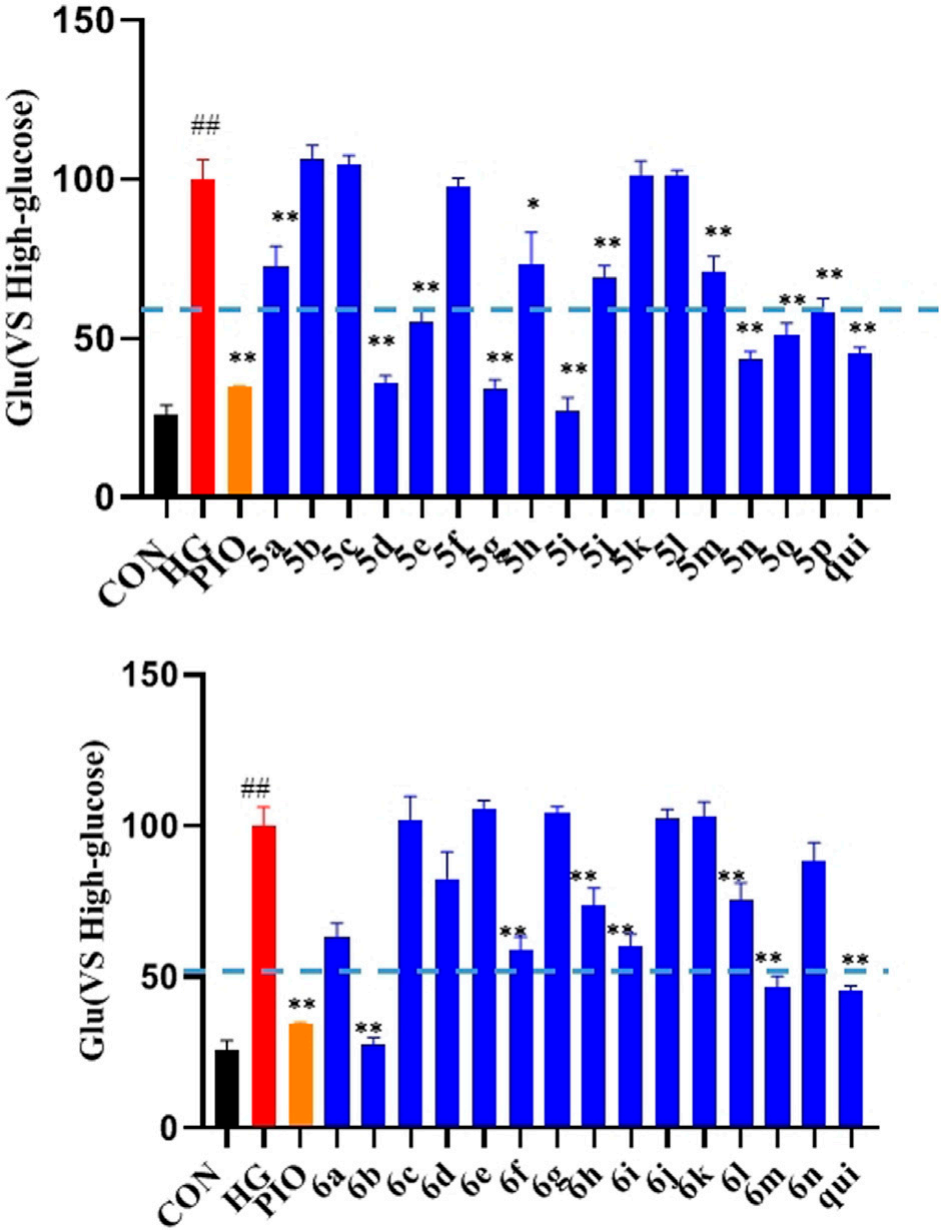
Effects of the compounds 5a-p and 6a-n on insulin resistance in LO2 cells. After treating the LO2 cells with 40 μM of the target compounds (5a–p and 6a–n) for 48 h, followed by incubation for 16 h in 1 ml of glucose-stimulating medium (phenol red-free RPMI1640 + 20 mmol/L sodium lactate and + 2 mmol/L sodium pyruvate). For the last 3 h, 1 nmol/L of insulin was added to each group, except the normal group. (# *vs*. CON, ##*p* < 0.01, * *vs*. HG, **p < 0.01, PIO, Pioglitazone; HG, High Glucose, qui: lead compound).

**FIGURE 3:**
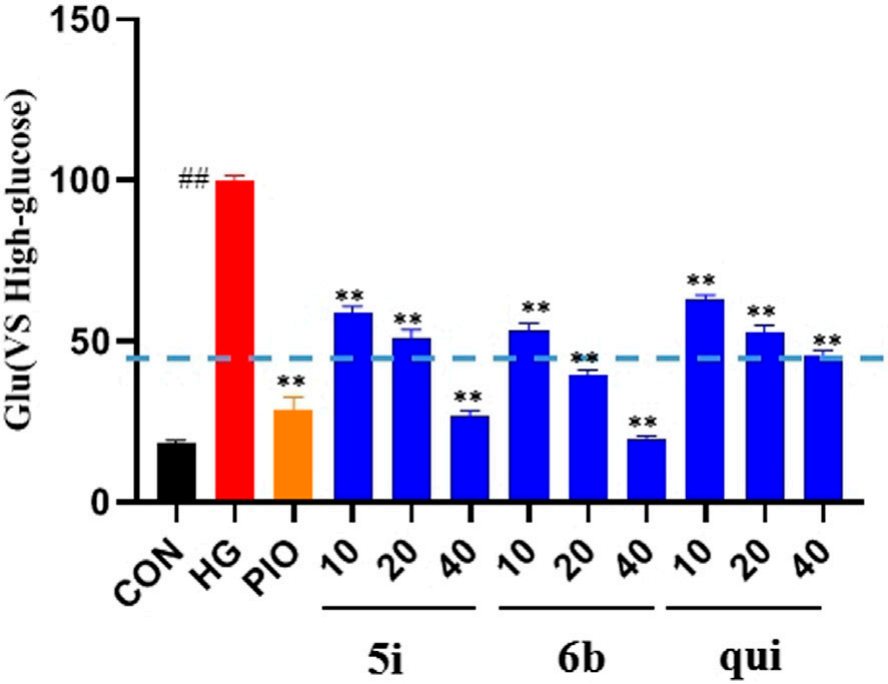
Effect of compounds 5i and 6b on insulin resistance in LO2 cells. 10, 20, and 40 μM of the compounds 5i and 6b were administered. In the positive control group, 40 μM of Pioglitazone was administered for 48 h (# *vs*. CON, ##*p* < 0.01, * *vs*. HG, **p < 0.01, PIO, Pioglitazone; HG, High Glucose, qui: lead compound).

The effect of the 30 derivatives 5a–p and 6a–n on the glucose content of high-glucose-induced LO2 cells was examined. The experimental results showed that LO2 cells in the model group developed insulin resistance after 48 h of high glucose induction. Compared with the model group, glucose levels were significantly lower in the administered group after the addition of compounds 5a, 5d, 5e, 5g, 5h, 5i, 5j, 5m, 5n, 5o, 5p, 6b, 6f, 6h, 6i, and 6m. Compounds 5b, 5g, 5i, 5n, and 6b demonstrated stronger glucose-lowering effects on LO2 cells than the lead compound (3-phenylquinoxalin-2(1*H*)-one). Of these, compounds 5i and 6b displayed better glucose-lowering activity than the positive agent Pioglitazone (Figure 2). Further studies revealed that compounds 5i and 6b were capable of reducing glucose levels and alleviating insulin resistance in a concentration-dependent manner. Moreover, both were more active than the lead compound as well as Pioglitazone (Figure 3), and compound 5i was more active than 6b.

### 2.5 Analysis of the effects of compounds 5i and 6b on the ROS content of LO2 cells

**FIGURE 4:**
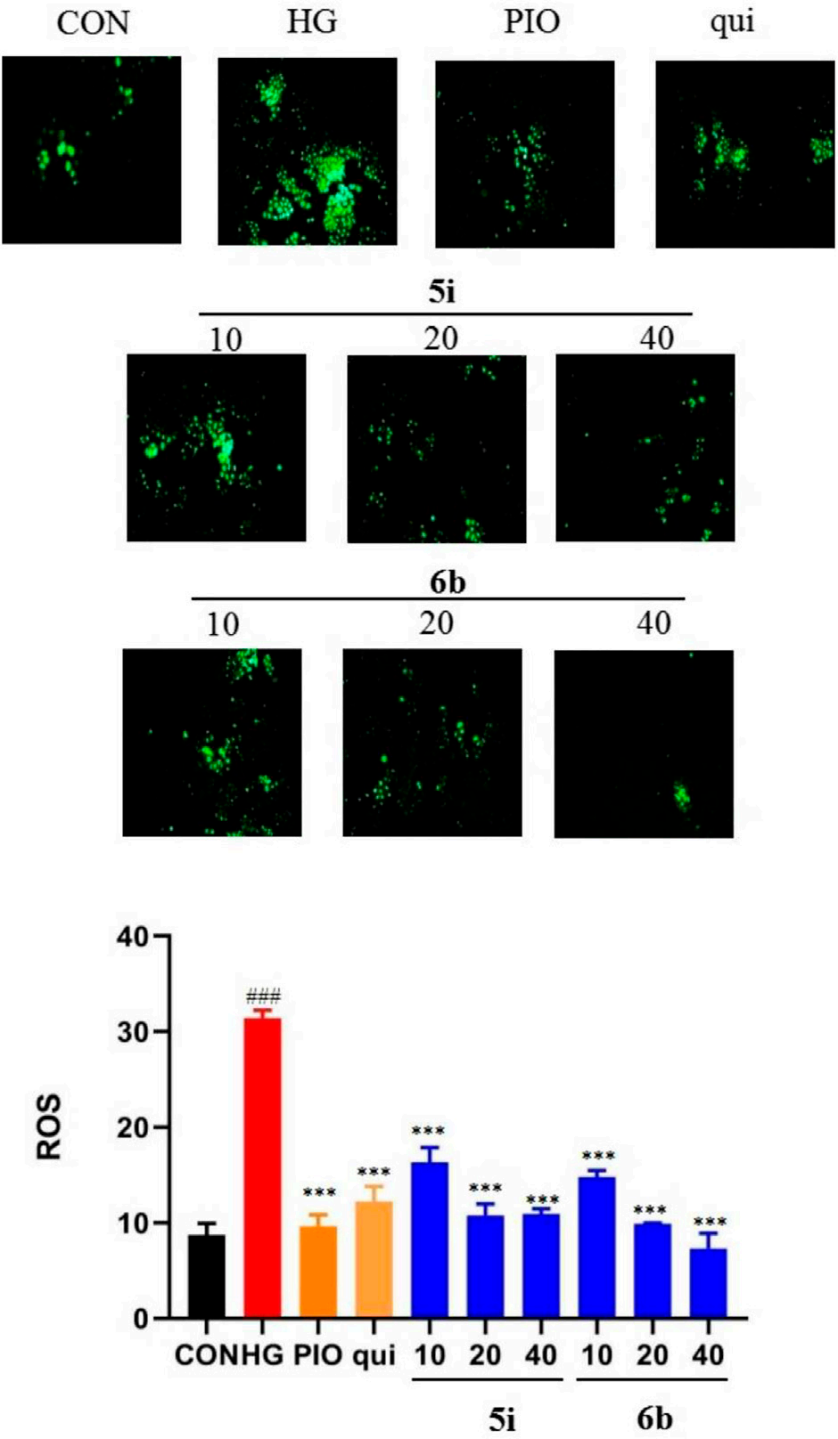
Effect of compounds 5i and 6b on the ROS content in LO2 cells. LO2 cells were treated with compounds 5i and 6b (10, 20, 40 μM) dissolved in DMSO for 48 h. The ROS content was measured using the DCFH-DA fluorescent probe assay, using a fluorescence microscope (# *vs*. CON, ###*p* < 0.01, * *vs*. HG, ***p < 0.001, 488 nm excitation wavelength and 525 nm emission wavelength; PIO, Pioglitazone; HG, High Glucose, qui: lead compound).

The effect of compounds 5i and 6b on the ROS content of LO2 cells was evaluated, and the results showed that the ROS content was significantly higher in the model group than that in the normal group (*p* < 0.01). Compounds 5i and 6b inhibited ROS production in a concentration-dependent manner compared with the model group (Figure 4), compared to the lead compound and positive control drug, and the inhibitory effect of compound 6b was better than that of 5i.

### 2.6 Analysis of the effects of compounds 5i and 6b on malondialdehyde (MDA), superoxide dismutase (SOD), and catalase (CAT) contents in LO2 cells

**FIGURE 5:**
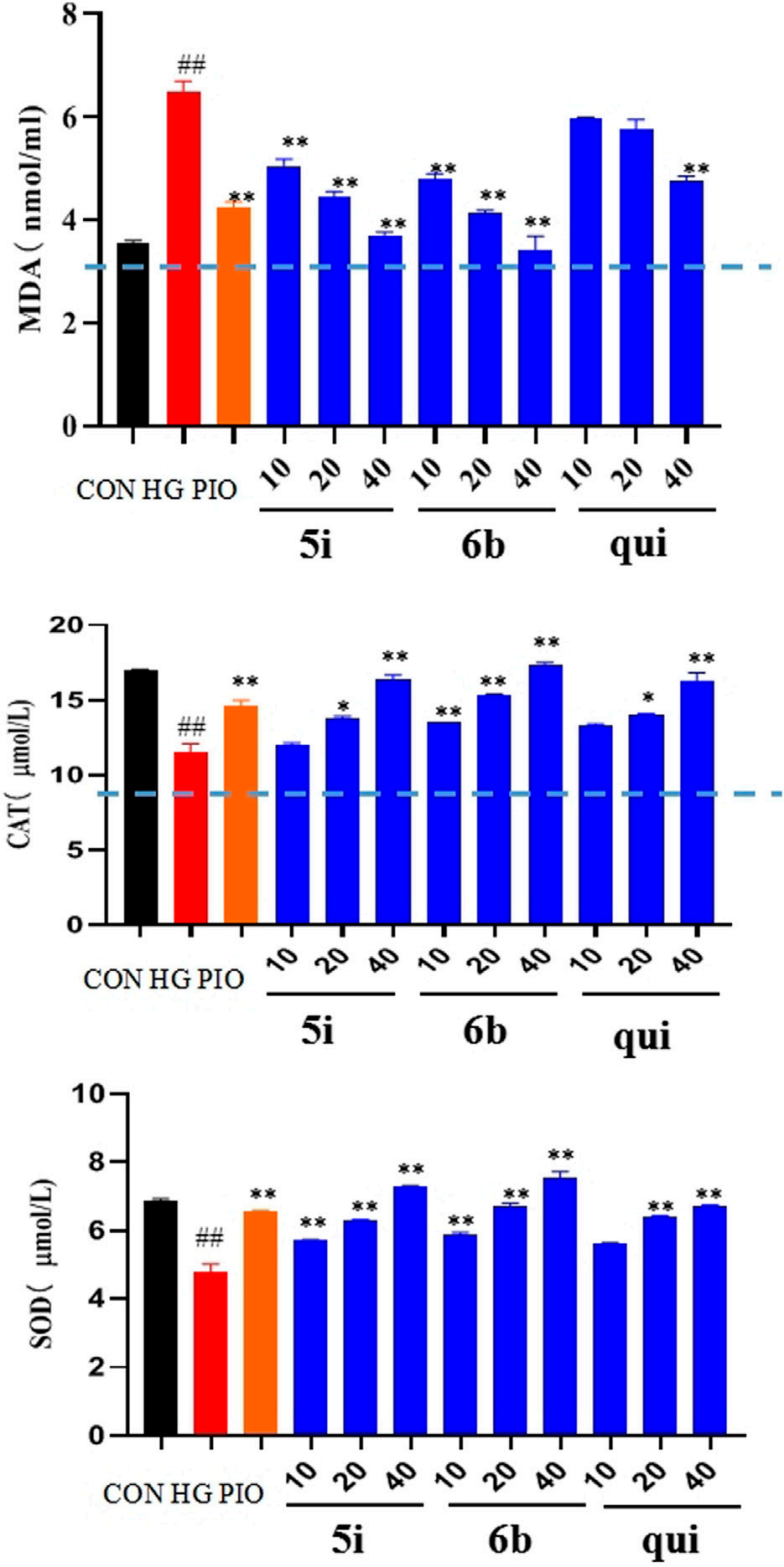
Effects of compounds 5i and 6b on the contents of MDA, SOD, and CAT in LO2 cells. LO2 cells were treated with compounds 5i and 6b (10, 20, 40 μM) dissolved in DMSO for 48 h. MDA, SOD, and CAT contents were determined using the appropriate ELISA kits. (# *vs*. CON, ##*p* < 0.01, * *vs*. HG, *p < 0.05, **p < 0.01, PIO, Pioglitazone; HG, High Glucose, qui: lead compound).

Compounds 5i and 6b were tested to determine whether they scavenged ROS by affecting the MDA, SOD, and CAT systems. The findings implied that the MDA content was significantly higher and the SOD and CAT contents were significantly lower in the model group compared with the normal group. The administration groups (5i and 6b) exhibited a concentration-dependent decrease in MDA levels and an increase in SOD and CAT levels compared with the model group (Figure 5), with compound 6b showing a better effect than 5i. This observation agrees with the experimental results obtained in 2.5.

### 2.7 Analysis of the docking results of compounds 5i and 6b with diabetes target molecules

**FIGURE 6:**
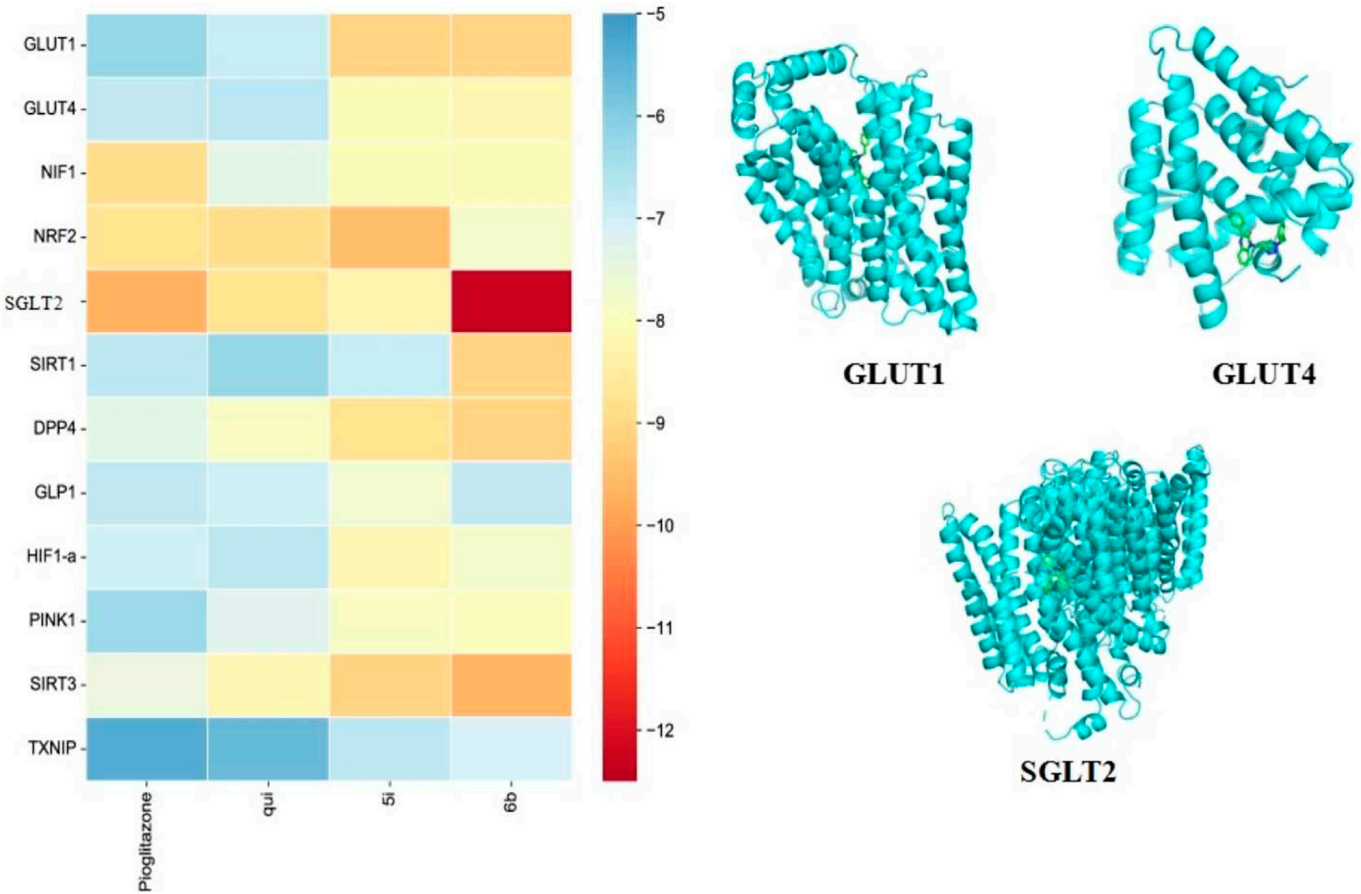
Docking results of the compounds 5i and 6b with selected target molecules in diabetes. Note: (Heat map demonstrating the docking-binding energy of compounds 5i and 6b with some of the target molecules in diabetes, and the docking diagram depict the optimal binding of compound 6b to GLUT1, GLUT4, and SGLT2, respectively. The blue helical structure represents the structural formula of the protein after the removal of water and heteroatoms. The green part represents small molecule ligands).

Molecular docking enables efficient and convenient discovery and screening of drug targets. The results showed that compound 6b possessed high binding energies of −12.3, −9.1, −9.1, −9.1, and −9.6 kcal/mol toward SGLT2, GLUT1, DPP4, SIRT1, and SIRT3, respectively. On the contrary, the binding energies of compound 5i toward SGLT2, GLUT1, DPP4, SIRT1, and SIRT3 were −8.3, −9.1, −8.8, −6.9, and −9.1 kcal/mol, respectively. Hence, compound 6b was found to bind better than 5i for most of the screening targets, which agrees with the drug screening results. Compound 6b exhibited the strongest binding capacity toward SGLT2 (Figure 6); hence, this compound could be a potential target that exerts hypoglycemic effects. Moreover, SGLT2, GLUT4, and GLUT1 are all glucose transporters. Therefore, whether compound 6b could affect GLUT1, GLUT4, and SGLT2 was further tested.

### 2.8 Effect of compounds 5i and 6b on 2-NBDG uptake in HK-2 cells

**FIGURE 7:**
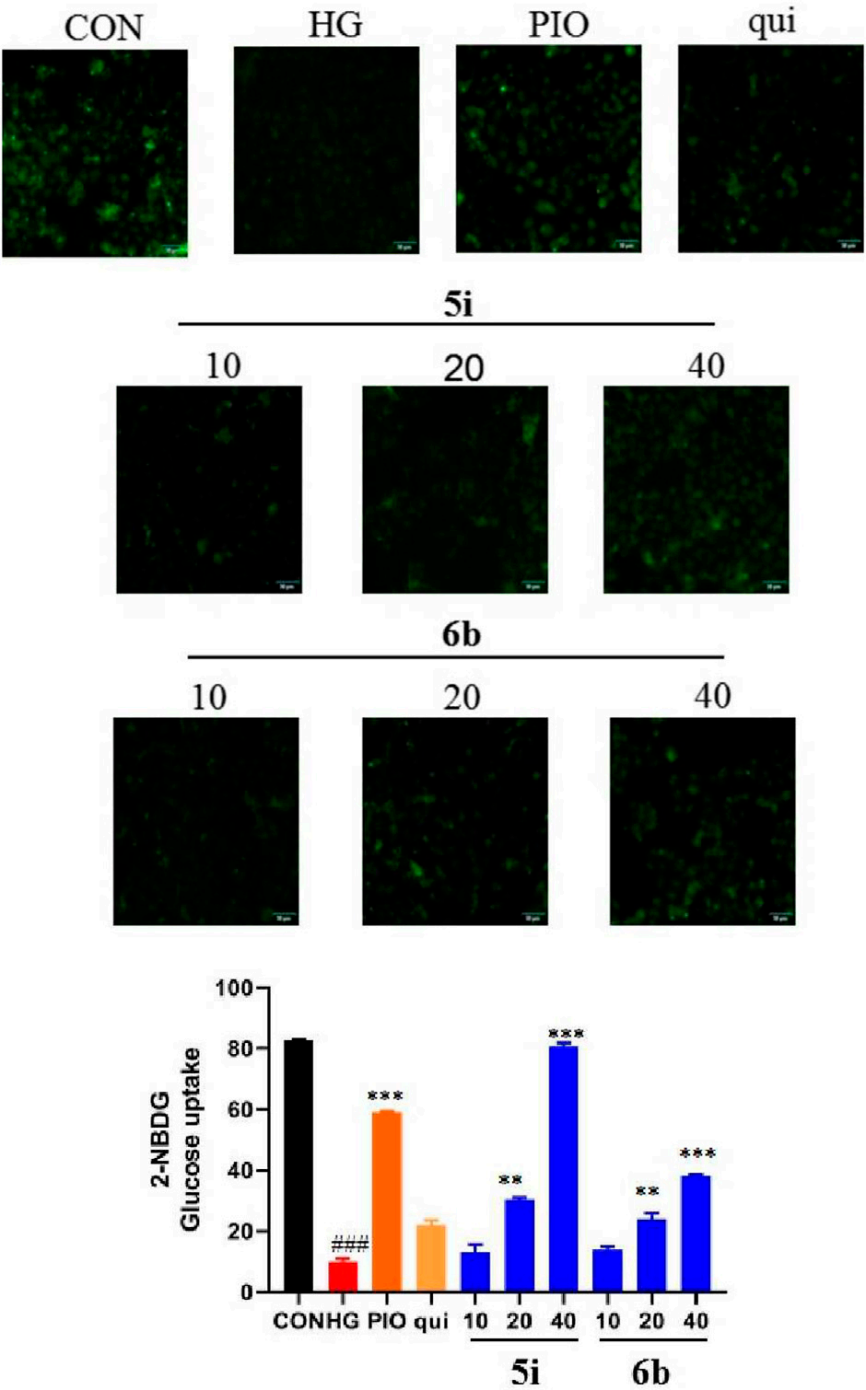
Compound 5i and 6b inhibits 2-NBDG uptake in HK-2 cells. HK-2 cells were seeded in a confocal laser dish for 24 h. These were then treated with compound 5i (10, 20, 40 μM), compound 6b (10, 20, 40 μM), Pioglitazone (40 μM) and the lead compound (40 μM) in serum-free medium for 6 h (# *vs*. CON, ###*p* < 0.001, * *vs*. HG, **p < 0.01, ***p < 0.001; PIO, Pioglitazone; HG, High Glucose, qui: lead compound).

The fluorescent glucose analogue, 2-NBDG, could be used to capture glucose uptake in cells pre-incubated with the compound. The results showed that compounds 5i and 6b increased 2-NBDG uptake in HK-2 cells at 40 μM (*p* < 0.001; Figure 7). Compounds 5i and 6b increased the glucose uptake of HK-2 cells and increased the hypoglycaemic activity in a concentration-dependent manner, superior to the lead compound.

### 2.9 Analysis of the effects of compound 6b on the expression of GLUT4 and PPAR-γ

**FIGURE 8:**
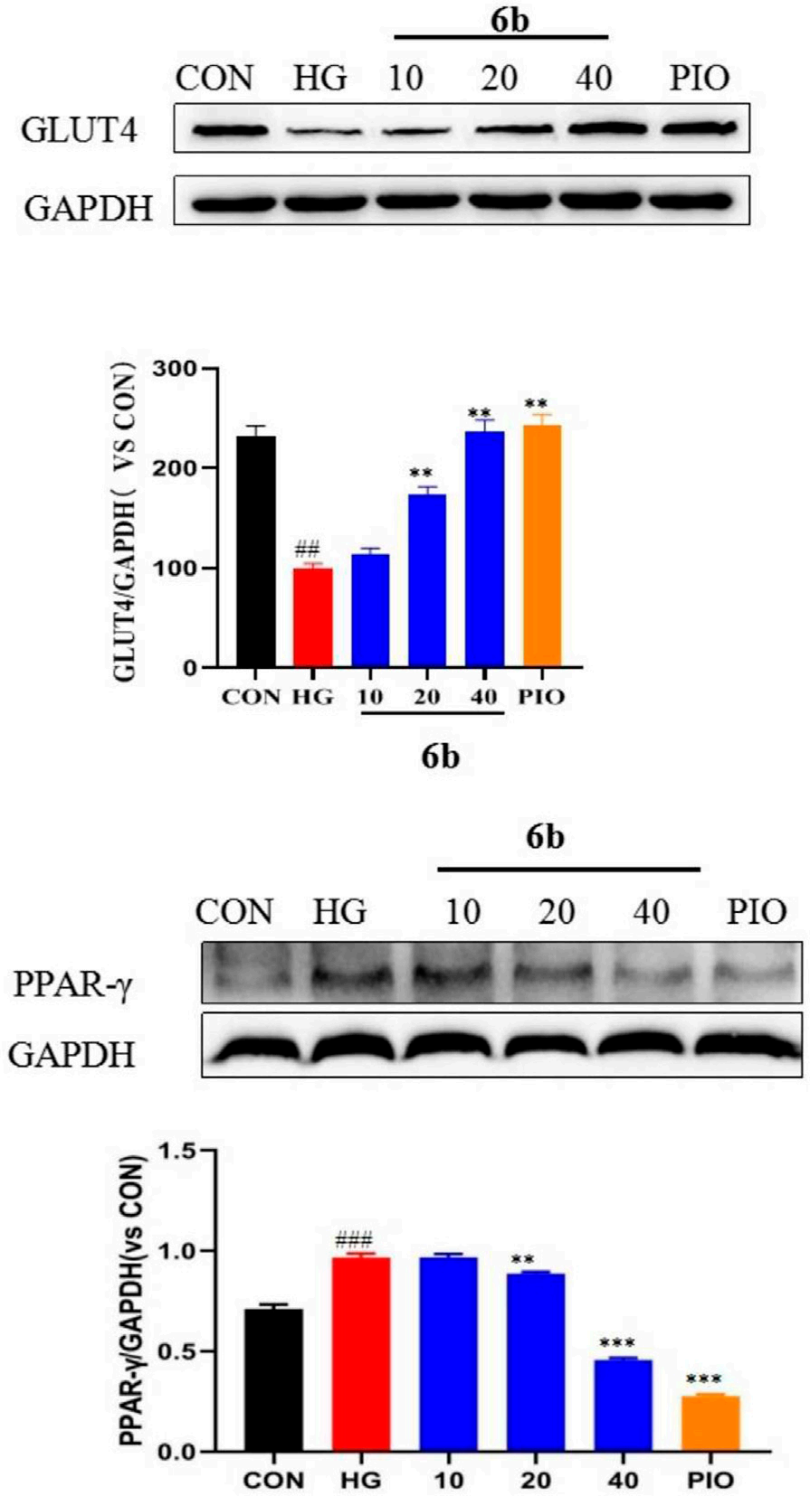
Effect of compound 6b on the PPAR-γ and GLUT4 content in LO2 cells. LO2 cells were treated with the compounds 6b (10, 20, 40 μM) dissolved in DMSO for 48 h. The expression of PPAR-γ and GLUT4 were analyzed by Western blot. (# *vs*. CON, ##*p* < 0.01, ###*p* < 0.01 * *vs*. HG, **p < 0.01, ***p < 0.001; PIO, Pioglitazone; HG, High Glucose).

Upon comprehensively analyzing the above experimental results, compound 6b was found to be the best among the 30 newly synthesized derivatives in alleviating insulin resistance and OS. Therefore, a Western blot assay was performed to validate the targets obtained via molecular docking screening for compound 6b. The results indicated that the expression of GLUT4 protein was significantly reduced in the high-glucose-induced LO2 cell model group. However, the expression was significantly increased in a concentration-dependent manner after treating the cells with compound 6b (Figure 8). This finding asserts that compound 6b is capable of improving glucose transport and insulin resistance via GLUT4. Pioglitazone, a well known insulin sensitizer drug, is directly or indirectly activated by its primary target, peroxisome proliferator-activated receptor-γ (PPAR-γ). We therefore tested the biological evaluation of quinoxaline derivatives against PPAR-γ *in vitro*. The results indicated that the expression of PPAR-γ protein was significantly increased in the high-glucose–induced LO2 cell model group. However, the expression was significantly reduced in a concentration-dependent manner after treating the cells with compound 6b (Figure 8).

### 2.10 Analysis of the effects of compound 6b on the expressions of GLUT1 and SGLT2

**FIGURE 9:**
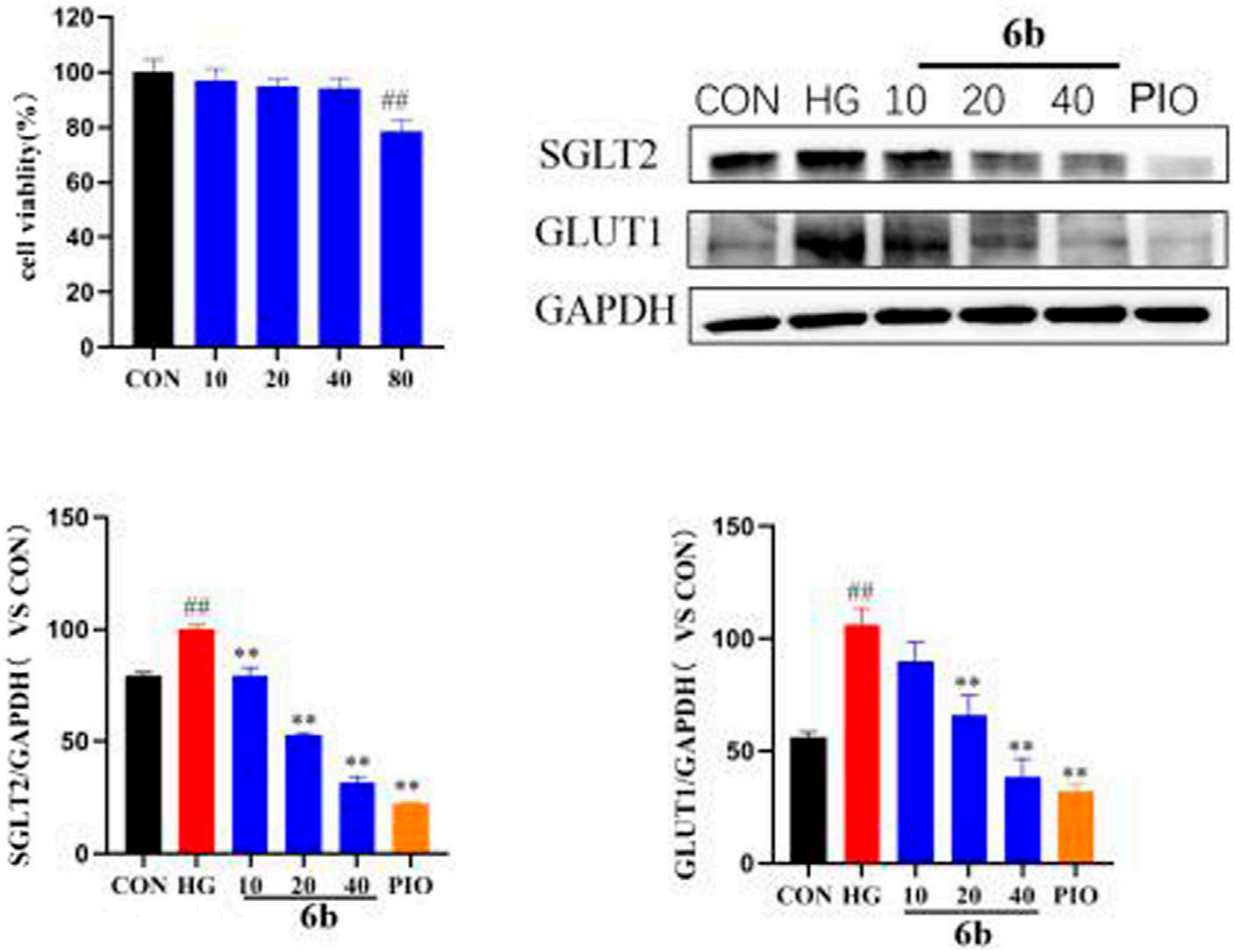
MTT results of compound 6b on HK-2 cells and the effects of SGLT2 and GLUT1 content in HK-2 cells. HK-2 cells were treated with the compounds 6b (10, 20, 40 μM) dissolved in DMSO for 48 h. The expression of SGLT2 and GLUT1 were analyzed by Western blot. (# *vs*. CON, ##*p* < 0.01, * *vs*. HG, **p < 0.01, PIO, Pioglitazone; HG, High Glucose).

Molecular docking alluded that SGLT2 and GLUT1 are likely to be key targets of compound 6b. SGLT2 and GLUT1 are glucose transporters that are found mainly in the kidney. By inhibiting the expressions of SGLT2 and GLUT1, compound 6b reduced glucose reabsorption and allowed it to be excreted in the urine, thereby lowering the blood glucose level, reducing the renal load, and protecting the kidney. High-glucose-induced HK-2 cells (human renal cortical proximal tubular epithelial cells) were selected to establish the model. The effect of compound 6b on the expressions of SGLT2 and GLUT1 was examined using the HK-2 hyperglycemic cell model after assessing the toxicity of compound 6b toward HK-2 cells (at 40 μM, compound 6b did not exhibit significant toxicity toward HK-2 cells). The results showed that the expressions of SGLT2 and GLUT1 were significantly downregulated in a concentration-dependent manner after treatment with compound 6b in the model group of high-glucose-induced HK-2 cells. Hence, compound 6b may modify diabetes via interactions of SGLT2 and GLUT1 proteins (Figure 9).

## 3 Conclusion

In this study, 30 novel derivatives of quinoxalinone were synthesized, profiled via ^1^H-NMR, ^13^C-NMR, and HRMS assays, and assessed for hypoglycemic activity. MTT bioassay indicated all of the synthesised compounds were not toxic or less toxic to LO2 cells at concentrations below 40 μM. However, at concentrations up to 80 μM, some compounds still showed no significant toxicity. Most of the derivatives exhibited hypoglycemic activity. A model of insulin resistance is established by high glucose induction in LO2 cells, which is used to detect the effect of synthetic derivatives on changes in the level of glucose in the model. The study showed that, of the tested compounds, the activities of 5i and 6b were better than those of the lead compounds and comparable to that of the positive control Pioglitazone. Overexpression of ROS is an influential factor in the development of diabetes and a key factor in the development of insulin resistance. Based on the results of this study, we found that compounds 5i and 6b inhibited the production of ROS in cells in a concentration-dependent manner, thereby inhibiting OS. MDA, SOD and CAT are a kind of radical enzymatic defense systems existing in the body, which can remove free radicals and protect the body from damage. Compounds 5i and 6b decreased MDA levels and increased SOD and CAT levels in a concentration-dependent manner, scavenging ROS and improving OS levels. GLUT4, SGLT2, and GLUT1 are the main intracellular transport proteins for glucose uptake, and their functional status is closely related to the disruption of glucose metabolism in diabetic cells and the development and progression of their complications. From our experimental results, it has appeared that the levels of GLUT1 was significantly higher and the levels of GLUT4 was significantly lower in the high glucose model. After treatment with compound 6b, the levels of GLUT1 decreased significantly and the levels of GLUT4 increased significantly. Compound 6b may exert hypoglycemic effects by improving OS levels and regulating GLUT4, SGLT2, and GLUT1 protein expression. These findings suggest that compound 6b is a potential lead compound with hypoglycaemic activity.

## 4 Experimental section

### 4.1 General procedures

Chemicals and spectral-grade solvents used in this study were purchased from commercial sources and not subjected to any additional purification. Standard protocols were applied to dry and utilize solvents. TLC was used to monitor all chemical reactions, and the plates were visualized via ultraviolet light exposure (254 and 365 nm). Flash column chromatography over silica gels (200–300 mesh) was performed to purify the compounds. An RY-1 MP apparatus was used to measure the melting points of the compounds. An AV-300 (Bruker, Switzerland) instrument was used to assess uncorrected ^1^H-NMR and ^13^C-NMR spectra, with tetramethylsilane serving as the reference standard. Chemical shifts were recorded as parts per million in comparison with tetramethylsilane. A Q Exactive instrument (Thermo Scientific, United States) was employed for mass spectra measurements.

### 4.2 Synthesis procedures

#### 4.2.1 Preparation of 3-phenylquinoxalin-2(1H)-one (1a)

In this procedure, 1,2-diaminobenzene (21.6 g, 200 mmol) was dissolved in acetic acid (10 ml) under heated conditions, the heating was stopped when dissolved, and it was cooled to room temperature. Methyl phenylglyoxylate (12.5 g, 70 mmol) was dissolved in ethanol (50 ml) and slowly added dropwise to the above solution. The reaction mixture was stirred for 30 min at room temperature and then heated to reflux for 1 h. When the mixture cooled down, the crystals were separated via filtration and washed with n-hexane. Recrystallization was achieved using anhydrous ethanol to yield yellow needle-like crystals of compound 1a.

#### 4.2.2 Synthesis of 1-propargyl-3-phenylquinolin-2(1H)-one (2a)

Compound 1a (5.0 g, 1 mmol), potassium carbonate (5.144 g, 2 mmol), and propargyl bromide (3.326 g, 1.4 mmol) were dissolved in acetone (50 ml) and refluxed at 80°C. The reaction was monitored at TLC. After the completion of the reaction, the solvent was evaporated and then extracted with dichloromethane. The combined organic layers were dried with anhydrous sodium sulfate, filtered, and distilled under reduced pressure to obtain the crude product. It was subjected to silica gel column chromatography to obtain the purified product of compound 2a for the next step of the synthesis.

#### 4.2.3 Synthesis of intermediate compound 3a-p

Aniline (100 mg, 1 mmol) of the corresponding substituent was dissolved in 10% HCl (15 ml) in Stir under ice bath conditions (0°C) and slowly added sodium nitrite (1.2 mmol) aqueous solution. After stirring for 30 min, aqueous sodium azide solution (1.3 mmol) was added slowly dropwise and the reaction was monitored by TLC for 2–4 h. After the completion of the reaction, the solvent was extracted with dichloromethane. The combined organic layers were dried with anhydrous sodium sulfate, filtered, and distilled under reduced pressure to obtain the crude product.

#### 4.2.4 Synthesis of intermediate compound 4a-n

Benzyl chloride (1 mmol) of the corresponding substituent was dissolved in 30 ml of N,N-dimethylacetamide, followed by the addition of sodium azide (1.3 mmol), refluxed at 110°C for 3–4 h, and the reaction process was monitored under TLC. After the completion of the reaction, the solvent was evaporated and then extracted with dichloromethane. The combined organic layers were dried with anhydrous sodium sulfate, filtered, and distilled under reduced pressure to obtain the crude product.

#### 4.2.5 Synthesis of target compounds 5a-p, 6a-n

Intermediates 3a-p, 4a-n (1.2 mmol) were dissolved in a mixture of water:n-butanol (1:1, V/V), respectively. Compound 2a (100 mg, 1 mmol), anhydrous copper sulfate (10 mg, 0.1 mmol) and sodium ascorbate (16 mg, 0.2 mmol) were added separately and left at room temperature overnight. The reaction process was monitored under TLC. After the completion of the reaction, the solvent was evaporated and then extracted with dichloromethane. The combined organic layers were dried with anhydrous sodium sulfate, filtered, and distilled under reduced pressure to obtain the crude product. It was subjected to silica gel column chromatography to obtain the purified product of compound 5a-p, 6a-n.

***1-*** ((***1-*** (***2-fluorophenyl***) ***-1H-1,2,3-triazol-4-yl***) ***methyl***) ***-3-phenylquinoxalin-2*** (***1H***) ***-one*** (***5a***) Pale yellow powder; yield 69.4%, m. p. = 158.7°C–160.2°C; ^1^H NMR (300 MHz, CDCl_3_) *δ* 8.37—8.28 (m, 2H), 8.26 (d, *J* = 2.7 Hz, 1H), 8.03—7.93 (m, 2H), 7.90—7.83 (m, 1H), 7.67—7.60 (m, 1H), 7.53—7.46 (m, 3H), 7.43—7.36 (m, 2H), 7.32—7.28 (m, 1H), 7.23 (d, *J* = 1.3 Hz, 1H), 5.71 (s, 2H, -CH_2_). ^13^C NMR (75 MHz, CDCl_3_) *δ* 154.92, 154.43, 153.78, 151.59, 142.73, 135.73, 133.23, 132.30, 130.59, 130.38, 130.32, 130.23, 129.37, 128.03, 125.16, 125.07, 125.03, 124.78, 123.97, 117.05, 116.78, 114.53, 38.04. HRMS (ESI) m/z calcd for C_23_H_17_FN_5_O^+^ (M + H)^+^ 397.1339, obtained 398.1413.

***1-*** ((***1-*** (***3-fluorophenyl***) ***-1H-1,2,3-triazol-4-yl***) ***methyl***) ***-3-phenylquinoxalin-2*** (***1H***) ***-one*** (***5b***) Pale yellow powder; yield 72.8%, m. p. = 179.4°C–182.5°C; ^1^H NMR (300 MHz, CDCl_3_) δ 8.41—8.22 (m, 2H), 8.18 (s, 1H), 7.96 (t, *J* = 8.0 Hz, 2H), 7.66—7.58 (m, 1H), 7.56—7.30 (m, 7H), 7.11 (s, 1H), 5.68 (s, 2H, -CH_2_). ^13^C NMR (75 MHz, CDCl_3_) δ 164.52, 161.23, 154.49, 153.71, 143.31, 135.67, 133.23, 132.19, 131.09, 130.98, 130.63, 130.37, 129.34, 128.05, 124.05, 122.00, 115.82, 115.63, 115.59, 115.54, 114.43, 108.31, 107.96, 38.08. HRMS (ESI) m/z calcd for C_23_H_17_FN_5_O^+^ (M + H)^+^ 397.1339, obtained 398.1414.

***1-*** ((***1-*** (***4-fluorophenyl***) ***-1H-1,2,3-triazol-4-yl***) ***methyl***) ***-3-phenylquinoxalin-2*** (***1H***) ***-one*** (***5c***) Pale yellow powder; yield 73.4%, m. p. = 194.6°C–195.9°C; ^1^H NMR (300 MHz, CDCl_3_) *δ* 8.36—8.26 (m, 2H), 8.13 (s, 1H), 8.02—7.92 (m, 2H), 7.70—7.59 (m, 3H), 7.54—7.46 (m, 3H), 7.42—7.35 (m, 1H), 7.21—7.13 (m, 2H, -CH_2_), 5.69 (s, 2H, -CH_3_). ^13^C NMR (75 MHz, CDCl_3_) *δ* 163.99, 160.68, 154.52, 153.73, 143.20, 135.69, 133.24, 132.94, 132.22, 130.62, 130.38, 129.34, 128.05, 124.03, 122.41, 122.29, 122.19, 122.17, 116.72, 116.42, 114.47, 38.13. HRMS (ESI) m/z calcd for C_23_H_17_FN_5_O^+^ (M + H)^+^ 397.1339, obtained 398.1420.

***1-*** ((***1-*** (***2-chlorophenyl***) ***-1H-1,2,3-triazol-4-yl***) ***methyl***) ***-3-phenylquinoxalin-2*** (***1H***) ***-one*** (***5d***) Pale yellow powder; yield 76.6%, m. p. = 167.9°C–169.7°C; ^1^H NMR (300 MHz, CDCl_3_) *δ* 8.30 (dd, *J* = 6.7, 3.0 Hz, 2H), 8.18 (s, 1H), 8.05—7.92 (m, 2H), 7.68—7.61 (m, 1H), 7.58—7.45 (m, 5H), 7.46—7.35 (m, 3H), 5.71 (s, 2H, -CH_2_). ^13^C NMR (75 MHz, CDCl_3_) *δ* 154.43, 153.74, 142.11, 135.79, 135.72, 134.55, 133.22, 132.32, 130.75, 130.63, 130.37, 130.32, 129.36, 128.50, 128.03, 127.73, 127.59, 125.98, 123.99, 114.57, 38.10. HRMS (ESI) m/z calcd for C_23_H_17_ClN_5_O^+^ (M + H)^+^ 413.1043, obtained 414.1119.

***1-((1-(3-chlorophenyl)-1H-1,2,3-triazol-4-yl)methyl)-3-phenylquinoxalin-2(1H)-one (5e)*** Pale yellow powder; yield 75.6%, m. p. = 187.1°C–189.1°C; ^1^H NMR (300 MHz, CDCl_3_) *δ* 8.39—8.23 (m, 2H), 8.18 (s, 1H), 8.03—7.91 (m, 2H), 7.74 (s, 1H), 7.67—7.48 (m, 5H), 7.41 (q, *J* = 8.3 Hz, 3H), 5.69 (s, 2H, -CH_2_). ^13^C NMR (75 MHz, CDCl_3_) *δ* 169.13, 154.50, 153.71, 143.33, 137.58, 137.45, 135.65, 135.42, 133.23, 132.16, 130.66, 130.39, 129.34, 128.82, 128.07, 124.07, 122.01, 120.60, 118.25, 114.42, 38.09. HRMS (ESI) m/z calcd for C_23_H_17_ClN_5_O^+^ (M + H)^+^ 413.1043, obtained 414.1126.

***1-*** ((***1-*** (***4-chlorophenyl***) ***-1H-1,2,3-triazol-4-yl***) ***methyl***) ***-3-phenylquinoxalin-2*** (***1H***) ***-one*** (***5f***) Pale yellow powder; yield 77.6%, m. p. = 205.8°C–208.4°C; ^1^H NMR (300 MHz, CDCl_3_) *δ* 8.35—8.27 (m, 2H), 8.16 (s, 1H), 8.00—7.93 (m, 2H), 7.66—7.59 (m, 3H), 7.53—7.48 (m, 3H), 7.48—7.40 (m, 3H), 7.37 (dd, *J* = 7.1, 0.9 Hz, 1H), 5.68 (s, 2H, -CH_2_). ^13^C NMR (75 MHz, CDCl_3_) *δ* 154.49, 153.70, 143.29, 135.66, 135.11, 134.52, 133.22, 132.18, 130.63, 130.38, 129.77, 129.34, 128.06, 124.06, 121.95, 121.47, 114.45, 38.09. HRMS (ESI) m/z calcd for C_23_H_17_ClN_5_O^+^ (M + H)^+^ 413.1043, obtained 414.1121.

***1-*** ((***1-*** (***2-bromophenyl***) ***-1H-1,2,3-triazol-4-yl***) ***methyl***) ***-3-phenylquinoxalin-2*** (***1H***) ***-one*** (***5g***) Pale yellow powder; yield 75.6%, m. p. = 145.7°C–147.3°C; ^1^H NMR (300 MHz, CDCl_3_) *δ* 8.37—8.25 (m, 2H), 8.13 (s, 1H), 8.04—7.92 (m, 2H), 7.74—7.69 (m, 1H), 7.67—7.60 (m, 1H), 7.52—7.32 (m, 7H), 5.71 (s, 2H, -CH_2_). ^13^C NMR (75 MHz, CDCl_3_) *δ* 154.48, 153.77, 142.12, 136.25, 135.77, 133.83, 133.28, 132.37, 131.21, 130.68, 130.42, 130.39, 129.43, 128.37, 128.09, 128.06, 126.09, 124.05, 118.49, 114.65, 38.18. HRMS (ESI) m/z calcd for C_23_H_17_BrN_5_O^+^ (M + H)^+^ 457.0538, obtained 458.0691.

***1-*** ((***1-*** (***3-bromophenyl***) ***-1H-1,2,3-triazol-4-yl***) ***methyl***) ***-3-phenylquinoxalin-2*** (***1H***) ***-one*** (***5h***) Pale yellow powder; yield 72.6%, m. p. = 176.5°C–178.7°C; ^1^H NMR (300 MHz, CDCl_3_) *δ* 8.35—8.27 (m, 2H), 8.17 (s, 1H), 7.99—7.92 (m, 2H), 7.89 (t, *J* = 1.9 Hz, 1H), 7.66—7.59 (m, 2H), 7.56—7.48 (m, 4H), 7.42—7.37 (m, 1H), 7.37—7.32 (m, 1H), 5.68 (s, 2H, -CH_2_). ^13^C NMR (75 MHz, CDCl^3^) *δ* 154.56, 153.77, 143.38, 137.58, 135.71, 133.33, 133.28, 132.23, 131.81, 130.96, 130.70, 130.45, 129.40, 128.12, 124.13, 123.46, 123.19, 122.06, 118.81, 114.48, 38.15. HRMS (ESI) m/z calcd for C_23_H_17_BrN_5_O^+^ (M + H)^+^ 457.0538, obtained 458.0619.

***1-*** ((***1-*** (***4-bromophenyl***) ***-1H-1,2,3-triazol-4-yl***) ***methyl***) ***-3-phenylquinoxalin-2*** (***1H***) ***-one*** (***5i***) Pale yellow powder; yield 69.3%, m. p. = 231.2°C–232.7°C; ^1^H NMR (300 MHz, CDCl_3_) *δ* 8.38—8.24 (m, 2H), 8.16 (s, 1H), 8.02—7.91 (m, 2H), 7.67—7.54 (m, 5H), 7.54—7.45 (m, 3H), 7.38 (t, *J* = 7.5 Hz, 1H), 5.68 (s, 2H, -CH_2_). ^13^C NMR (75 MHz, CDCl_3_) *δ* 154.60, 153.85, 145.58, 143.43, 135.73, 133.31, 133.07, 132.82, 132.25, 132.08, 130.72, 130.46, 129.39, 128.14, 124.14, 121.96, 121.79, 114.51, 38.18. HRMS (ESI) m/z calcd for C_23_H_17_BrN_5_O^+^ (M + H)^+^ 457.0538, obtained 458.0615.

***1-*** ((***1-2-Tolyl-1H-1,2,3-triazole***) ***-4-methyl***) ***-3-phenylquinoxaline-2*** (***1H***) ***-one*** (***5j***) Pale yellow powder; yield 70.2%, m. p. = 169.5°C–172.0°C; ^1^H NMR (300 MHz, CDCl_3_) *δ* 8.35—8.27 (m, 2H), 8.03 (d, *J* = 8.4 Hz, 1H), 7.99—7.92 (m, 2H), 7.68—7.61 (m, 1H), 7.53—7.47 (m, 3H), 7.43—7.36 (m, 2H), 7.37—7.30 (m, 2H), 7.29—7.24 (m, 2H), 5.71 (s, 2H, -CH_2_), 2.19 (s, 3H, -CH_3_). ^13^C NMR (75 MHz, CDCl_3_) *δ* 155.35, 154.52, 153.75, 144.88, 142.22, 136.18, 135.80, 133.43, 133.30, 132.42, 131.45, 130.69, 130.41, 129.83, 129.45, 128.10, 126.75, 125.77, 125.42, 124.05, 114.68, 38.27, 17.93. HRMS (ESI) m/z calcd for C_24_H_20_N_5_O^+^ (M + H)^+^ 393.15896, obtained 394.1667.

***1-*** ((***1-3-Tolyl-1H-1,2,3-triazole***) ***-4-methyl***) ***-3-phenylquinoxaline-2*** (***1H***) ***-one*** (***5k***) Pale yellow powder; yield 71.5%, m. p. = 175.9°C–177.4°C; ^1^H NMR (300 MHz, CDCl_3_) *δ* 8.38—8.26 (m, 2H), 8.16 (s, 1H), 8.02—7.92 (m, 2H), 7.66—7.59 (m, 1H), 7.54—7.44 (m, 5H), 7.41—7.37 (m, 1H), 7.36—7.31 (m, 1H), 7.21 (d, *J* = 7.7 Hz, 1H), 5.69 (s, 2H, -CH_2_), 2.40 (s, 3H, -CH_3_). ^13^C NMR (75 MHz, CDCl_3_) *δ* 154.60, 153.84, 143.17, 143.01, 139.90, 136.68, 135.81, 133.30, 132.32, 130.71, 130.42, 129.57, 129.43, 128.14, 124.09, 122.13, 122.11, 121.03, 117.51,114.63, 38.28, 21.32. HRMS (ESI) m/z calcd for C_24_H_20_N_5_O^+^ (M + H)^+^ 393.15896, obtained 394.1665.

***1-*** ((***1-4-Tolyl-1H-1,2,3-triazole***) ***-4-methyl***) ***-3-phenylquinoxaline-2*** (***1H***) ***-one*** (***5l***) Pale yellow powder; yield 72.3%, m. p. = 227.6°C–229.3°C; ^1^H NMR (300 MHz, CDCl_3_) *δ* 8.36—8.27 (m, 2H), 8.12 (s, 1H), 8.00 (d, *J* = 7.7 Hz, 1H), 7.94 (dd, *J* = 8.0, 1.4 Hz, 1H), 7.65—7.58 (m, 1H), 7.57—7.47 (m, 5H), 7.41—7.35 (m, 1H), 7.28—7.24 (m, 2H), 5.68 (s, 2H, -CH_2_), 2.38 (s, 3H, -CH_3_). ^13^C NMR (75 MHz, CDCl_3_) *δ* 154.60, 143.45, 142.95, 138.97, 135.80, 134.47, 134.39, 133.29, 132.33, 130.70, 130.40, 130.14, 129.42, 128.12, 124.07, 122.01, 120.33, 114.65, 38.28, 21.02. HRMS (ESI) m/z calcd for C_24_H_20_N_5_O^+^ (M + H)^+^ 393.441, obtained 394.1672.

***1-*** ((***1-*** (***2-methoxyphenyl***) ***-1H-1,2,3-triazol-4-yl***) ***methyl***) ***-3-phenylquinoxalin-2*** (***1H***) ***-one*** (***5m***) Brown powder; yield 68.6%, m. p. = 176.6°C–179.1°C; ^1^H NMR (300 MHz, CDCl_3_) *δ* 8.37—8.23 (m, 3H), 8.05 (d, *J* = 8.5 Hz, 1H), 7.93 (dd, *J* = 8.0, 1.3 Hz, 1H), 7.70—7.58 (m, 2H), 7.53—7.45 (m, 3H), 7.40—7.33 (m, 2H), 7.03 (t, *J* = 7.9 Hz, 2H), 5.69 (s, 2H, -CH_2_), 3.82 (s, 3H, -CH_3_). ^13^C NMR (75 MHz, CDCl_3_) *δ* 154.42, 153.76, 150.95, 141.69, 137.14, 135.84, 133.20, 132.40, 130.57, 130.27, 130.12, 129.36, 128.02, 126.01, 125.92, 125.35, 123.90, 120.96, 114.78, 111.99, 55.81, 38.17. HRMS (ESI) m/z calcd for C_24_H_20_N_5_O_2_^+^ (M + H)^+^ 409.1539, obtained 410.1617.

***1-*** ((***1-*** (***3-methoxyphenyl***) ***-1H-1,2,3-triazol-4-yl***) ***methyl***) ***-3-phenylquinoxalin-2*** (***1H***) ***-one*** (***5n***) Brown powder; yield 67.4%, m. p. = 177.4°C–181.0°C; ^1^H NMR (300 MHz, CDCl_3_) *δ* 8.35—8.27 (m, 2H), 8.15 (s, 1H), 8.02—7.92 (m, 2H), 7.65—7.58 (m, 1H), 7.53—7.47 (m, 3H), 7.39 (d, *J* = 7.1 Hz, 1H), 7.34 (d, *J* = 8.1 Hz, 1H), 7.28—7.26 (m, 1H), 7.23—7.19 (m, 1H), 6.93 (dd, *J* = 8.3, 2.4 Hz, 1H), 5.69 (s, 2H, -CH_2_), 3.84 (s, 3H, -CH_3_). ^13^C NMR (75 MHz, CDCl_3_) *δ* 160.48, 154.57, 153.81, 146.45, 143.08, 137.74, 135.79, 133.39, 133.30, 132.32, 130.68, 130.43, 129.43, 128.12, 124.08, 122.12, 114.86, 144.62, 112.33, 106.04, 55.56, 38.23. HRMS (ESI) m/z calcd for C_24_H_20_N_5_O_2_^+^ (M + H)^+^ 409.1539, obtained 410.1613.

***1-*** ((***1-*** (***4-methoxyphenyl***) ***-1H-1,2,3-triazol-4-yl***) ***methyl***) ***-3-phenylquinoxalin-2*** (***1H***) ***-one*** (***5o***) Brown powder; yield 70.0%, m. p. = 213.0°C–214.9°C; ^1^H NMR (300 MHz, CDCl_3_) *δ* 8.35—8.28 (m, 2H), 8.08 (s, 1H), 8.01 (d, *J* = 8.0 Hz, 1H), 7.95 (dd, *J* = 8.0, 1.4 Hz, 1H), 7.66—7.61 (m, 1H), 7.60—7.55 (m, 2H), 7.53—7.47 (m, 3H), 7.41—7.35 (m, 1H), 6.97 (d, *J* = 9.1 Hz, 2H), 5.68 (s, 2H, -CH_2_), 3.84 (s, 3H, -OCH_3_). ^13^C NMR (75 MHz, CDCl_3_) *δ* 159.81, 154.58, 153.81, 142.19, 135.82, 135.73, 135.31, 133.30, 132.35, 130.68, 130.40, 130.20, 129.43, 128.11, 124.05, 122.14, 122.03, 114.66, 55.53, 38.28. HRMS (ESI) m/z calcd for C_24_H_20_N_5_O_2_^+^ (M + H)^+^ 409.1539, obtained 410.1619.

***1-*** ((***1-Phenyl-1H-1,2,3-triazole***) ***-4-methyl***) ***-3-phenylquinoxaline-2*** (***1H***) ***-one*** (***5p***) Pale yellow powder; yield 77.4%, m. p. = 233.8°C–235.7°C; ^1^H NMR (300 MHz, CDCl_3_) *δ* 8.39—8.25 (m, 2H), 8.17 (s, 1H), 8.03—7.92 (m, 2H), 7.71—7.65 (m, 2H), 7.64—7.58 (m, 1H), 7.54—7.45 (m, 5H), 7.45—7.34 (m, 3H), 5.70 (s, 2H, -CH_2_). ^13^C NMR (75 MHz, CDCl_3_) *δ* 154.58, 153.83, 143.13, 136.74, 135.79, 133.30, 132.32, 130.69, 130.42, 130.40, 129.65, 129.42, 128.82, 128.11, 124.07, 122.05, 120.42, 114.60, 38.24. HRMS (ESI) m/z calcd for C_23_H_18_N_5_O^+^ (M + H)^+^ 379.1433, obtained 380.1513.

***1-*** ((***1-*** (***2-fluorobenzyl***) ***-1H-1,2,3-triazol-4-yl***) ***methyl***) ***-3-phenylquinoxalin-2*** (***1H***) ***-one*** (***6a***) Pale yellow powder; yield 70.6%, m. p. = 163.8°C–165.0°C; ^1^H NMR (300 MHz, CDCl_3_) *δ* 8.33—8.22 (m, 2H), 7.98—7.88 (m, 2H), 7.68 (s, 1H), 7.63—7.56 (m, 1H), 7.52—7.43 (m, 3H), 7.40—7.28 (m, 2H), 7.06—6.97 (m, 2H), 6.93 (d, *J* = 9.2 Hz, 1H), 5.58 (s, 2H, -CH_2_), 5.43 (s, 2H, -CH_2_). ^13^C NMR (75 MHz, CDCl_3_) *δ* 164.49, 154.48, 153.75, 142.95, 136.54, 136.44, 135.76, 133.24, 132.31, 130.76, 130.63, 130.36, 129.37, 128.09, 124.02, 123.84, 123.68, 123.64, 115.95, 115.68, 115.26, 114.97, 114.60, 53.53, 38.25. HRMS (ESI) m/z calcd for C_24_H_19_FN_5_O^+^ (M + H)^+^ 411.1495, obtained 412.1573.

***1-*** ((***1-*** (***3-fluorobenzyl***) ***-1H-1,2,3-triazol-4-yl***) ***methyl***) ***-3-phenylquinoxalin-2*** (***1H***) ***-one*** (***6b***) Pale yellow powder; yield 71.3%, m. p. = 154.9°C–157.3°C; ^1^H NMR (300 MHz, CDCl_3_) *δ* 8.34—8.20 (m, 2H), 7.99—7.89 (m, 2H), 7.71 (s, 1H), 7.62—7.55 (m, 1H), 7.52—7.45 (m, 3H), 7.39—7.28 (m, 2H), 7.22 (dd, *J* = 7.4, 1.6 Hz, 1H), 7.14—7.04 (m, 2H), 5.58 (s, 2H, -CH_2_), 5.51 (s, 2H, -CH_2_). ^13^C NMR (75 MHz, CDCl_3_) *δ* 162.08, 158.79, 154.45, 153.76, 142.75, 135.80, 133.22, 132.34, 130.97, 130.86, 130.60, 130.35, 130.31, 129.39, 128.08, 124.75, 124.71, 123.97, 123.86, 121.37, 115.97, 115.69, 114.66, 47.68, 38.23. HRMS (ESI) m/z calcd for C_24_H_19_FN_5_O^+^ (M + H)^+^ 411.1495, obtained 412.1571.

***1-*** ((***1-*** (***4-fluorobenzyl***) ***-1H-1,2,3-triazol-4-yl***) ***methyl***) ***-3-phenylquinoxalin-2*** (***1H***) ***-one*** (***6c***) Pale yellow powder; yield 71.8%, m. p. = 170.3°C–173.2°C; ^1^H NMR (300 MHz, CDCl_3_) *δ* 8.33—8.20 (m, 2H), 7.97—7.88 (m, 2H), 7.64 (s, 1H), 7.62—7.56 (m, 1H), 7.52—7.44 (m, 3H), 7.39—7.33 (m, 1H), 7.26—7.20 (m, 2H), 7.06—6.96 (m, 2H), 5.57 (s, 2H, -CH_2_), 5.40 (s, 2H, -CH_2_). ^13^C NMR (75 MHz, CDCl_3_) *δ* 164.44, 161.15, 154.48, 153.76, 143.66, 142.88, 135.76, 133.22, 132.31, 130.63, 130.39, 130.34, 130.14, 130.03, 129.36, 128.10, 124.01, 123.63, 116.21, 115.92, 114.62, 53.46, 38.25. HRMS (ESI) m/z calcd for C_24_H_19_FN_5_O^+^ (M + H)^+^ 411.1495, obtained 412.1573.

***1-*** ((***1-*** (***2-chlorobenzyl***) ***-1H-1,2,3-triazol-4-yl***) ***methyl***) ***-3-phenylquinoxalin-2*** (***1H***) ***-one*** (***6d***) Pale yellow powder; yield 76.8%, m. p. = 167.9°C–169.7°C; ^1^H NMR (300 MHz, CDCl_3_) *δ* 8.31—8.23 (m, 2H), 7.97—7.89 (m, 2H), 7.67 (s, 1H), 7.60 (ddd, *J* = 8.5, 7.4, 1.5 Hz, 1H), 7.51—7.45 (m, 3H), 7.40—7.28 (m, 3H), 7.23 (s, 1H), 7.11 (d, *J* = 6.9 Hz, 1H), 5.58 (s, 2H, -CH_2_), 5.41 (s, 2H, -CH_2_). ^13^C NMR (75 MHz, CDCl_3_) *δ* 154.42, 153.67, 142.91, 136.03, 135.70, 134.82, 133.18, 132.25, 130.56, 130.29, 129.32, 128.92, 128.14, 128.02, 126.13, 123.94, 123.77, 114.53, 53.41, 38.18. HRMS (ESI) m/z calcd for C_24_H_19_ClN_5_O^+^ (M + H)^+^ 427.1200, obtained 428.1278.

***1-*** ((***1-*** (***3-chlorobenzyl***) ***-1H-1,2,3-triazol-4-yl***) ***methyl***) ***-3-phenylquinoxalin-2*** (***1H***) ***-one*** (***6e***) Pale yellow powder; yield 68.7%, m. p. = 152.5°C–154.6°C; ^1^H NMR (300 MHz, CDCl_3_) *δ* 8.34—8.22 (m, 2H), 7.99—7.89 (m, 2H), 7.72 (s, 1H), 7.63—7.56 (m, 1H), 7.53—7.45 (m, 3H), 7.42—7.35 (m, 2H), 7.34—7.28 (m, 1H), 7.24—7.14 (m, 2H), 5.59 (s, 4H, -CH_2_, -CH_2_). ^13^C NMR (75 MHz, CDCl_3_) *δ* 154.39, 153.71, 135.76, 133.46, 133.18, 132.30, 131.93, 130.53, 130.28, 130.17, 129.83, 129.34, 128.01, 127.43, 123.91, 114.64,51.38, 38.20. HRMS (ESI) m/z calcd for C_24_H_19_ClN_5_O^+^ (M + H)^+^ 427.1200, obtained 428.1275.

***1-*** ((***1-*** (***4-chlorobenzyl***) ***-1H-1,2,3-triazol-4-yl***) ***methyl***) ***-3-phenylquinoxalin-2*** (***1H***) ***-one*** (***6f***) Pale yellow powder; yield 72.4%, m. p. = 188.6°C–190.3°C; ^1^H NMR (300 MHz, CDCl_3_) *δ* 8.33—8.21 (m, 2H), 7.98—7.87 (m, 2H), 7.65—7.56 (m, 2H), 7.53—7.45 (m, 3H), 7.40—7.33 (m, 1H), 7.26—7.20 (m, 2H), 7.06—6.97 (m, 2H), 5.57 (s, 2H, -CH_2_), 5.41 (s, 2H, -CH_2_). ^13^C NMR (75 MHz, CDCl_3_) *δ* 153.73, 142.84, 135.71, 133.18, 132.26, 130.57, 130.30, 130.06, 129.31, 128.04, 123.95, 123.55, 123.51, 123.32, 116.17, 115.88, 114.56, 90.26, 53.42, 38.21. HRMS (ESI) m/z calcd for C^24^H^19^ClN^5^O^+^ (M + H)^+^ 427.1200, obtained 428.1281.

***1-*** ((***1-*** (***3-bromobenzyl***) ***-1H-1,2,3-triazol-4-yl***) ***methyl***) ***-3-phenylquinoxalin-2*** (***1H***) ***-one*** (***6g***) Pale yellow powder; yield 72.4%, m. p. = 166.4°C–168.6°C; ^1^H NMR (300 MHz, CDCl_3_) *δ* 8.35—8.21 (m, 2H), 7.99—7.88 (m, 2H), 7.68 (s, 1H), 7.62—7.55 (m, 1H), 7.53—7.44 (m, 3H), 7.40—7.26 (m, 3H), 7.23 (s, 1H), 7.11 (d, *J* = 7.0 Hz, 1H), 5.58 (s, 2H, -CH_2_), 5.41 (s, 2H, -CH_2_). ^13^C NMR (75 MHz, CDCl_3_) *δ* 154.44, 153.70, 142.92, 136.02, 135.71, 134.84, 133.19, 132.26, 130.59, 130.31, 129.33, 128.95, 128.17, 128.05, 126.16, 123.97, 123.80, 114.55, 53.45, 38.20. HRMS (ESI) m/z calcd for C_24_H_19_BrN_5_O^+^ (M + H)^+^ 471.0694, obtained 472.0769.

***1-*** ((***1-*** (***4-bromobenzyl***) ***-1H-1,2,3-triazol-4-yl***) ***methyl***) ***-3-phenylquinoxalin-2*** (***1H***) ***-one*** (***6h***) Pale yellow powder; yield 75.3%, m. p. = 196.4°C–198.2°C; ^1^H NMR (300 MHz, CDCl_3_) *δ* 8.32—8.22 (m, 2H), 7.98—7.89 (m, 2H), 7.67—7.56 (m, 2H), 7.51—7.43 (m, 5H), 7.40—7.34 (m, 1H), 7.11 (d, *J* = 8.4 Hz, 2H), 5.58 (s, 2H, -CH_2_), 5.39 (s, 2H, -CH_2_). ^13^C NMR (75 MHz, CDCl_3_) *δ* 154.45, 153.73, 142.91, 135.71, 133.14, 133.19, 133.08, 132.26, 13219, 130.60, 130.35, 129.74, 129.33, 128.07, 123.99, 123.67, 122.94, 114.56, 53.50, 38.22. HRMS (ESI) m/z calcd for C_24_H_19_BrN_5_O^+^ (M + H)^+^ 471.0694, obtained 472.0771.

***1-*** ((***1-*** (***2-methylbenzyl***) ***-1H-1,2,3-triazol-4-yl***) ***methyl***) ***-3-phenylquinoxalin-2*** (***1H***) ***-one*** (***6i***) Pale yellow powder; yield 70.3%, m. p. = 167.3°C–169.8°C; ^1^H NMR (300 MHz, CDCl_3_) *δ* 8.33—8.20 (m, 2H), 7.99—7.89 (m, 2H), 7.62—7.56 (m, 1H), 7.53 (s, 1H), 7.51—7.45 (m, 3H), 7.39—7.33 (m, 1H), 7.26—7.21 (m, 1H), 7.21—7.14 (m, 2H), 7.14—7.09 (m, 1H), 5.57 (s, 2H, -CH_2_), 5.46 (s, 2H, -CH_2_), 2.25 (s, 3H, -CH_3_). ^13^C NMR (75 MHz, CDCl_3_) *δ* 154.45, 153.76, 142.60, 136.82, 135.83, 133.25, 132.37, 132.11, 130.99, 130.99, 130.61, 130.33, 129.66, 129.52, 129.40, 129.15, 128.09, 126.63, 123.97, 123.49, 114.73, 52.37, 38.28, 18.98. HRMS (ESI) m/z calcd for C_25_H_22_N_5_O^+^ (M + H)^+^ 407.1746, obtained 408.1238.

***1-*** ((***1-*** (***3-methylbenzyl***) ***-1H-1,2,3-triazol-4-yl***) ***methyl***) ***-3-phenylquinoxalin-2*** (***1H***) ***-one*** (***6j***) Pale yellow powder; yield 68.6%, m. p. = 133.9°C–135.5°C; ^1^H NMR (300 MHz, CDCl_3_) *δ* 8.34—8.22 (m, 2H), 7.93 (dd, *J* = 13.6, 5.2 Hz, 2H), 7.63 (s, 1H), 7.59 (t, *J* = 7.2 Hz, 1H), 7.52—7.44 (m, 3H), 7.36 (t, *J* = 7.7 Hz, 1H), 7.25—7.19 (m, 1H), 7.13 (d, *J* = 7.8 Hz, 1H), 7.04 (d, *J* = 6.8 Hz, 2H), 5.58 (s, 2H, -CH_2_), 5.40 (s, 2H, -CH_2_), 2.30 (s, 3H, -CH_3_). ^13^C NMR (75 MHz, CDCl_3_) *δ* 154.49, 153.80, 142.75, 138.90, 135.83, 134.05, 133.25, 132.37, 130.63, 130.36, 129.67, 12955, 129.40, 129.06, 128.94, 128.10, 125.28, 123.99, 123.66, 114.71, 54.28, 38.32, 21.27. HRMS (ESI) m/z calcd for C_25_H_22_N_5_O^+^ (M + H)^+^ 407.1746, obtained 408.1825.

***1-*** ((***1-*** (***4-methylbenzyl***) ***-1H-1,2,3-triazol-4-yl***) ***methyl***) ***-3-phenylquinoxalin-2*** (***1H***) ***-one*** (***6k***) Pale yellow powder; yield 68.3%, m. p. = 196.1°C–198.1°C; ^1^H NMR (300 MHz, CDCl_3_) *δ* 8.33—8.21 (m, 2H), 7.93 (t, *J* = 8.8 Hz, 2H), 7.64—7.53 (m, 2H), 7.51—7.44 (m, 3H), 7.35 (t, *J* = 7.5 Hz, 1H), 7.13 (s, 4H), 5.55 (s, 2H, -CH_2_), 5.38 (s, 2H, -CH_2_), 2.31 (s, 3H, -CH_3_). ^13^C NMR (75 MHz, CDCl_3_) *δ* 154.48, 153.79, 142.70, 138.73, 135.84, 133.25, 132.38, 131.11, 130.63, 130.35, 130.33, 129.74, 129.40, 128.25, 128.10, 123.98, 123.56, 114.73, 54.08, 38.31, 21.12. HRMS(ESI) m/z calcd for C_25_H_22_N_5_O^+^ (M + H)^+^ 407.1746, obtained 408.1820.

***1-*** ((***1-*** (***3-methoxybenzyl***) ***-1H-1,2,3-triazol-4-yl***) ***methyl***) ***-3-phenylquinoxalin-2*** (***1H***) ***-one*** (***6l***) Brown powder; yield 67.3%, m. p. = 139.4°C–141.8°C; ^1^H NMR (300 MHz, CDCl_3_) *δ* 8.33—8.22 (m, 2H), 7.94 (t, *J* = 8.2 Hz, 2H), 7.65 (s, 1H), 7.59 (t, *J* = 7.7 Hz, 1H), 7.52—7.45 (m, 3H), 7.36 (t, *J* = 7.5 Hz, 1H), 7.23 (d, *J* = 7.8 Hz, 1H), 6.88—6.79 (m, 2H), 6.74 (s, 1H), 5.58 (s, 2H, -CH_2_), 5.41 (s, 2H, -CH_2_), 3.74 (s, 3H, -OCH_3_). ^13^C NMR (75 MHz, CDCl_3_) *δ* 159.99, 154.48, 153.78, 142.79, 135.82, 135.58, 133.26, 132.37, 130.62, 132.37, 130.62, 130.35, 130.14, 129.74, 129.41, 128.09, 123.98, 123.73, 120.34, 114.68, 114.24, 113.74, 55.20, 54.19, 38.29. HRMS (ESI) m/z calcd for C_25_H_22_N_5_O_2_^+^ (M + H)^+^ 423.1695, obtained 424.1773.

***1-*** ((***1-*** (***4-methoxybenzyl***) ***-1H-1,2,3-triazol-4-yl***) ***methyl***) ***-3-phenylquinoxalin-2*** (***1H***) ***-one*** (***6m***) Brown powder; yield 67.3%, m. p. = 139.4°C–141.8°C; ^1^H NMR (300 MHz, CDCl_3_) *δ* 8.32—8.23 (m, 2H), 7.98—7.89 (m, 2H), 7.62—7.55 (m, 2H), 7.52—7.45 (m, 3H), 7.36 (t, *J* = 7.6 Hz, 1H), 7.19 (d, *J* = 8.6 Hz, 2H), 6.85 (d, *J* = 8.6 Hz, 2H), 5.57 (s, 2H, -CH_2_), 5.37 (s, 2H, -CH_2_), 3.78 (s, 3H, -OCH_3_). ^13^C NMR (75 MHz, CDCl_3_) *δ* 159.89, 154.48, 153.77, 142.68, 135.84, 133.24, 132.37, 130.62, 130.35, 130.32, 129.77, 129.40, 128.09, 126.13, 123.97, 123.43, 114.72, 114.43, 55.24, 53.80, 38.31. HRMS (ESI) m/z calcd for C_25_H_22_N_5_O_2_^+^ (M + H)^+^ 423.1695, obtained 424.1770.

***1-*** ((***1-benzyl-1H-1,2,3-triazol-4-yl***) ***methyl***) ***-3-phenylquinoxalin-2*** (***1H***) ***-one*** (***6n***) Pale yellow powder; yield 75.5%, m. p. = 183.7°C–184.8°C; ^1^H NMR (300 MHz, CDCl_3_) *δ* 8.34—8.21 (m, 2H), 7.92 (t, *J* = 7.7 Hz, 2H), 7.65 (s, 1H), 7.56 (t, *J* = 7.8 Hz, 1H), 7.51—7.43 (m, 3H), 7.38—7.28 (m, 4H), 7.25—7.18 (m, 2H), 5.55 (s, 2H, -CH_2_), 5.42 (s, 2H, -CH_2_). ^13^C NMR (75 MHz, CDCl_3_) *δ* 154.47, 153.76, 142.78, 135.81, 134.16, 133.24, 132.35, 130.61, 130.33, 129.39, 129.05, 128.76, 128.16, 128.08, 123.97, 123.70, 114.68, 114.43, 55.24, 38.29. HRMS (ESI) m/z calcd for C_24_H_20_N_5_O^+^ (M + H)^+^ 393.1589, obtained 494.1664.

### 4.3 Cell culturing

LO2 and HK-2 cells were cultured at 37°C in RPMI1640 (Hyclone, United States) and DMEM low-sugar medium (Hyclone) supplemented with 10% fetal bovine serum (Gibco™, United States) together with 1% penicillin/streptomycin (Beyotime™, China), respectively, in a 5% CO_2_ incubator.

### 4.4 Cell viability assay

Cytotoxic effects of the synthesized compounds were assessed using the MTT assay. LO2 cell cultures were added to 96-well plates (1 × 10^4^ cells/well in 100 µl/well) and treated for 48 h with the 30 synthesized compounds dissolved in DMSO (10, 20, 40, and 80 μg/ml) for 1 day. Subsequently, 10 µl of the MTT reagent was added to each well, followed by incubation for 4 h at 37°C. Later, 150 μl of dimethylsulfoxide was added to dissolve the precipitate. Absorbance at 570 nm was determined using a microplate reader (Thermo Scientific, MA, United States).

Cell viability (%) = optical density (OD) of the measurement group/OD of the normal group (%)

### 4.5 Cell molding and drug handling

LO2 and HK-2 cells were inoculated in 6-well plates (1 × 10^5^ cells/well in 100 µL/well). After the LO2 and HK-2 cells were plastered, they were treated with RPMI1640 and DMEM low-sugar medium, respectively, for 6 h to homogenize them and categorized into the following six groups: normal group, high-sugar group, low-dose administration group, medium-dose administration group, high-dose administration group, and positive control group. Except for the normal group, which was incubated in 3.0 mM of the low-sugar medium, each group was induced with 45 mM of high-sugar medium for 48 h. The drug administration group was induced with a high-sugar medium and then administered with 40 μM of the target compounds (5a–p and 6a–n) for 48 h. In the quantitative efficacy study of compounds 5i and 6b dissolved in DMSO, 10, 20, and 40 μM of the compounds were administered. In the positive control group, 40 μM of Pioglitazone and the lead compound dissolved in DMSO were administered for 48 h. Subsequently, the samples were stored at −80°C.

### 4.6 Glucose content testing

After treating the LO2 cells with the appropriate concentrations of the target compounds (5a–p and 6a–n) for 48 h as described in Section 4.5, the medium was removed and the residual medium was washed three times with phosphate-buffered saline (PBS), followed by incubation for 16 h in 1 ml of glucose-stimulating medium (phenol red-free RPMI1640 + 20 mmol/L sodium lactate and + 2 mmol/L sodium pyruvate). For the last 3 h, 1 nmol/L of insulin was added to each group, except the normal group. The supernatant of LO2 cells was then aspirated. The glucose content in the supernatant was determined using the glucose oxidase kit according to the manufacturer’s instructions. The glucose content was calculated from the obtained absorbance (OD) values.

Glucose concentration (mmol/L) = standard concentration × (sample tube OD—Blank tube OD)/(standard tube OD—Blank tube OD) OD—Blank tube OD.

### 4.7 Measurement of intracellular ROS content

LO2 cells were treated with compounds 5i and 6b (10, 20, 40 μM) dissolved in DMSO for 48 h as described in Section 4.5, and the ROS content was measured using the DCFH-DA fluorescent probe assay. As per the ROS kit instructions, the treated cells were aspirated from the original medium, added to serum-free RPMI, and incubated for 20 min with 10 μM of DCFH-DA working solution. Subsequently, the samples were washed three times with PBS, and the fluorescence intensity was measured using a fluorescence microscope (Olympus, JAPAN; 488 nm excitation wavelength and 525 nm emission wavelength).

### 4.8 Determination of intracellular MDA, SOD, and CAT contents

LO2 cells were treated with compounds 5i and 6b (10, 20, 40 μM) dissolved in DMSO for 48 h as described in Section 4.5, and the supernatant was discarded. MDA, SOD, and CAT contents were determined using the appropriate ELISA kits, and the absorbance values were used to calculate MDA, SOD, and CAT contents according to the kit instructions.

### 4.9 Molecular docking analysis

Molecular docking is one of the most important methods of molecular modeling, and it is used to effectively screen drug targets. The 3D crystal structure of the protein of interest was obtained from the Rcsb protein database (http://www.rcsb.org/pdb), such as SIRT3 (PDB: 3g1s), HIF1a (PDB: 1ka8), GLP1 (PDB: 3iol), PINK1 (PDB: 7t3x), TXNIP (PDB: 4gfx), DPP4 (PDB: 1j2e), GLUT1 (PDB: 4pyp), SGLT2(PDB: 2xp2), GLUT4 (PDB: 2xq2), SIRT1 (PDB: 5btr), NRF2 (PDB: 3zgc), and NIF1(PDB: 2hhl) (Yeo et al., 2002; Hiramatsu et al., 2003; Almo et al., 2007; Almo et al., 2007; Jin et al., 2009; Underwood et al., 2010; Deng et al., 2014; Liu et al., 2016; Stalin et al., 2020; Gan et al., 2022; Kp et al., 2022; Najafi et al., 2022). ChemDraw 15.0 was used to obtain the 3D structures of compounds 5i and 6b. First, the compounds 5i and 6b were examined for charge and protonation states using the visualization software AutoDock Tools-1.5.6 and then the protein molecules were processed to remove water molecules, add hydrogen atoms, and remove unwanted heteroatoms, all of which were saved as pdbpt files. Next, the grid size, center coordinates, and the number of grid points and grid distance were docked by Grid Box in the software with reference to the literature. AutoDock software was used to analyze the bonding and binding energies in complex protein-ligand conformational interactions, which were ultimately viewed using the AutoDock Vina plug-in.

### 4.10 2-NBDG uptake

HK-2 cells were seeded in a confocal laser dish for 24 h. These were then treated with compound 5i (10, 20, 40 μM), compound 6b (10, 20, 40 μM), Pioglitazone (40 μM) and the lead compound (40 μM) in serum-free medium for 6 h. After this, 2-NBDG (50 μM) was added to cells for 2 h, the cells washed in HBSS buffer three times, and then observed by laser confocal microscopy.

### 4.11 Western blotting

LO2 and HK-2 cells were treated with the compounds 6b dissolved in DMSO for 48 h as described in Section 4.5. Consequently, the cultures were lysed in a lysis buffer supplemented with phosphatase inhibitors (1:100; 20 min: 4°C), followed by centrifugation (10 min; 13,000 x*g*; 4°C). The BCA kit was used to measure the protein levels in the collected supernatants, followed by separation of the proteins via 8%–10% SDS-PAGE and transported onto 0.22-µM PVDF membranes (GE Healthcare, UΚ). The blots were consequently probed with bespoke antibodies against PPAR-γ, SGLT2, GLUT4 and GLUT1 (CST, United States) (overnight/4°C). The blotting was exposed to triple-wash with TBST (TBS + 0.1% Tween 20; Beyotime™), followed by incubation of the cultures (60 min/ambient temperature) with the relevant HRP-conjugated 2° antibodies.

### 4.12 Statistical analysis

Statistical analysis was performed using SPSS 26.0 software, and GraphPad Prism 8 software was used to process the statistical graphs. The results were presented as the mean ± standard error (SE). One-way analysis of variance (ANOVA) was applied to determine the statistical significance between the control and test groups. *p* < 0.05 was considered to indicate a statistically significant difference.

## Data Availability

The original contributions presented in the study are included in the article/Supplementary Material, further inquiries can be directed to the corresponding authors.

## References

[B1] Almo S. C. Bonanno J. B. Sauder J. M. Emtage S. Dilorenzo T. P. Malashkevich V. (2007). Structural genomics of protein phosphatases. J. Struct. Funct. Genomics. 8 (2-3), 121–140. 10.1007/s10969-007-9036-1 18058037 PMC4163028

[B2] Bistrović A. Krstulović L. Harej A. Grbčić P. Sedić M. Koštrun S. (2018). Design, synthesis and biological evaluation of novel benzimidazole amidines as potent multi-target inhibitors for the treatment of non-small cell lung cancer. Eur. J. Med. Chem. 143, 1616–1634. 10.1016/j.ejmech.2017.10.061 29133046

[B3] Bozorov K. Zhao J. Aisa H. A. (2019). 1,2,3-Triazole-containing hybrids as leads in medicinal chemistry: A recent overview. Bioorg. Med. Chem. 27 (16), 3511–3531. 10.1016/j.bmc.2019.07.005 31300317 PMC7185471

[B4] Deng D. Xu C. Sun P. Wu J. Yan C. Hu M. (2014). Crystal structure of the human glucose transporter GLUT1. Nature 510 (7503), 121–125. 10.1038/nature13306 24847886

[B5] Forbes J. M. Cooper M. E. (2013). Mechanisms of diabetic complications. Physiol. Rev. 93 (1), 137–188. 10.1152/physrev.00045.2011 23303908

[B6] Fuh M. T. Tseng C. C. Li S. M. Tsai S. E. Chuang T. J. Lu C. H. (2021). Design, synthesis and biological evaluation of glycolamide, glycinamide, and β-amino carbonyl 1,2,4-triazole derivatives as DPP-4 inhibitors. Bioorg. Chem. 114, 105049. 10.1016/j.bioorg.2021.105049 34147879

[B7] Gan Z. Y. Callegari S. Cobbold S. A. Cotton T. R. Mlodzianoski M. J. Schubert A. F. (2022). Activation mechanism of PINK1. Nature 602 (7896), 328–335. 10.1038/s41586-021-04340-2 34933320 PMC8828467

[B8] Gao F. Wang T. Xiao J. Huang G. (2019). Antibacterial activity study of 1,2,4-triazole derivatives. Eur. J. Med. Chem. 173, 274–281. 10.1016/j.ejmech.2019.04.043 31009913

[B9] Greenhill C. (2021). T2DM remission - consensus on definition. Nat. Rev. Endocrinol. 17 (11), 639. 10.1038/s41574-021-00565-3 34508251

[B10] Gross B. Pawlak M. Lefebvre P. Staels B. (2017). PPARs in obesity-induced T2DM, dyslipidaemia and NAFLD. Nat. Rev. Endocrinol. 13 (1), 36–49. 10.1038/nrendo.2016.135 27636730

[B11] Heppner K. M. Perez-Tilve D. (2015). GLP-1 based therapeutics: Simultaneously combating T2DM and obesity. Front. Neurosci. 9, 92. 10.3389/fnins.2015.00092 25852463 PMC4367528

[B12] Hiramatsu H. Kyono K. Higashiyama Y. Fukushima C. Shima H. Sugiyama S. (2003). The structure and function of human dipeptidyl peptidase IV, possessing a unique eight-bladed beta-propeller fold. Biochem. Biophys. Res. Commun. 302 (4), 849–854. 10.1016/s0006-291x(03)00258-4 12646248

[B13] Indalkar K. S. Khatri C. K. Chaturbhuj G. U. (2017). Rapid, efficient and eco-friendly procedure for the synthesis of quinoxalines under solvent-free conditions using sulfated polyborate as a recyclable catalyst. J. Chem. Sci. 129 (2), 141–148. 10.1007/s12039-017-1235-0

[B14] Jin L. Wei W. Jiang Y. Peng H. Cai J. Mao C. (2009). Crystal structures of human SIRT3 displaying substrate-induced conformational changes. J. Biol. Chem. 284 (36), 24394–24405. 10.1074/jbc.M109.014928 19535340 PMC2782032

[B15] Kamboj V. K. VermaDhanda P. K. A. Ranjan S. (2015). 1,2,4-triazole derivatives as potential scaffold for anticonvulsant activity. Cent. Nerv. Syst. Agents Med. Chem. 15 (1), 17–22. 10.2174/1871524915666150209100533 25675400

[B16] Kojima K. Yakushiji F. Katsuyama A. Ichikawa S. (2020). Total synthesis of echinomycin and its analogues. Org. Lett. 22 (11), 4217–4221. 10.1021/acs.orglett.0c01268 32379459

[B17] Kp A. D. Shimoga Janakirama A. R. Martin A. (2022). SIRT1 activation by taurine: *In vitro* evaluation, molecular docking and molecular dynamics simulation studies. J. Nutr. Biochem. 102, 108948. 10.1016/j.jnutbio.2022.108948 35051560

[B18] Liu Y. Lau J. Li W. Tempel W. Li L. Dong A. (2016). Structural basis for the regulatory role of the PPxY motifs in the thioredoxin-interacting protein TXNIP. Biochem. J. 473 (2), 179–187. 10.1042/BJ20150830 26527736

[B19] Najafi F. Kavoosi G. Siahbalaei R. Kariminia A. (2022). Anti-oxidative and anti-hyperglycemic properties of agastache foeniculum essential oil and oily fraction in hyperglycemia-stimulated and lipopolysaccharide-stimulated macrophage cells: *In vitro* and *in silico* studies. J. Ethnopharmacol. 284, 114814. 10.1016/j.jep.2021.114814 34775034

[B20] Patidar A. K. Jeyakandan M. Mobiya A. K. Selvam G. (2011). ChemInform abstract: Exploring potential of quinoxaline moiety. Cheminform 42 (28), 386–392. 10.1002/chin.201128256

[B21] Sagar S. R. Singh D. P. Das R. D. Panchal N. B. Sudarsanam V. Nivsarkar M. (2019). Pharmacological investigation of quinoxaline-bisthiazoles as multitarget-directed ligands for the treatment of Alzheimer's disease. Bioorg. Chem. 89, 102992. 10.1016/j.bioorg.2019.102992 31174042

[B22] Stalin A. Kandhasamy S. Kannan B. S. Verma R. S. Ignacimuthu S. Kim Y. (2020). Synthesis of a 1,2,3-bistriazole derivative of embelin and evaluation of its effect on high-fat diet fed-streptozotocin-induced type 2 diabetes in rats and molecular docking studies. Bioorg. Chem. 96, 103579. 10.1016/j.bioorg.2020.103579 31978685

[B23] Tariq S. Somakala K. Amir M. (2018). Quinoxaline: An insight into the recent pharmacological advances. Eur. J. Med. Chem. 143, 542–557. 10.1016/j.ejmech.2017.11.064 29207337

[B24] Underwood C. R. Garibay P. Knudsen L. B. Hastrup S. Peters G. H. Rudolph R. (2010). Crystal structure of glucagon-like peptide-1 in complex with the extracellular domain of the glucagon-like peptide-1 receptor. J. Biol. Chem. 285 (1), 723–730. 10.1074/jbc.M109.033829 19861722 PMC2804221

[B25] Xia Y. Qu F. Peng L. (2010). Triazole nucleoside derivatives bearing aryl functionalities on the nucleobases show antiviral and anticancer activity. Mini. Rev. Med. Chem. 10 (9), 806–821. 10.2174/138955710791608316 20482498

[B26] Xu L. Li Y. Dai Y. Peng J. (2018). Natural products for the treatment of type 2 diabetes mellitus: Pharmacology and mechanisms. Pharmacol. Res. 130, 451–465. 10.1016/j.phrs.2018.01.015 29395440

[B27] Yang L. Liu D. Yan H. Chen K. (2022). Dapagliflozin attenuates cholesterol overloading-induced injury in mice hepatocytes with type 2 diabetes mellitus (T2DM) via eliminating oxidative damages. Cell. Cycle 21 (6), 641–654. 10.1080/15384101.2022.2031429 35100086 PMC8942414

[B28] Yeo H. J. Ziegelin G. Korolev S. Calendar R. Lanka E. Waksman G. (2002). Phage P4 origin-binding domain structure reveals a mechanism for regulation of DNA-binding activity by homo- and heterodimerization of winged helix proteins. Mol. Microbiol. 43 (4), 855–867. 10.1046/j.1365-2958.2002.02796.x 11929537

[B29] Zimmet P. Shi Z. El-Osta A. Ji L. (2018). Epidemic T2DM, early development and epigenetics: Implications of the Chinese famine. Nat. Rev. Endocrinol. 14 (12), 738–746. 10.1038/s41574-018-0106-1 30310152

