# Supplementary material for: Synthesis and hypoglycemic activity of quinoxaline derivatives
Source: Front Chem. 2023 Jul 6;11:1197124. doi: 10.3389/fchem.2023.1197124 (PMC10358274; doi:10.3389/fchem.2023.1197124)
Supplement: Supplementary file 1 [file DataSheet1.PDF]

Supporting Information for

Synthesis and Hypoglycemic Activity of Quinoxaline Derivatives

Weidong Jia[a]+, Jingjing Wang[b]+,Chengxi Wei[c], Ming Bian*[d] ,Shuyin Bao*[e] and Lijun Yu*[f]

[a] Weidong Jia; Medical College, Inner Mongolia Minzu University, Tongliao, 028000, Inner Mongolia Autonomous Region, PR China.

[b] Jingjing Wang; Medical College, Inner Mongolia Minzu University, Tongliao, 028000, Inner Mongolia Autonomous Region, PR China.

[c] Chengxi Wei; Medical College, Inner Mongolia Minzu University, Tongliao, 028000, Inner Mongolia Autonomous Region, PR China.

[d] Ming Bian; Medical College, Inner Mongolia Minzu University, Tongliao, 028000, Inner Mongolia Autonomous Region, PR China. E-mail: bmz3@163.com

[e] Shuyin Bao; Medical College, Inner Mongolia Minzu University, Tongliao, 028000, Inner Mongolia Autonomous Region, PR China. E-mail: baoshuyin8881@163.com

[f] Lijun Yu; Medical College, Inner Mongolia Minzu University, Tongliao, 028000, Inner Mongolia Autonomous Region, PR China. E-mail: tl_ylj@163.com

Content

The characterization of target compounds..3-21

The spectrum of IR, ^{1}H NMR, ^{13}C NMR , HRMS of compounds and FT-IR spectrum of compounds 5a-5p...24-55

The spectrum of IR, ^{1}H NMR, ^{13}C NMR , HRMS of compounds and FT-IR spectrum of compounds 6a-6n...56-84

The absorbance of compounds 5a-5p and 6a-6n..85

The characterization of target compounds

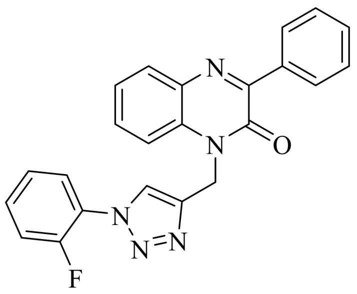

1-((1-(2-fluorophenyl)-1H-1,2,3-triazol-4-yl)methyl)-3-phenylquinoxalin-2(1H)-one (5a)

Pale yellow powder; Yield 69.4%, m. p. = 158.7-160.2 °C; IR (KBr, cm^{-1}): 3740.71, 3608.12, 3128.98, 3083.67, 2923.12, 2359.18, 2319.26, 1650.66, 1519.25, 1459.57, 1379.73, 1296.92, 1240,46, 1118.06, 1057.55, 758.78, 687.74. ^{1}H NMR (300 MHz, $CDCl_3$) δ 8.37 – 8.28 (m, 2H), 8.26 (d, J = 2.7 Hz, 1H), 8.03 – 7.93 (m, 2H), 7.90 – 7.83 (m, 1H), 7.67 – 7.60 (m, 1H), 7.53 – 7.46 (m, 3H), 7.43 – 7.36 (m, 2H), 7.32 – 7.28 (m, 1H), 7.23 (d, J = 1.3 Hz, 1H), 5.71 (s, 2H, -CH_2). ^{13}C NMR (75 MHz, $CDCl_3$) δ 154.92, 154.43, 153.78, 151.59, 142.73, 135.73, 133.23, 132.30, 130.59, 130.38, 130.32, 130.23, 129.37, 128.03, 125.16, 125.07, 125.03, 124.78, 123.97, 117.05, 116.78, 114.53, 38.04. HRMS (ESI) m/z calcd for $C_{23}H_{17}FN_5O^+$ $(M+H)^+$ 397.1339, found 398.1413.

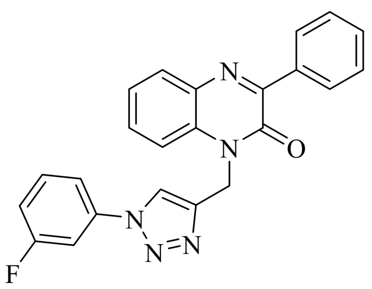

1-((1-(3-fluorophenyl)-1H-1,2,3-triazol-4-yl)methyl)-3-phenylquinoxalin-2(1H)-one (5b)

Pale yellow powder; Yield 72.8%, m. p. = 179.4-182.5 °C; IR (KBr, cm^{-1}): 3850.94, 3743.83, 3600.06, 3129.57, 3068.04, 2367.35, 2318.82, 1650.66, 1519.30, 1459.53, 1379.72, 1296.91, 1240.45, 1117.81, 1053.65, 936.73, 814.62, 758.68, 686.88, 532.65. ^{1}H NMR (300 MHz, $CDCl_3$) δ 8.41 – 8.22 (m, 2H), 8.18 (s, 1H), 7.96 (t, J = 8.0 Hz, 2H), 7.66 – 7.58 (m, 1H), 7.56 – 7.30 (m, 7H), 7.11 (s, 1H), 5.68 (s, 2H, $-CH_2$). ^{13}C NMR (75 MHz, $CDCl_3$) δ 164.52, 161.23, 154.49, 153.71, 143.31, 135.67, 133.23, 132.19, 131.09, 130.98, 130.63, 130.37, 129.34, 128.05, 124.05, 122.00, 115.82, 115.63, 115.59, 115.54, 114.43, 108.31, 107.96, 38.08. HRMS (ESI) m/z calcd for $C_{23}H_{17}FN_5O^+$ $(M+H)^+$ 397.1339, found 398.1414.

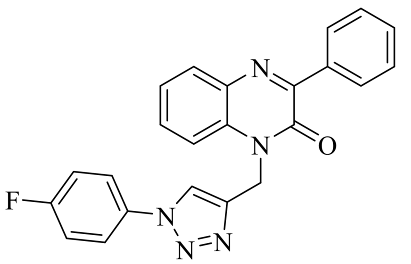

1-((1-(4-fluorophenyl)-1H-1,2,3-triazol-4-yl)methyl)-3-phenylquinoxalin-2(1H)-one (5c)

Pale yellow powder; Yield 73.4%, m. p. = 194.6-195.9 °C; IR (KBr, cm^{-1}): 3854.80, 3739.01, 3604.37, 3132.02, 3074.55, 2361.22, 2319.25, 1697.96, 1650.66, 1519.26, 1459.58, 1379.78, 1296.95, 1240.49, 1118.12, 1057.66, 932.65, 822.45, 758.72, 687.72.^{1}H NMR (300 MHz, $CDCl_3$) δ 8.36 – 8.26 (m, 2H), 8.13 (s, 1H), 8.02 – 7.92 (m, 2H), 7.70 – 7.59 (m, 3H), 7.54 – 7.46 (m, 3H), 7.42 – 7.35 (m, 1H), 7.21 – 7.13 (m, 2H, $-CH_2$), 5.69 (s, 2H, $-CH_3$). ^{13}C NMR (75 MHz, $CDCl_3$) δ 163.99, 160.68, 154.52, 153.73, 143.20, 135.69, 133.24, 132.94, 132.22, 130.62, 130.38, 129.34,

128.05, 124.03, 122.41, 122.29, 122.19, 122.17, 116.72, 116.42, 114.47, 38.13. HRMS (ESI) m/z calcd for $C_{23}H_{17}FN_5O^+$ $(M+H)^+$ 397.1339, found 498.1420.

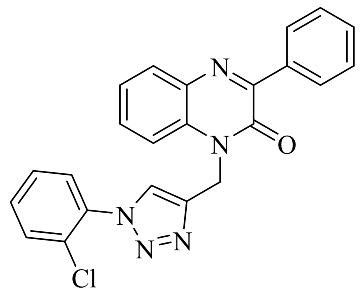

1-((1-(2-chlorophenyl)-1H-1,2,3-triazol-4-yl)methyl)-3-phenylquinoxalin-2(1H)-one (5d)

Pale yellow powder; Yield 76.6%, m. p. = 167.9-169.7 °C; IR (KBr, cm^{-1}): 3854.64, 3741.34, 3611.15, 3133.79, 3085.71, 2357.17, 1751.35, 1696.24, 1648.50, 1592.73, 1506.48, 1454.82, 1294.27, 1236.78, 1038.67, 760.85, 685.27.^{1}H NMR (300 MHz, $CDCl_3$) δ 8.30 (dd, J = 6.7, 3.0 Hz, 2H), 8.18 (s, 1H), 8.05 – 7.92 (m, 2H), 7.68 – 7.61 (m, 1H), 7.58 – 7.45 (m, 5H), 7.46 – 7.35 (m, 3H), 5.71 (s, 2H, -CH_2). ^{13}C NMR (75 MHz, $CDCl_3$) δ 154.43, 153.74, 142.11, 135.79, 135.72, 134.55, 133.22, 132.32, 130.75, 130.63, 130.37, 130.32, 129.36, 128.50, 128.03, 127.73, 127.59, 125.98, 123.99, 114.57, 38.10. HRMS (ESI) m/z calcd for $C_{23}H_{17}ClN_5O^+$ $(M+H)^+$ 413.1043, found 414.1119.

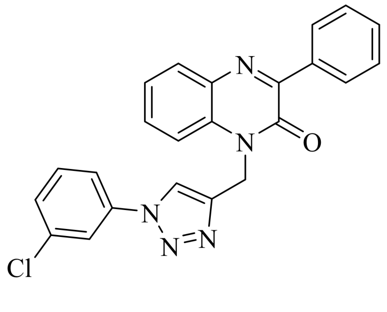

1-((1-(3-chlorophenyl)-1H-1,2,3-triazol-4-yl)methyl)-3-phenylquinoxalin-2(1H)-one (5e)

Pale yellow powder; Yield 75.6%, m. p. = 187.1-189.1 °C; IR (KB, cm^{-1}): 3854.33, 3742.95, 3609.27, 3151.02, 3079.59, 2308.72, 1750.96, 1695.95, 1647.43, 1593.80, 1507.46, 1453.06, 1301.51, 1239.48, 1045.12, 768.57, 688.35. ^1H NMR (300 MHz, $CDCl_3$) δ 8.39 – 8.23 (m, 2H), 8.18 (s, 1H), 8.03 – 7.91 (m, 2H), 7.74 (s, 1H), 7.67 – 7.48 (m, 5H), 7.41 (q, J = 8.3 Hz, 3H), 5.69 (s, 2H, $-CH_2$). ^{13}C NMR (75 MHz, $CDCl_3$) δ 169.13, 154.50, 153.71, 143.33, 137.58, 137.45, 135.65, 135.42, 133.23, 132.16, 130.66, 130.39, 129.34, 128.82, 128.07, 124.07, 122.01, 120.60, 118.25, 114.42, 38.09. HRMS (ESI) m/z calcd for $C_{23}H_{17}ClN_5O^+$ $(M+H)^+$ 413.1043, found 414.1126.

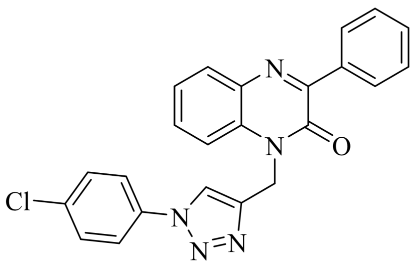

1-((1-(4-chlorophenyl)-1H-1,2,3-triazol-4-yl)methyl)-3-phenylquinoxalin-2(1H)-one (5f)

Pale yellow powder; Yield 77.6%, m. p. = 205.8-208.4 °C; IR (KB, cm^{-1}): 3850.95, 3740.48, 3606.76, 3128.47, 3083.67, 2308.44, 1750.14, 1652.69, 1593.20, 1539.43, 1504.43, 1454.95, 1296.99, 1236.46, 1182.60,1094.75,1037.22,828.48,756.72, 693.49, 637.51, 524.49. ^1H NMR (300 MHz, $CDCl_3$) δ 8.35 – 8.27 (m, 2H), 8.16 (s, 1H), 8.00 – 7.93 (m, 2H), 7.66 – 7.59 (m, 3H), 7.53 – 7.48 (m, 3H), 7.48 – 7.40 (m, 3H), 7.37 (dd, J = 7.1, 0.9 Hz, 1H), 5.68 (s, 2H, $-CH_2$). ^{13}C NMR (75 MHz, $CDCl_3$) δ 154.49, 153.70, 143.29, 135.66, 135.11, 134.52, 133.22, 132.18, 130.63, 130.38, 129.77, 129.34, 128.06, 124.06, 121.95, 121.47, 114.45, 38.09. HRMS (ESI) m/z calcd for $C_{23}H_{17}ClN_5O^+$ $(M+H)^+$ 413.1043, found 414.1121.

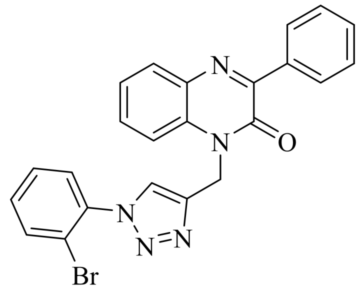

1-((1-(2-bromophenyl)-1H-1,2,3-triazol-4-yl)methyl)-3-phenylquinoxalin-2(1H)-one (5g)

Pale yellow powder; Yield 75.6%, m. p. = 145.7-147.3 °C; IR (KB, cm^{-1}): 3854.12, 3740.21, 3610.21, 3136.10, 3079.59, 2355.88, 1791.84, 1750.66, 1648.31, 1593.46, 1493.09, 1453.50, 1296.81, 1238.46, 1181.82, 1039.30, 933.52, 811.54, 760.81, 689.50, 651.60. ^{1}H NMR (300 MHz, $CDCl_3$) δ 8.37 – 8.25 (m, 2H), 8.13 (s, 1H), 8.04 – 7.92 (m, 2H), 7.74 – 7.69 (m, 1H), 7.67 – 7.60 (m, 1H), 7.52 – 7.32 (m, 7H), 5.71 (s, 2H, $-CH_2$). ^{13}C NMR (75 MHz, $CDCl_3$) δ 154.48, 153.77, 142.12, 136.25, 135.77, 133.83, 133.28, 132.37, 131.21, 130.68, 130.42, 130.39, 129.43, 128.37, 128.09, 128.06, 126.09, 124.05, 118.49, 114.65, 38.18. HRMS (ESI) m/z calcd for $C_{23}H_{17}BrN_5O^+$ $(M+H)^+$ 457.0538, found 458.0691.

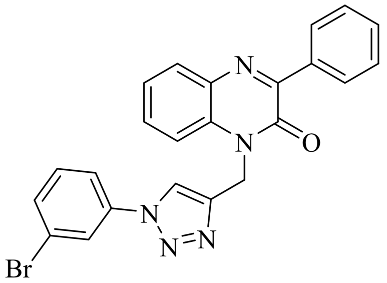

1-((1-(3-bromophenyl)-1H-1,2,3-triazol-4-yl)methyl)-3-phenylquinoxalin-2(1H)-one (5h)

Pale yellow powder; Yield 72.6%, m. p. = 176.5-178.7 °C; IR (KBr, cm^{-1}): 3851.33, 3738.41, 3607.80, 3512.24, 3430.32, 2355.58, 1789.39, 1750.31, 1648.74, 1590.99,

1523.49, 1302.84, 1237.78, 1038.31, 979.89, 767.07, 682.57. ^{1}H NMR (300 MHz, $CDCl_3$) δ 8.35 – 8.27 (m, 2H), 8.17 (s, 1H), 7.99 – 7.92 (m, 2H), 7.89 (t, J = 1.9 Hz, 1H), 7.66 – 7.59 (m, 2H), 7.56 – 7.48 (m, 4H), 7.42 – 7.37 (m, 1H), 7.37 – 7.32 (m, 1H), 5.68 (s, 2H, $-CH_2$). ^{13}C NMR (75 MHz, $CDCl^3$) δ 154.56, 153.77, 143.38, 137.58, 135.71, 133.33, 133.28, 132.23, 131.81, 130.96, 130.70, 130.45, 129.40, 128.12, 124.13, 123.46, 123.19, 122.06, 118.81, 114.48, 38.15. HRMS (ESI) m/z calcd for $C_{23}H_{17}BrN_5O^+$ $(M+H)^+$ 457.0538, found 458.0619.

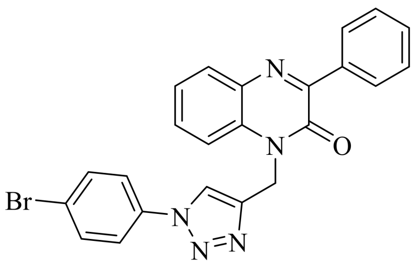

1-((1-(4-bromophenyl)-1H-1,2,3-triazol-4-yl)methyl)-3-phenylquinoxalin-2(1H)-one (5i)

Pale yellow powder; Yield 69.3%, m. p. = 231.2-232.7 °C; IR (KBr, cm^{-1}): 3852.83, 3738.34, 3612.75, 3132.65, 3083.67, 2354.05, 1785.71, 1759.18, 1750.20, 1651.96, 1506.78, 1300.25, 1236.46, 979.02, 828.57, 757.28, 684.44, 417.68.^{1}H NMR (300 MHz, $CDCl_3$) δ 8.38 – 8.24 (m, 2H), 8.16 (s, 1H), 8.02 – 7.91 (m, 2H), 7.67 – 7.54 (m, 5H), 7.54 – 7.45 (m, 3H), 7.38 (t, J = 7.5 Hz, 1H), 5.68 (s, 2H, $-CH_2$). ^{13}C NMR (75 MHz, $CDCl_3$) δ 154.60, 153.85, 145.58, 143.43, 135.73, 133.31, 133.07, 132.82 , 132.25, 132.08, 130.72, 130.46, 129.39, 128.14, 124.14, 121.96, 121.79, 114.51, 38.18. HRMS (ESI) m/z calcd for $C_{23}H_{17}BrN_5O^+$ $(M+H)^+$ 457.0538, found 458.0615.

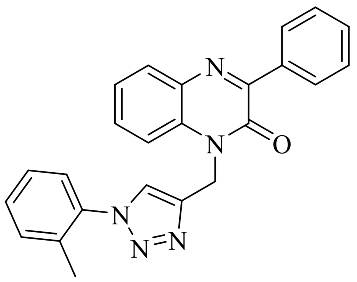

1-((1-2-Tolyl-1H-1,2,3-triazole)-4-methyl)-3-phenylquinoxaline-2(1H)-one (5j)

Pale yellow powder; Yield 70.2%, m. p. = 169.5-172.0 °C; IR (KBr, cm^{-1}): 3869.75, 3739.96, 3621.23, 3140.70, 3089.55, 2356.02, 1750.65, 1651.00, 1602.50, 1504.59, 1464.42, 1296.15, 1230.99, 1180.34, 1045.69, 763.28, 695.69, 531.23. ^{1}H NMR (300 MHz, $CDCl_3$) δ 8.35 – 8.27 (m, 2H), 8.03 (d, J = 8.4 Hz, 1H), 7.99 – 7.92 (m, 2H), 7.68 – 7.61 (m, 1H), 7.53 – 7.47 (m, 3H), 7.43 – 7.36 (m, 2H), 7.37 – 7.30 (m, 2H), 7.29 – 7.24 (m, 2H), 5.71 (s, 2H, -CH_2), 2.19 (s, 3H, -CH_3). ^{13}C NMR (75 MHz, $CDCl_3$) δ 155.35, 154.52, 153.75, 144.88, 142.22, 136.18, 135.80, 133.43, 133.30, 132.42, 131.45, 130.69, 130.41, 129.83, 129.45, 128.10, 126.75, 125.77, 125.42, 124.05, 114.68, 38.27, 17.93. HRMS (ESI) m/z calcd for $C_{24}H_{20}N_5O^+$ $(M+H)^+$ 393.15896, found 394.1667.

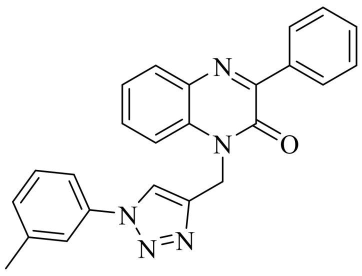

1-((1-3-Tolyl-1H-1,2,3-triazole)-4-methyl)-3-phenylquinoxaline-2(1H)-one (5k)

Pale yellow powder; Yield 71.5%, m. p. = 175.9-177.4 °C; IR (KBr, cm^{-1}): 3861.06, 3739.21, 3604.94, 3140.85, 3074.07, 2356.01, 1750.92, 1650.17, 1608.88, 1504.27, 1456.38, 1299.76, 1239.77, 1175.65, 1046.01, 931.01, 765.88, 689.57, 650.29, 532.92.

^{1}H NMR (300 MHz, $CDCl_3$) δ 8.38 – 8.26 (m, 2H), 8.16 (s, 1H), 8.02 – 7.92 (m, 2H), 7.66 – 7.59 (m, 1H), 7.54 – 7.44 (m, 5H), 7.41 – 7.37 (m, 1H), 7.36 – 7.31 (m, 1H), 7.21 (d, J = 7.7 Hz, 1H), 5.69 (s, 2H, $-CH_2$), 2.40 (s, 3H, $-CH_3$). ^{13}C NMR (75 MHz, $CDCl_3$) δ 154.60, 153.84, 143.17, 143.01, 139.90, 136.68, 135.81, 133.30, 132.32, 130.71, 130.42, 129.57, 129.43, 128.14, 124.09, 122.13, 122.11, 121.03, 117.51,114.63, 38.28, 21.32. HRMS (ESI) m/z calcd for $C_{24}H_{20}N_5O^+$ $(M+H)^+$ 393.15896, found 394.1665.

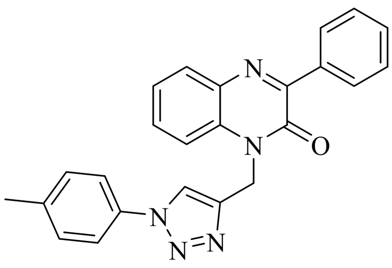

1-((1-4-Tolyl-1H-1,2,3-triazole)-4-methyl)-3-phenylquinoxaline-2(1H)-one (5l)

Pale yellow powder; Yield 72.3%, m. p. = 227.6-229.3 °C; IR (KBr, cm^{-1}): 3860.77, 3745.44, 3616.03, 3132.03, 3074.19, 2355.97, 1751.08, 1648.44, 1591.23, 1520.46, 1455.28, 1293.25, 1235.02, 1181.72, 1041.70, 932.65, 813.19, 754.25, 685.56, 528.57.^{1}H NMR (300 MHz, $CDCl_3$) δ 8.36 – 8.27 (m, 2H), 8.12 (s, 1H), 8.00 (d, J = 7.7 Hz, 1H), 7.94 (dd, J = 8.0, 1.4 Hz, 1H), 7.65 – 7.58 (m, 1H), 7.57 – 7.47 (m, 5H), 7.41 – 7.35 (m, 1H), 7.28 – 7.24 (m, 2H), 5.68 (s, 2H, $-CH_2$), 2.38 (s, 3H, $-CH_3$). ^{13}C NMR (75 MHz, $CDCl_3$) δ 154.60, 143.45, 142.95, 138.97, 135.80, 134.47, 134.39, 133.29, 132.33, 130.70, 130.40, 130.14, 129.42, 128.12, 124.07, 122.01, 120.33, 114.65, 38.28, 21.02. HRMS (ESI) m/z calcd for $C_{24}H_{20}N_5O^+$ $(M+H)^+$ 393.441, found 394.1672.

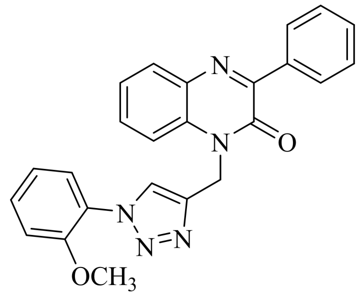

1-((1-(2-ethylphenyl)-1H-1,2,3-triazol-4-yl)methyl)-3-phenylquinoxalin-2(1H)-one (5m)

Brown powder; Yield 68.6%, m. p. = 176.6-179.1 °C; IR (KBr, cm^{-1}): 3853.84, 3740.35, 3611.19, 3077.77, 2961.78, 2355.96, 1696.06, 1648.31, 1604.22, 1508.31, 1468.08, 1288.85, 1248.02, 1178.77, 1118.37, 1032.57, 808.16, 761.45, 687.94, 648.98, 524.83.^{1}H NMR (300 MHz, $CDCl_3$) δ 8.37 – 8.23 (m, 3H), 8.05 (d, J = 8.5 Hz, 1H), 7.93 (dd, J = 8.0, 1.3 Hz, 1H), 7.70 – 7.58 (m, 2H), 7.53 – 7.45 (m, 3H), 7.40 – 7.33 (m, 2H), 7.03 (t, J = 7.9 Hz, 2H), 5.69 (s, 2H, $-CH_2$), 3.82 (s, 3H, $-CH_3$). ^{13}C NMR (75 MHz, $CDCl_3$) δ 154.42, 153.76, 150.95, 141.69, 137.14, 135.84, 133.20, 132.40, 130.57, 130.27, 130.12, 129.36, 128.02, 126.01, 125.92, 125.35, 123.90, 120.96, 114.78, 111.99, 55.81, 38.17. HRMS (ESI) m/z calcd for $C_{24}H_{20}N_5O_2^+$ $(M+H)^+$ 409.1539, found 410.1617.

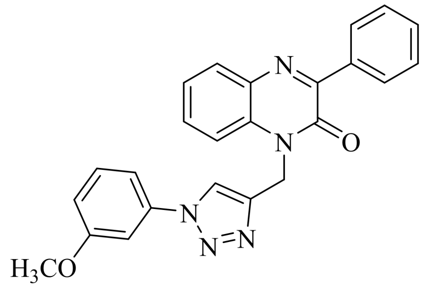

1-((1-(3-ethylphenyl)-1H-1,2,3-triazol-4-yl)methyl)-3-phenylquinoxalin-2(1H)-one (5n)

Brown powder; Yield 67.4%, m. p. = 177.4-181.0 °C; IR (KBr, cm^{-1}): 3854.08, 3740.47, 3610.08, 3135.45, 3075.51, 2355.81, 1750.78, 1648.15, 1601.83, 1506.33, 1458.20, 1301.60, 1248.72, 1146.10, 1043.31, 851.73, 760.08, 685.07. ^{1}H NMR (300 MHz, $CDCl_3$) δ 8.35 – 8.27 (m, 2H), 8.15 (s, 1H), 8.02 – 7.92 (m, 2H), 7.65 – 7.58 (m, 1H), 7.53 – 7.47 (m, 3H), 7.39 (d, J = 7.1 Hz, 1H), 7.34 (d, J = 8.1 Hz, 1H), 7.28 – 7.26 (m, 1H), 7.23 – 7.19 (m, 1H), 6.93 (dd, J = 8.3, 2.4 Hz, 1H), 5.69 (s, 2H, $-CH_2$), 3.84 (s, 3H, $-CH_3$). ^{13}C NMR (75 MHz, $CDCl_3$) δ 160.48, 154.57, 153.81, 146.45, 143.08, 137.74, 135.79, 133.39, 133.30, 132.32, 130.68, 130.43, 129.43, 128.12, 124.08, 122.12, 114.86, 144.62, 112.33, 106.04, 55.56, 38.23. HRMS (ESI) m/z calcd for $C_{24}H_{20}N_5O_2^+$ $(M+H)^+$ 409.1539, found 410.1613.

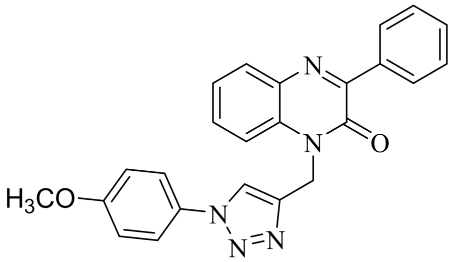

1-((1-(4-ethylphenyl)-1H-1,2,3-triazol-4-yl)methyl)-3-phenylquinoxalin-2(1H)-one (5o)

Brown powder; Yield 70.0%, m. p. = 213.0-214.9 °C;IR (KBr, cm^{-1}): 3853.37, 3740.17, 3612.58, 3130.61, 3075.51, 2355.84, 1750.97, 1696.05, 1651.20, 1522.85, 1469.20, 1248.89, 969.39, 757.14.1, 685.16. ^{1}H NMR (300 MHz, $CDCl_3$) δ 8.35 – 8.28 (m, 2H), 8.08 (s, 1H), 8.01 (d, J = 8.0 Hz, 1H), 7.95 (dd, J = 8.0, 1.4 Hz, 1H), 7.66 – 7.61 (m, 1H), 7.60 – 7.55 (m, 2H), 7.53 – 7.47 (m, 3H), 7.41 – 7.35 (m, 1H), 6.97 (d, J = 9.1 Hz, 2H), 5.68 (s, 2H, $-CH_2$), 3.84 (s, 3H, $-OCH_3$). ^{13}C NMR (75 MHz, $CDCl_3$) δ 159.81, 154.58, 153.81, 142.19, 135.82, 135.73, 135.31, 133.30, 132.35,

130.68, 130.40, 130.20, 129.43, 128.11, 124.05, 122.14, 122.03, 114.66, 55.53, 38.28. HRMS (ESI) m/z calcd for $C_{24}H_{20}N_5O_2^+$ $(M+H)^+$ 409.1539, found 410.1619.

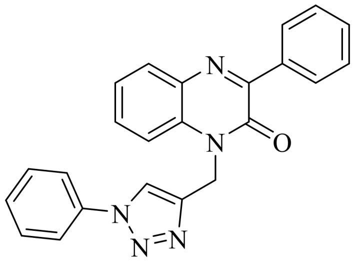

1-((1-Phenyl-1H-1,2,3-triazole)-4-methyl)-3-phenylquinoxaline-2(1H)-one (5p)

Pale yellow powder; Yield 77.4%, m. p. = 233.8-235.7 °C; IR (KBr, cm^{-1}): 3853.99, 3742.55, 3615.57, 3554.49, 3135.08, 3086.21, 2308.19, 1789.49, 1750.81, 1696.12, 1647.41, 1594.05, 1507.31, 1301.82, 1239.08, 1045.77, 769.35, 689.05. ^{1}H NMR (300 MHz, $CDCl_3$) δ 8.39 – 8.25 (m, 2H), 8.17 (s, 1H), 8.03 – 7.92 (m, 2H), 7.71 – 7.65 (m, 2H), 7.64 – 7.58 (m, 1H), 7.54 – 7.45 (m, 5H), 7.45 – 7.34 (m, 3H), 5.70 (s, 2H, $-CH_2$). ^{13}C NMR (75 MHz, $CDCl_3$) δ 154.58, 153.83, 143.13, 136.74, 135.79, 133.30, 132.32, 130.69, 130.42, 130.40, 129.65, 129.42, 128.82, 128.11, 124.07, 122.05, 120.42, 114.60, 38.24. HRMS (ESI) m/z calcd for $C_{23}H_{18}N_5O^+$ $(M+H)^+$ 379.1433, found 380.1513.

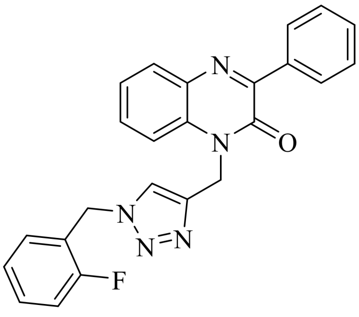

1-((1-(2-fluorobenzyl)-1H-1,2,3-triazol-4-yl)methyl)-3-phenylquinoxalin-2(1H)-one (6a)

Pale yellow powder; Yield 70.6%, m. p. = 163.8-165.0 °C; IR (KBr, cm^{-1}): 3853.73, 3740.99, 3612.12, 3110.20, 3070.24, 2356.75, 1751.14, 1695.70, 1650.25, 1591.25, 1489.25, 1453.76, 1289.52, 1251.86, 1219.33, 1178.16, 1059.80, 929.58, 766.29, 686.75,532.44.^{1}H NMR (300 MHz, $CDCl_3$) δ 8.33 – 8.22 (m, 2H), 7.98 – 7.88 (m, 2H), 7.68 (s, 1H), 7.63 – 7.56 (m, 1H), 7.52 – 7.43 (m, 3H), 7.40 – 7.28 (m, 2H), 7.06 – 6.97 (m, 2H), 6.93 (d, J = 9.2 Hz, 1H), 5.58 (s, 2H, -CH_2), 5.43 (s, 2H, -CH_2). ^{13}C NMR (75 MHz, $CDCl_3$) δ 164.49, 154.48, 153.75, 142.95, 136.54, 136.44, 135.76, 133.24, 132.31, 130.76, 130.63, 130.36, 129.37, 128.09, 124.02, 123.84, 123.68, 123.64, 115.95, 115.68, 115.26, 114.97, 114.60, 53.53, 38.25. HRMS (ESI) m/z calcd for $C_{24}H_{19}FN_5O^+$ $(M+H)^+$ 411.1495, found 412.1573.

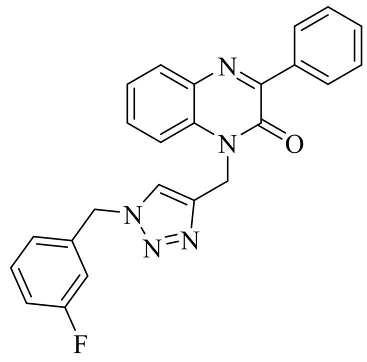

1-((1-(3-fluorobenzyl)-1H-1,2,3-triazol-4-yl)methyl)-3-phenylquinoxalin-2(1H)-one (6b)

Pale yellow powder; Yield 71.3%, m. p. = 154.9-157.3 °C; IR (KBr, cm^{-1}): 3854.80, 3741.82, 3611.99, 3126.69, 3077.55, 2357.45, 1750.81, 1643.55, 1591.58, 1492.55, 1455.33, 1288.93, 1235.77, 1186.24, 1137.76, 1036.19, 924.49, 855.10, 806.12, 761.31, 687.63, 535.11, 420.66. ^{1}H NMR (300 MHz, $CDCl_3$) δ 8.34 – 8.20 (m, 2H),

7.99 – 7.89 (m, 2H), 7.71 (s, 1H), 7.62 – 7.55 (m, 1H), 7.52 – 7.45 (m, 3H), 7.39 – 7.28 (m, 2H), 7.22 (dd, *J* = 7.4, 1.6 Hz, 1H), 7.14 – 7.04 (m, 2H), 5.58 (s, 2H, $-CH_2$), 5.51 (s, 2H, $-CH_2$). ^{13}C NMR (75 MHz, $CDCl_3$) δ 162.08, 158.79, 154.45, 153.76, 142.75, 135.80, 133.22, 132.34, 130.97, 130.86, 130.60, 130.35, 130.31, 129.39, 128.08, 124.75, 124.71, 123.97, 123.86, 121.37, 115.97, 115.69, 114.66, 47.68, 38.23. HRMS (ESI) m/z calcd for $C_{24}H_{19}FN_5O^+$ $(M+H)^+$ 411.1495, found 412.1571.

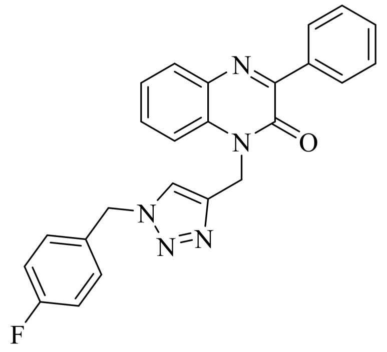

1-((1-(4-fluorobenzyl)-1H-1,2,3-triazol-4-yl)methyl)-3-phenylquinoxalin-2(1H)-one (6c)

Pale yellow powder; Yield 71.8%, m. p. = 170.3-173.2 °C; IR (KBr, cm^{-1}): 3853.98, 3741.62, 3662.51, 3612.71, 3130.27, 3083.72, 2357.80, 1832.77, 1788.92, 1750.69, 1695.99, 1637.31, 1520.57, 1457.16, 1376.99, 1294.67, 1230.49, 1178.11, 762.28, 685.05.^{1}H NMR (300 MHz, $CDCl_3$) δ 8.33 – 8.20 (m, 2H), 7.97 – 7.88 (m, 2H), 7.64 (s, 1H), 7.62 – 7.56 (m, 1H), 7.52 – 7.44 (m, 3H), 7.39 – 7.33 (m, 1H), 7.26 – 7.20 (m, 2H), 7.06 – 6.96 (m, 2H), 5.57 (s, 2H, $-CH_2$), 5.40 (s, 2H, $-CH_2$). ^{13}C NMR (75 MHz, $CDCl_3$) δ 164.44, 161.15, 154.48, 153.76, 143.66, 142.88, 135.76, 133.22, 132.31, 130.63, 130.39, 130.34, 130.14, 130.03, 129.36, 128.10, 124.01, 123.63, 116.21, 115.92, 114.62, 53.46, 38.25. HRMS (ESI) m/z calcd for $C_{24}H_{19}FN_5O^+$ $(M+H)^+$ 411.1495, found 412.1573.

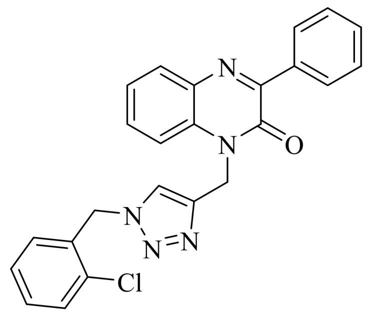

1-((1-(2-chlorobenzyl)-1H-1,2,3-triazol-4-yl)methyl)-3-phenylquinoxalin-2(1H)-one (6d)

Pale yellow powder; Yield 76.8%, m. p. = 167.9-169.7 °C; IR (KBr, cm^{-1}): 3853.27, 3741.53, 3612.87, 3118.48, 3065.31, 2358.86, 2318.37, 1750.78, 1695.86, 1650.88, 1523.60, 1470.54, 1378.41, 1299.52, 1227.93, 1219.33, 1177.55, 1059.32, 931.58, 767.96, 690.66.^{1}H NMR (300 MHz, $CDCl_3$) δ 8.31 – 8.23 (m, 2H), 7.97 – 7.89 (m, 2H), 7.67 (s, 1H), 7.60 (ddd, J = 8.5, 7.4, 1.5 Hz, 1H), 7.51 – 7.45 (m, 3H), 7.40 – 7.28 (m, 3H), 7.23 (s, 1H), 7.11 (d, J = 6.9 Hz, 1H), 5.58 (s, 2H, -CH_2), 5.41 (s, 2H, -CH_2). ^{13}C NMR (75 MHz, $CDCl_3$) δ 154.42, 153.67, 142.91, 136.03, 135.70, 134.82, 133.18, 132.25, 130.56, 130.29, 129.32, 128.92, 128.14, 128.02, 126.13, 123.94, 123.77, 114.53, 53.41, 38.18. HRMS (ESI) m/z calcd for $C_{24}H_{19}ClN_5O^+$ $(M+H)^+$ 427.1200, found 428.1278.

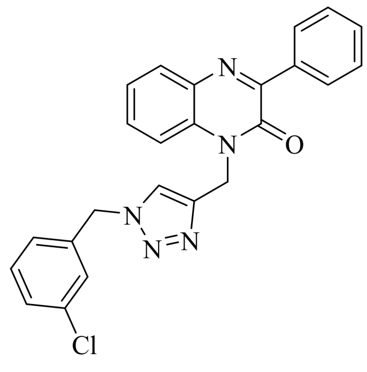

1-((1-(3-chlorobenzyl)-1H-1,2,3-triazol-4-yl)methyl)-3-phenylquinoxalin-2(1H)-one (6e)

Pale yellow powder; Yield 68.7%, m. p. = 152.5-154.6 °C; IR (KBr, cm^{-1}): 3854.23, 3741.83, 3609.88, 3130.39, 3073.47, 2359.27, 2318.37, 1750.74, 1594.18, 1454.55, 1292.75, 1188.97, 1045.72, 940.82, 756.19, 692.09, 533.79. ^{1}H NMR (300 MHz, $CDCl_3$) δ 8.34 – 8.22 (m, 2H), 7.99 – 7.89 (m, 2H), 7.72 (s, 1H), 7.63 – 7.56 (m, 1H), 7.53 – 7.45 (m, 3H), 7.42 – 7.35 (m, 2H), 7.34 – 7.28 (m, 1H), 7.24 – 7.14 (m, 2H), 5.59 (s, 4H, $-CH_2$, $-CH_2$). ^{13}C NMR (75 MHz, $CDCl_3$) δ 154.39, 153.71, 135.76, 133.46, 133.18, 132.30, 131.93, 130.53, 130.28, 130.17, 129.83, 129.34, 128.01, 127.43, 123.91, 114.64,51.38, 38.20. HRMS (ESI) m/z calcd for $C_{24}H_{19}ClN_5O^+$ $(M+H)^+$ 427.1200, found 428.1275.

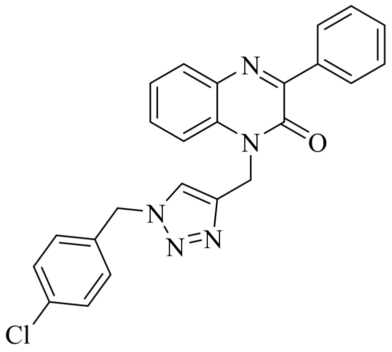

1-((1-(4-chlorobenzyl)-1H-1,2,3-triazol-4-yl)methyl)-3-phenylquinoxalin-2(1H)-one (6f)

Pale yellow powder; Yield 72.4%, m. p. = 188.6-190.3 °C; IR (KBr, cm^{-1}): 3854.27, 3742.91, 3609.05, 3556.93, 3153.06, 3083.67, 2308.59, 1789.24, 1750.94, 1647.44, 1593.91, 1507.44, 1379.04, 1301.45, 1239.50, 1045.13, 768.71, 688.41.^{1}H NMR (300 MHz, $CDCl_3$) δ 8.33 – 8.21 (m, 2H), 7.98 – 7.87 (m, 2H), 7.65 – 7.56 (m, 2H), 7.53 – 7.45 (m, 3H), 7.40 – 7.33 (m, 1H), 7.26 – 7.20 (m, 2H), 7.06 – 6.97 (m, 2H), 5.57 (s, 2H, $-CH_2$), 5.41 (s, 2H, $-CH_2$). ^{13}C NMR (75 MHz, $CDCl_3$) δ 153.73, 142.84, 135.71, 133.18, 132.26, 130.57, 130.30, 130.06, 129.31, 128.04, 123.95, 123.55, 123.51,

123.32, 116.17, 115.88, 114.56, 90.26, 53.42, 38.21. HRMS (ESI) m/z calcd for $C^{24}H^{19}ClN^{5}O^{+}$ $(M+H)^{+}$ 427.1200, found 428.1281.

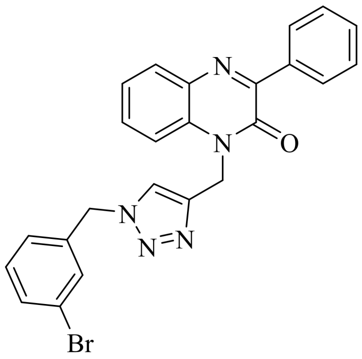

1-((1-(3-bromobenzyl)-1H-1,2,3-triazol-4-yl)methyl)-3-phenylquinoxalin-2(1H)-one (6g)

Pale yellow powder; Yield 72.4%, m. p. = 166.4-168.6 °C; IR (KBr, cm^{-1}): 3852.40, 3740.61, 3608.38, 3118.79, 3067.89, 2356.41, 1752.65, 1650.52, 1590.68, 1524.00, 1469.78, 1298.61, 1226.93, 1048.20, 768.12, 692.12. ^{1}H NMR (300 MHz, $CDCl_3$) δ 8.35 – 8.21 (m, 2H), 7.99 – 7.88 (m, 2H), 7.68 (s, 1H), 7.62 – 7.55 (m, 1H), 7.53 – 7.44 (m, 3H), 7.40 – 7.26 (m, 3H), 7.23 (s, 1H), 7.11 (d, J = 7.0 Hz, 1H), 5.58 (s, 2H, $-CH_2$), 5.41 (s, 2H, $-CH_2$). ^{13}C NMR (75 MHz, $CDCl_3$) δ 154.44, 153.70, 142.92, 136.02, 135.71, 134.84, 133.19, 132.26, 130.59, 130.31, 129.33, 128.95, 128.17, 128.05, 126.16, 123.97, 123.80, 114.55, 53.45, 38.20 . HRMS (ESI) m/z calcd for $C_{24}H_{19}BrN_5O^{+}$ $(M+H)^{+}$ 471.0694, found 472.0769.

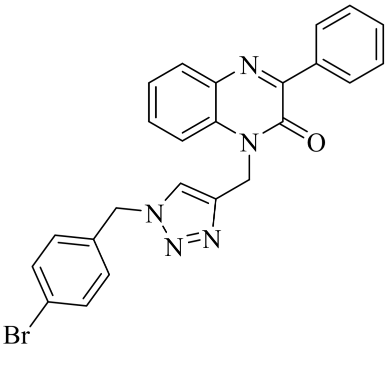

1-((1-(4-bromobenzyl)-1H-1,2,3-triazol-4-yl)methyl)-3-phenylquinoxalin-2(1H)-one (6h)

Pale yellow powder; Yield 75.3%, m. p. = 196.4-198.2 °C; IR (KBr, cm^{-1}): 3854.09, 3742.06, 3610.46, 3130.61, 3085.71, 2361.93, 2315.42, 1750.98, 1695.77, 1645.51, 1524.02, 1471.67, 1302.04, 1236.73, 942.68, 755.10, 691.84. ^1H NMR (300 MHz, $CDCl_3$) δ 8.32 – 8.22 (m, 2H), 7.98 – 7.89 (m, 2H), 7.67 – 7.56 (m, 2H), 7.51 – 7.43 (m, 5H), 7.40 – 7.34 (m, 1H), 7.11 (d, J = 8.4 Hz, 2H), 5.58 (s, 2H, $-CH_2$), 5.39 (s, 2H, $-CH_2$). ^{13}C NMR (75 MHz, $CDCl_3$) δ 154.45, 153.73, 142.91, 135.71, 133.14, 133.19, 133.08, 132.26, 13219, 130.60, 130.35, 129.74, 129.33, 128.07, 123.99, 123.67, 122.94, 114.56, 53.50, 38.22. HRMS (ESI) m/z calcd for $C_{24}H_{19}BrN_5O^+$ $(M+H)^+$ 471.0694, found 472.0771.

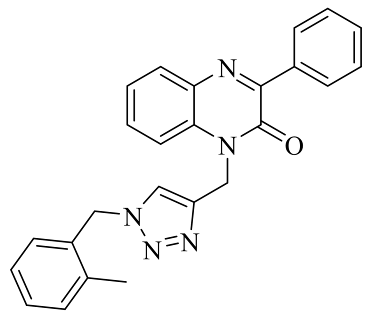

1-((1-(2-methylbenzyl)-1H-1,2,3-triazol-4-yl)methyl)-3-phenylquinoxalin-2(1H)-one (6i)

Pale yellow powder; Yield 70.3%, m. p. = 167.3-169.8 °C; IR (KBr, cm^{-1}): 3851.55, 3740.44, 3608.17, 3134.69, 3073.47, 2356.45, 2326.53, 1750.37, 1695.49, 1648.18, 1523.32, 1455.85, 1296.77 1232.65, 1181.63, 1055.10, 969.39, 753.45, 685.71, 410.01.^1H NMR (300 MHz, $CDCl_3$) δ 8.33 – 8.20 (m, 2H), 7.99 – 7.89 (m, 2H), 7.62 – 7.56 (m, 1H), 7.53 (s, 1H), 7.51 – 7.45 (m, 3H), 7.39 – 7.33 (m, 1H), 7.26 – 7.21 (m,

1H), 7.21 – 7.14 (m, 2H), 7.14 – 7.09 (m, 1H), 5.57 (s, 2H, $-CH_2$), 5.46 (s, 2H, $-CH_2$), 2.25 (s, 3H, $-CH_3$). ^{13}C NMR (75 MHz, $CDCl_3$) δ 154.45, 153.76, 142.60, 136.82, 135.83, 133.25, 132.37, 132.11, 130.99, 130.99, 130.61, 130.33, 129.66, 129.52, 129.40, 129.15, 128.09, 126.63, 123.97, 123.49, 114.73, 52.37, 38.28, 18.98. HRMS (ESI) m/z calcd for $C_{25}H_{22}N_5O^+$ $(M+H)^+$ 407.1746, found 408.1238.

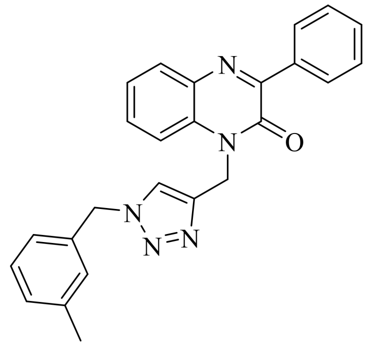

1-((1-(3-methylbenzyl)-1H-1,2,3-triazol-4-yl)methyl)-3-phenylquinoxalin-2(1H)-one (6j)

Pale yellow powder; Yield 68.6%, m. p. = 133.9-135.5 °C; IR (KBr, cm^{-1}): 3854.12, 3741.76, 3609.77, 3120.41, 3069.39, 2951.02, 2360.11, 1749.87, 1650.93, 1594.65, 1524.65, 1468.59, 1299.16, 1231.20, 1177.45, 1048.71, 926.53, 760.24, 692.11. ^{1}H NMR (300 MHz, $CDCl_3$) δ 8.34 – 8.22 (m, 2H), 7.93 (dd, J = 13.6, 5.2 Hz, 2H), 7.63 (s, 1H), 7.59 (t, J = 7.2 Hz, 1H), 7.52 – 7.44 (m, 3H), 7.36 (t, J = 7.7 Hz, 1H), 7.25 – 7.19 (m, 1H), 7.13 (d, J = 7.8 Hz, 1H), 7.04 (d, J = 6.8 Hz, 2H), 5.58 (s, 2H, $-CH_2$), 5.40 (s, 2H, $-CH_2$), 2.30 (s, 3H, $-CH_3$). ^{13}C NMR (75 MHz, $CDCl_3$) δ 154.49, 153.80, 142.75, 138.90, 135.83, 134.05, 133.25, 132.37, 130.63, 130.36, 129.67, 12955, 129.40, 129.06, 128.94, 128.10, 125.28, 123.99, 123.66, 114.71, 54.28, 38.32, 21.27. HRMS (ESI) m/z calcd for $C_{25}H_{22}N_5O^+$ $(M+H)^+$ 407.1746, found 408.1825.

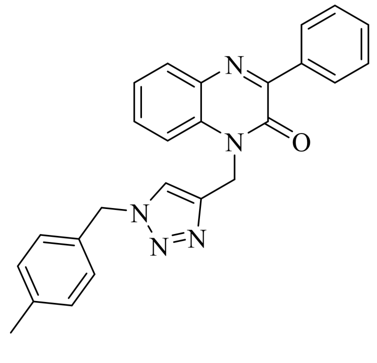

1-((1-(4-methylbenzyl)-1H-1,2,3-triazol-4-yl)methyl)-3-phenylquinoxalin-2(1H)-one (6k)

Pale yellow powder; Yield 68.3%, m. p. = 196.1-198.1 °C; IR (KBr,cm^{-1}): 3853.61, 3741.59, 3609.27, 3122.45, 3077.55, 2363.27, 2317.33, 1750.62, 1638.80, 1522.97, 1455.91, 1294.78, 1234.28, 1130.92, 1032.15, 932.65, 767.35, 688.10. ^{1}H NMR (300 MHz, $CDCl_3$) δ 8.33 – 8.21 (m, 2H), 7.93 (t, J = 8.8 Hz, 2H), 7.64 – 7.53 (m, 2H), 7.51 – 7.44 (m, 3H), 7.35 (t, J = 7.5 Hz, 1H), 7.13 (s, 4H), 5.55 (s, 2H, $-CH_2$), 5.38 (s, 2H, $-CH_2$), 2.31 (s, 3H, $-CH_3$). ^{13}C NMR (75 MHz, $CDCl_3$) δ 154.48, 153.79, 142.70, 138.73, 135.84, 133.25, 132.38, 131.11, 130.63, 130.35, 130.33, 129.74, 129.40, 128.25, 128.10, 123.98, 123.56, 114.73, 54.08, 38.31, 21.12. HRMS(ESI) m/z calcd for $C_{25}H_{22}N_5O^+$ $(M+H)^+$ 407.1746, found 408.1820.

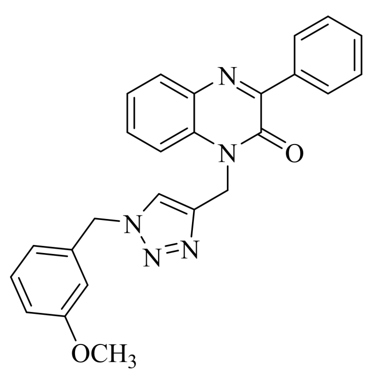

1-((1-(3-methoxybenzyl)-1H-1,2,3-triazol-4-yl)methyl)-3-phenylquinoxalin-2(1H)-one (6l)

Brown powder; Yield 67.3%, m. p. = 139.4-141.8 °C; IR (KBr, cm^{-1}): 3861.14, 3740.40, 3623.84, 3133.93, 3071.10, 2361.33, 1649.35, 1595.86, 1455.77, 1283.73, 1167.82, 1049.17, 762.05, 691.39. ^{1}H NMR (300 MHz, $CDCl_3$) δ 8.33 – 8.22 (m, 2H), 7.94 (t, J = 8.2 Hz, 2H), 7.65 (s, 1H), 7.59 (t, J = 7.7 Hz, 1H), 7.52 – 7.45 (m, 3H), 7.36 (t, J = 7.5 Hz, 1H), 7.23 (d, J = 7.8 Hz, 1H), 6.88 – 6.79 (m, 2H), 6.74 (s, 1H), 5.58 (s, 2H, $-CH_2$), 5.41 (s, 2H, $-CH_2$), 3.74 (s, 3H, $-OCH_3$). ^{13}C NMR (75 MHz, $CDCl_3$) δ 159.99, 154.48, 153.78, 142.79, 135.82, 135.58, 133.26, 132.37, 130.62, 132.37, 130.62, 130.35, 130.14, 129.74, 129.41, 128.09, 123.98, 123.73, 120.34, 114.68, 114.24, 113.74, 55.20, 54.19, 38.29. HRMS (ESI) m/z calcd for $C_{25}H_{22}N_5O_2^+$ $(M+H)^+$ 423.1695, found 424.1773.

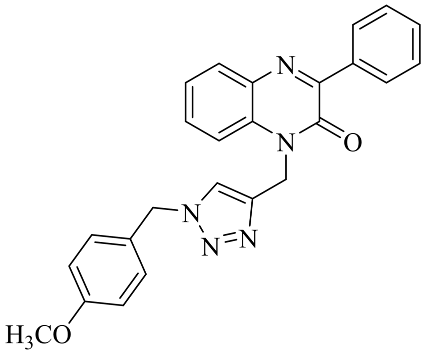

1-((1-(4-methoxybenzyl)-1H-1,2,3-triazol-4-yl)methyl)-3-phenylquinoxalin-2(1H)-one (6m)

Brown powder; Yield 67.3%, m. p. = 139.4-141.8 °C; IR (KBr, cm^{-1}): 3854.50, 3743.01, 3556.85, 3134.69, 3073.47, 2944.90, 2832.65, 2312.24, 1750.90, 1650.62, 1603.19, 1515.98, 1455.08, 1297.73, 1250.08, 1180.31, 1180.29, 1040.63, 924.49, 769.84, 693.92, 537.75. ^{1}H NMR (300 MHz, $CDCl_3$) δ 8.32 – 8.23 (m, 2H), 7.98 – 7.89 (m, 2H), 7.62 – 7.55 (m, 2H), 7.52 – 7.45 (m, 3H), 7.36 (t, J = 7.6 Hz, 1H), 7.19 (d, J = 8.6 Hz, 2H), 6.85 (d, J = 8.6 Hz, 2H), 5.57 (s, 2H, $-CH_2$), 5.37 (s, 2H, $-CH_2$),

3.78 (s, 3H, -OCH_3). ^{13}C NMR (75 MHz, $CDCl_3$) δ 159.89, 154.48, 153.77, 142.68, 135.84, 133.24, 132.37, 130.62, 130.35, 130.32, 129.77, 129.40, 128.09, 126.13, 123.97, 123.43, 114.72, 114.43, 55.24, 53.80, 38.31. HRMS (ESI) m/z calcd for $C_{25}H_{22}N_5O_2^+$ $(M+H)^+$ 423.1695, found 424.1770.

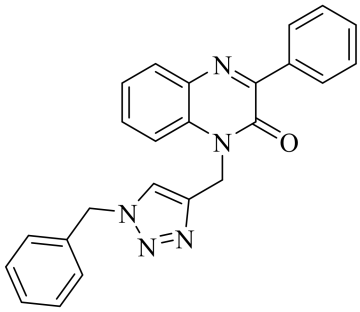

1-((1-benzyl-1H-1,2,3-triazol-4-yl)methyl)-3-phenylquinoxalin-2(1H)-one (6n)

Pale yellow powder; Yield 75.5%, m. p. = 183.7-184.8 °C; IR (KBr, cm^{-1}): 3854.79, 3743.42, 3610.58, 3132.81, 3081.63, 2373.47, 2318.37, 1751.00, 1637.92, 1592.79, 1455.45, 1289.09, 1184.31, 1034.69, 942.86, 765.04, 727.33, 555.10.^1H NMR (300 MHz, $CDCl_3$) δ 8.34 – 8.21 (m, 2H), 7.92 (t, J = 7.7 Hz, 2H), 7.65 (s, 1H), 7.56 (t, J = 7.8 Hz, 1H), 7.51 – 7.43 (m, 3H), 7.38 – 7.28 (m, 4H), 7.25 – 7.18 (m, 2H), 5.55 (s, 2H, -CH_2), 5.42 (s, 2H, -CH_2). ^{13}C NMR (75 MHz, $CDCl_3$) δ 154.47, 153.76, 142.78, 135.81, 134.16, 133.24, 132.35, 130.61, 130.33, 129.39, 129.05, 128.76, 128.16, 128.08, 123.97, 123.70, 114.68, 114.43, 55.24, 38.29. HRMS (ESI) m/z calcd for $C_{24}H_{20}N_5O^+$ $(M+H)^+$ 393.1589, found 494.1664.

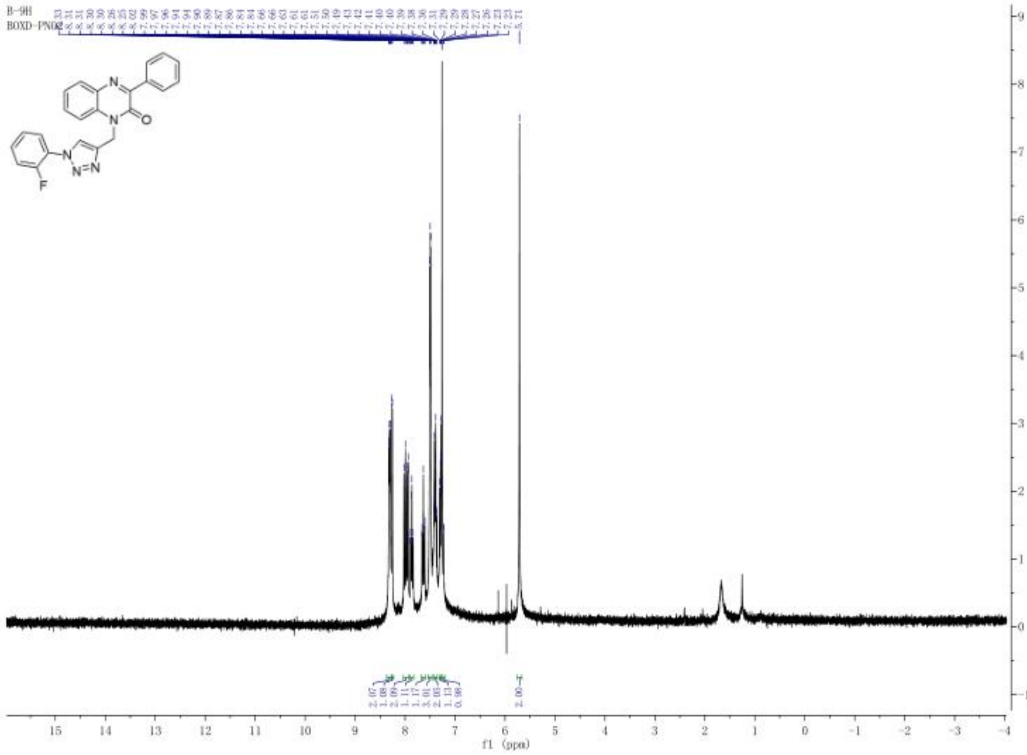

^{1}H-NMR (300 MHz, $CDCl_3$) spectrum of compound **5a**

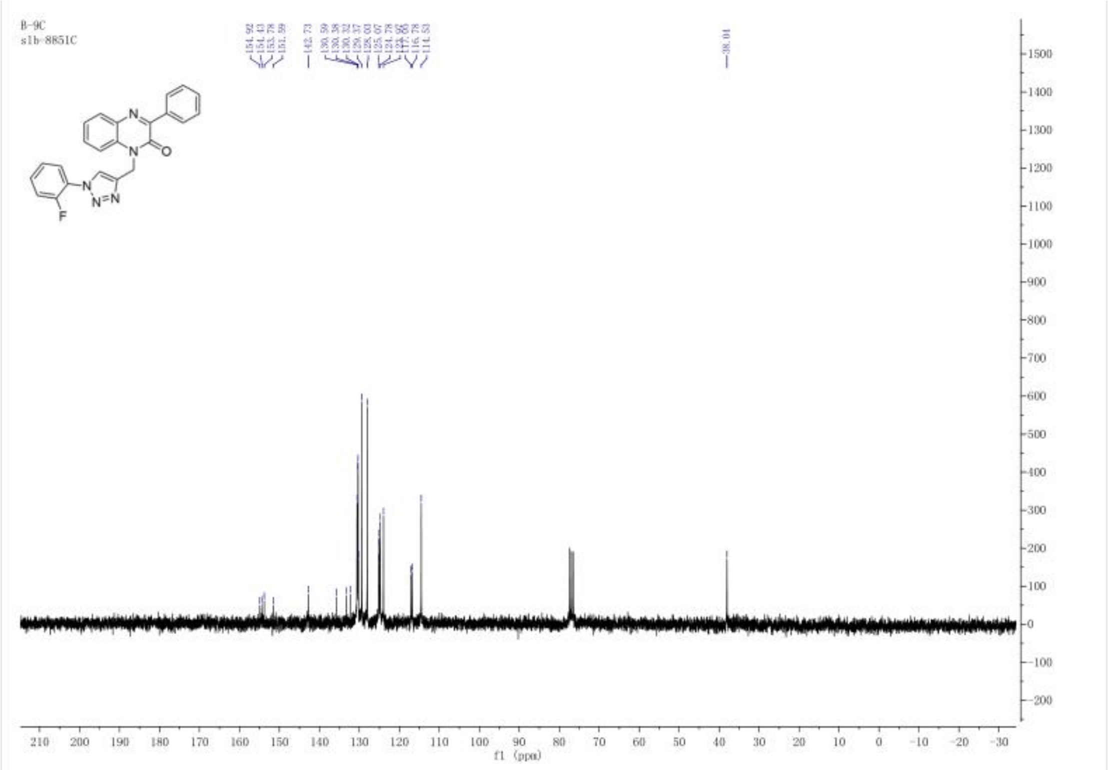

^{13}C NMR (75 MHz, $CDCl_3$) spectrum of compound **5a**

Elemental Composition Report **Page 1**

Single Mass Analysis
Tolerance = 10.0 PPM / DBE: min = -1.5, max = 50.0
Element prediction: Off
Number of isotope peaks used for i-FIT = 3

Monoisotopic Mass, Even Electron Ions
411 formula(e) evaluated with 1 results within limits (up to 50 closest results for each mass)
Elements Used:
C: 23-23 H: 0-80 N: 0-6 O: 0-20 F: 1-3
3
0223-1-9 213 (1.198) 1: TOF MS ES+ 8.46e+006

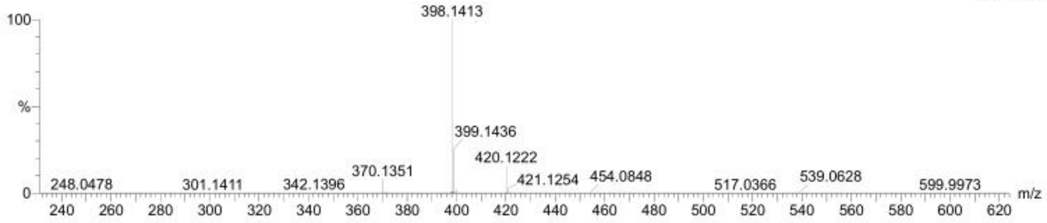

Minimum:				-1.5				
Maximum:		5.0	10.0	50.0				
Mass	Calc. Mass	mDa	PPM	DBE	i-FIT	Norm	Conf(%)	Formula
398.1413	398.1417	-0.4	-1.0	17.5	1095.2	n/a	n/a	C23 H17 N5 O F

HRMS spectrum of compound **5a**

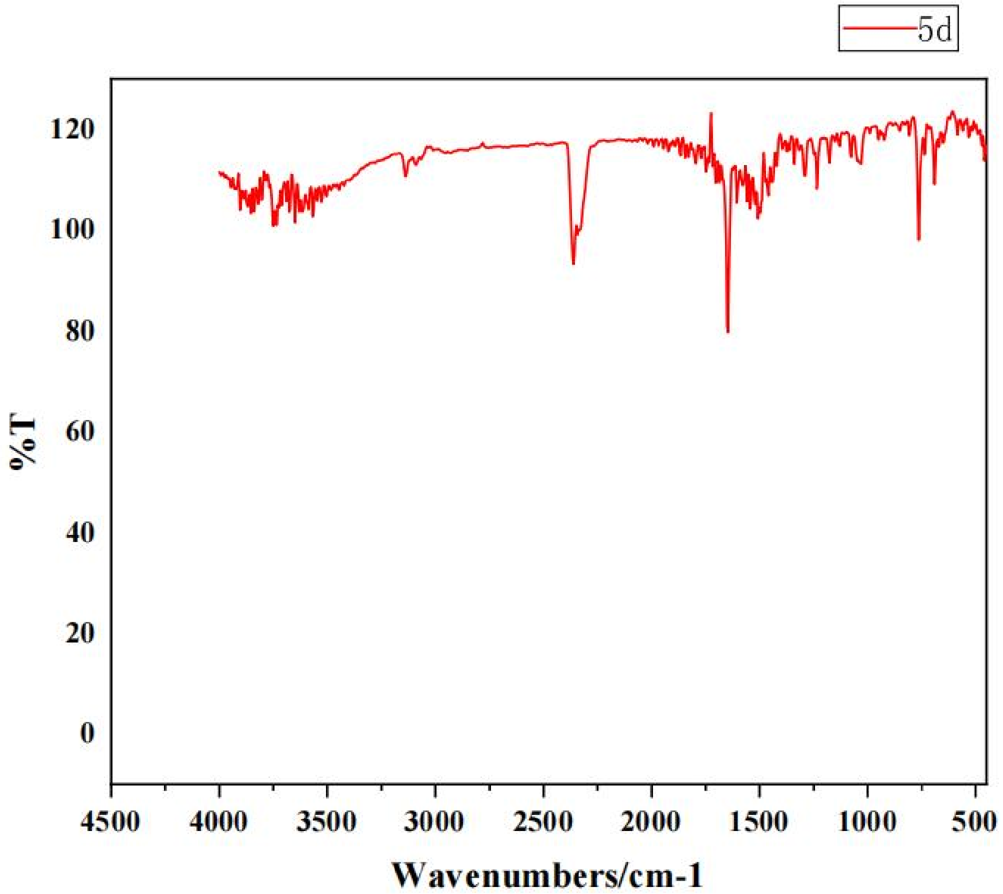

FT-IR spectrum of compound **5a**

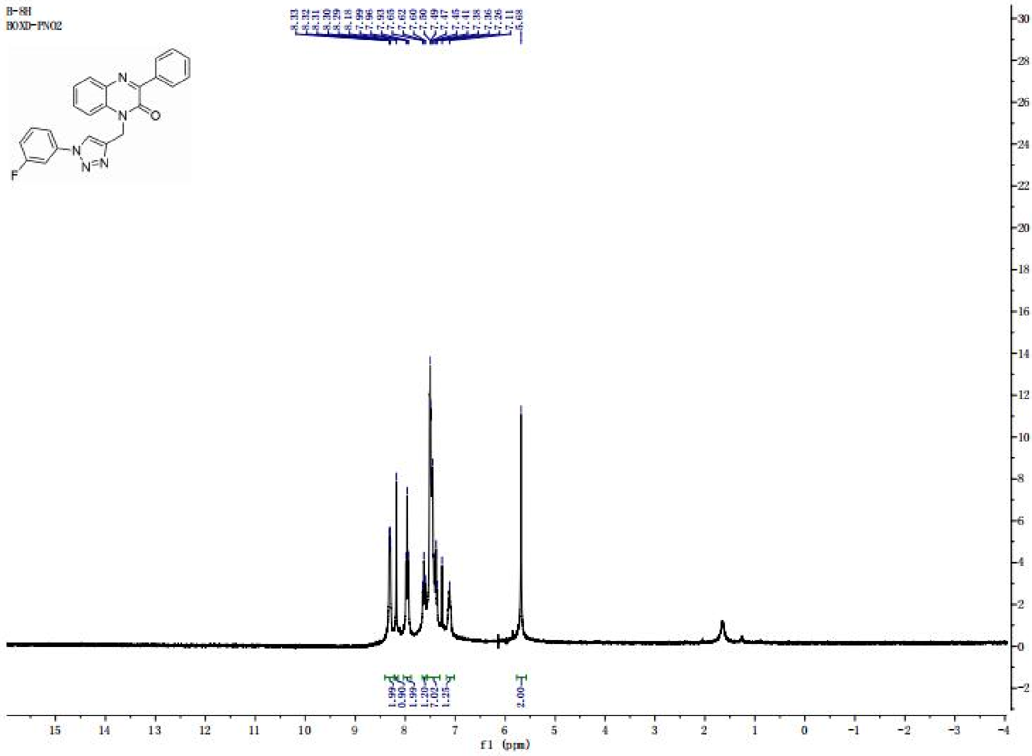

^{1}H-NMR (300 MHz, $CDCl_3$) spectrum of compound **5b**

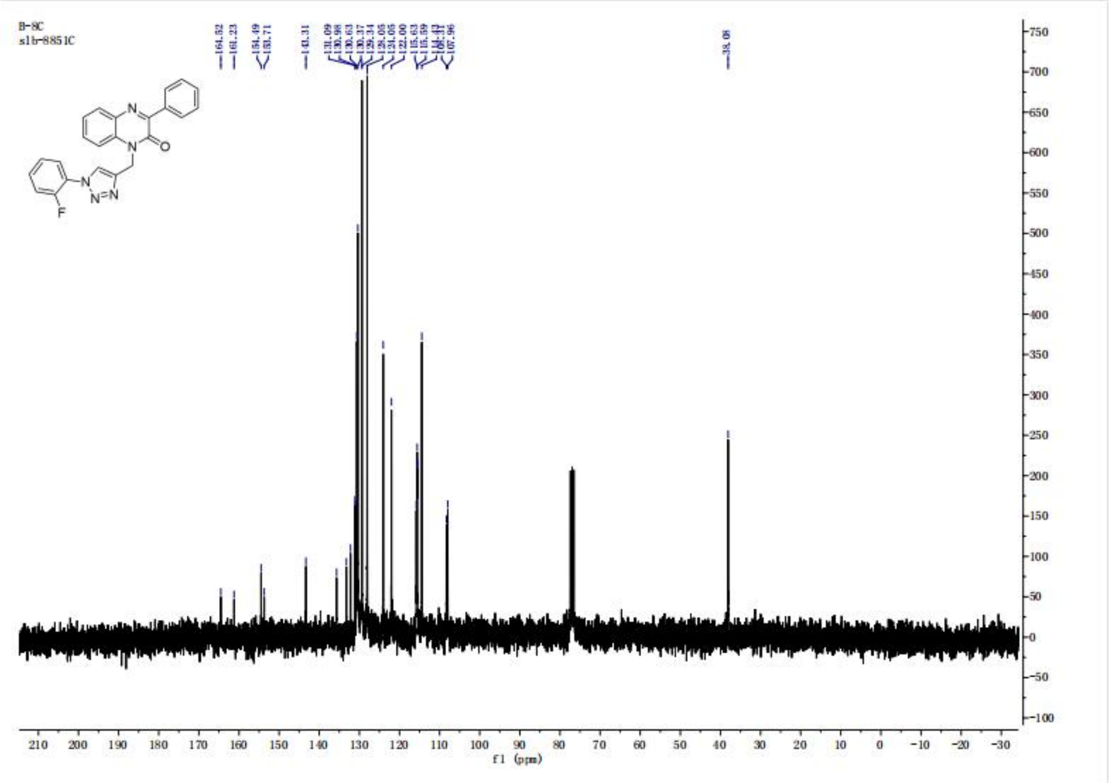

^{13}C NMR (75 MHz, $CDCl_3$) spectrum of compound **5b**

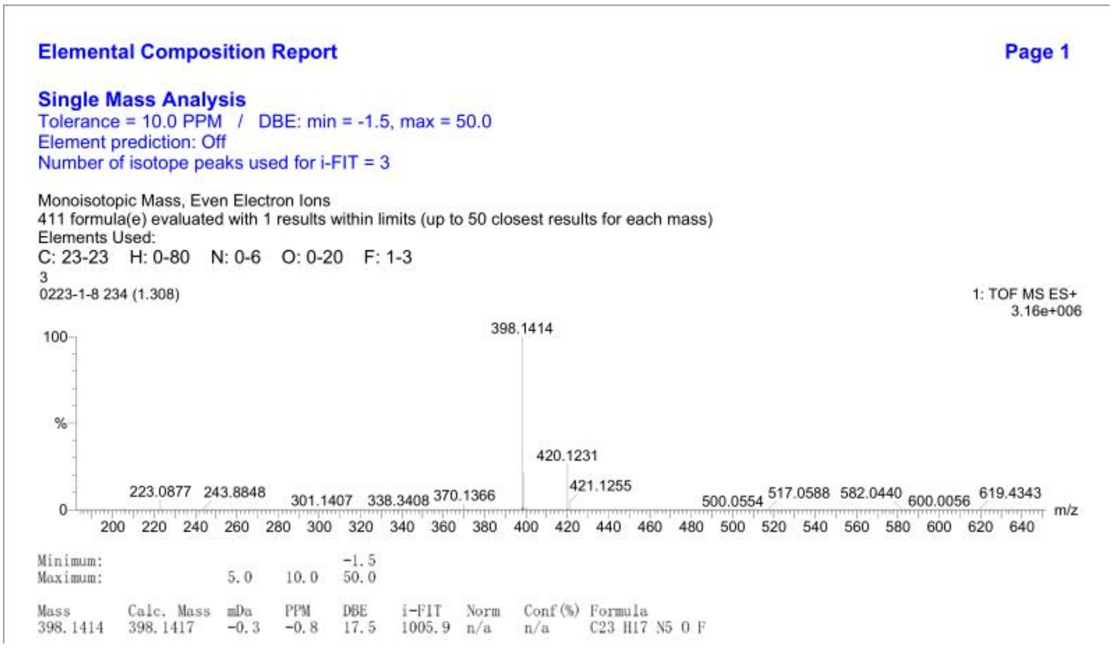
Elemental Composition Report Page 1

Single Mass Analysis
Tolerance = 10.0 PPM / DBE: min = -1.5, max = 50.0
Element prediction: Off
Number of isotope peaks used for i-FIT = 3

Monoisotopic Mass, Even Electron Ions
411 formula(e) evaluated with 1 results within limits (up to 50 closest results for each mass)
Elements Used:
C: 23-23 H: 0-80 N: 0-6 O: 0-20 F: 1-3
3
0223-1-8 234 (1.308) 1: TOF MS ES+ 3.16e+006

Minimum:				-1.5					
Maximum:		5.0	10.0	50.0					
Mass	Calc. Mass	mDa	PPM	DBE	i-FIT	Norm	Conf(%)	Formula	
398.1414	398.1417	-0.3	-0.8	17.5	1005.9	n/a	n/a	C23 H17 N5 O F	

HRMS spectrum of compound **5b**

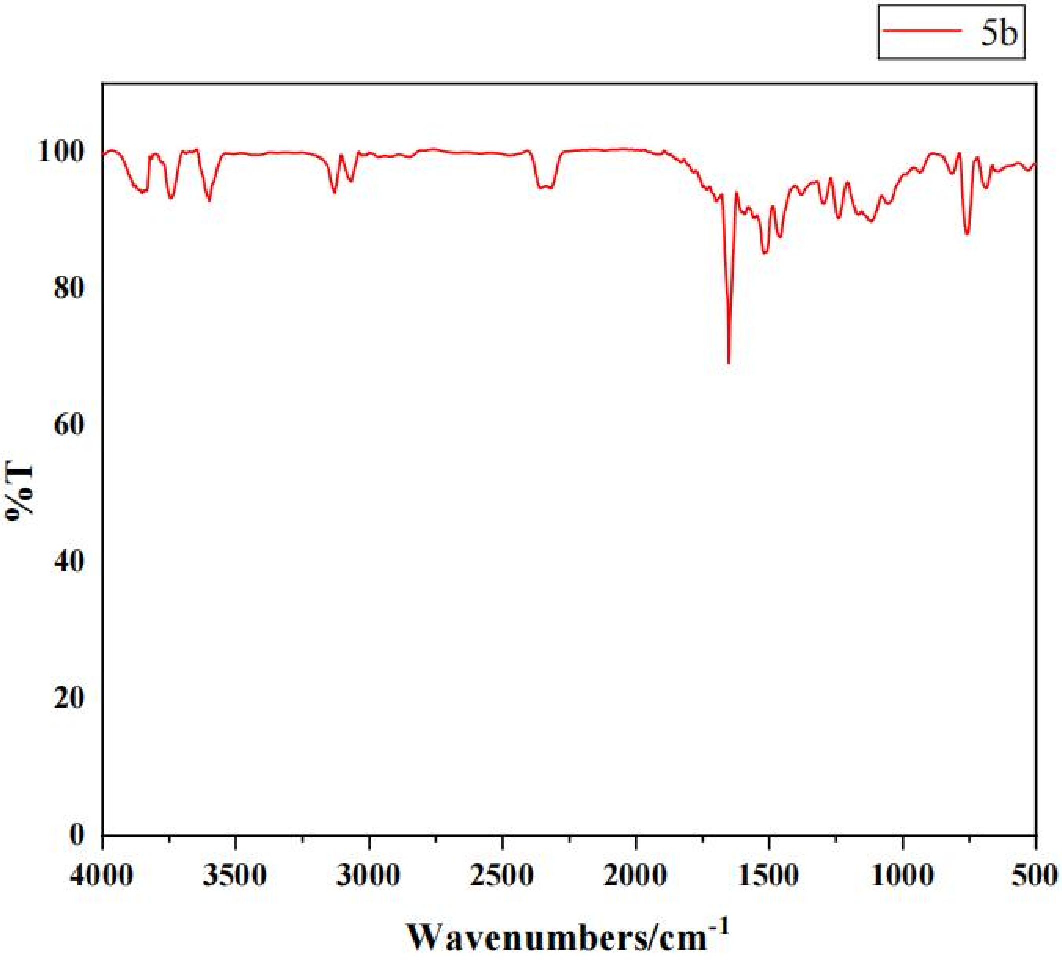

FT-IR spectrum of compound **5b**

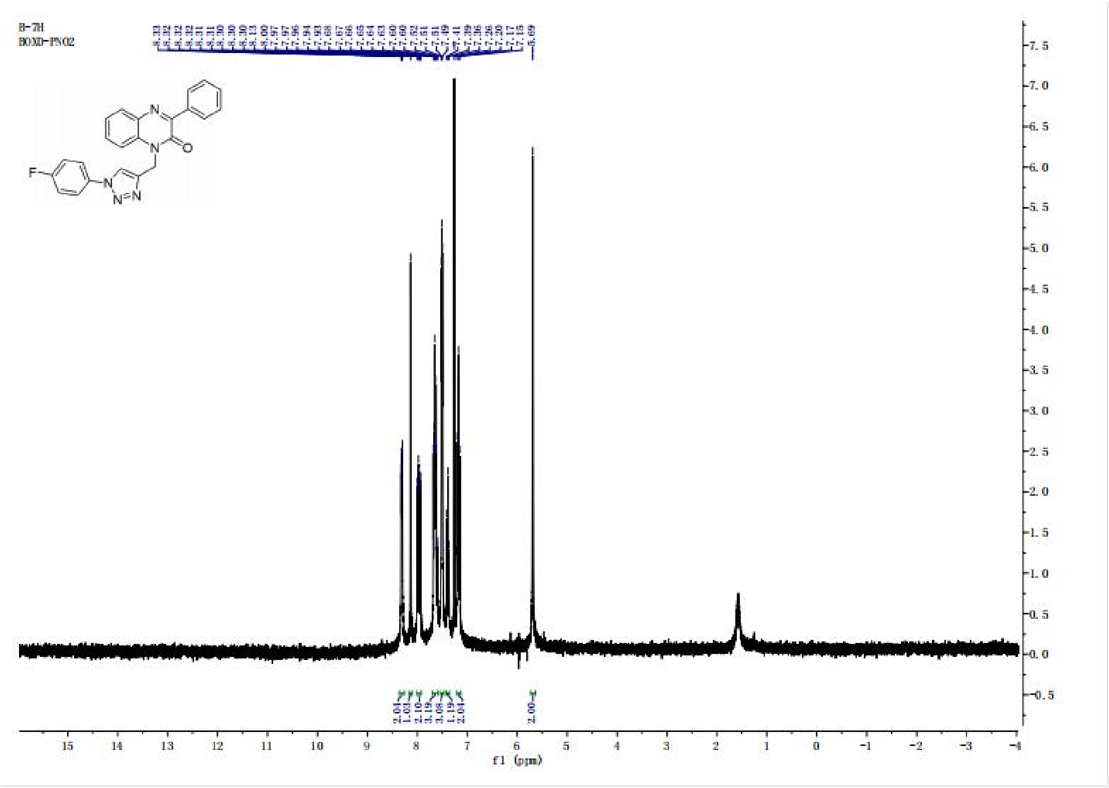

`

^{1}H-NMR (300 MHz, $CDCl_3$) spectrum of compound **5c**

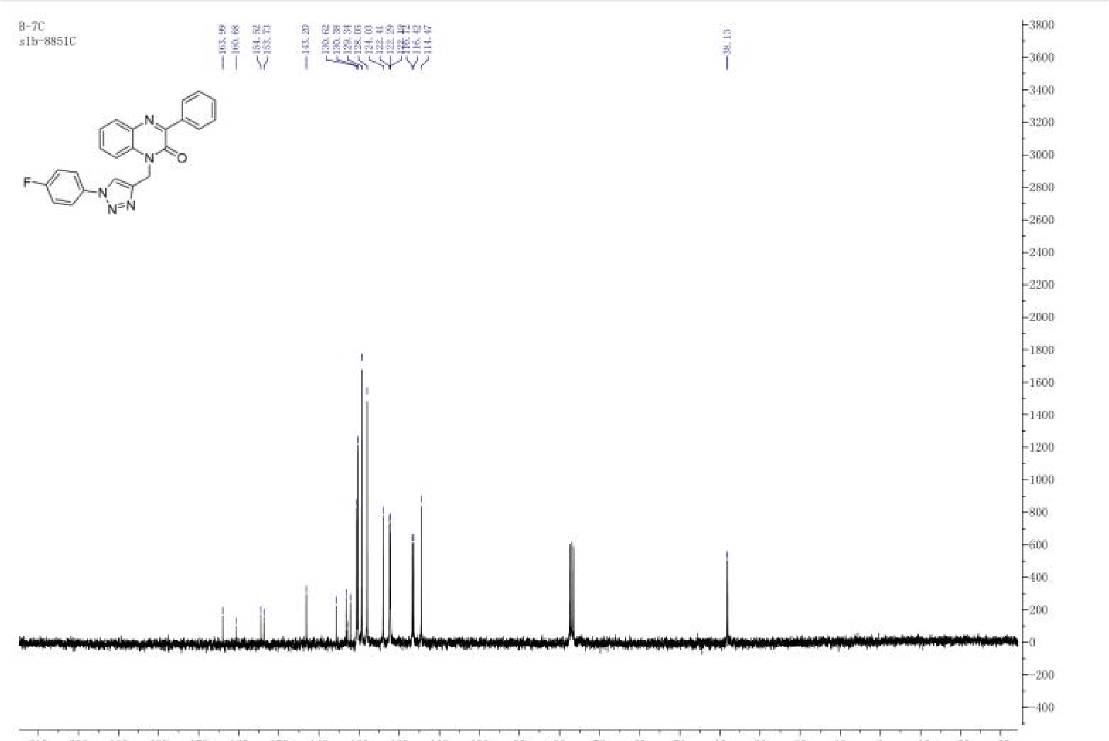

^{13}C NMR (75 MHz, $CDCl_3$) spectrum of compound **5c**

Elemental Composition Report Page 1

Single Mass Analysis
Tolerance = 10.0 PPM / DBE: min = -1.5, max = 50.0
Element prediction: Off
Number of isotope peaks used for i-FIT = 3

Monoisotopic Mass, Even Electron Ions
411 formula(e) evaluated with 1 results within limits (up to 50 closest results for each mass)
Elements Used:
C: 23-23 H: 0-80 N: 0-6 O: 0-20 F: 1-3

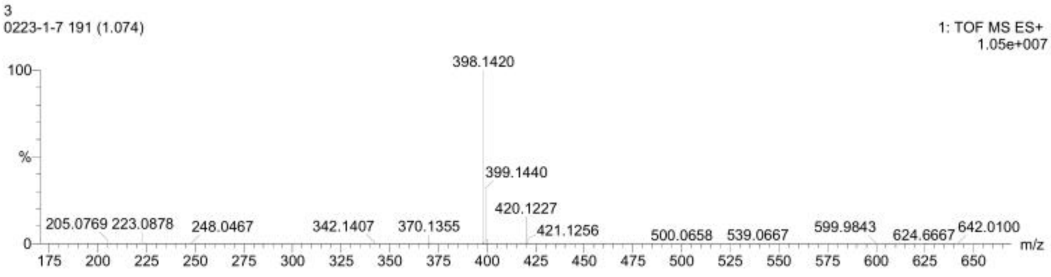

Minimum:				-1.5					
Maximum:		5.0	10.0	50.0					
Mass	Calc. Mass	mDa	PPM	DBE	i-FIT	Norm	Conf(%)	Formula	
398.1420	398.1417	0.3	0.8	17.5	1071.8	n/a	n/a	C23 H17 N5 O F	

HRMS spectrum of compound **5c**

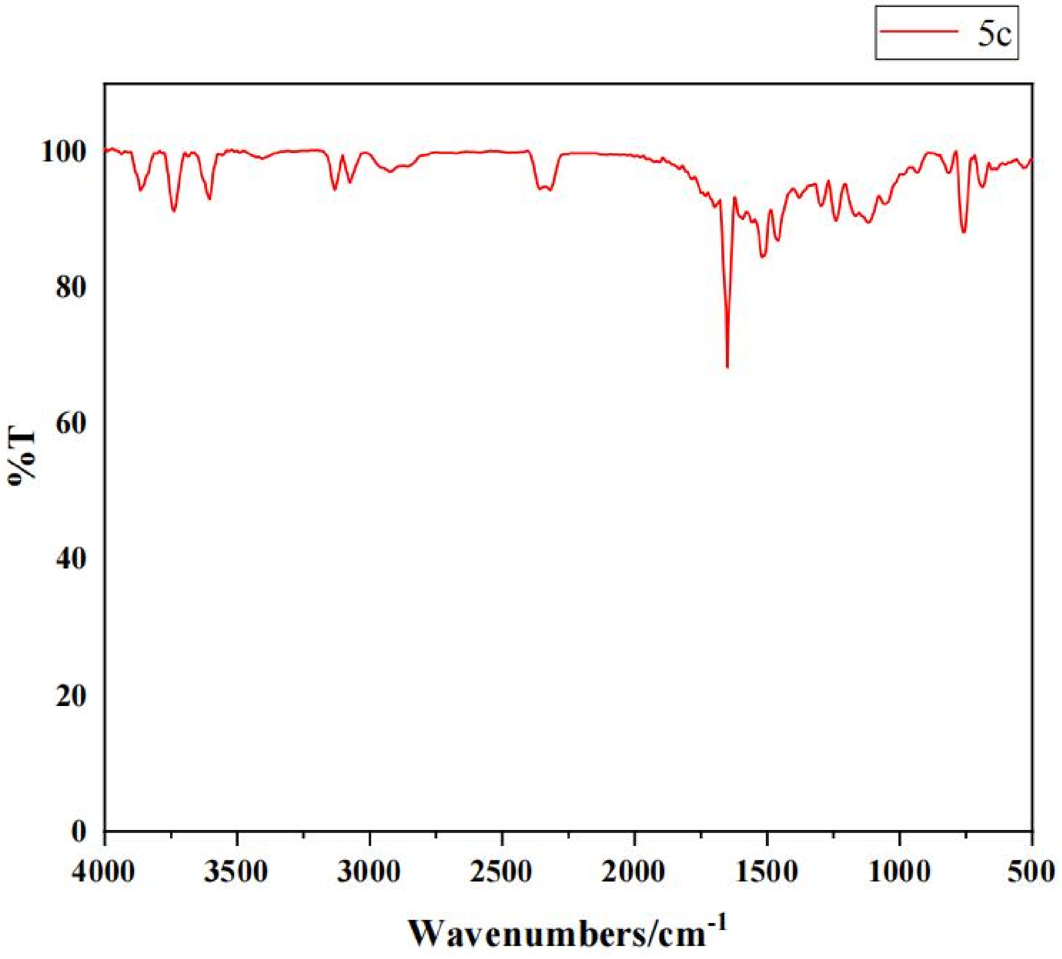

FT-IR spectrum of compound **5c**

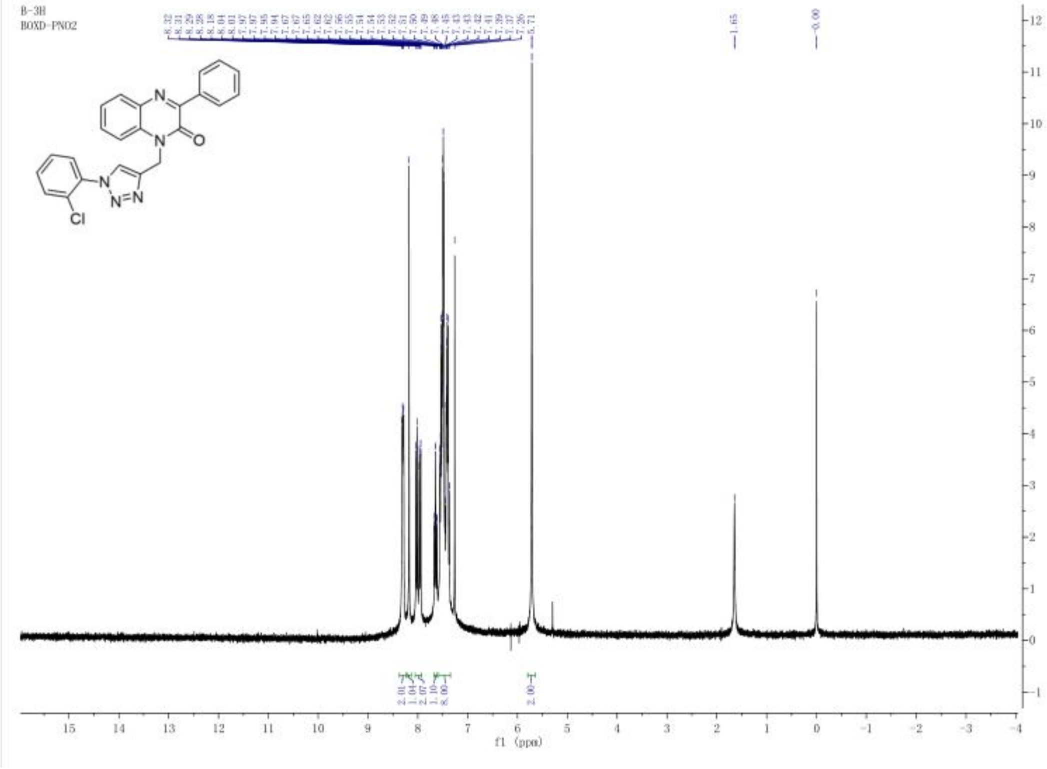

^{1}H-NMR (300 MHz, $CDCl_3$) spectrum of compound **5d**

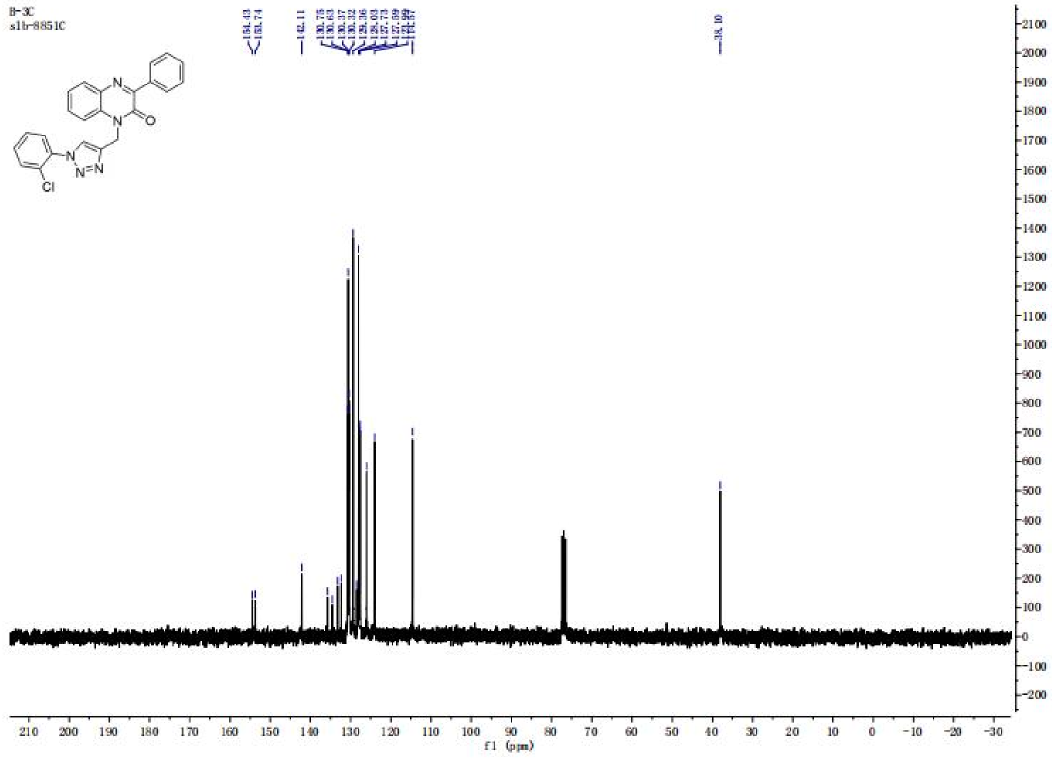

^{13}C NMR (75 MHz, $CDCl_3$) spectrum of compound **5d**

Elemental Composition Report **Page 1**

Single Mass Analysis
Tolerance = 10.0 PPM / DBE: min = -1.5, max = 50.0
Element prediction: Off
Number of isotope peaks used for i-FIT = 3

Monoisotopic Mass, Even Electron Ions
274 formula(e) evaluated with 1 results within limits (up to 50 closest results for each mass)
Elements Used:
C: 23-23 H: 0-80 N: 0-6 O: 0-20 Cl: 1-2
3
0223-1-3 247 (1.384)

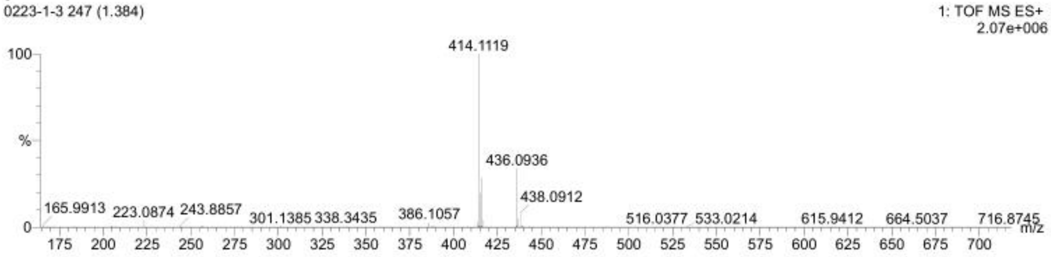

Minimum:				-1.5				
Maximum:		5.0	10.0	50.0				
Mass	Calc. Mass	mDa	PPM	DBE	i-FIT	Norm	Conf(%)	Formula
414.1119	414.1122	-0.3	-0.7	17.5	1009.9	n/a	n/a	C23 H17 N5 O Cl

HRMS spectrum of compound **5d**

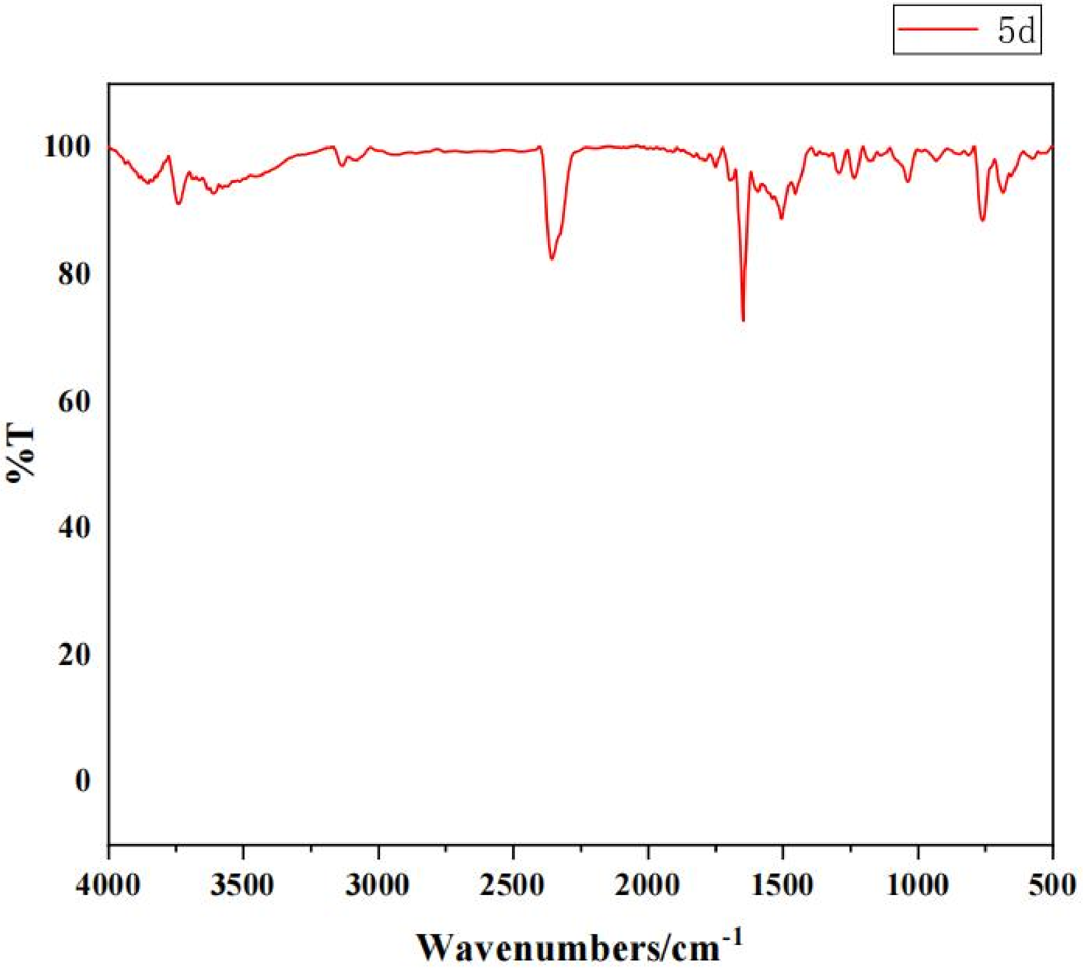

FT-IR spectrum of compound **5d**

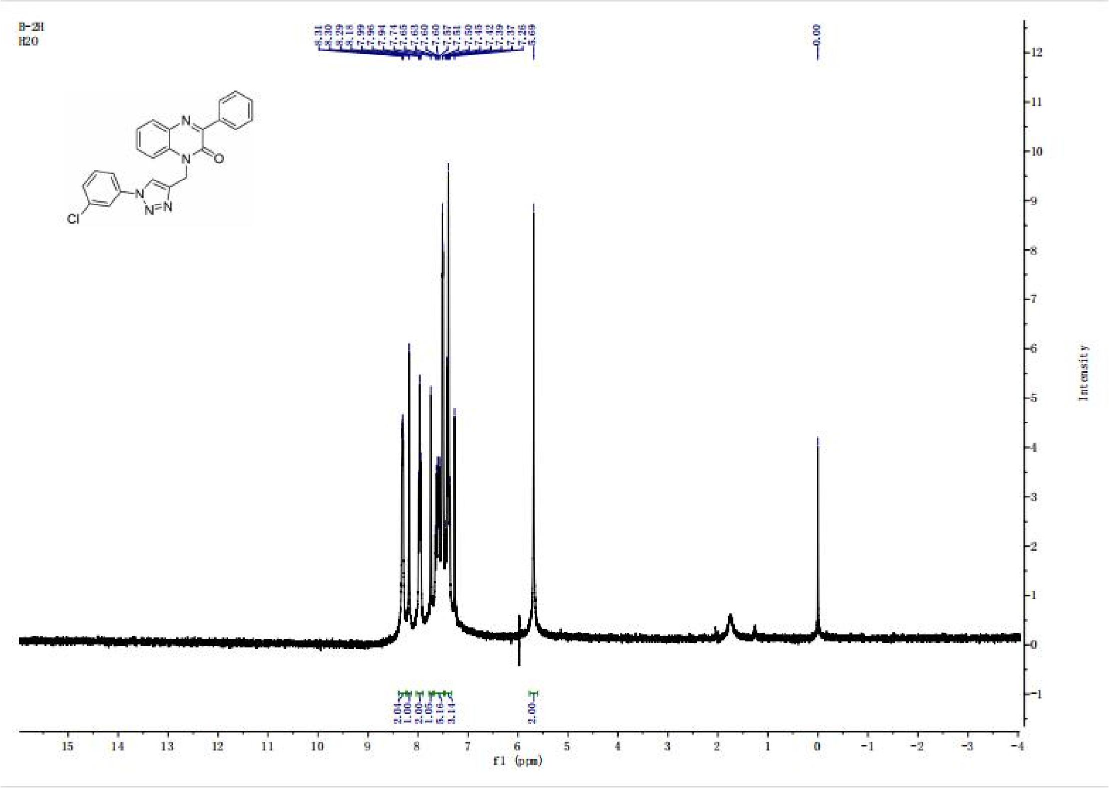

`

^{1}H-NMR (300 MHz, $CDCl_3$) spectrum of compound **5e**

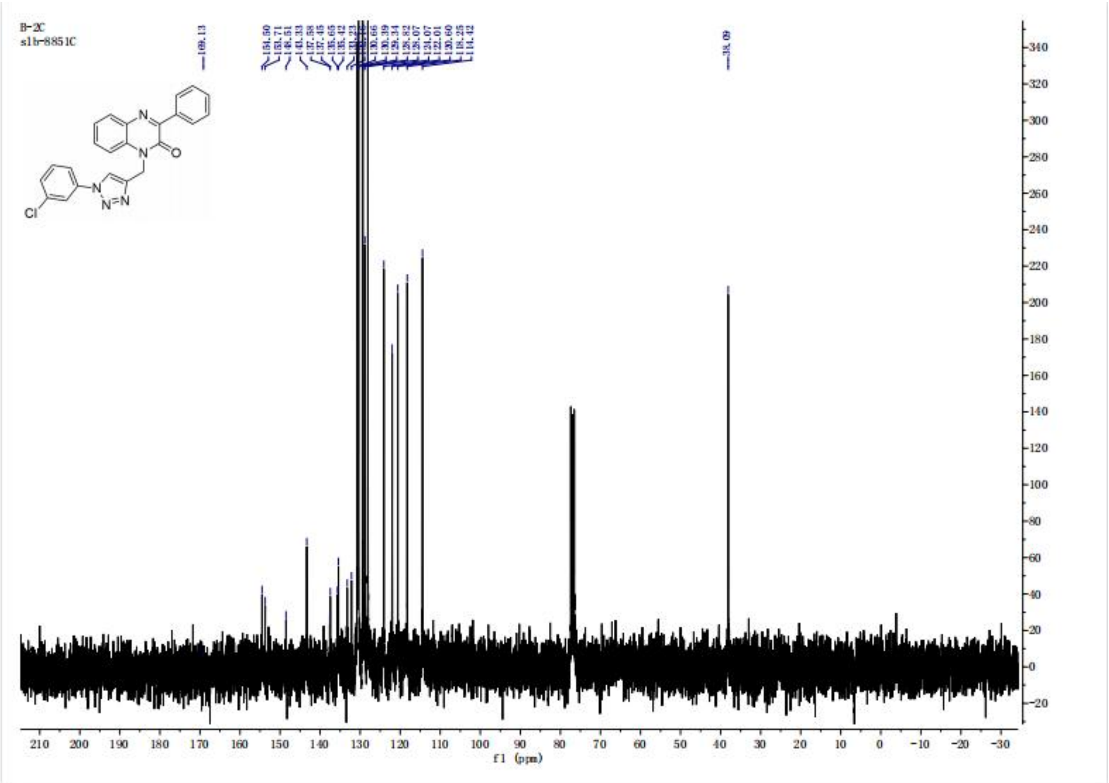

^{13}C NMR (75 MHz, $CDCl_3$) spectrum of compound **5e**

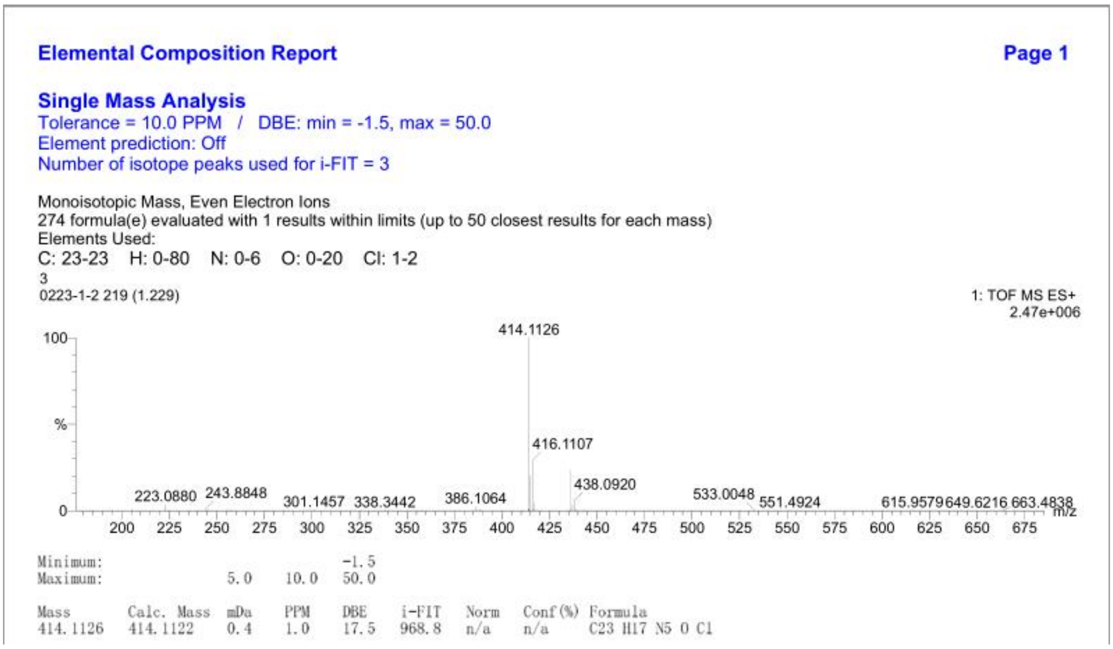

HRMS spectrum of compound **5e**

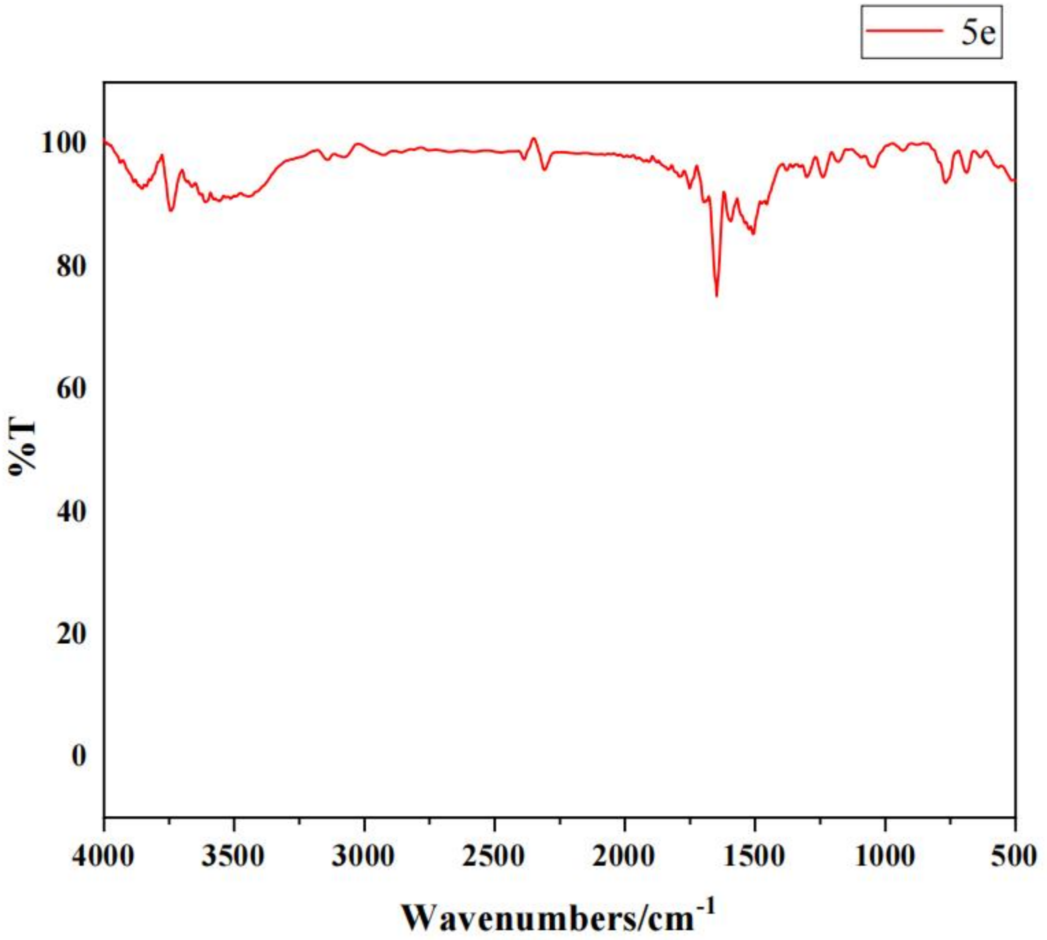

FT-IR spectrum of compound **5e**

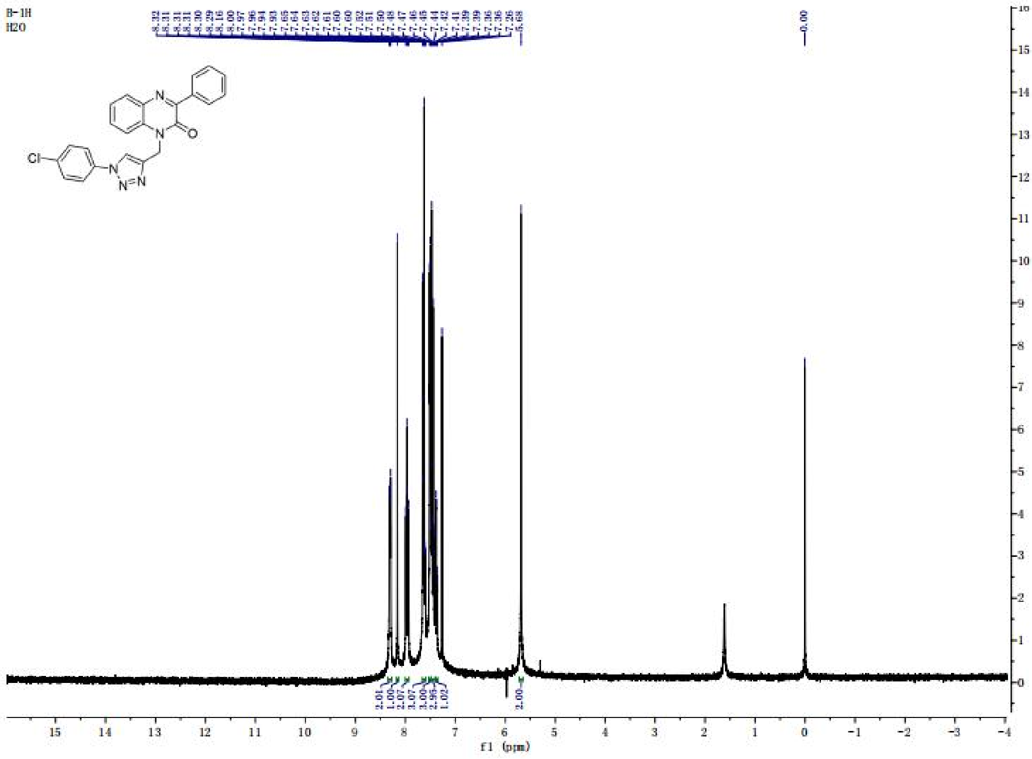

^{1}H-NMR (300 MHz, $CDCl_3$) spectrum of compound **5f**

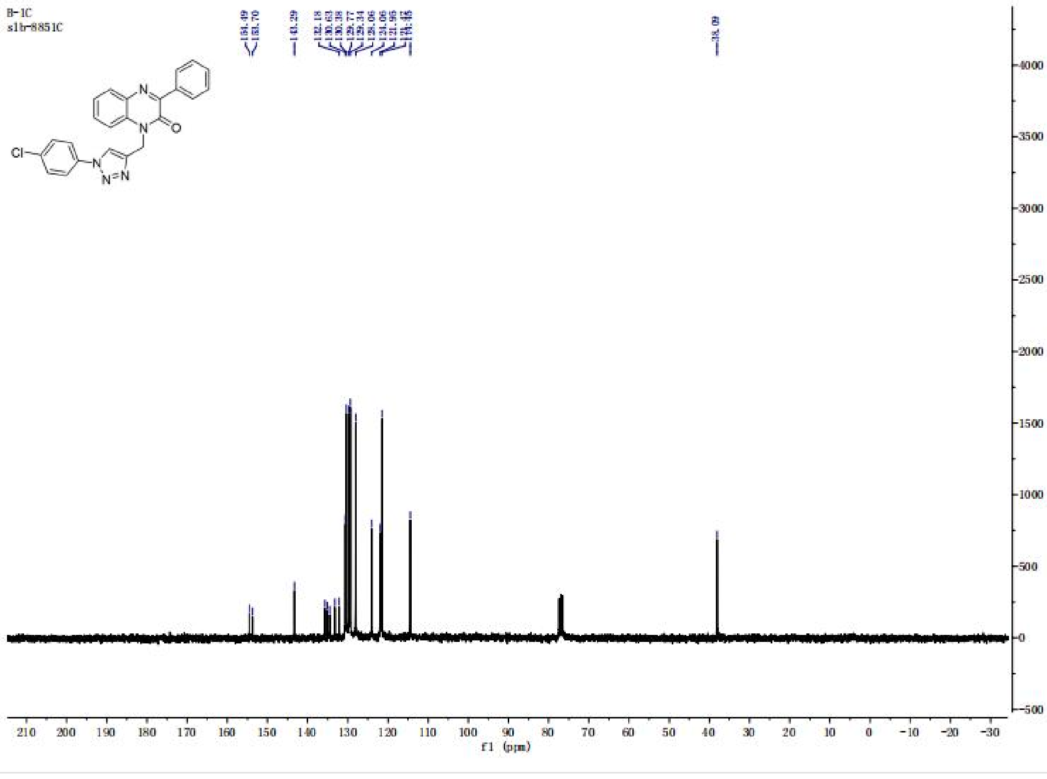

^{13}C NMR (75 MHz, $CDCl_3$) spectrum of compound **5f**

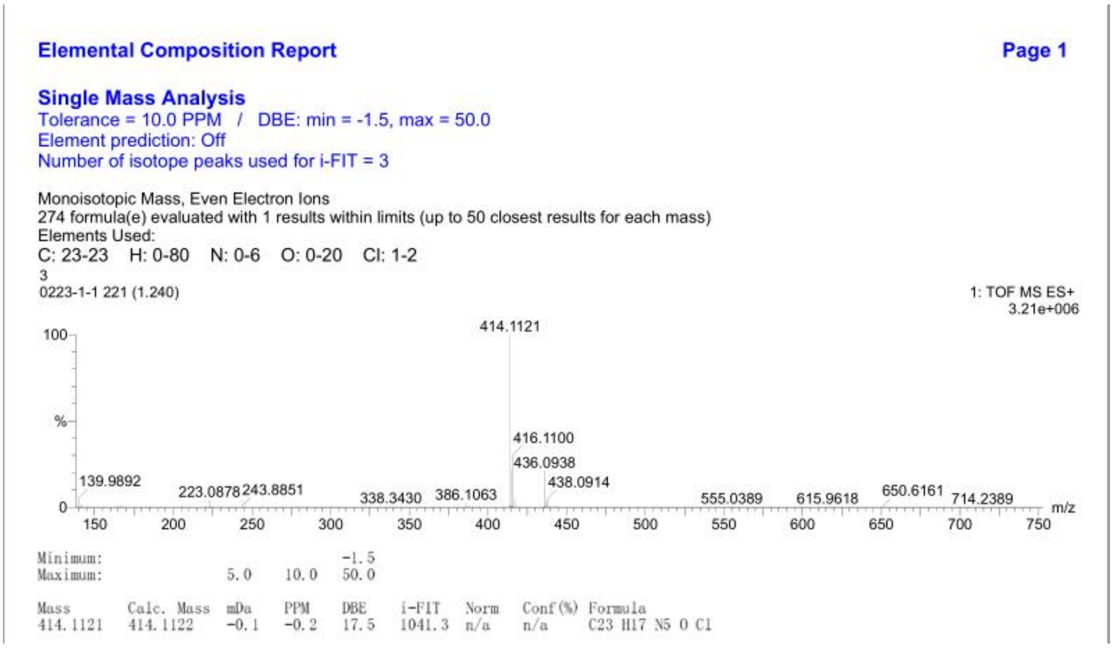

HRMS spectrum of compound **5f**

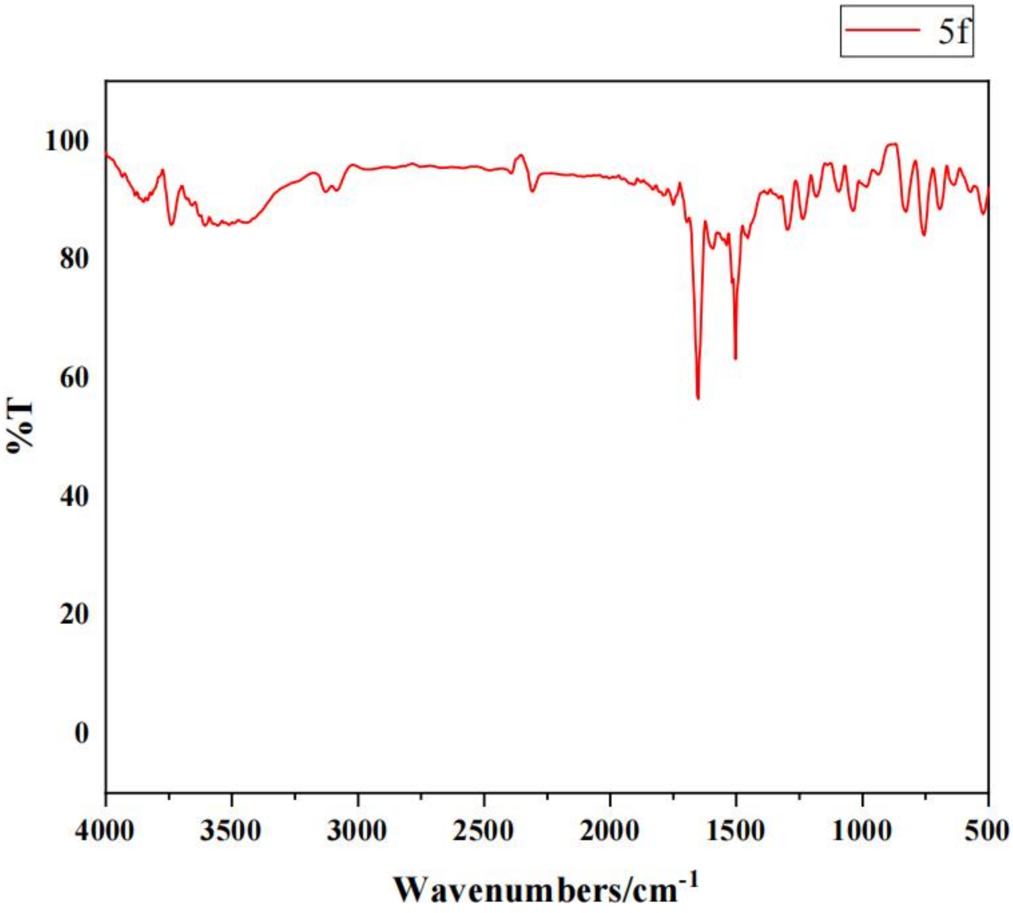

FT-IR spectrum of compound **5f**

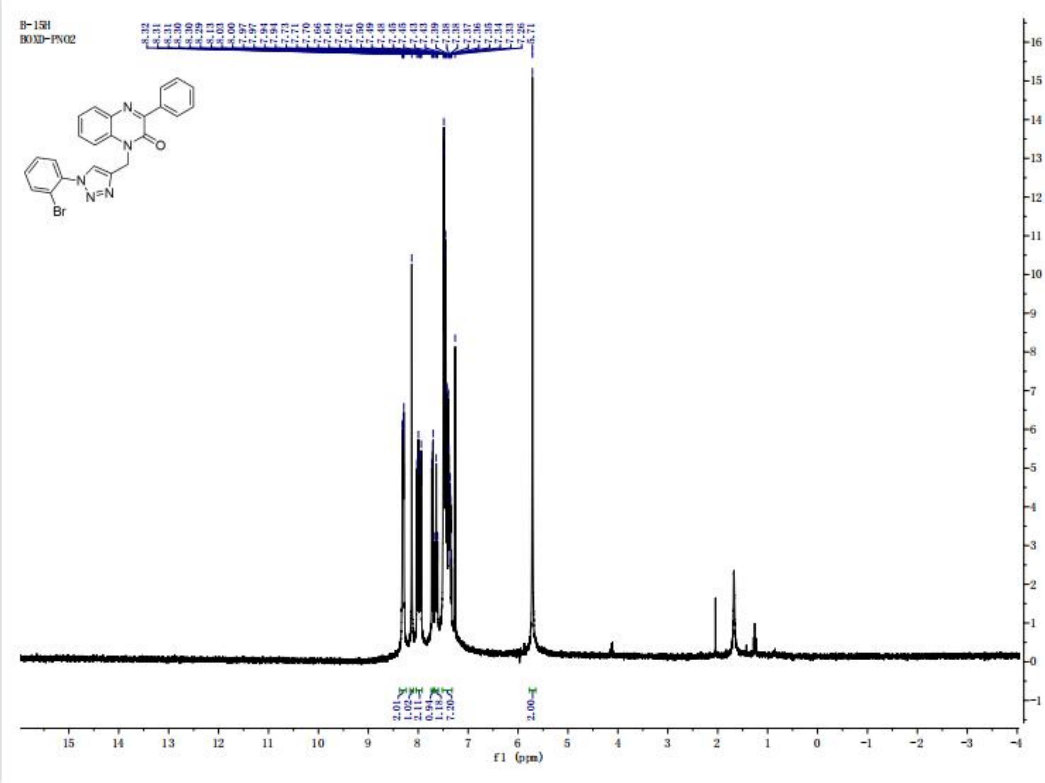

^{1}H-NMR (300 MHz, $CDCl_3$) spectrum of compound **5g**

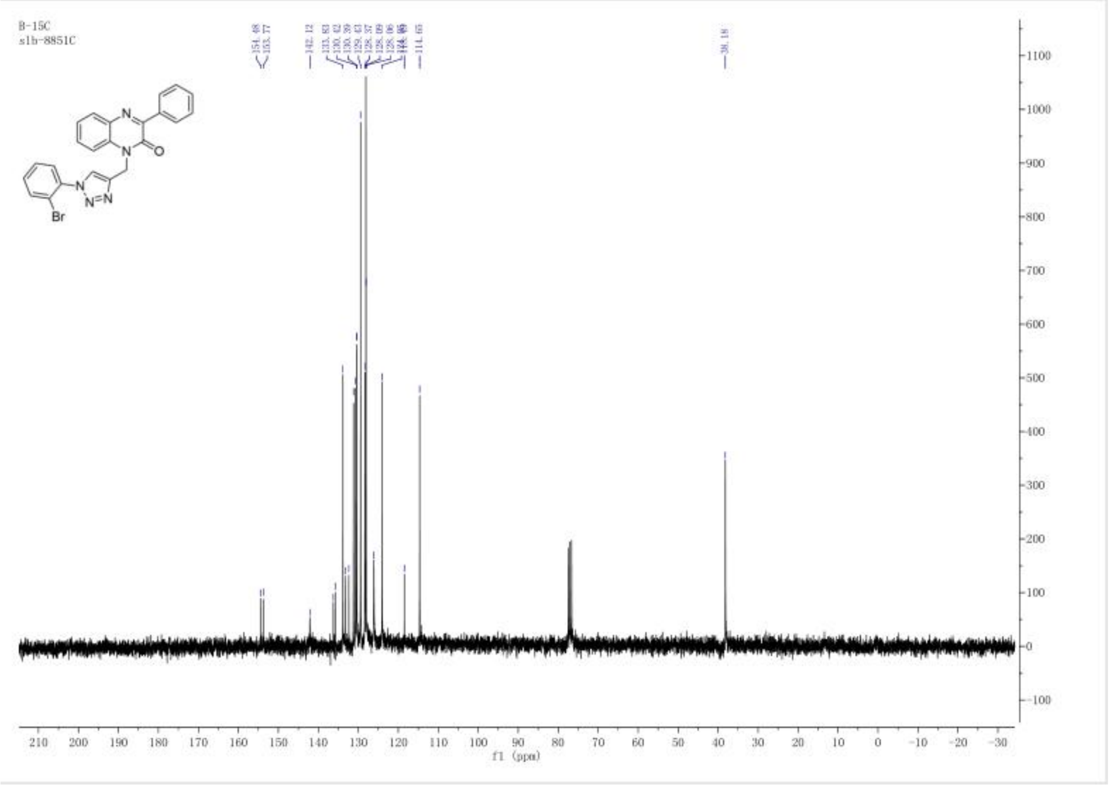

^{13}C NMR (75 MHz, $CDCl_3$) spectrum of compound **5g**

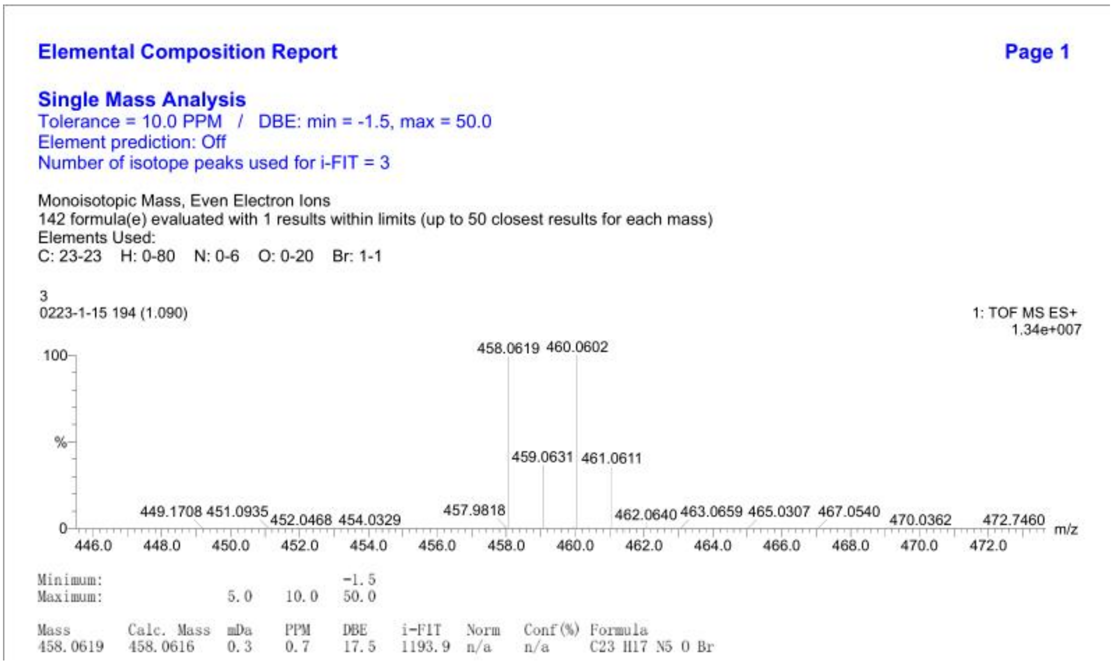

HRMS spectrum of compound **5g**

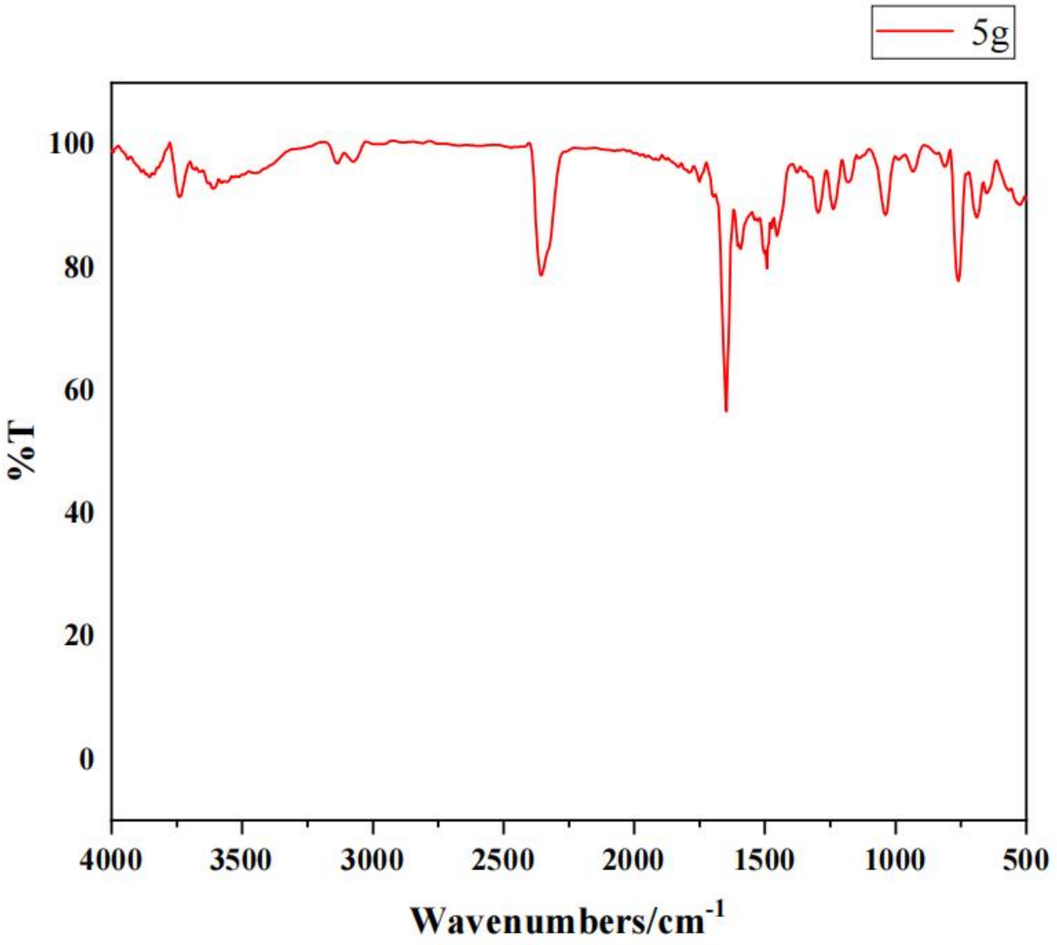

FT-IR spectrum of compound **5g**

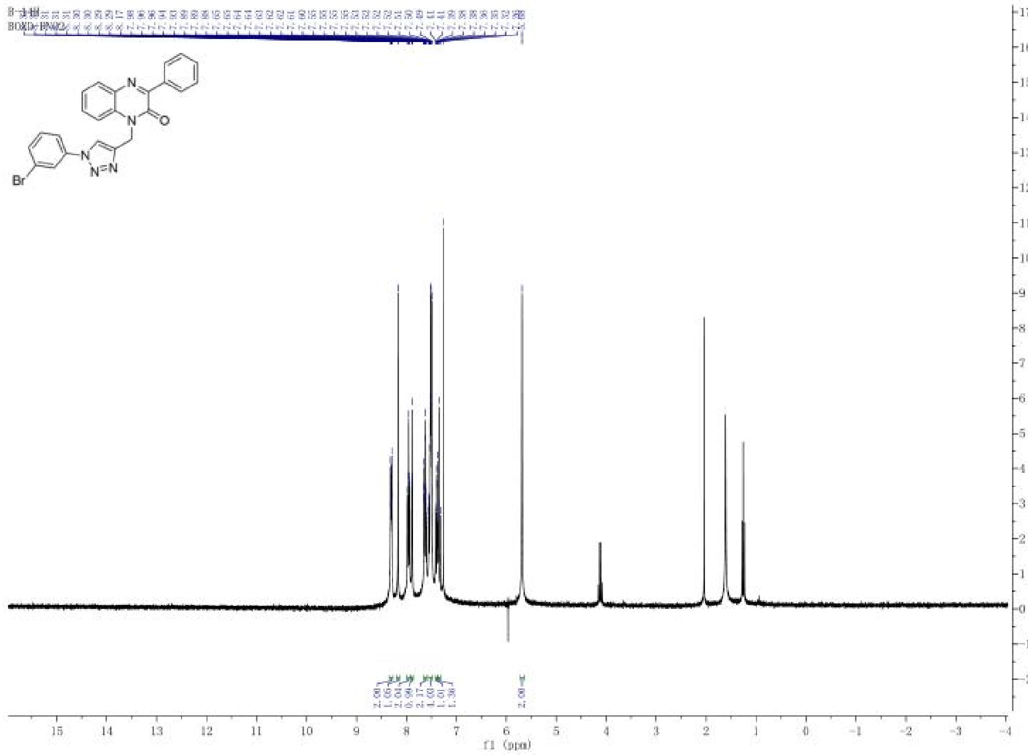

^{1}H-NMR (300 MHz, $CDCl_3$) spectrum of compound **5h**

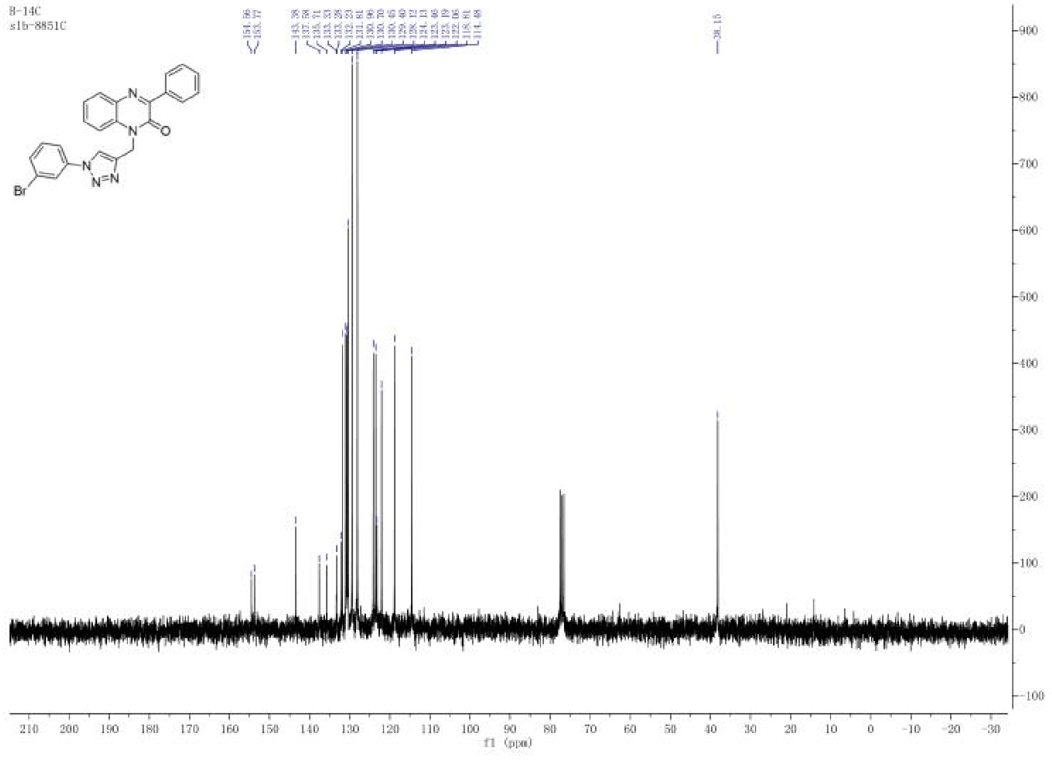

^{13}C NMR (75 MHz, $CDCl_3$) spectrum of compound **5h**

Elemental Composition Report

Single Mass Analysis
Tolerance = 10.0 PPM / DBE: min = -1.5, max = 50.0
Element prediction: Off
Number of isotope peaks used for i-FIT = 3

Monoisotopic Mass, Even Electron Ions
142 formula(e) evaluated with 1 results within limits (up to 50 closest results for each mass)
Elements Used:
C: 23-23 H: 0-80 N: 0-6 O: 0-20 Br: 1-1

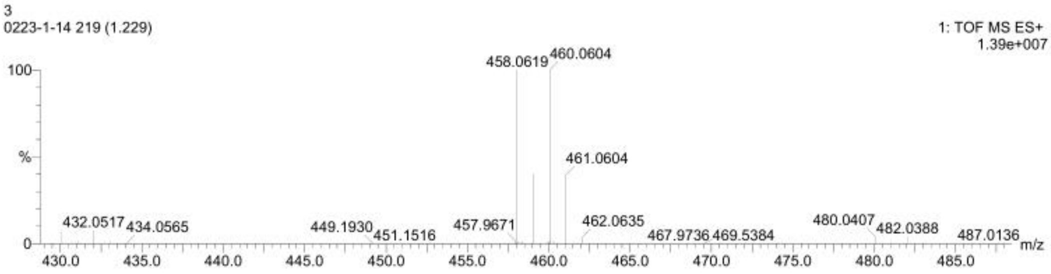

Minimum:				-1.5					
Maximum:		5.0	10.0	50.0					

Mass	Calc. Mass	mDa	PPM	DBE	i-FIT	Norm	Conf(%)	Formula
458.0619	458.0616	0.3	0.7	17.5	1161.6	n/a	n/a	C23 H17 N5 O Br

HRMS spectrum of compound **5h**

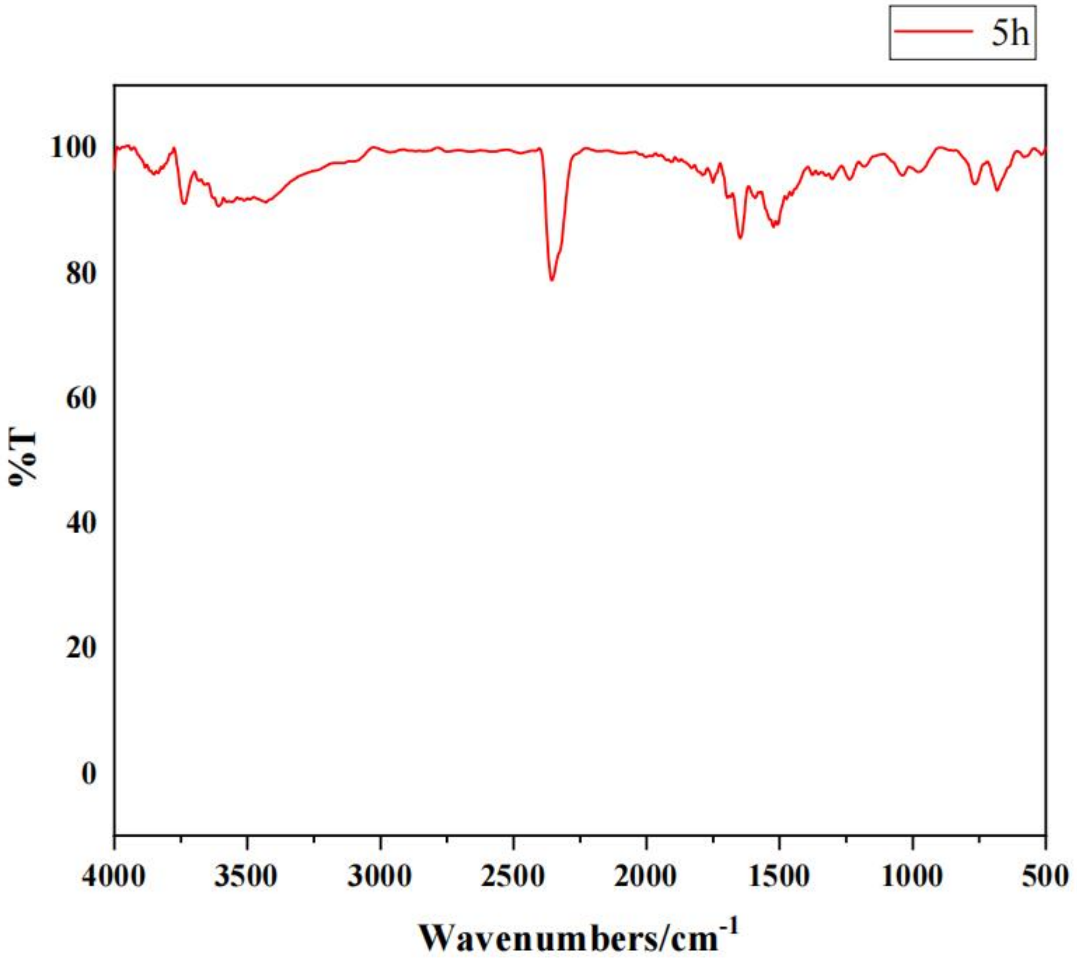

FT-IR spectrum of compound **5h**

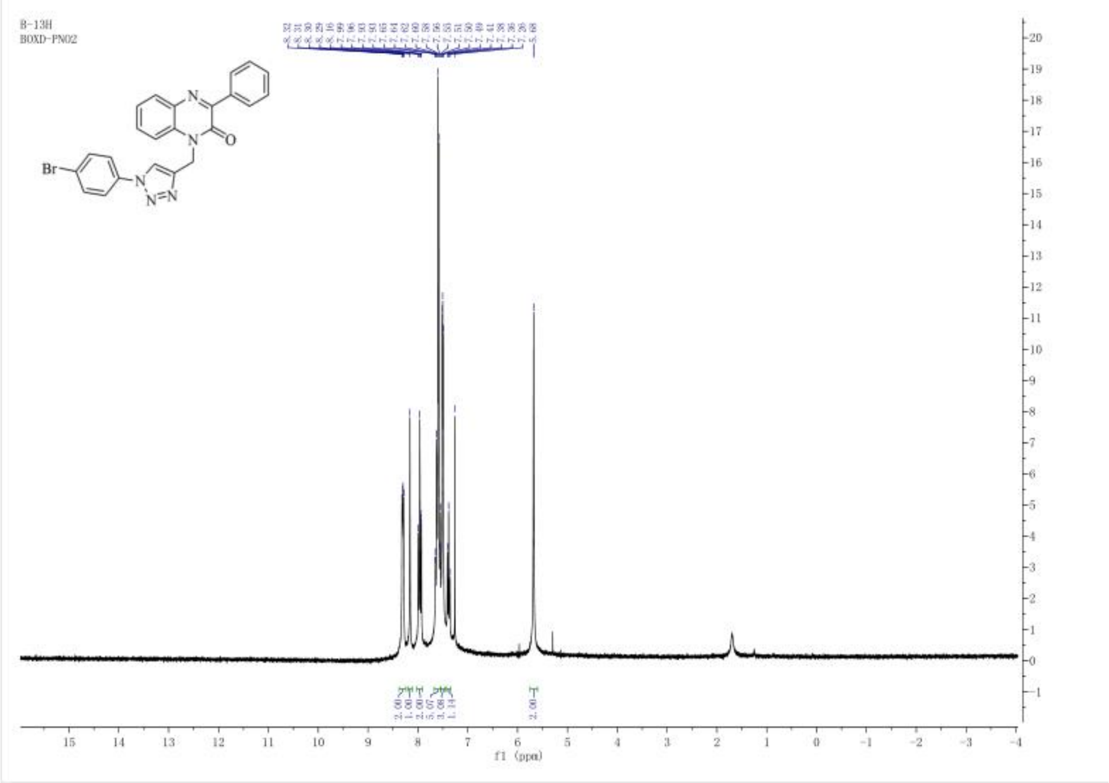

^{1}H-NMR (300 MHz, $CDCl_3$) spectrum of compound **5i**

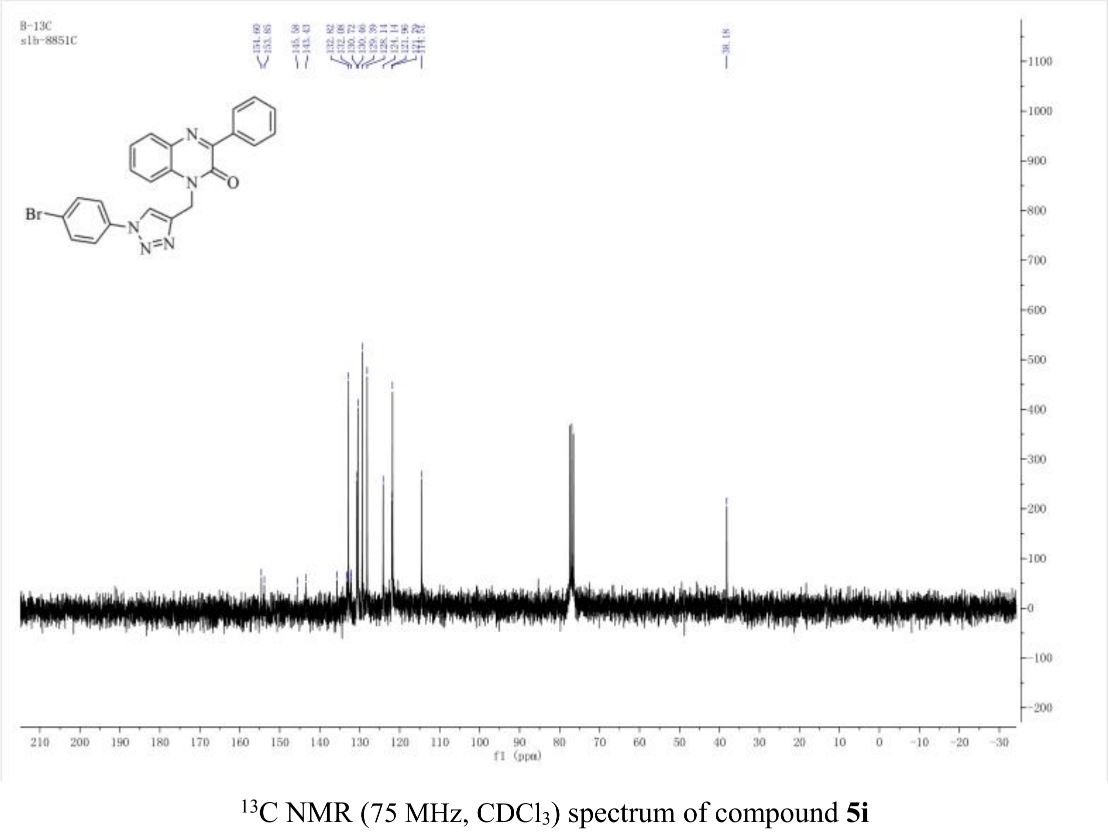

^{13}C NMR (75 MHz, $CDCl_3$) spectrum of compound **5i**

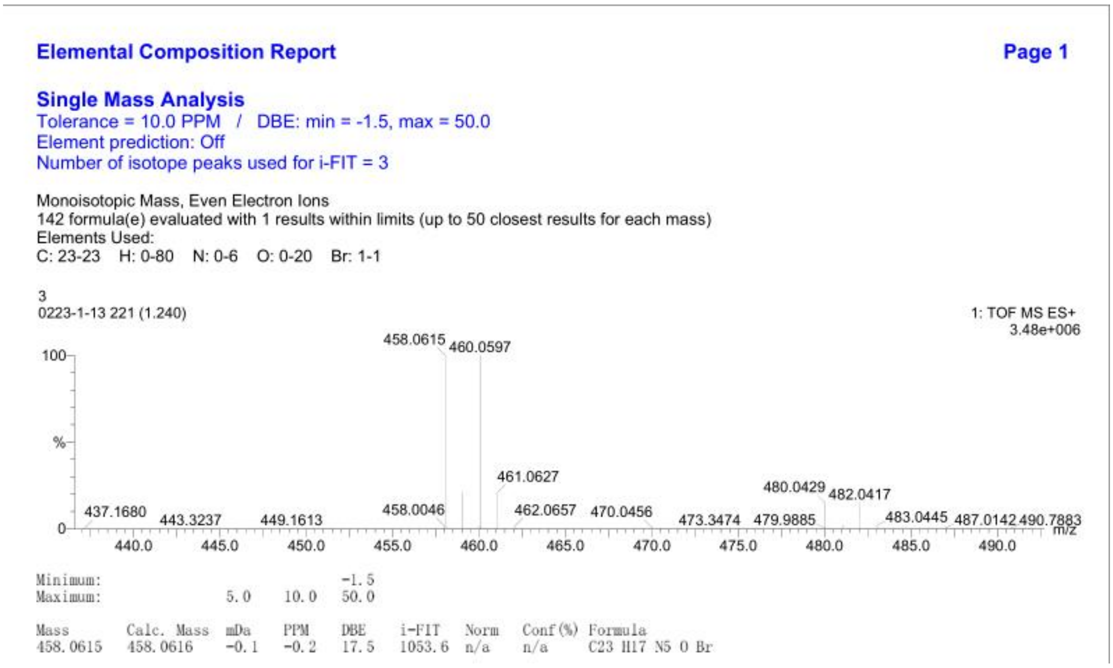

HRMS spectrum of compound **5i**

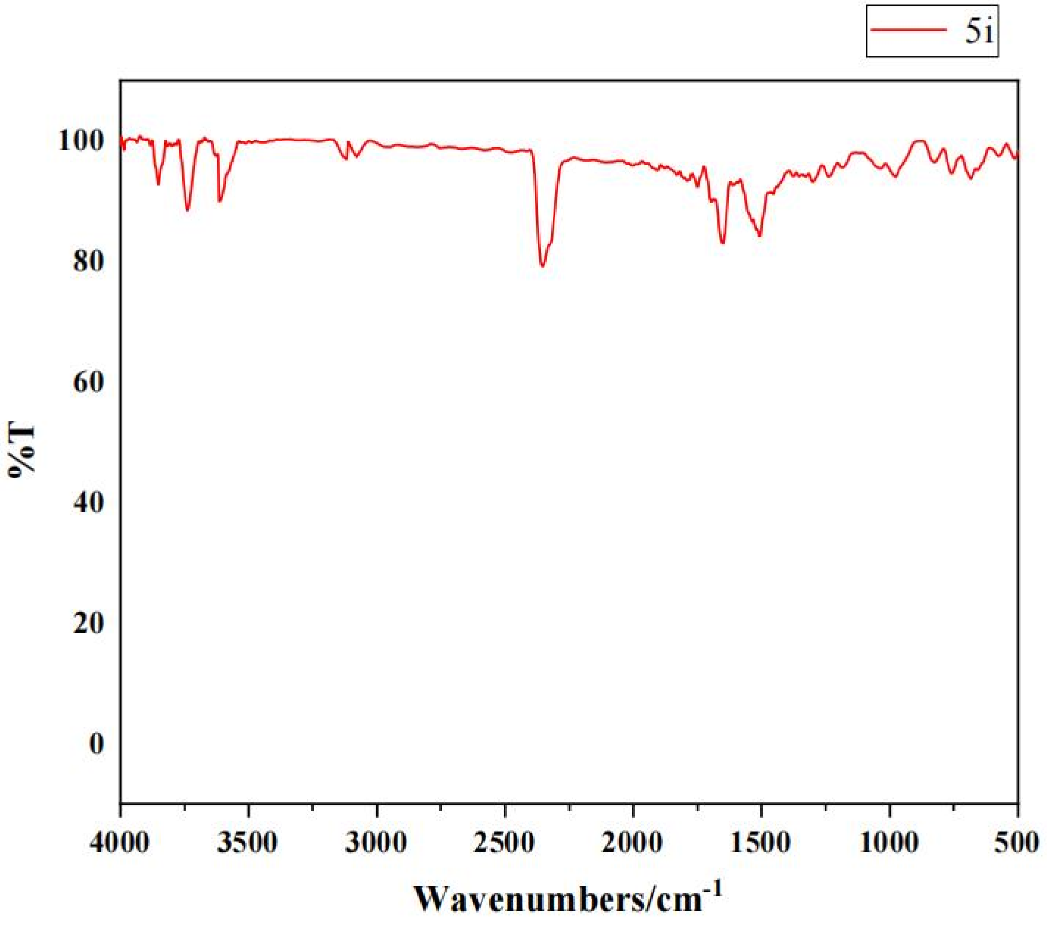

FT-IR spectrum of compound **5i**

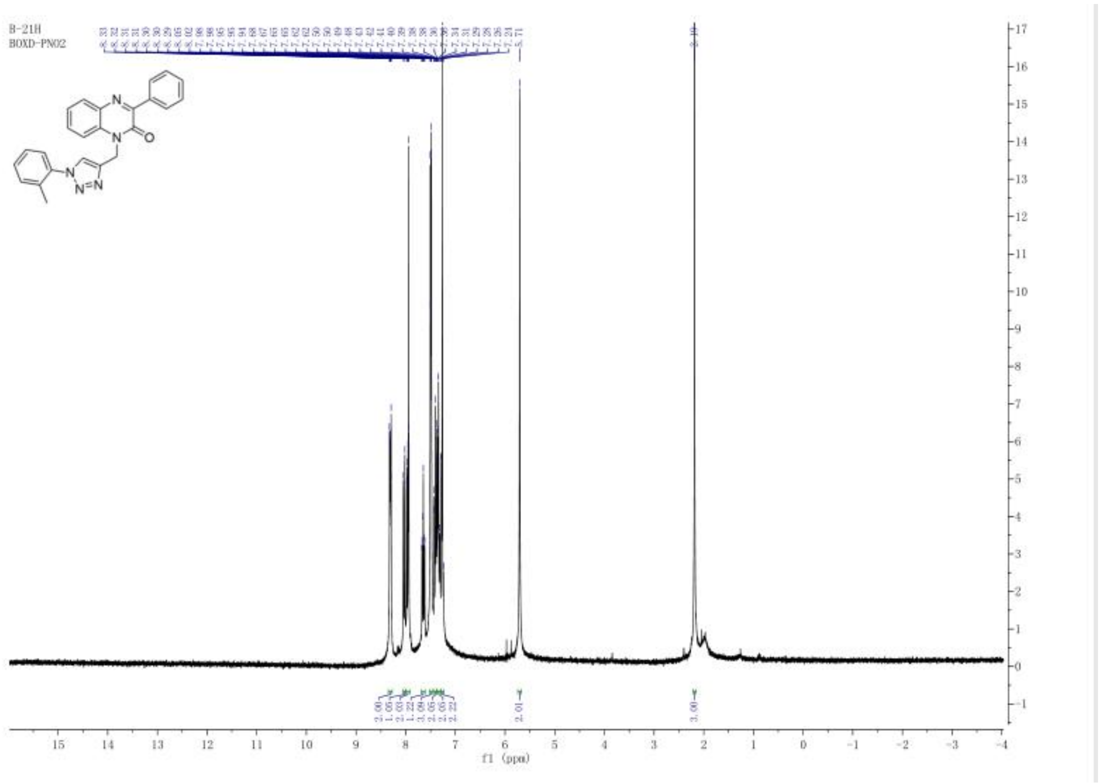

^{1}H-NMR (300 MHz, $CDCl_3$) spectrum of compound **5j**

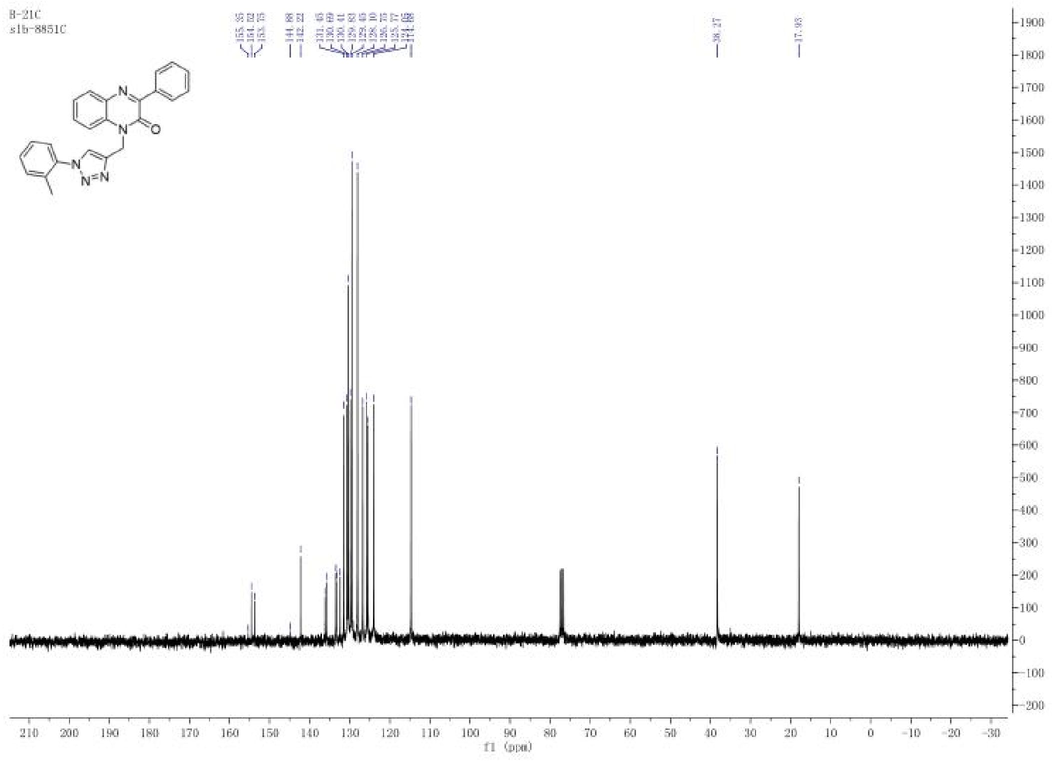

^{13}C NMR (75 MHz, $CDCl_3$) spectrum of compound **5j**

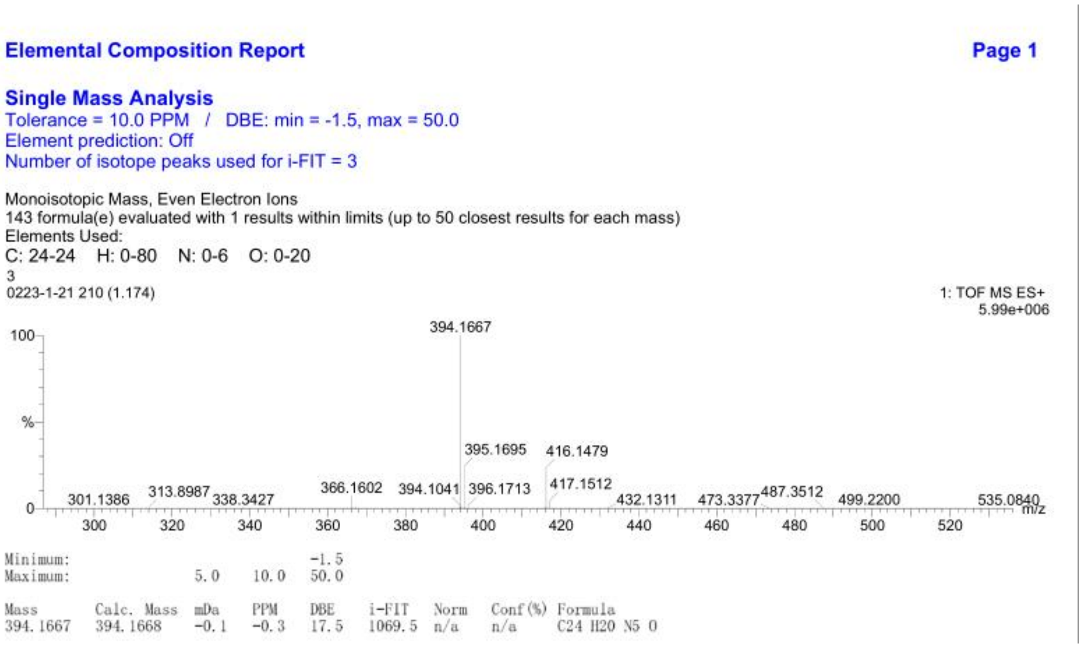
Elemental Composition Report Page 1

Single Mass Analysis
Tolerance = 10.0 PPM / DBE: min = -1.5, max = 50.0
Element prediction: Off
Number of isotope peaks used for i-FIT = 3

Monoisotopic Mass, Even Electron Ions
143 formula(e) evaluated with 1 results within limits (up to 50 closest results for each mass)
Elements Used:
C: 24-24 H: 0-80 N: 0-6 O: 0-20
3
0223-1-21 210 (1.174) 1: TOF MS ES+ 5.99e+006

Minimum:				-1.5				
Maximum:		5.0	10.0	50.0				
Mass	Calc. Mass	mDa	PPM	DBE	i-FIT	Norm	Conf(%)	Formula
394.1667	394.1668	-0.1	-0.3	17.5	1069.5	n/a	n/a	C24 H20 N5 O

HRMS spectrum of compound **5j**

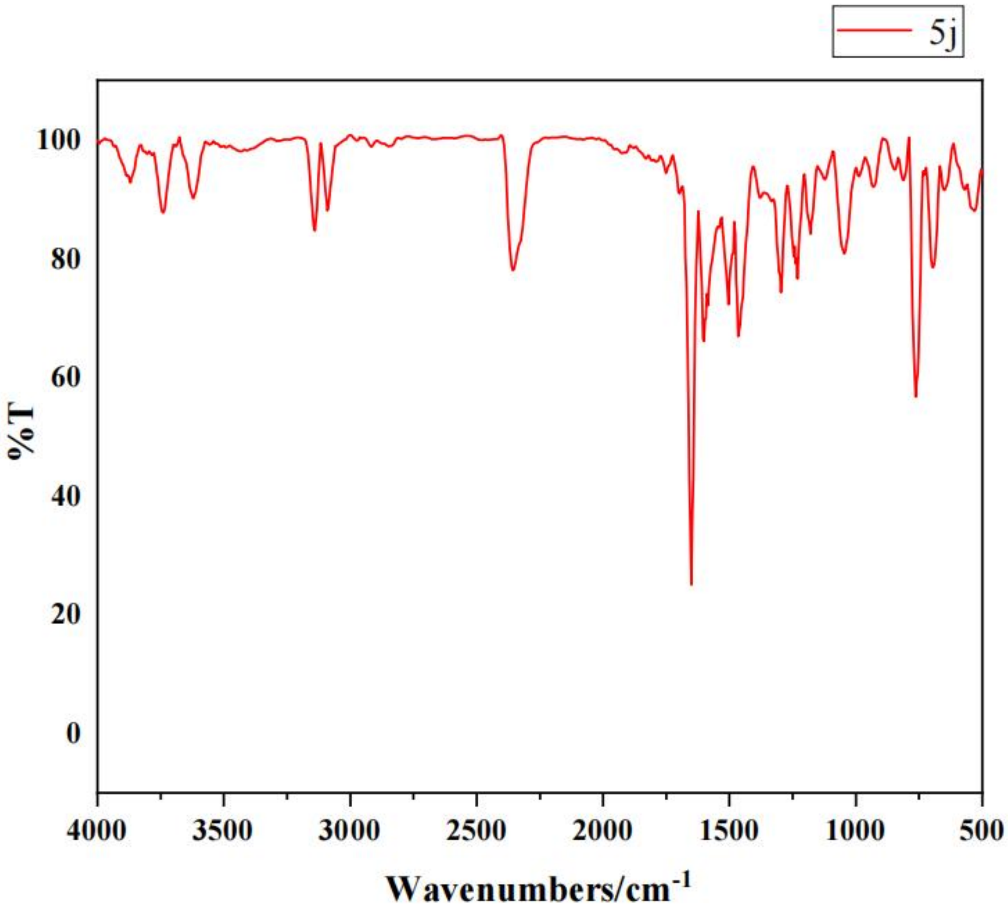

FT-IR spectrum of compound **5j**

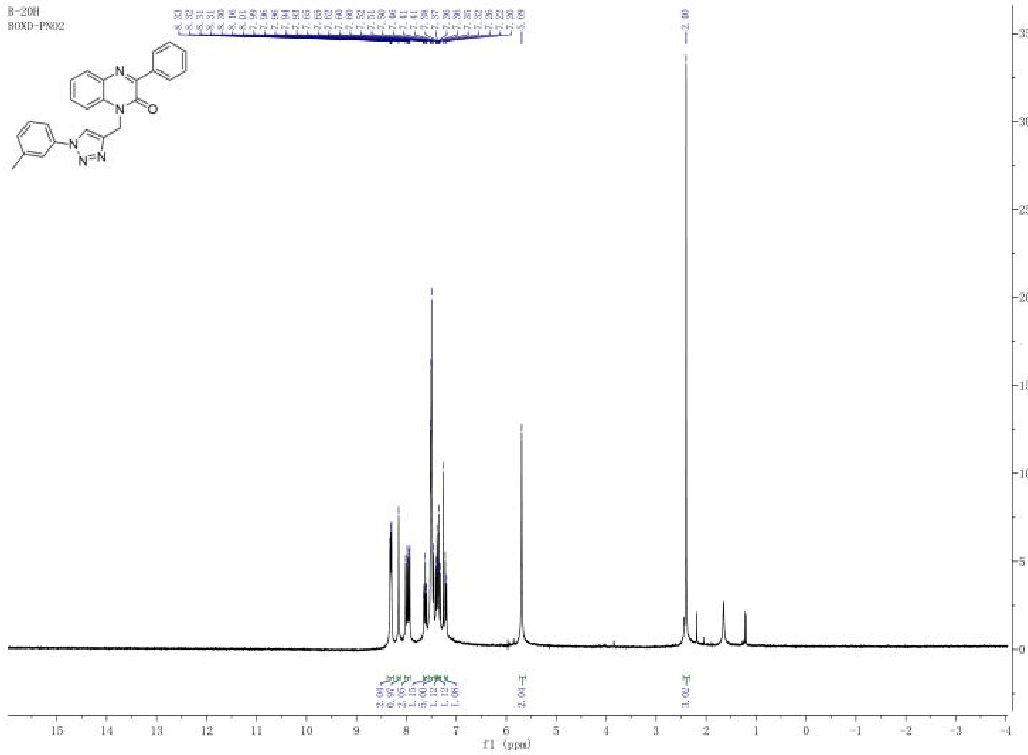

^{1}H-NMR (300 MHz, $CDCl_3$) spectrum of compound **5k**

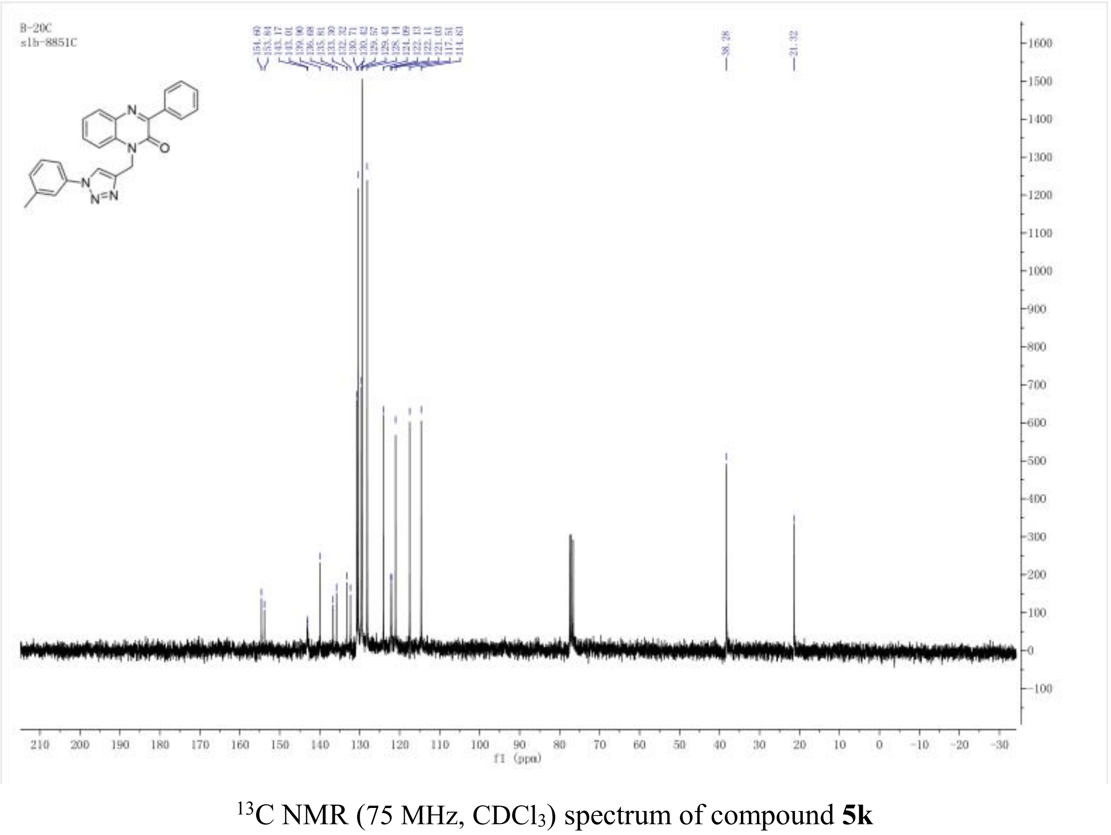

^{13}C NMR (75 MHz, $CDCl_3$) spectrum of compound **5k**

Elemental Composition Report **Page 1**

Single Mass Analysis
Tolerance = 10.0 PPM / DBE: min = -1.5, max = 50.0
Element prediction: Off
Number of isotope peaks used for i-FIT = 3

Monoisotopic Mass, Even Electron Ions
143 formula(e) evaluated with 1 results within limits (up to 50 closest results for each mass)
Elements Used:
C: 24-24 H: 0-80 N: 0-6 O: 0-20
3
0223-1-20 217 (1.219) 1: TOF MS ES+ 1.13e+007

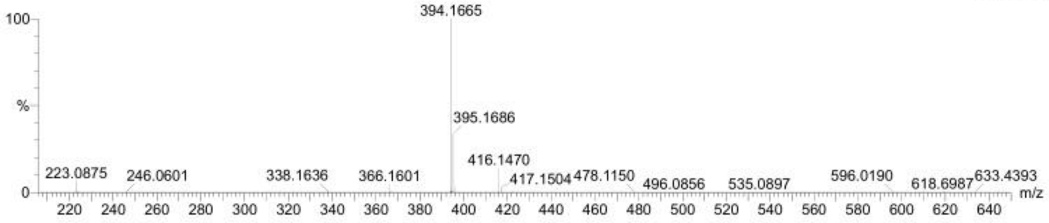

Minimum:				-1.5				
Maximum:		5.0	10.0	50.0				

Mass	Calc. Mass	mDa	PPM	DBE	i-FIT	Norm	Conf(%)	Formula
394.1665	394.1668	-0.3	-0.8	17.5	1143.3	n/a	n/a	C24 H20 N5 O

HRMS spectrum of compound **5k**

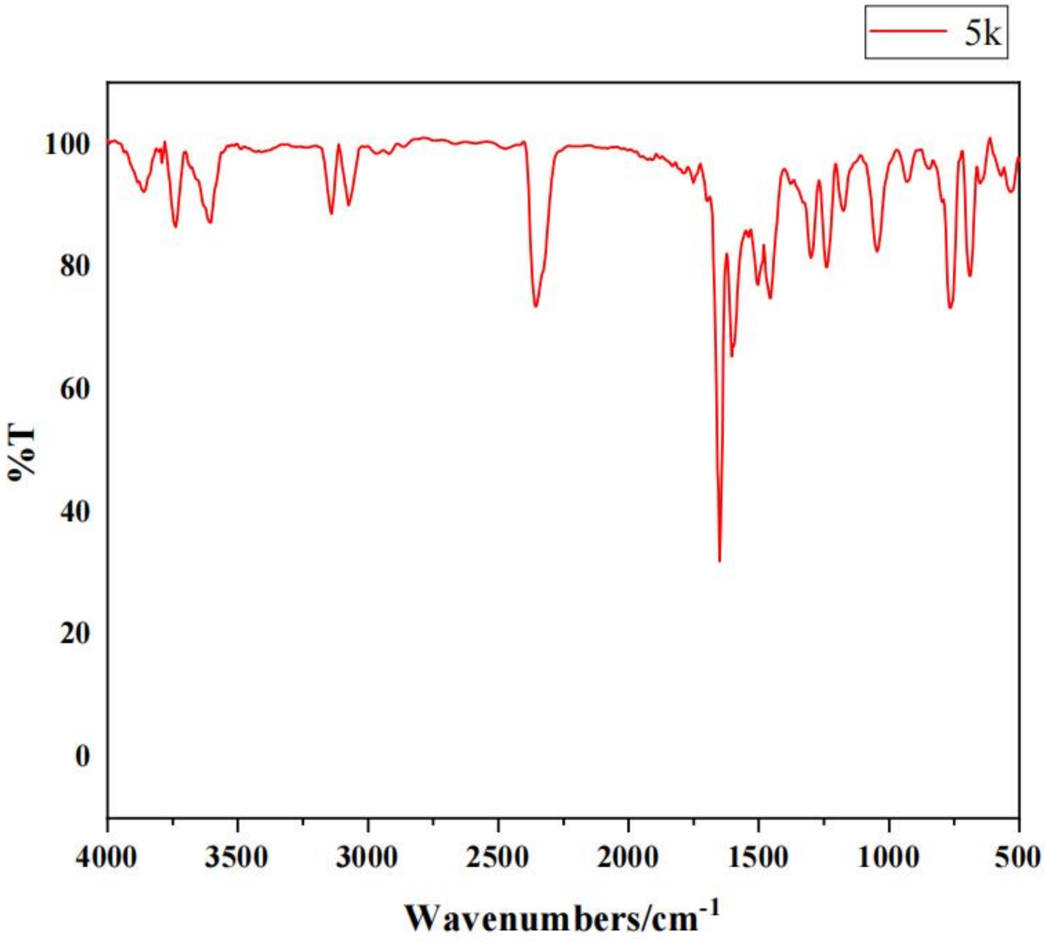

FT-IR spectrum of compound **5k**

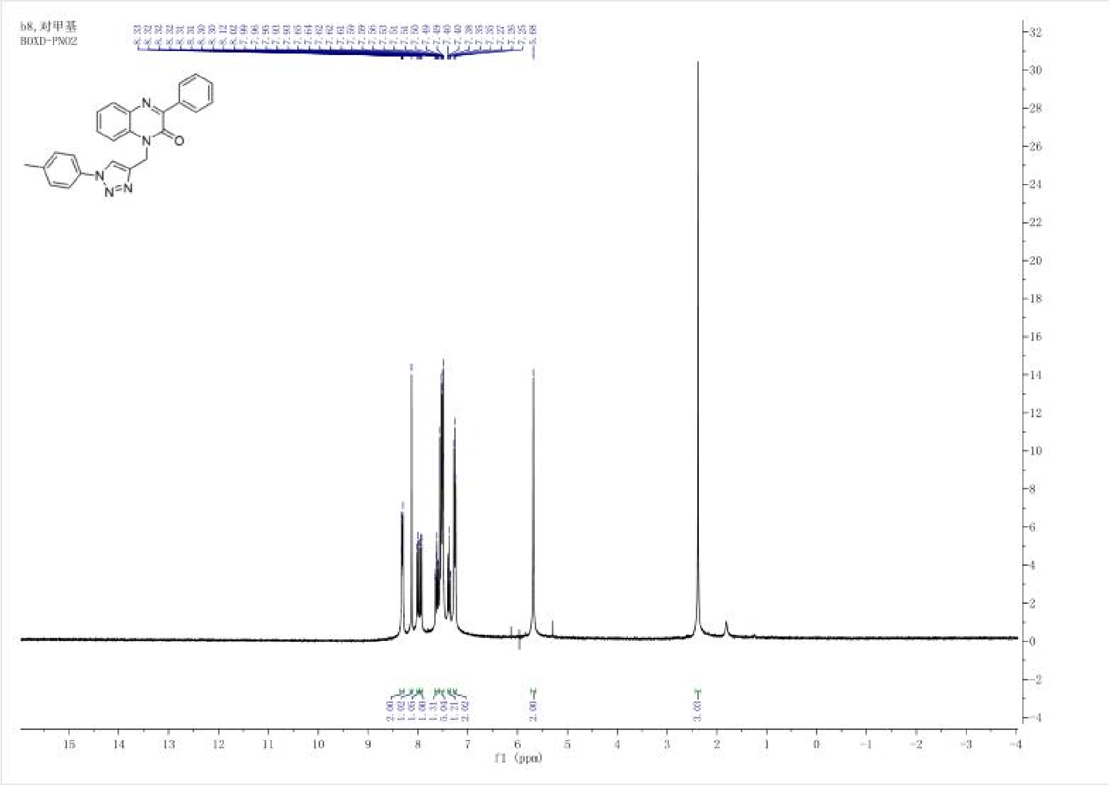

^{1}H-NMR (300 MHz, $CDCl_3$) spectrum of compound **5l**

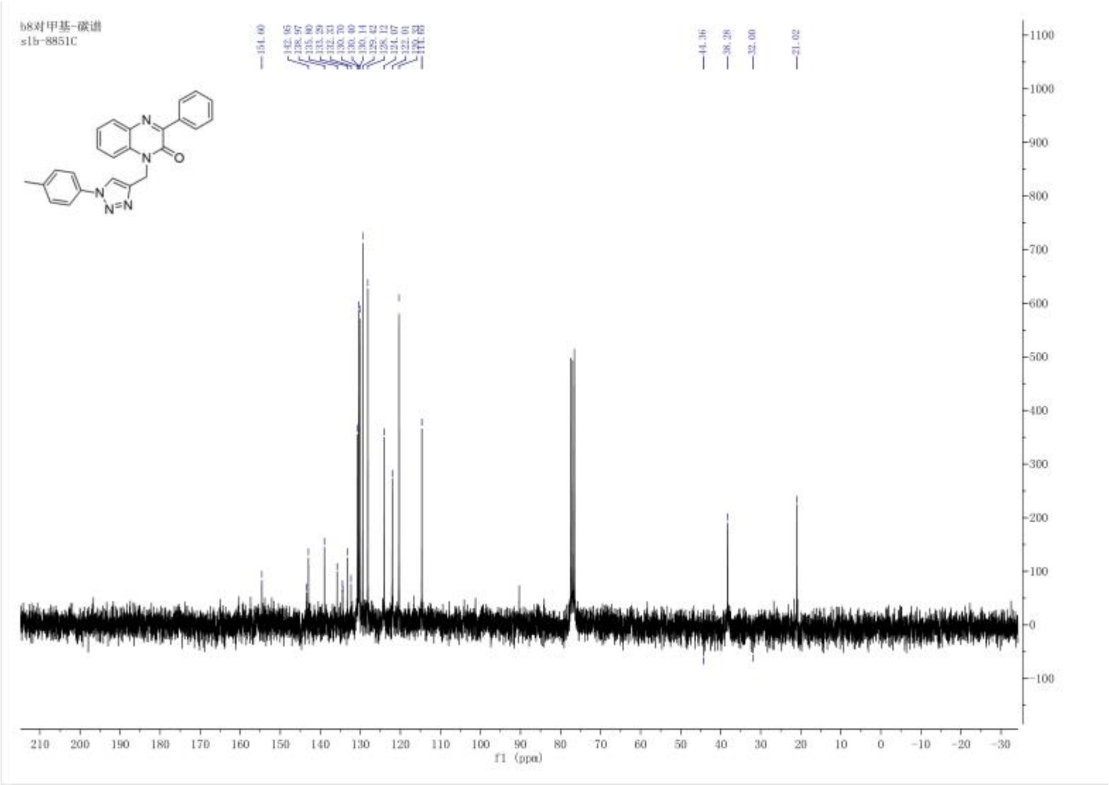

^{13}C NMR (75 MHz, $CDCl_3$) spectrum of compound **5l**

Elemental Composition Report Page 1

Single Mass Analysis
Tolerance = 10.0 PPM / DBE: min = -1.5, max = 50.0
Element prediction: Off
Number of isotope peaks used for i-FIT = 3

Monoisotopic Mass, Even Electron Ions
143 formula(e) evaluated with 1 results within limits (up to 50 closest results for each mass)
Elements Used:
C: 24-24 H: 0-80 N: 0-6 O: 0-20
3
0223-1-19 211 (1.187) 1: TOF MS ES+ 6.32e+006

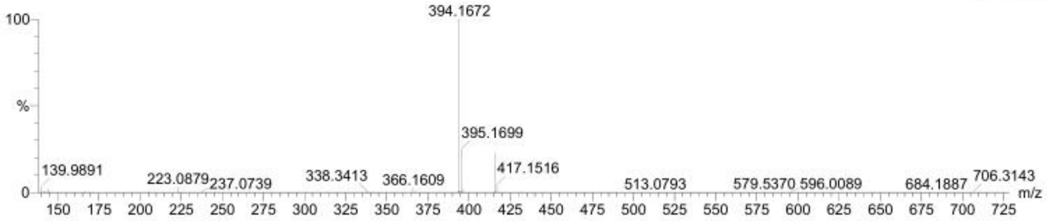

Minimum:				-1.5				
Maximum:		5.0	10.0	50.0				
Mass	Calc. Mass	mDa	PPM	DBE	i-FIT	Norm	Conf(%)	Formula
394.1672	394.1668	0.4	1.0	17.5	1086.5	n/a	n/a	C24 H20 N5 O

HRMS spectrum of compound **5l**

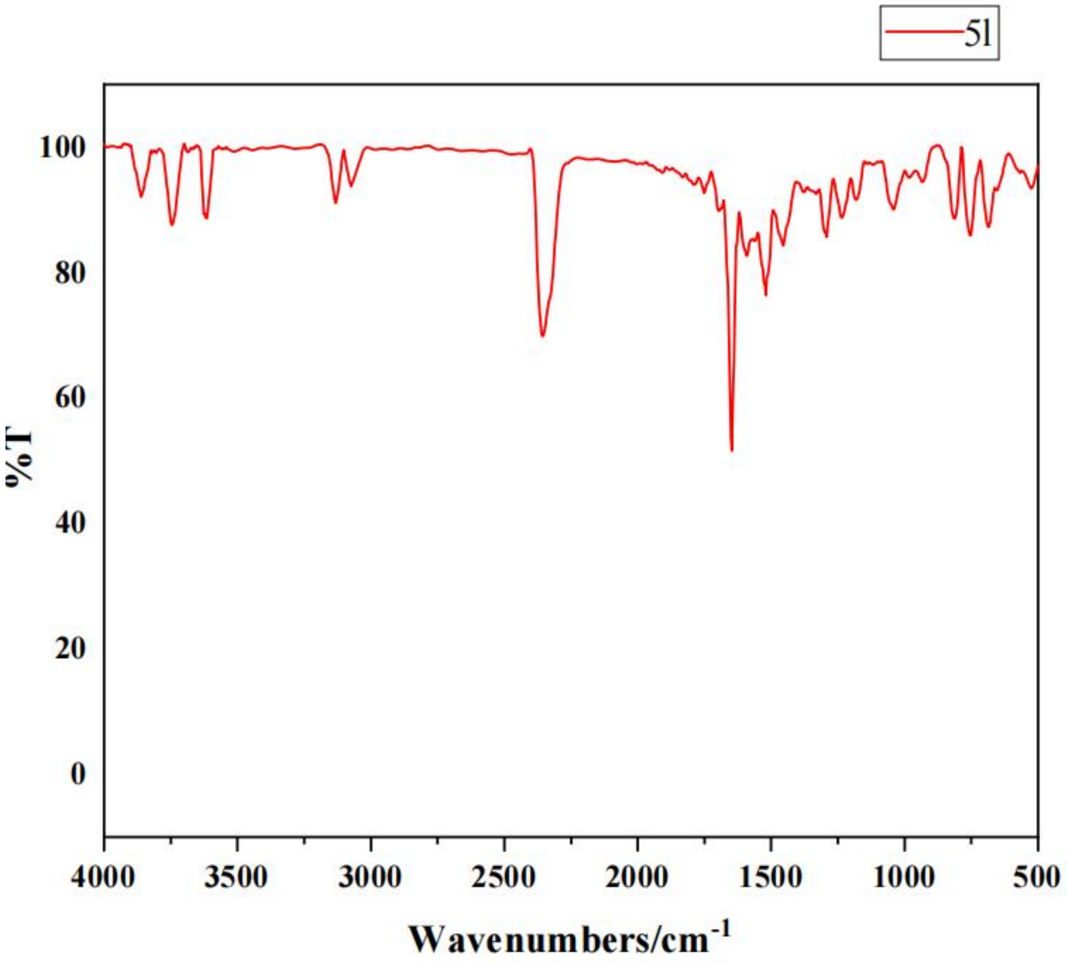

FT-IR spectrum of compound **5l**

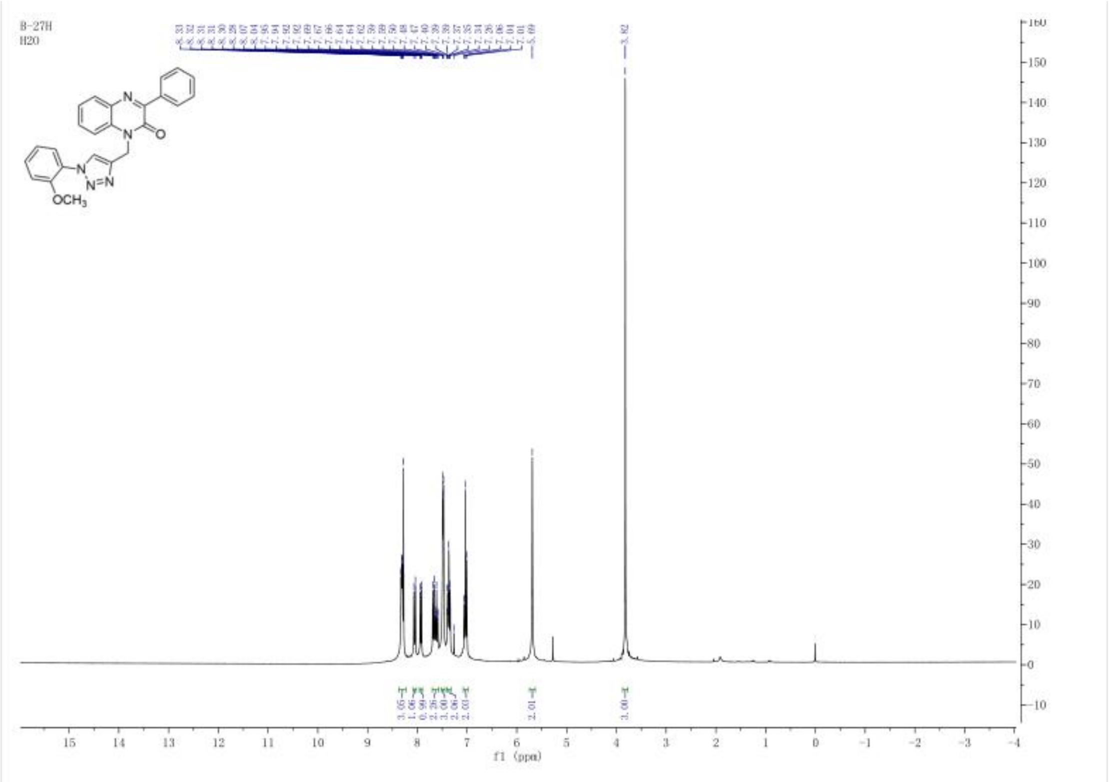

^{1}H-NMR (300 MHz, $CDCl_3$) spectrum of compound **5m**

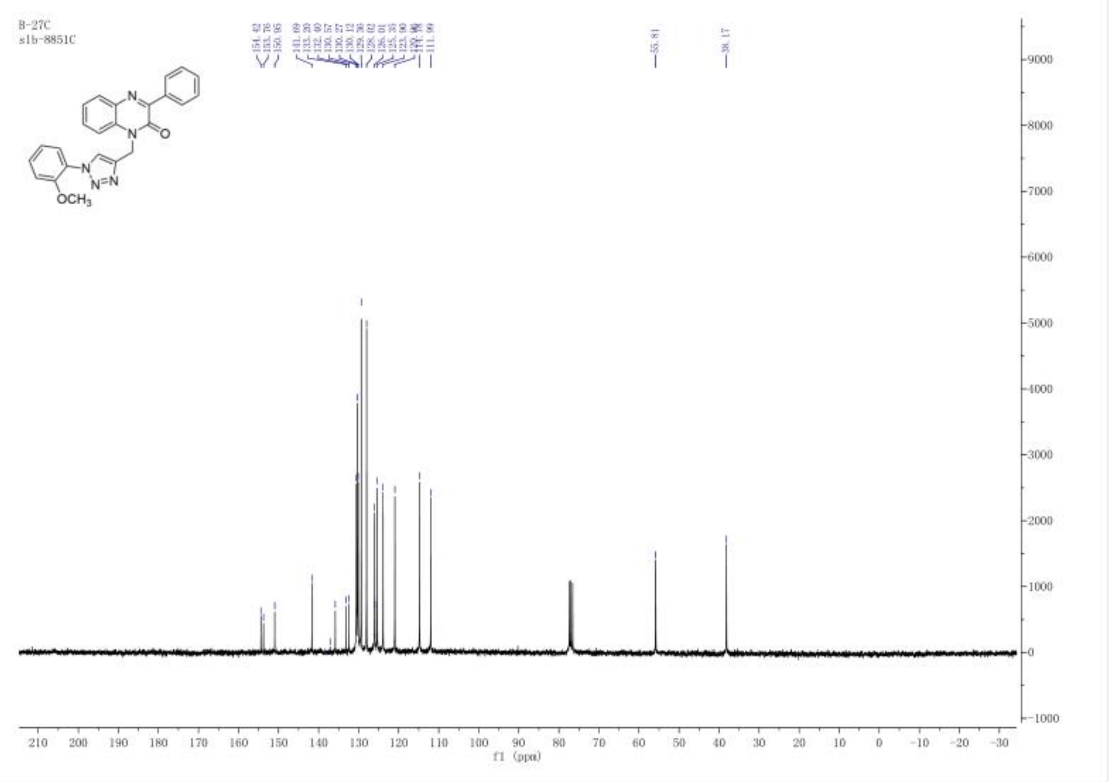

^{13}C NMR (75 MHz, $CDCl_3$) spectrum of compound **5m**

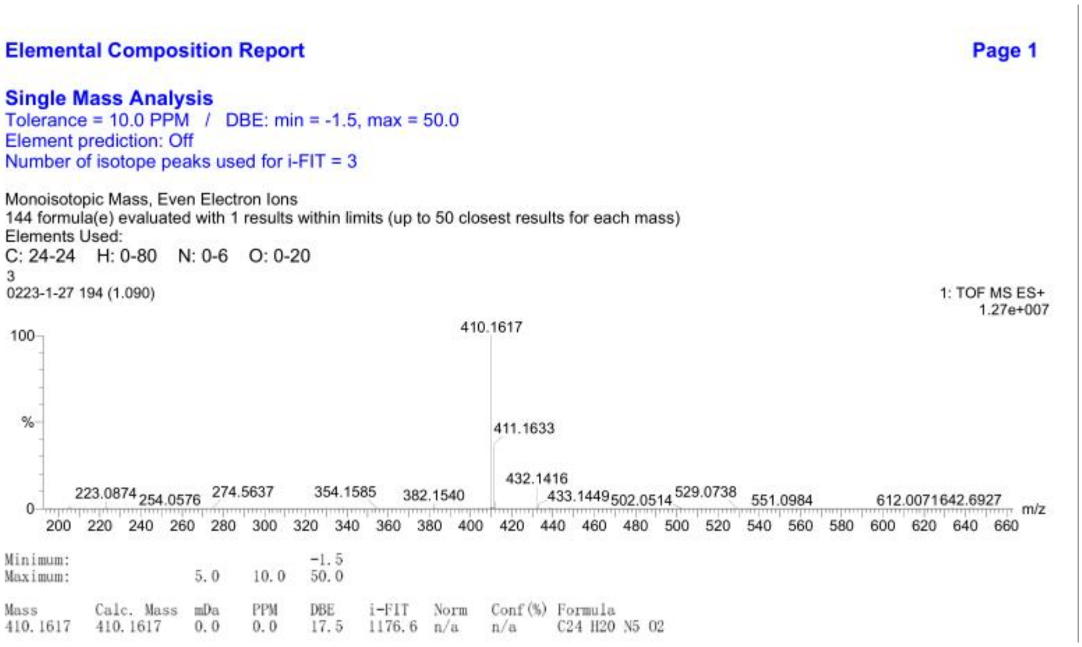

HRMS spectrum of compound **5m**

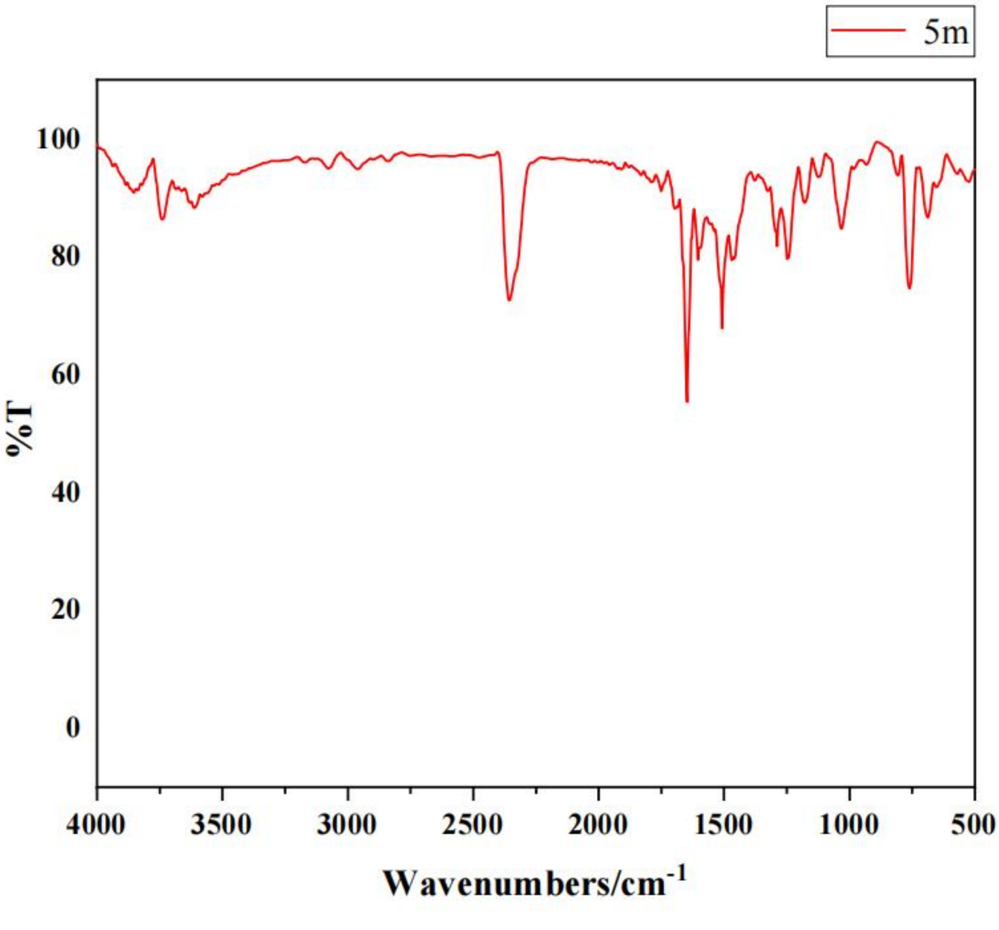

FT-IR spectrum of compound **5m**

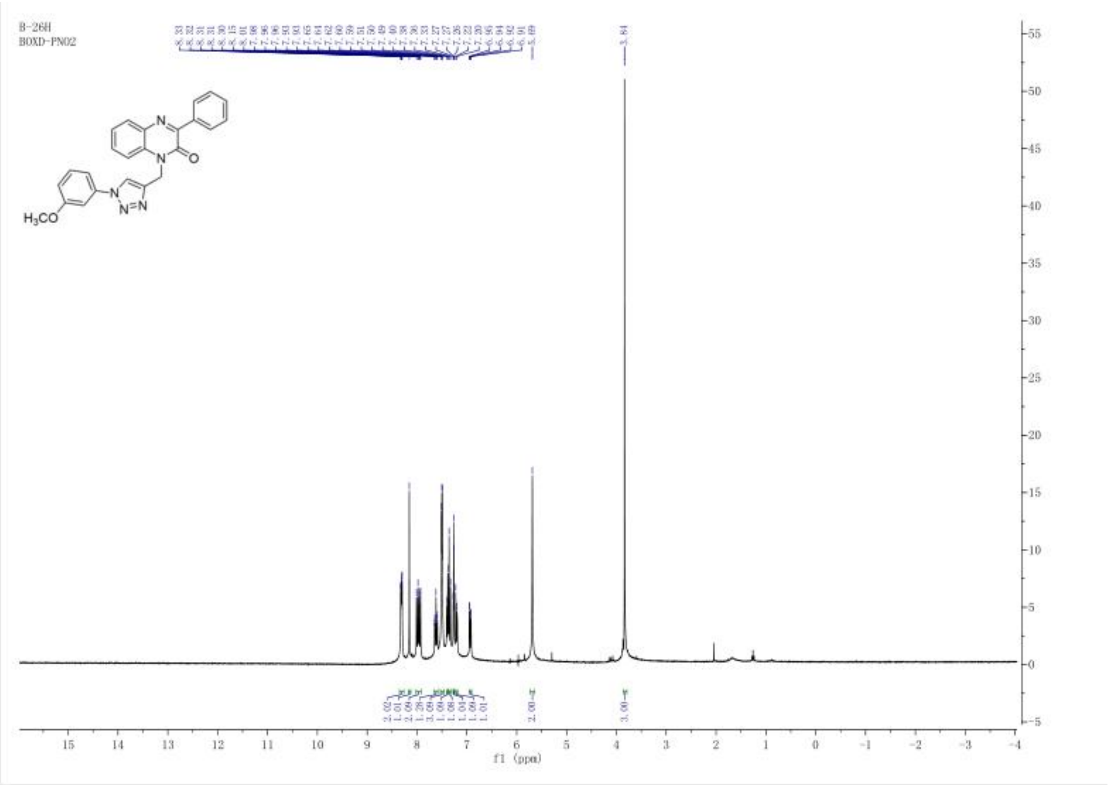

^{1}H-NMR (300 MHz, $CDCl_3$) spectrum of compound **5n**

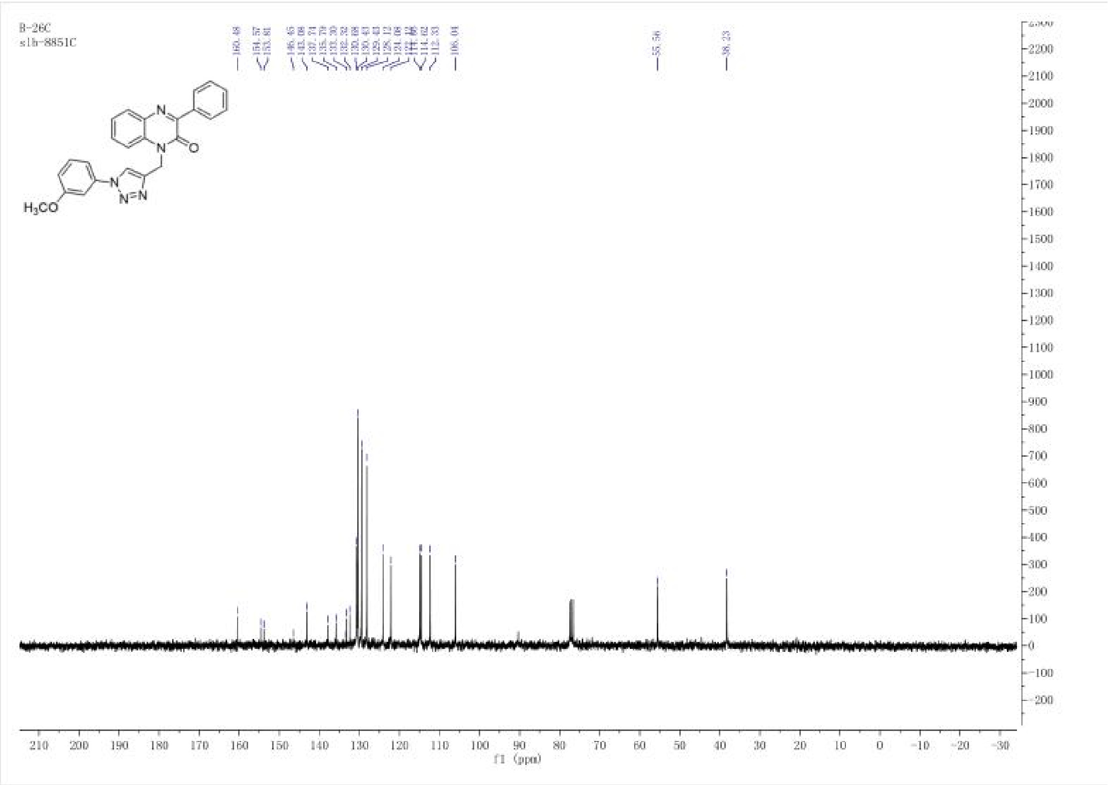

^{13}C NMR (75 MHz, $CDCl_3$) spectrum of compound **5n**

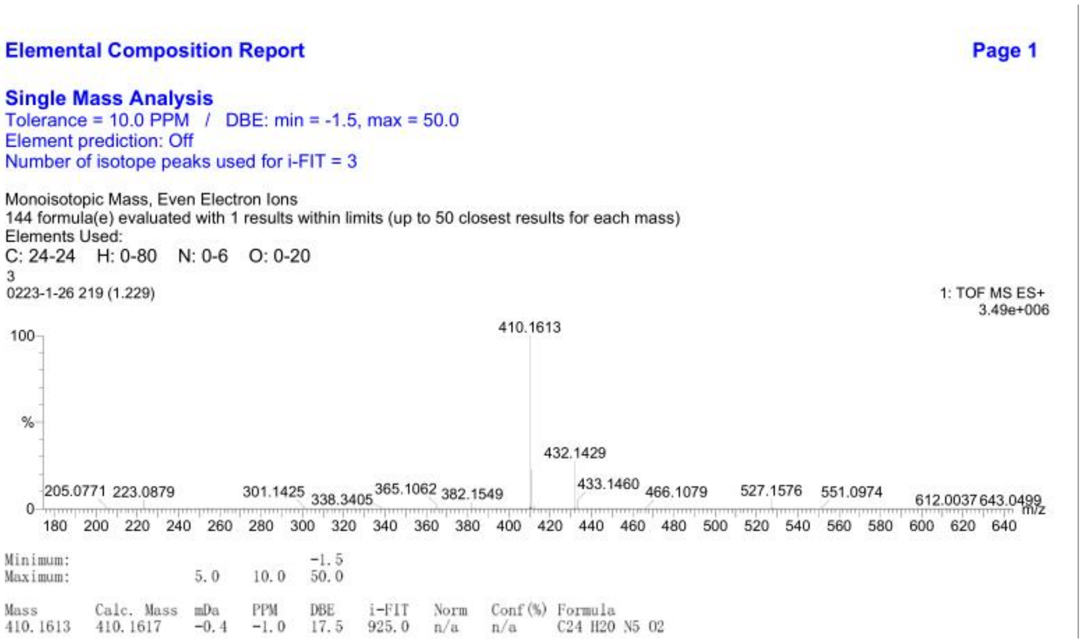

HRMS spectrum of compound **5n**

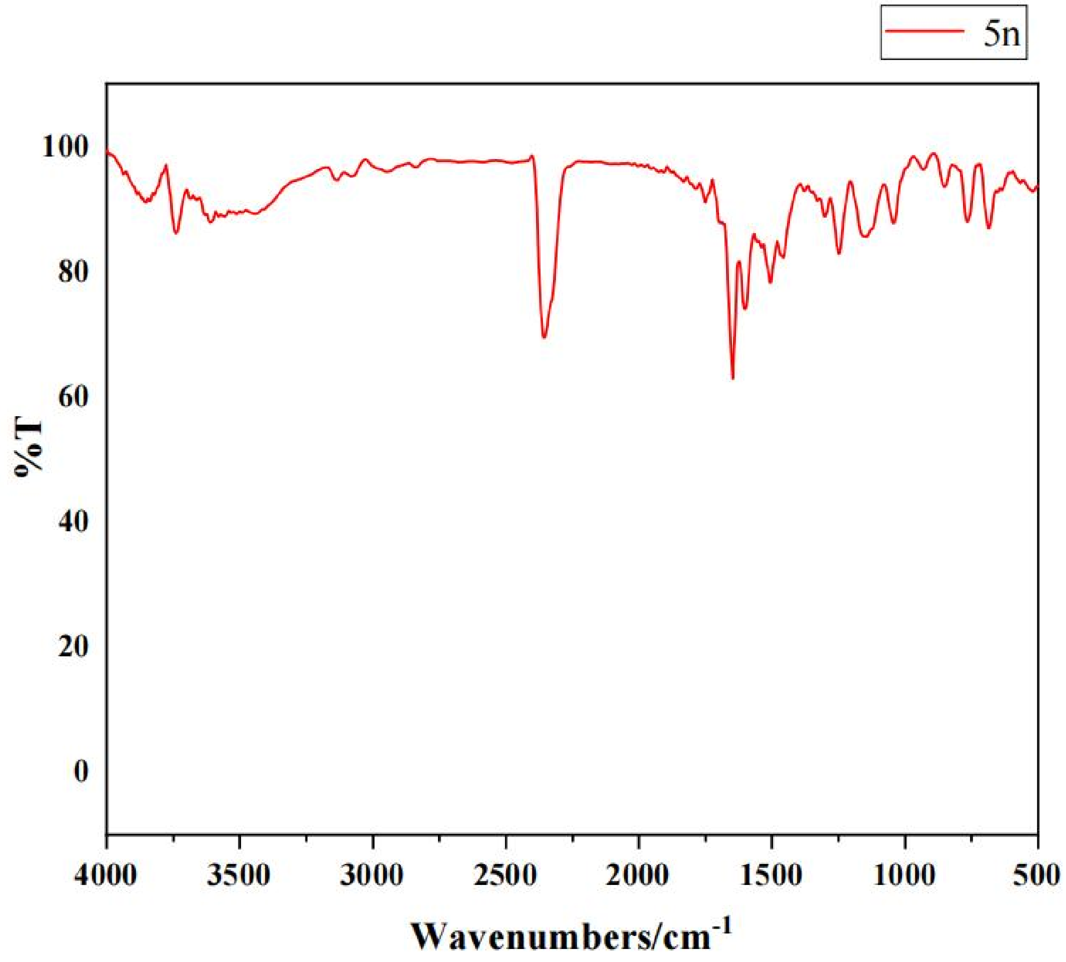

FT-IR spectrum of compound **5n**

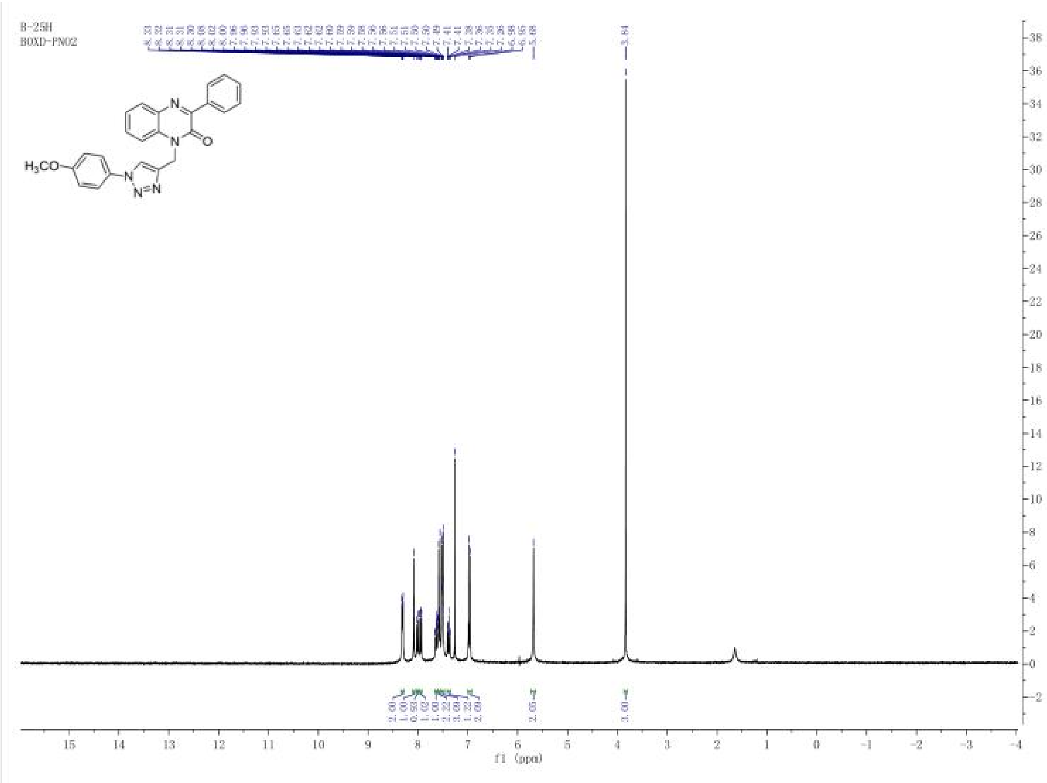

^{1}H-NMR (300 MHz, $CDCl_3$) spectrum of compound **5o**

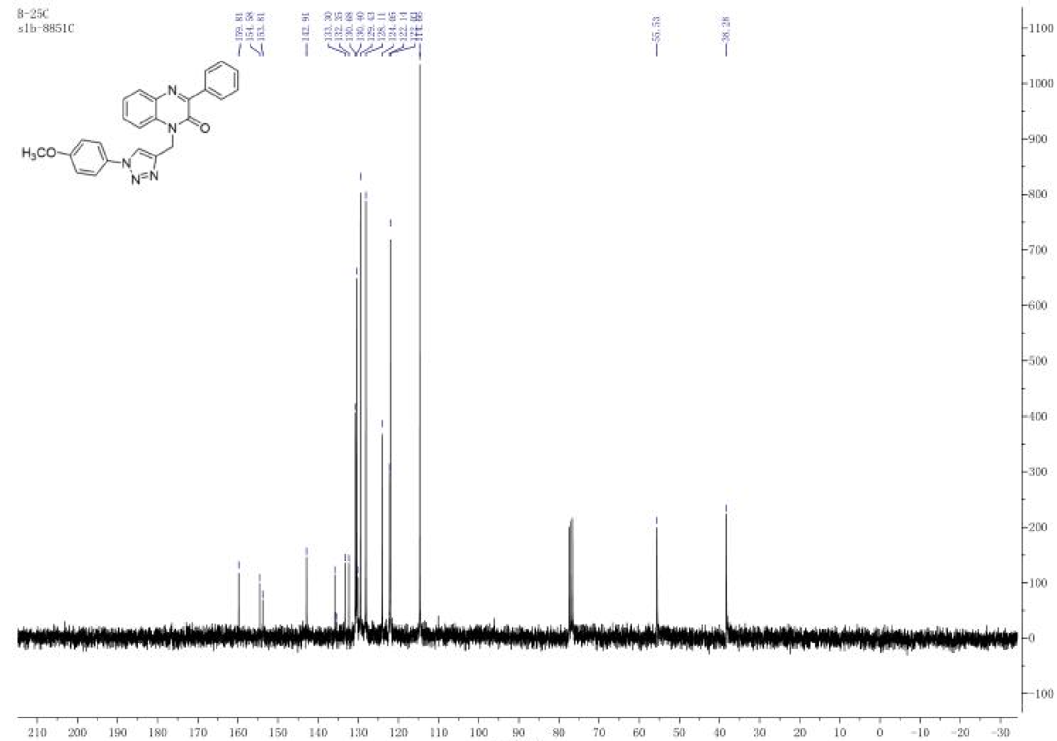

^{13}C NMR (75 MHz, $CDCl_3$) spectrum of compound **5o**

Elemental Composition Report **Page 1**

Single Mass Analysis
Tolerance = 10.0 PPM / DBE: min = -1.5, max = 50.0
Element prediction: Off
Number of isotope peaks used for i-FIT = 3

Monoisotopic Mass, Even Electron Ions
144 formula(e) evaluated with 1 results within limits (up to 50 closest results for each mass)
Elements Used:
C: 24-24 H: 0-80 N: 0-6 O: 0-20
3
0223-1-25 193 (1.085) 1: TOF MS ES+
2.35e+006

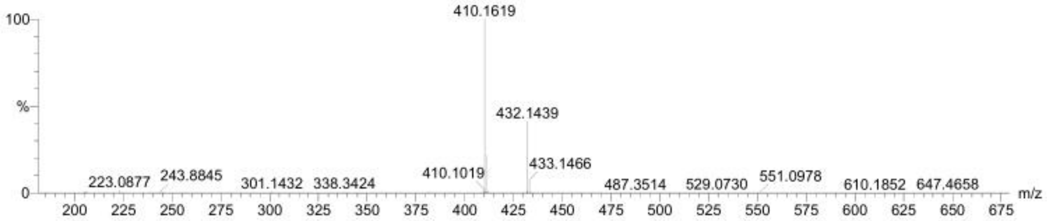

Minimum:				-1.5
Maximum:		5.0	10.0	50.0

Mass	Calc. Mass	mDa	PPM	DBE	i-FIT	Norm	Conf(%)	Formula
410.1619	410.1617	0.2	0.5	17.5	892.6	n/a	n/a	C24 H20 N5 O2

HRMS spectrum of compound **5o**

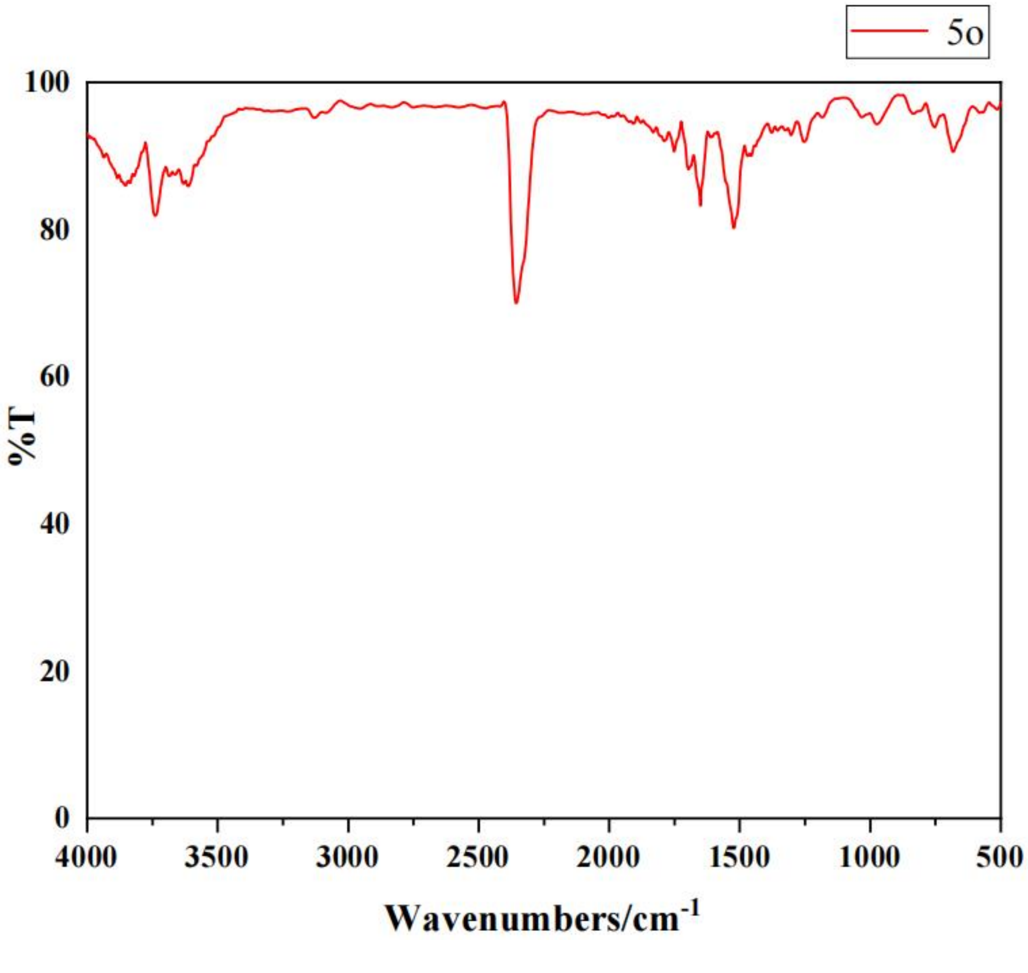

FT-IR spectrum of compound **5o**

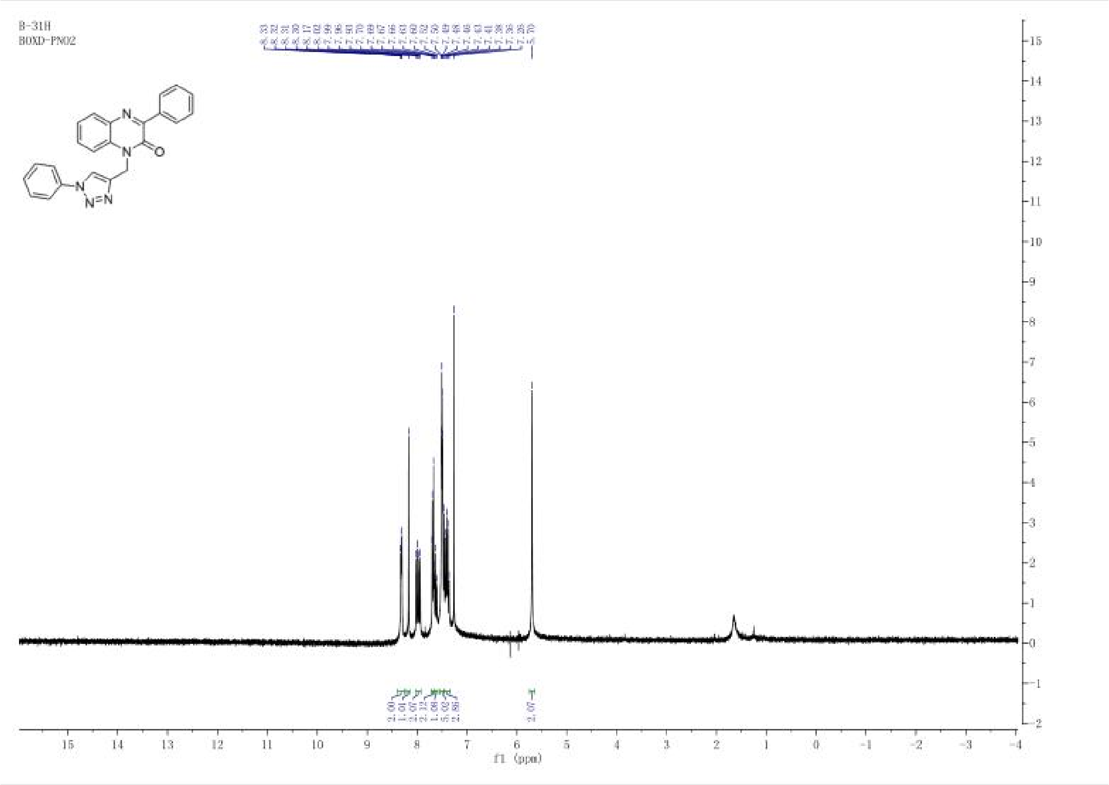

^{1}H-NMR (300 MHz, $CDCl_3$) spectrum of compound **5p**

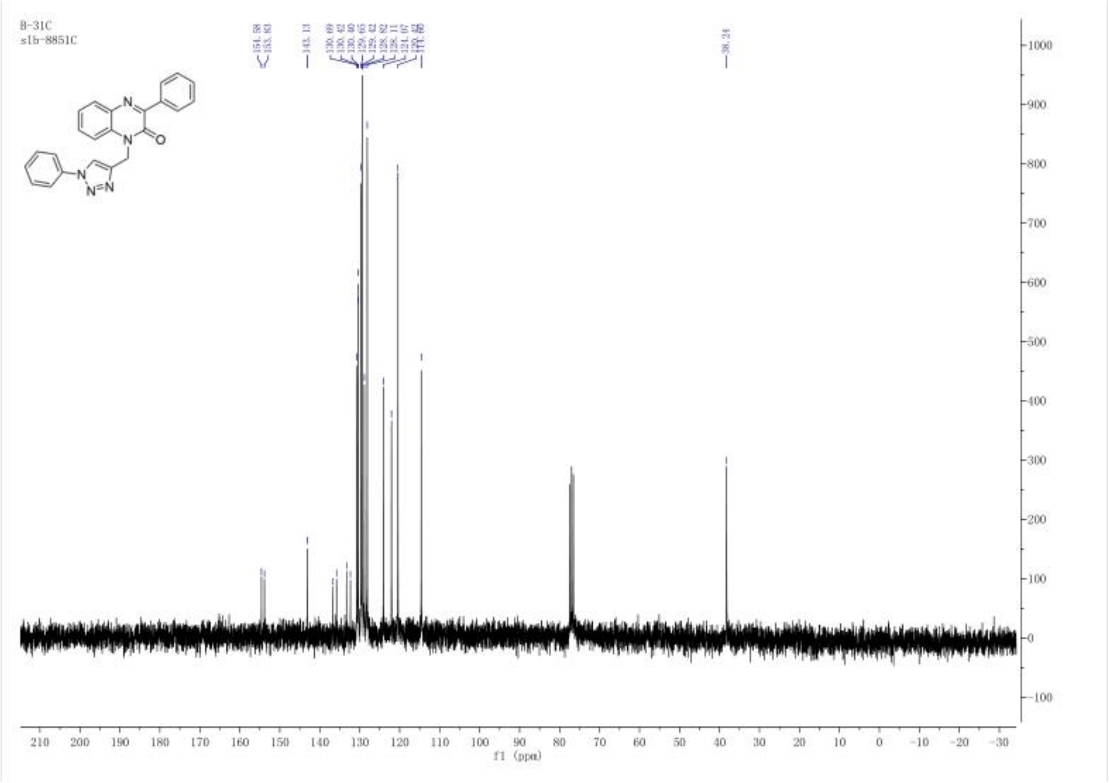

^{13}C NMR (75 MHz, $CDCl_3$) spectrum of compound **5p**

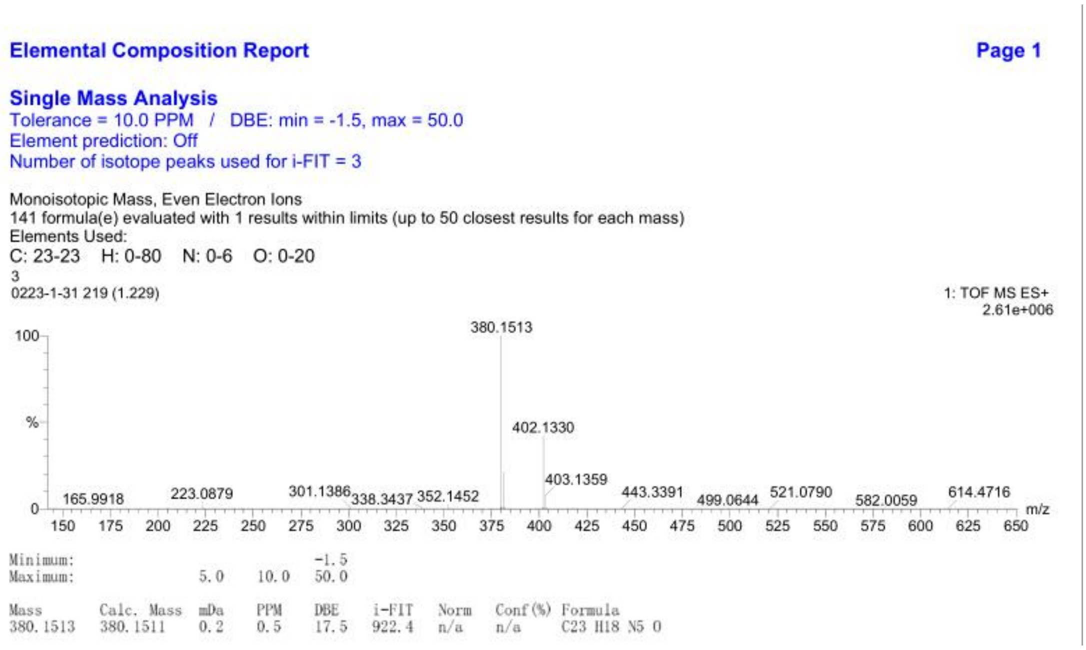

HRMS spectrum of compound **5p**

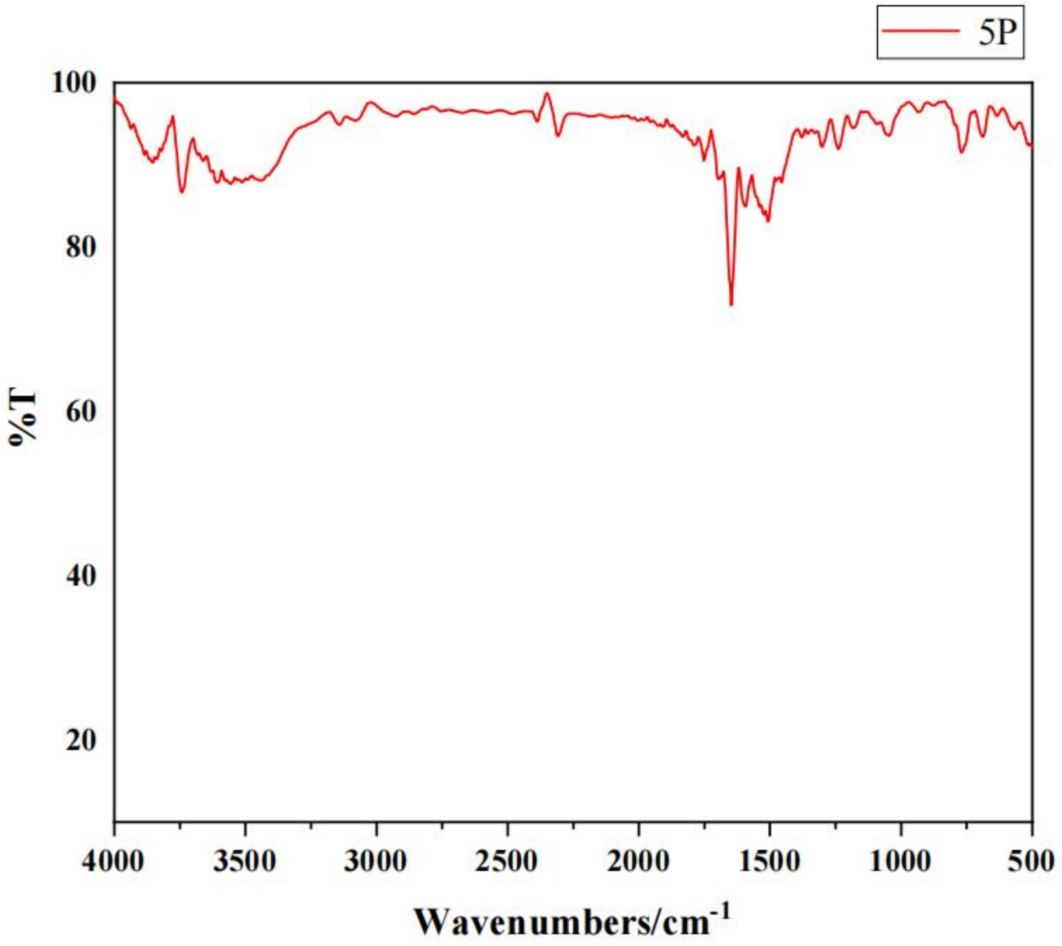

FT-IR spectrum of compound **5p**

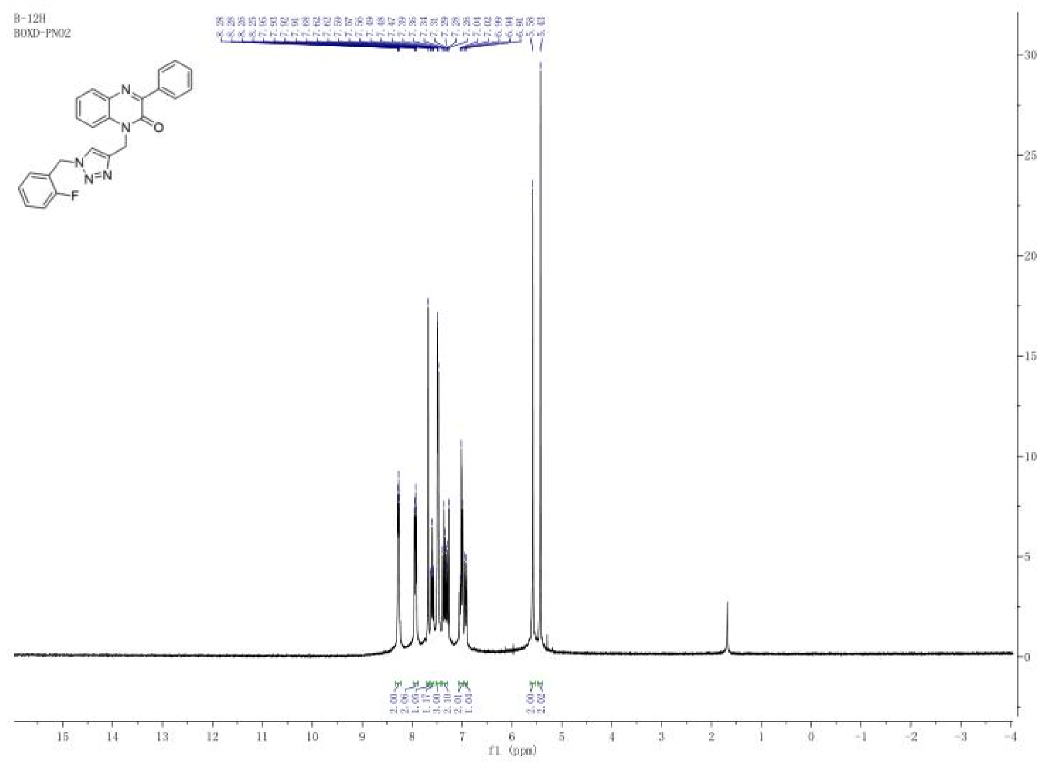

^{1}H-NMR (300 MHz, $CDCl_3$) spectrum of compound **6a**

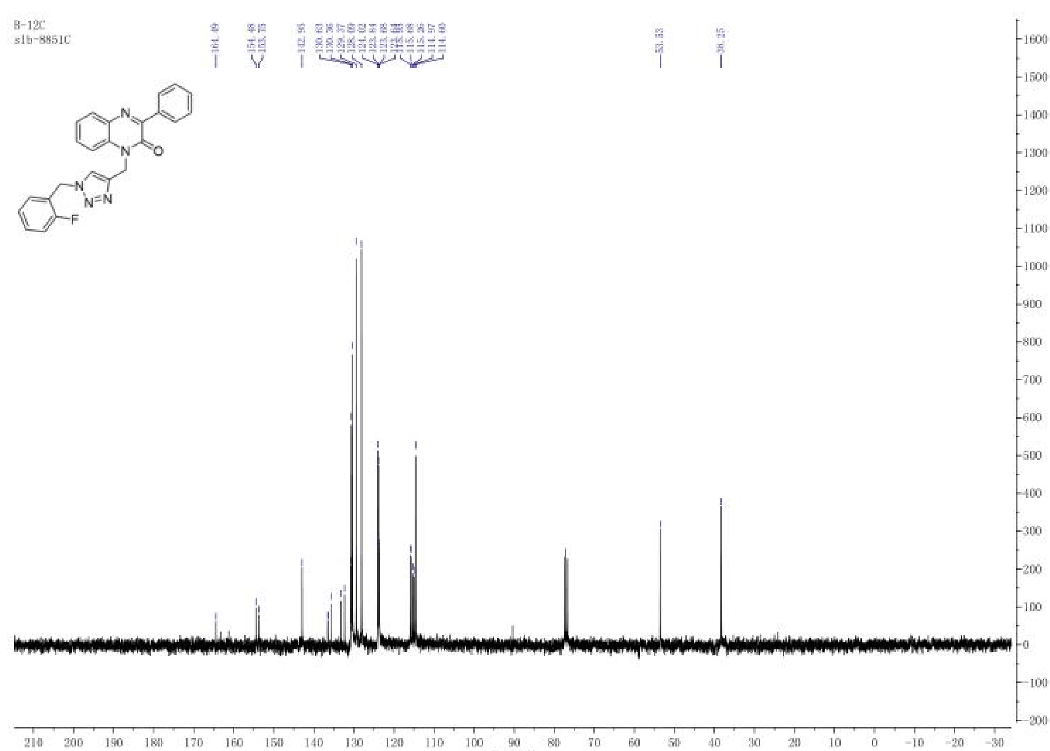

^{13}C NMR (75 MHz, $CDCl_3$) spectrum of compound **6a**

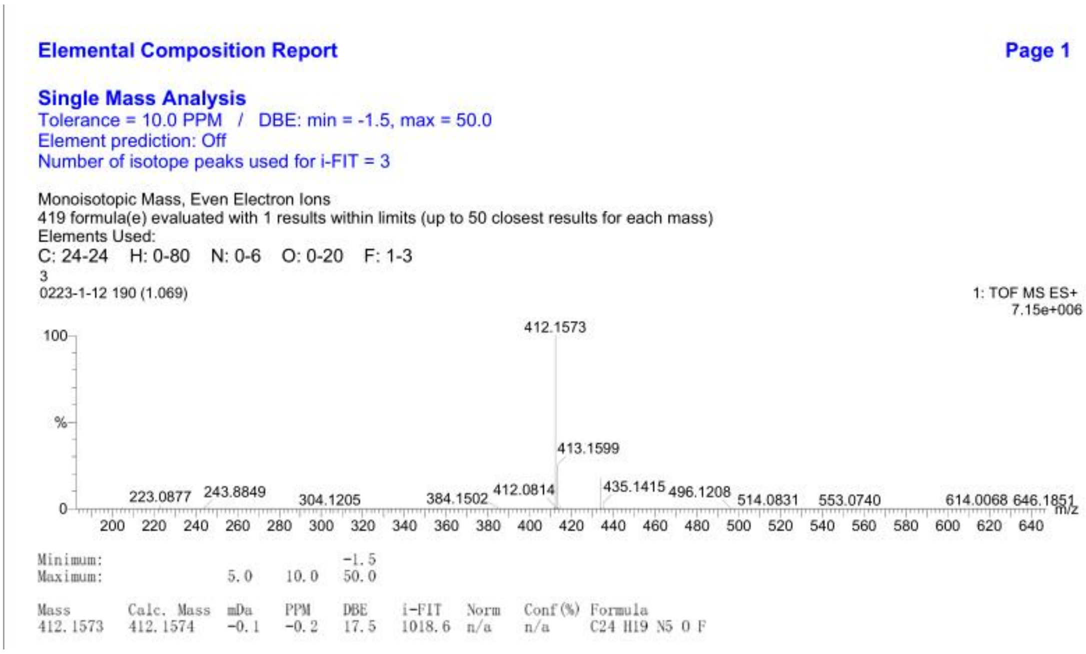

HRMS spectrum of compound **6a**

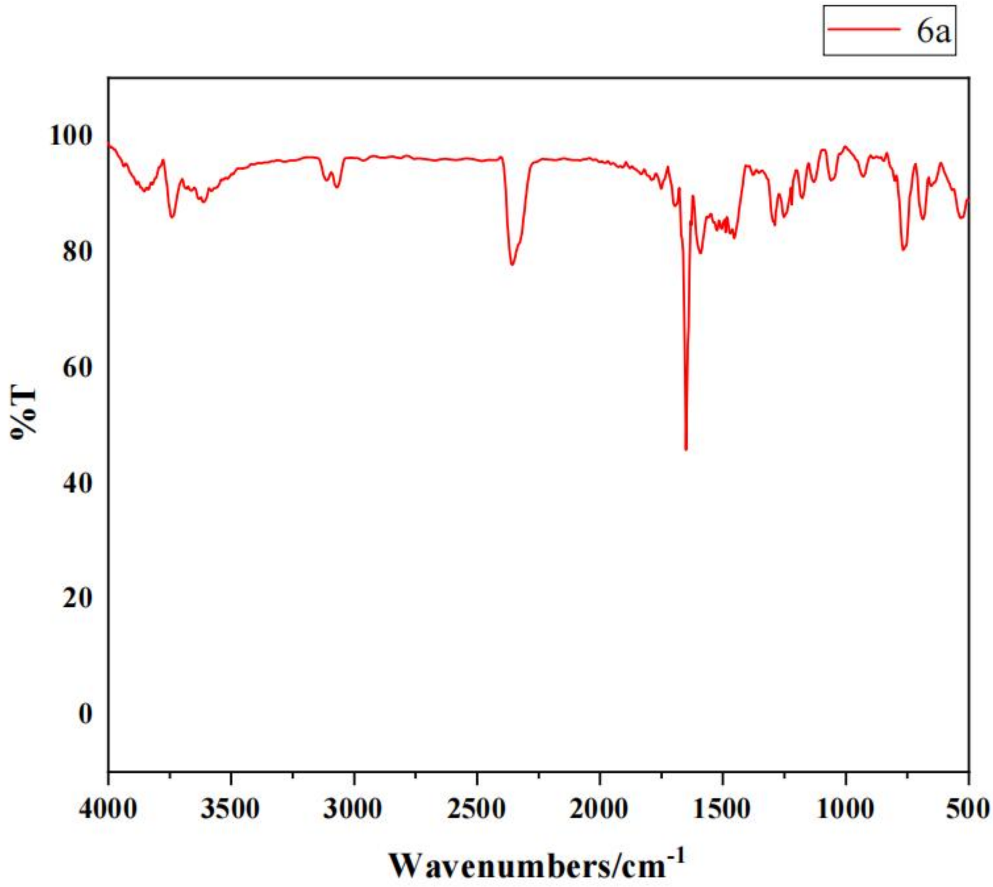

FT-IR spectrum of compound **6a**

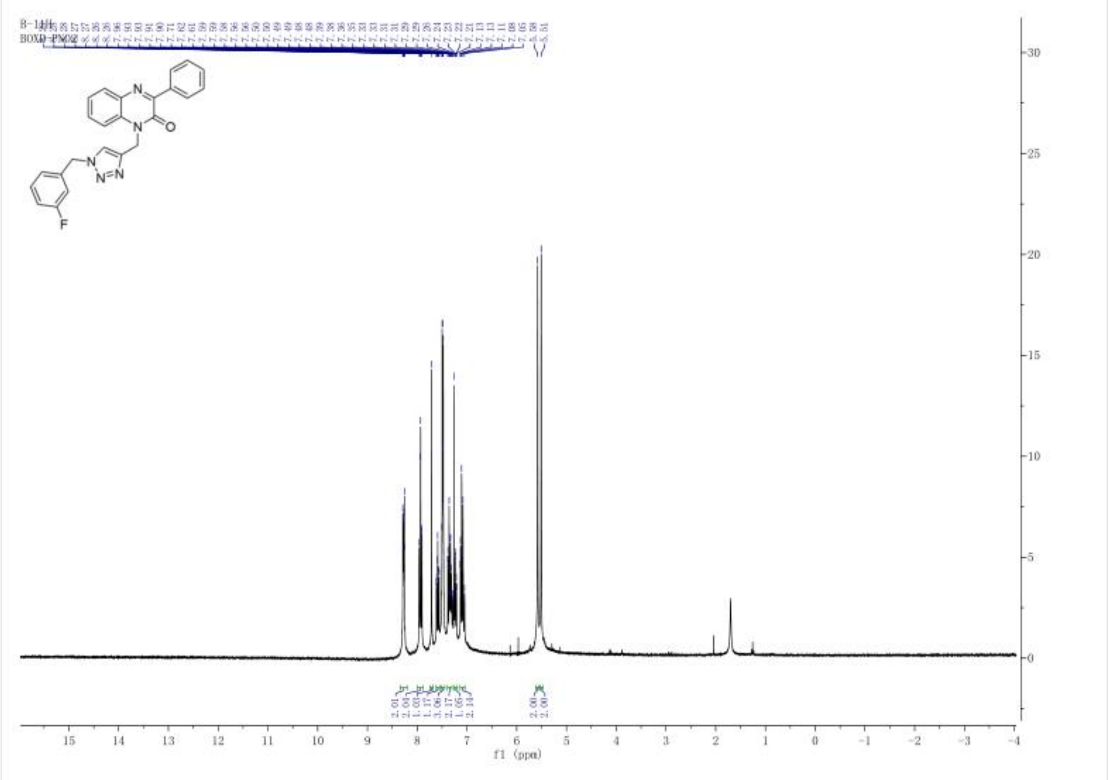

^{1}H-NMR (300 MHz, $CDCl_3$) spectrum of compound **6b**

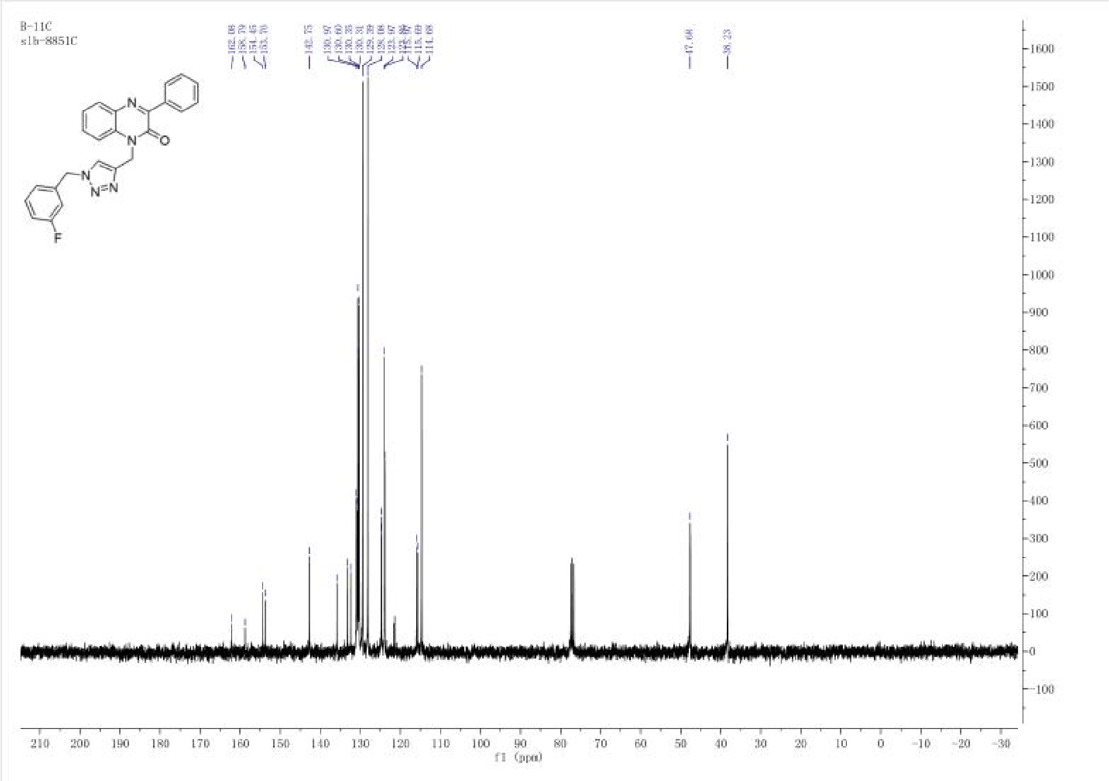

^{13}C NMR (75 MHz, $CDCl_3$) spectrum of compound **6b**

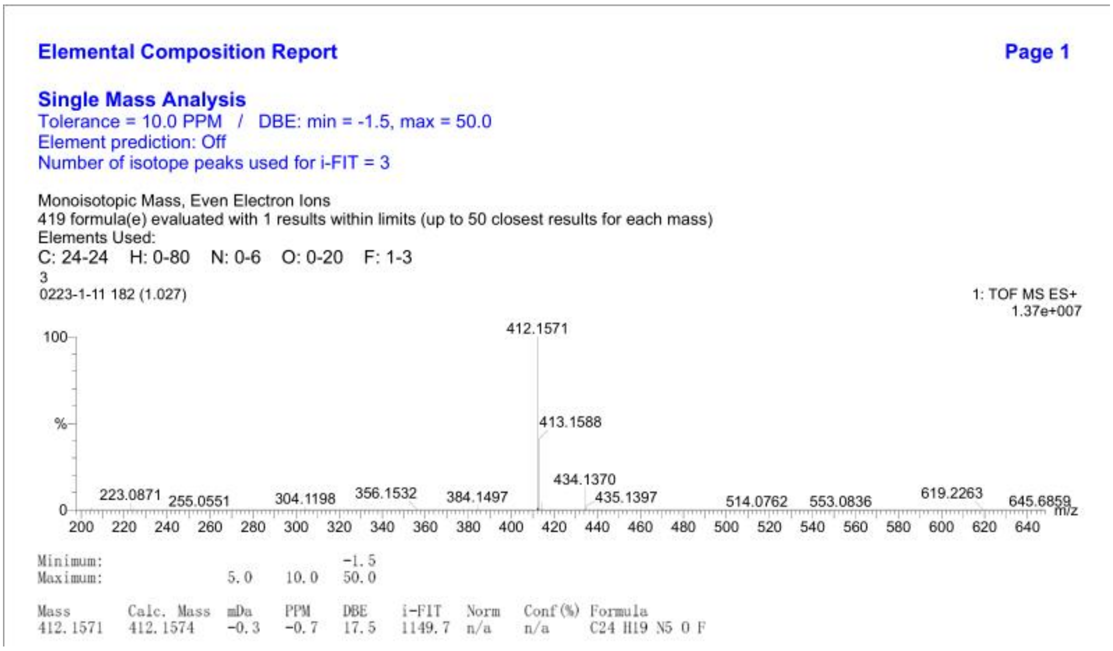

HRMS spectrum of compound **6b**

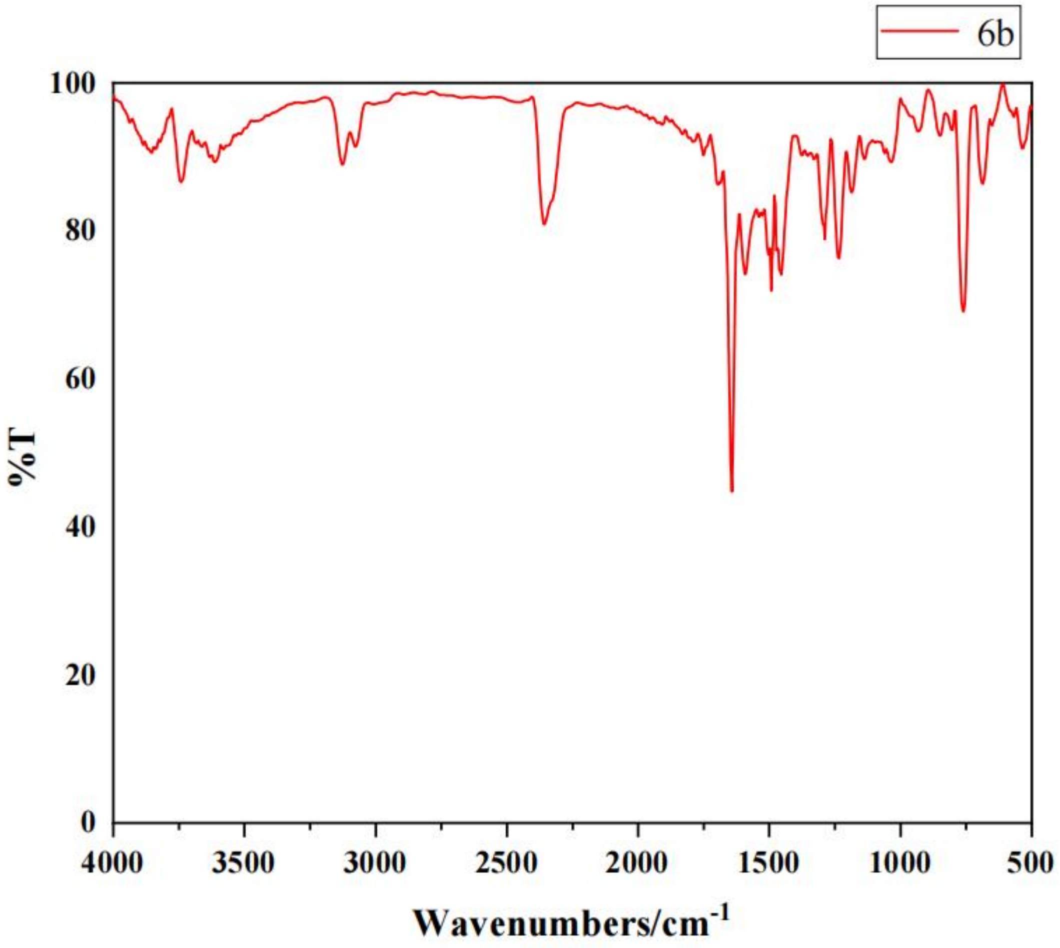

FT-IR spectrum of compound **6b**

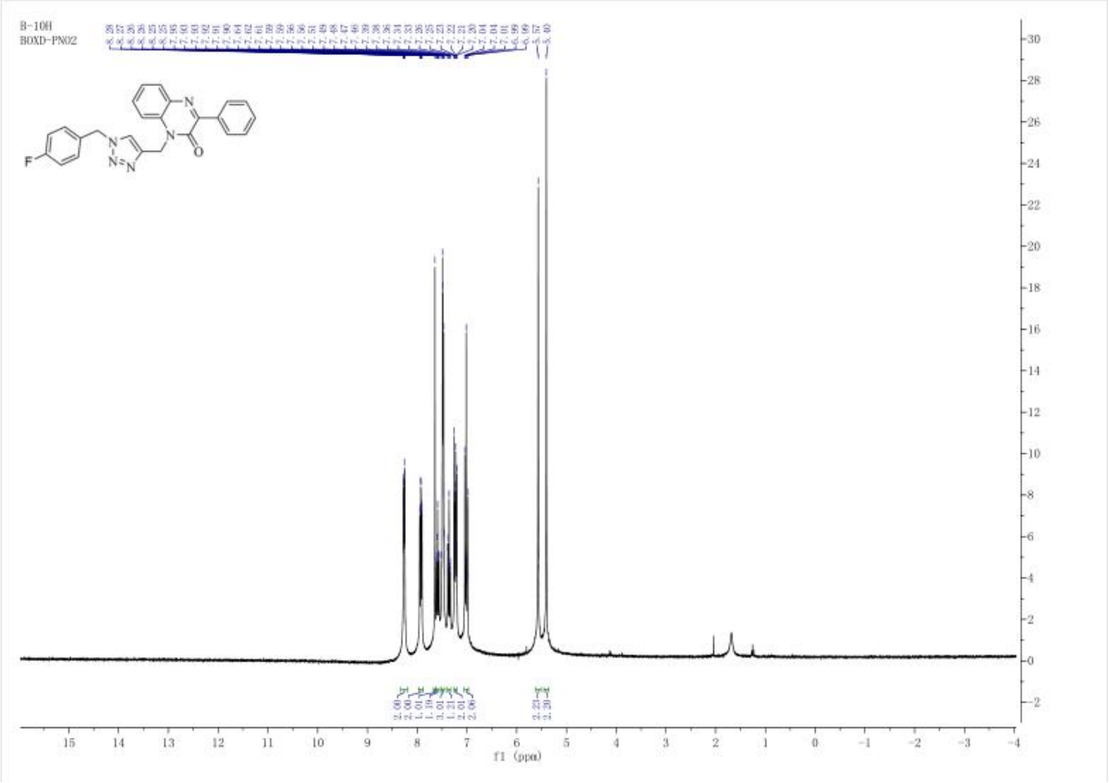

^{1}H-NMR (300 MHz, $CDCl_3$) spectrum of compound **6c**

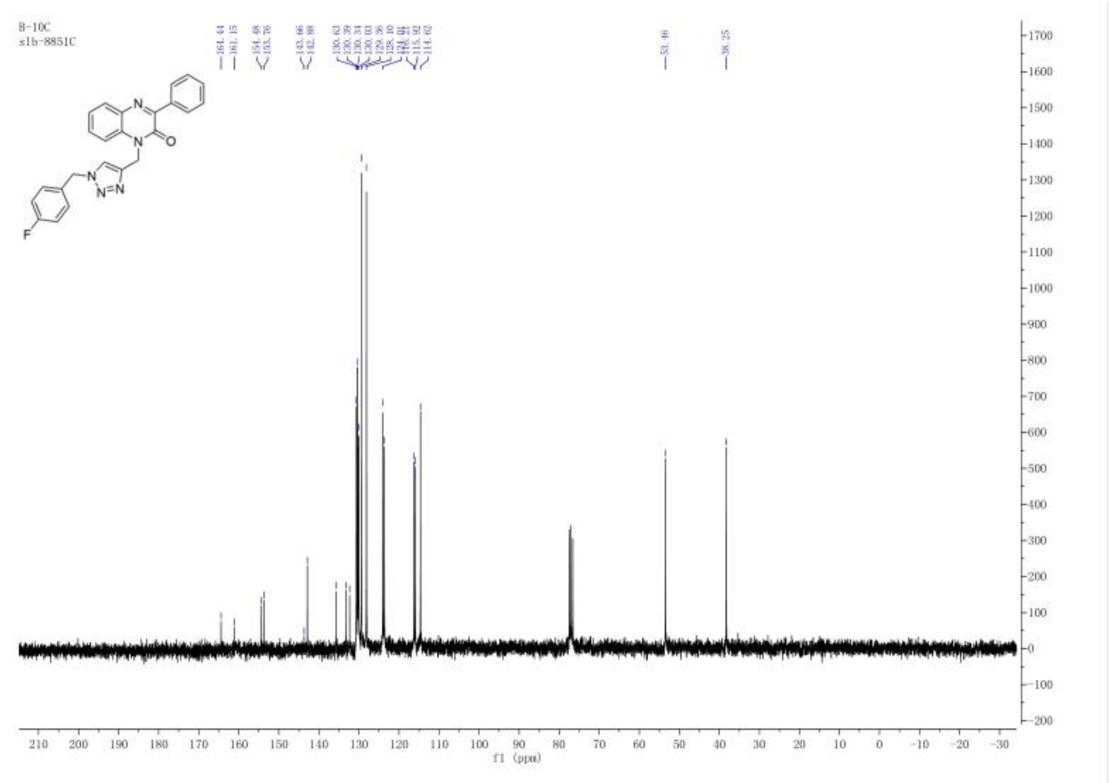

^{13}C NMR (75 MHz, $CDCl_3$) spectrum of compound **6c**

Elemental Composition Report **Page 1**

Single Mass Analysis
Tolerance = 10.0 PPM / DBE: min = -1.5, max = 50.0
Element prediction: Off
Number of isotope peaks used for i-FIT = 3

Monoisotopic Mass, Even Electron Ions
419 formula(e) evaluated with 1 results within limits (up to 50 closest results for each mass)
Elements Used:
C: 24-24 H: 0-80 N: 0-6 O: 0-20 F: 1-3
3
0223-1-10 186 (1.048) 1: TOF MS ES+ 1.27e+007

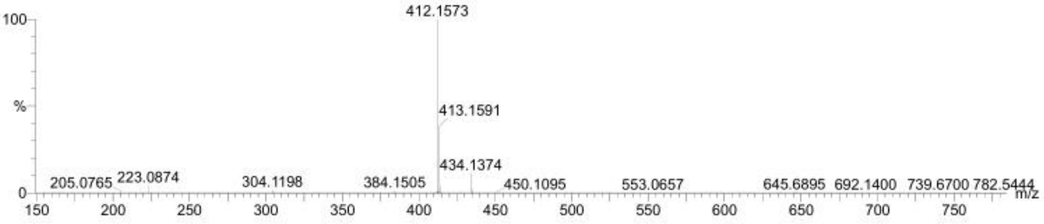

Minimum:				-1.5				
Maximum:		5.0	10.0	50.0				

Mass	Calc. Mass	mDa	PPM	DBE	i-FIT	Norm	Conf(%)	Formula
412.1573	412.1574	-0.1	-0.2	17.5	1172.6	n/a	n/a	C24 H19 N5 O F

HRMS spectrum of compound **6c**

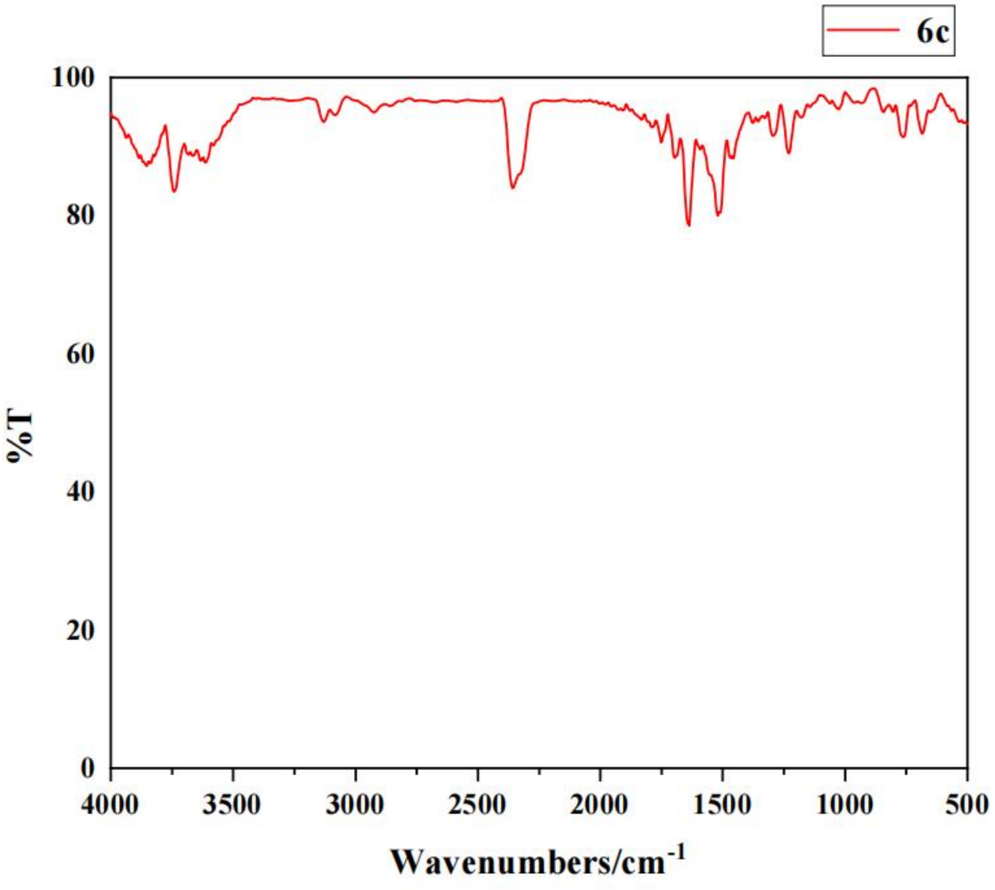

FT-IR spectrum of compound **6c**

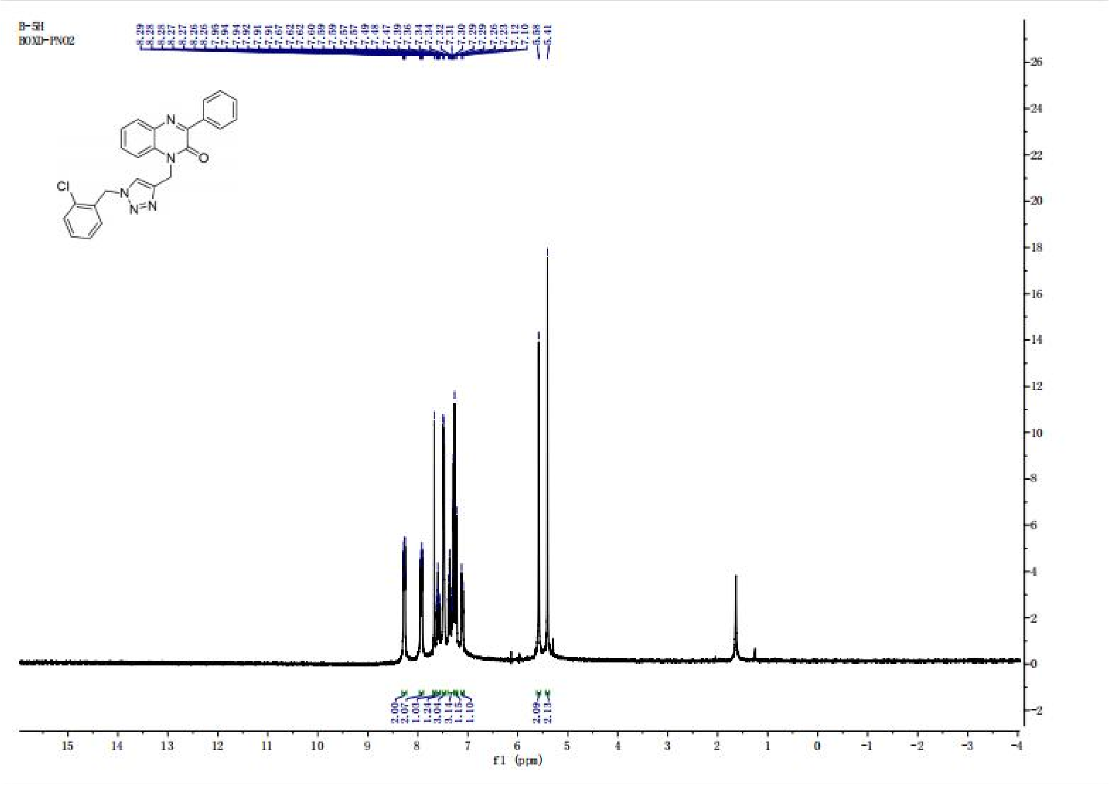

^{1}H-NMR (300 MHz, $CDCl_3$) spectrum of compound **6d**

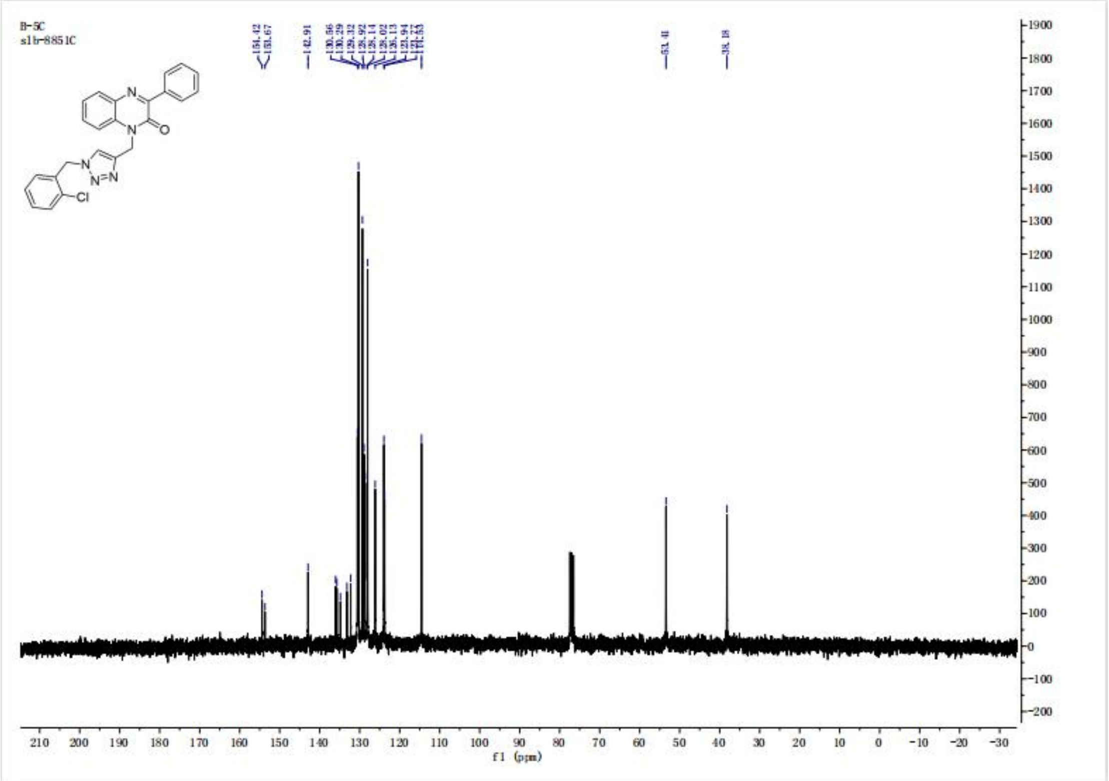

^{13}C NMR (75 MHz, $CDCl_3$) spectrum of compound **6d**

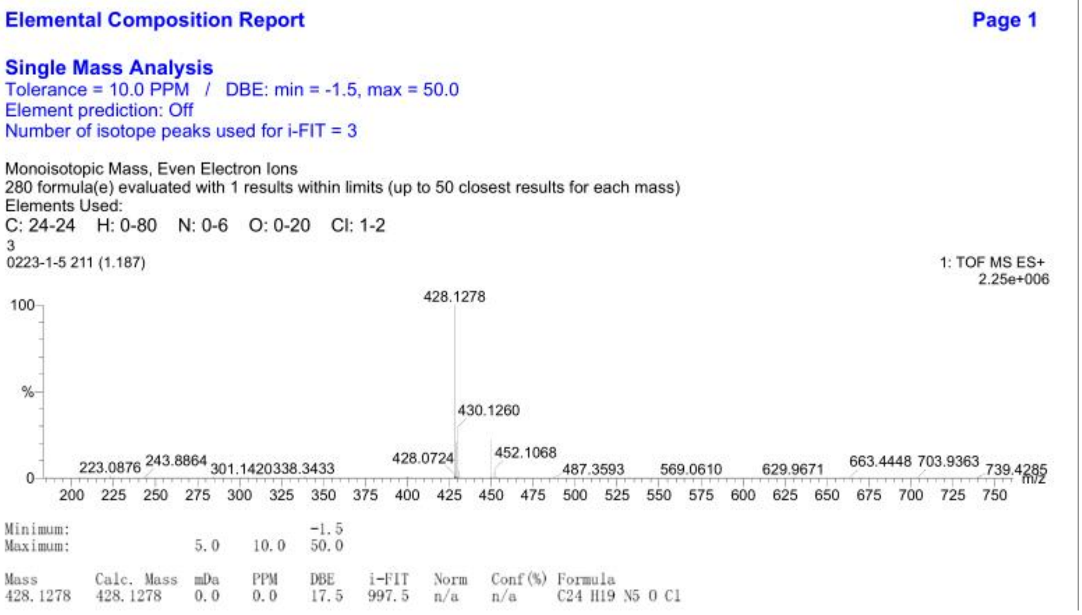

HRMS spectrum of compound **6d**

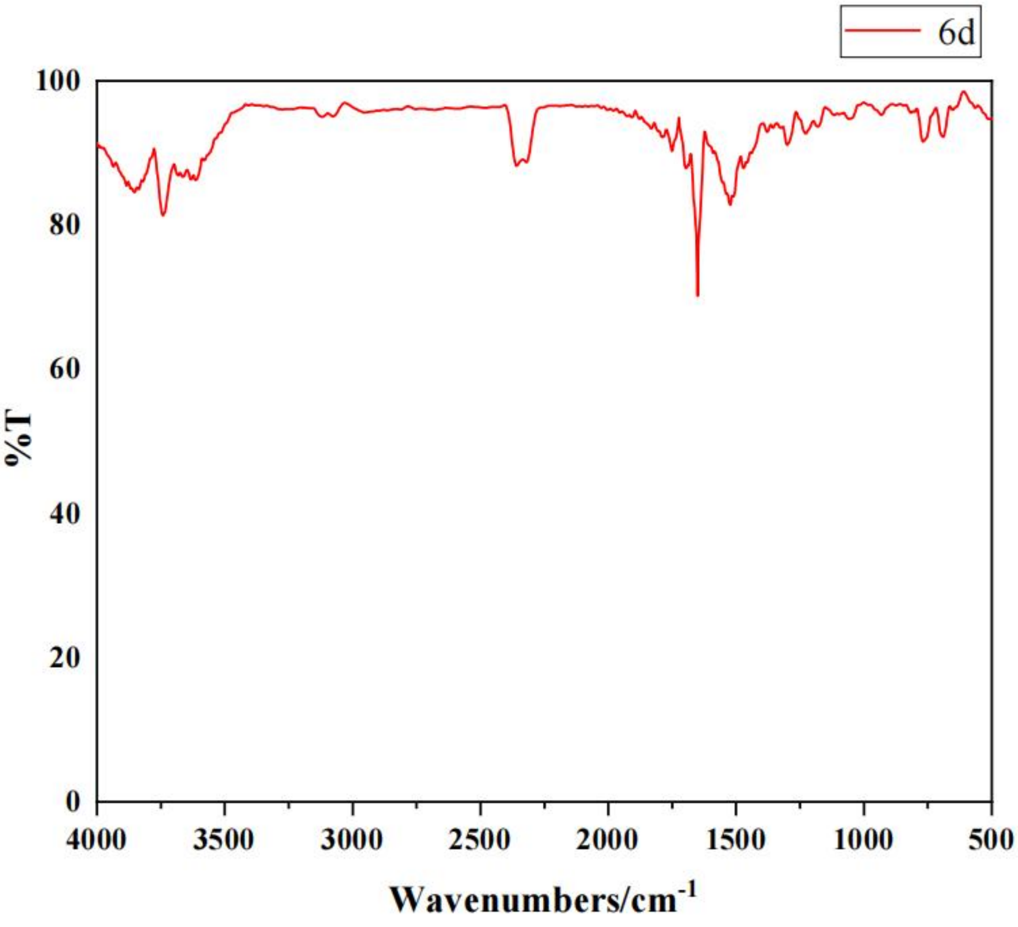

FT-IR spectrum of compound **6d**

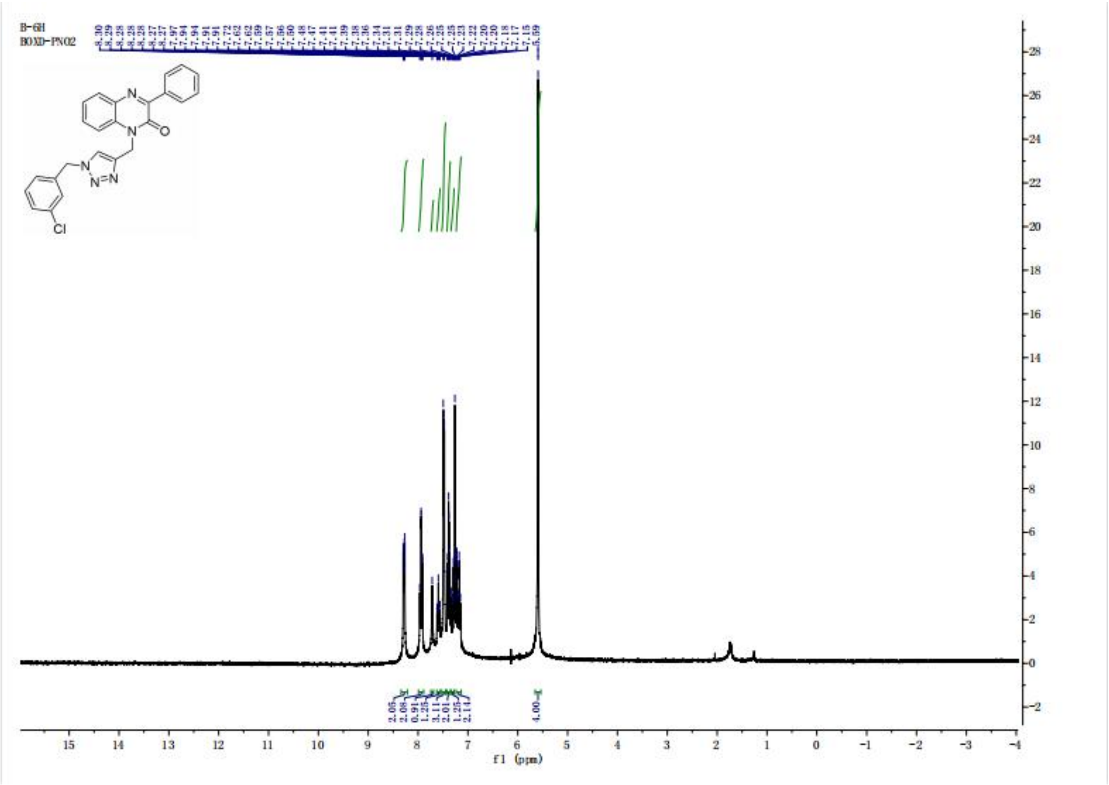

^{1}H-NMR (300 MHz, $CDCl_3$) spectrum of compound **6e**

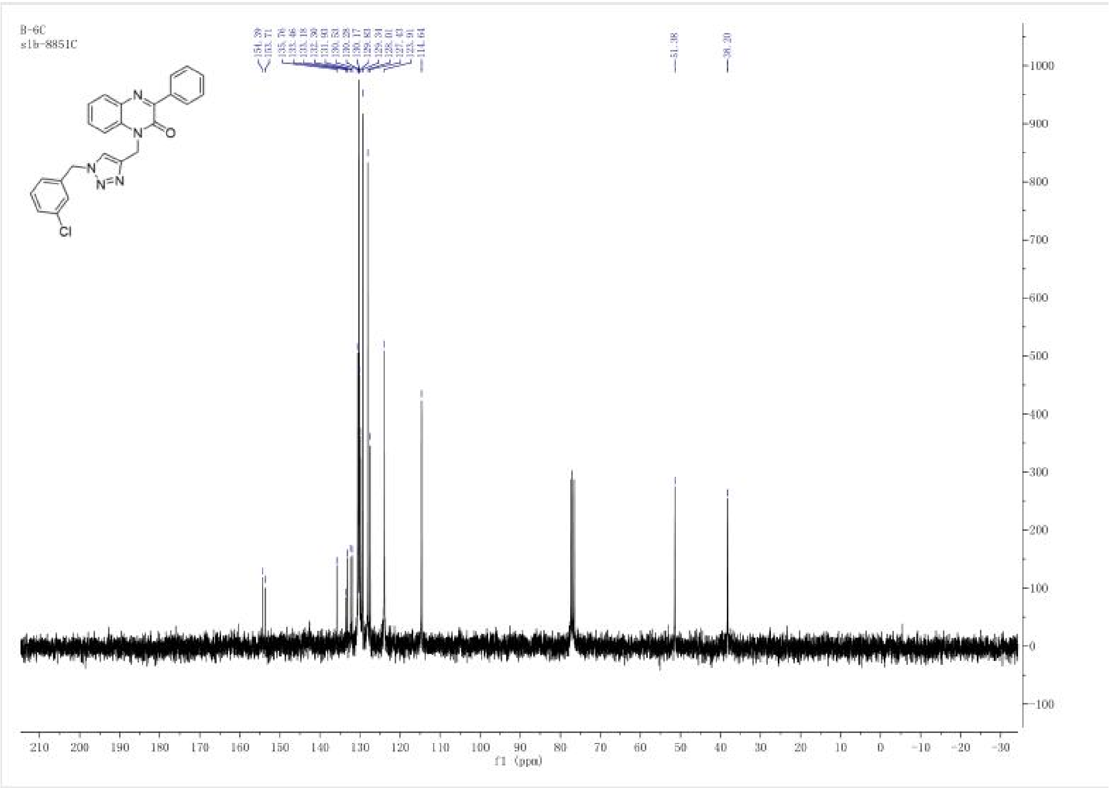

^{13}C NMR (75 MHz, $CDCl_3$) spectrum of compound **6e**

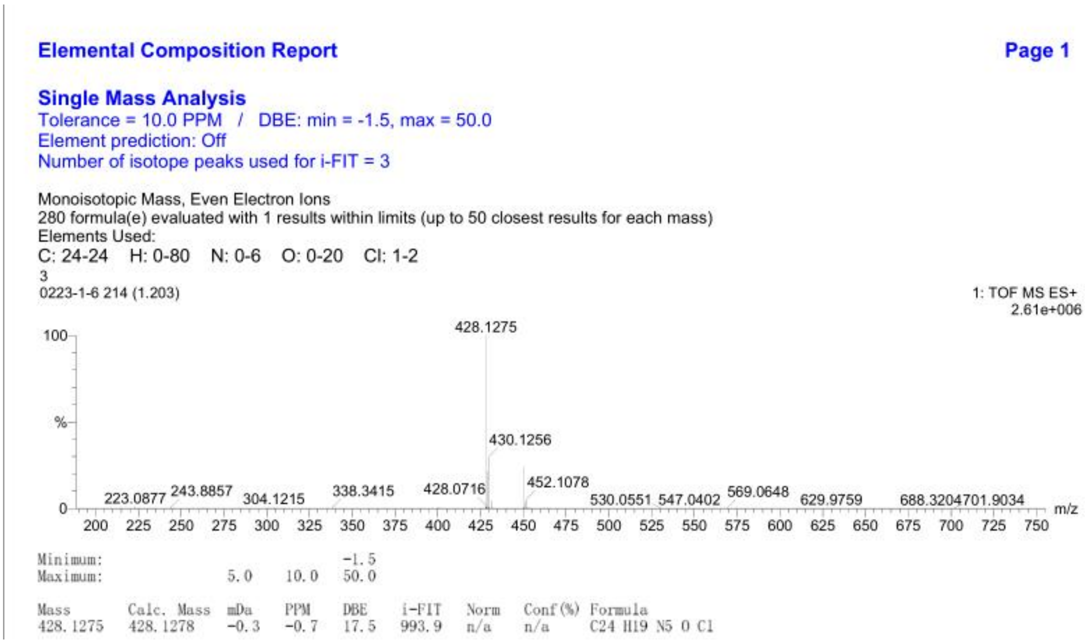

HRMS spectrum of compound **6e**

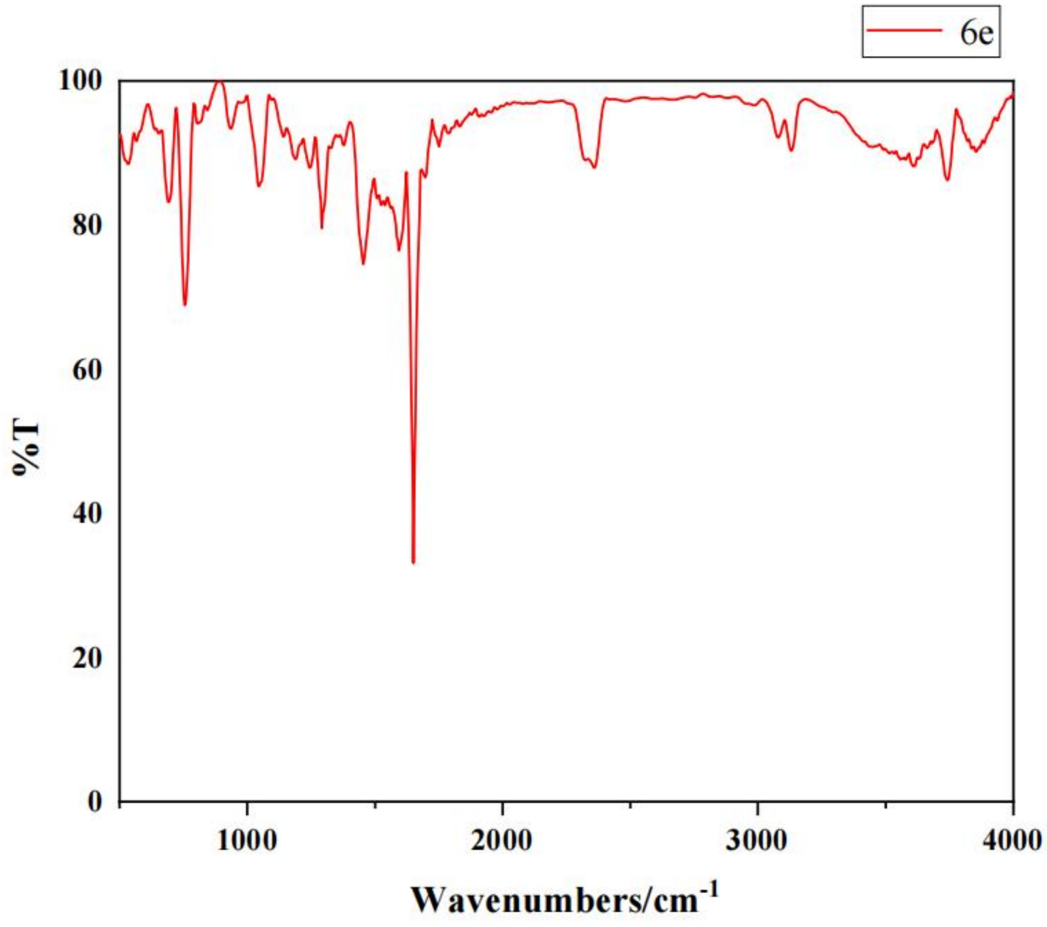

FT-IR spectrum of compound **6e**

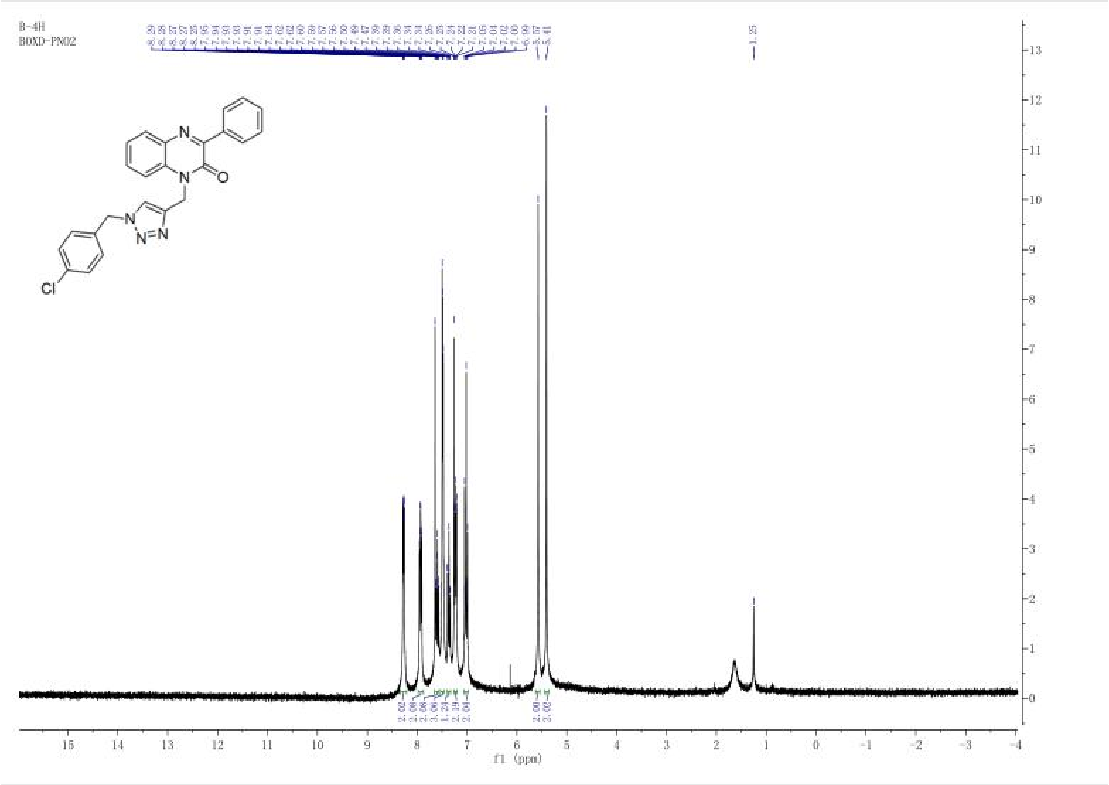

^{1}H-NMR (300 MHz, $CDCl_3$) spectrum of compound **6f**

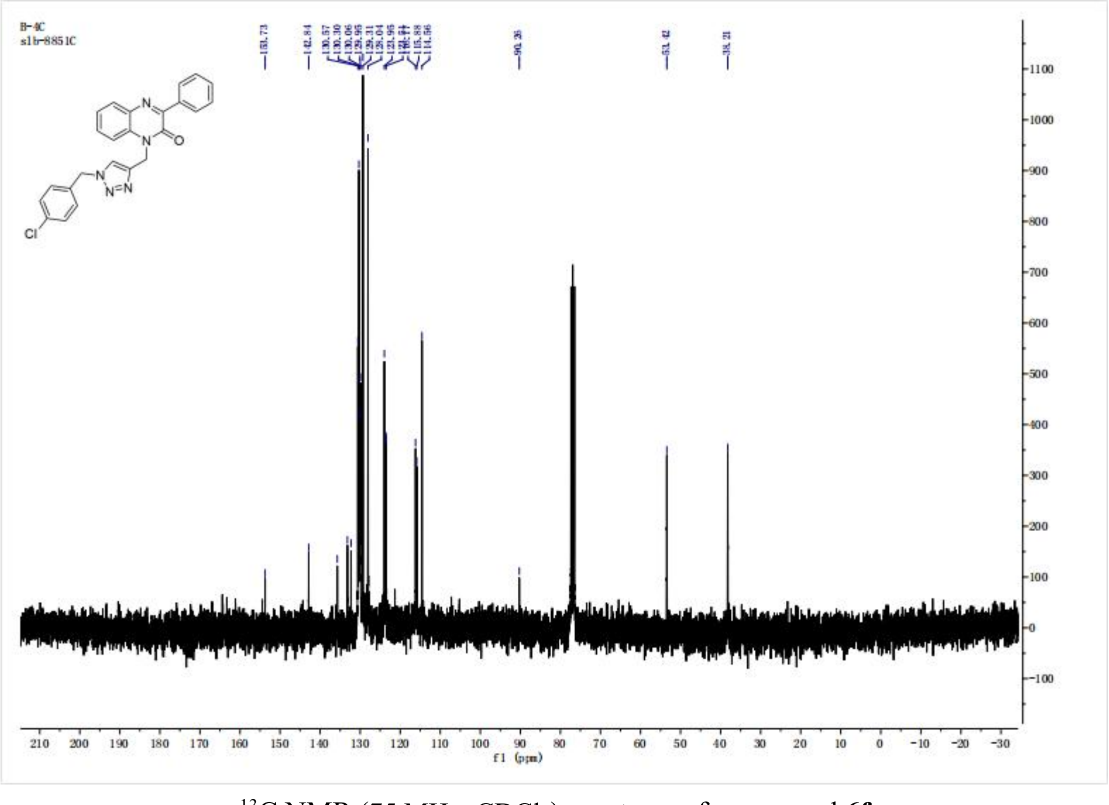

^{13}C NMR (75 MHz, $CDCl_3$) spectrum of compound **6f**

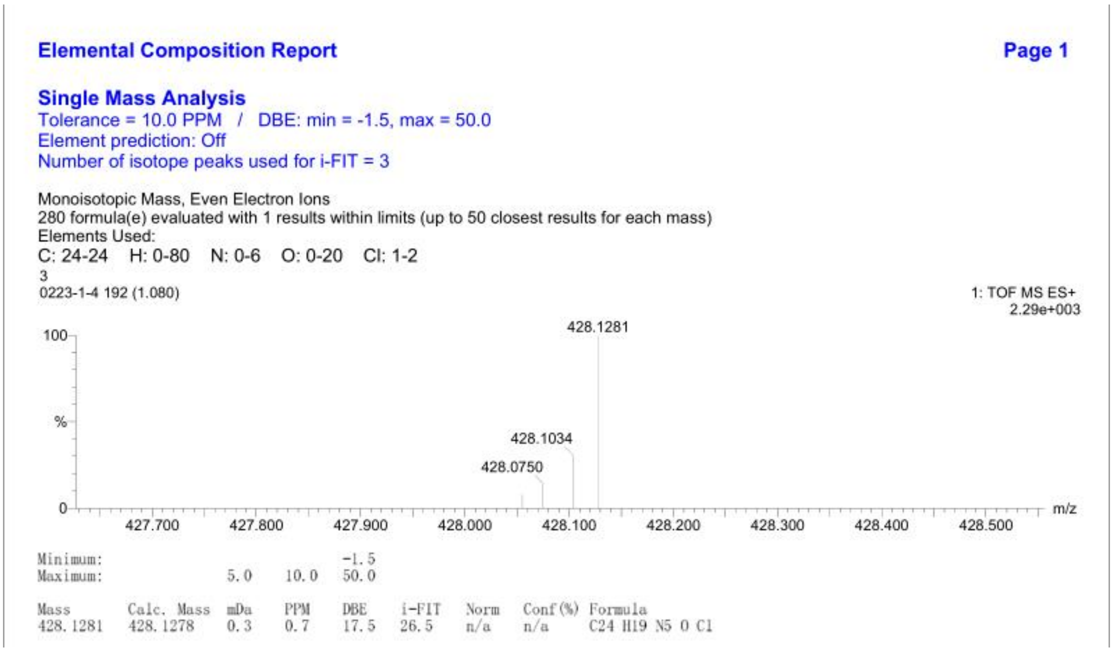

HRMS spectrum of compound **6f**

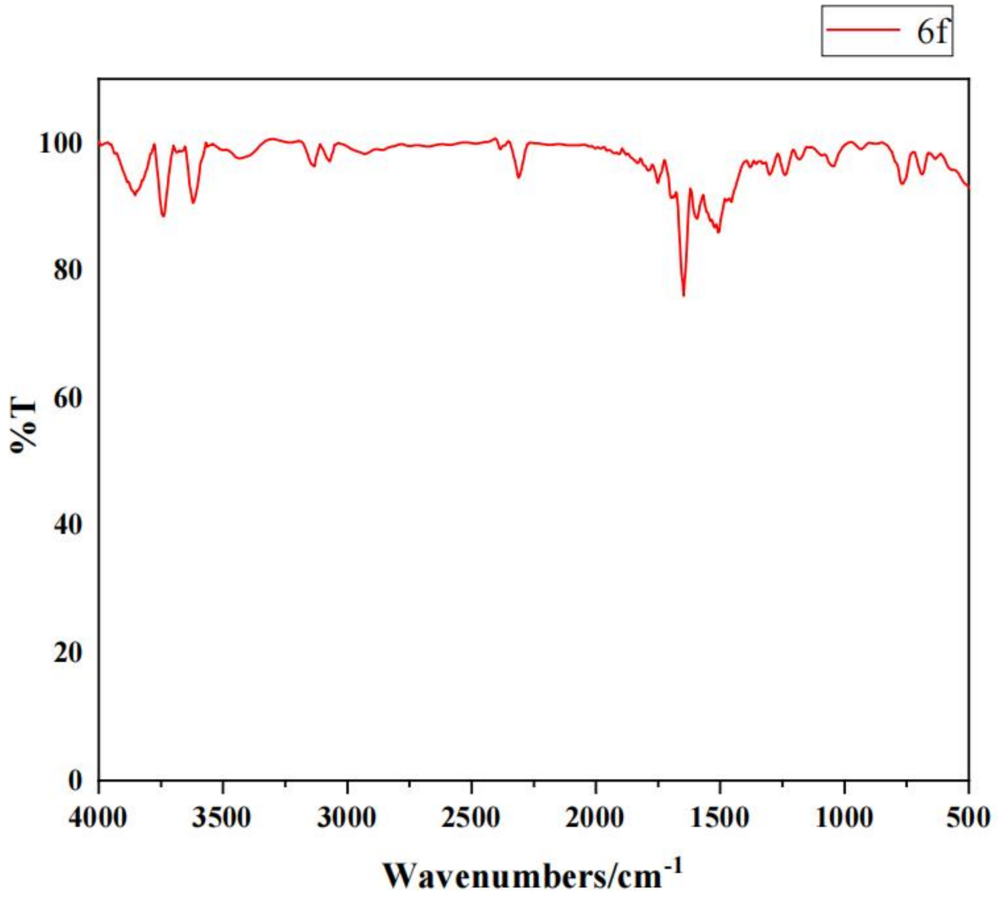

FT-IR spectrum of compound **6f**

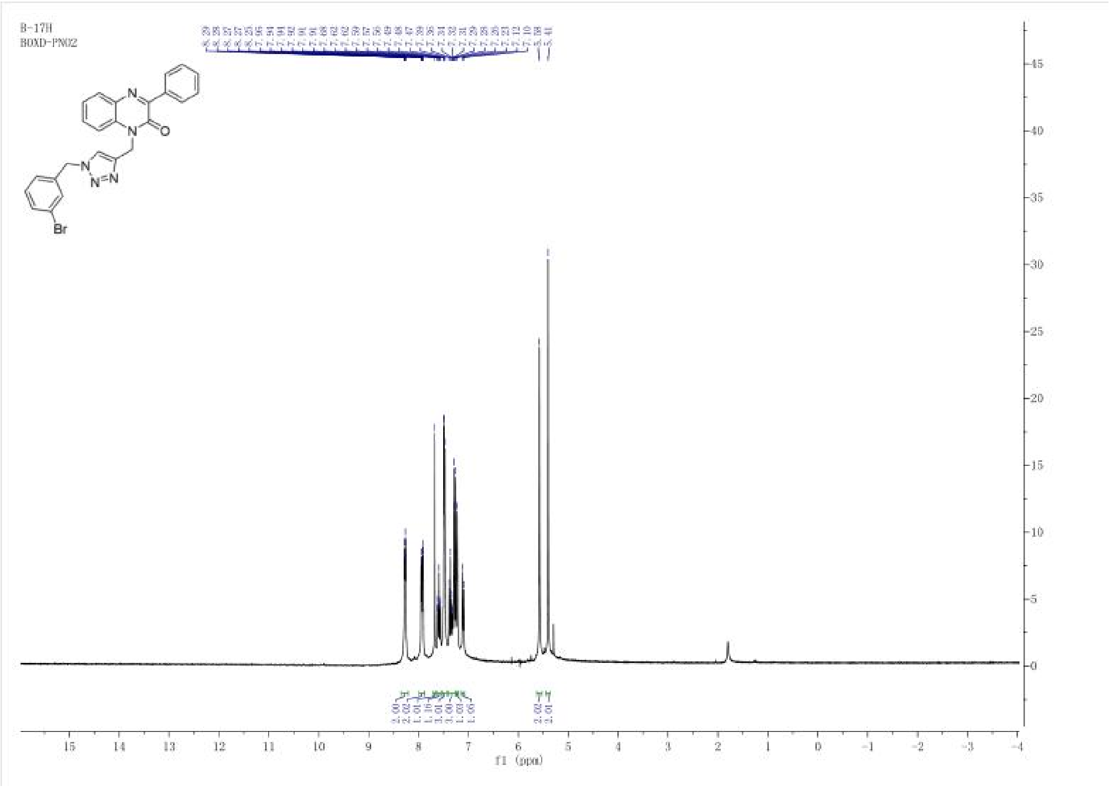

^{1}H-NMR (300 MHz, $CDCl_3$) spectrum of compound **6g**

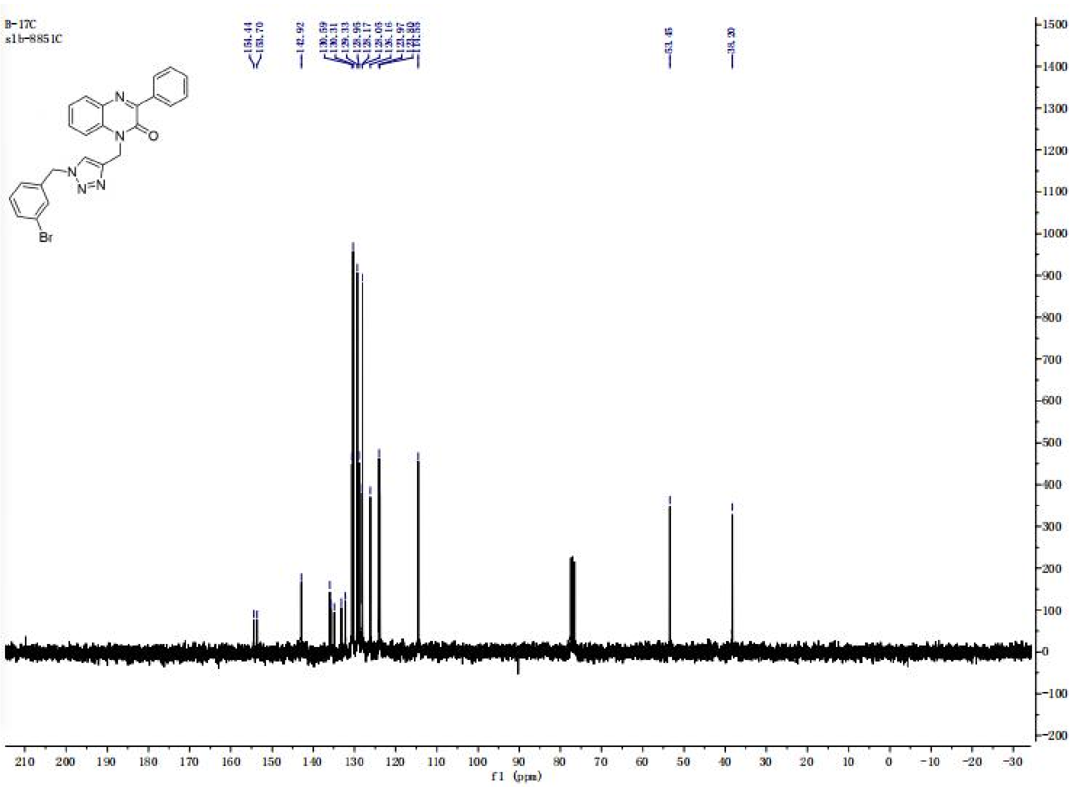

^{13}C NMR (75 MHz, $CDCl_3$) spectrum of compound **6g**

Elemental Composition Report **Page 1**

Single Mass Analysis
Tolerance = 10.0 PPM / DBE: min = -1.5, max = 50.0
Element prediction: Off
Number of isotope peaks used for i-FIT = 3

Monoisotopic Mass, Even Electron Ions
144 formula(e) evaluated with 1 results within limits (up to 50 closest results for each mass)
Elements Used:
C: 24-24 H: 0-80 N: 0-6 O: 0-20 Br: 1-1

3
0223-1-17 192 (1.080) 1: TOF MS ES+
1.21e+002

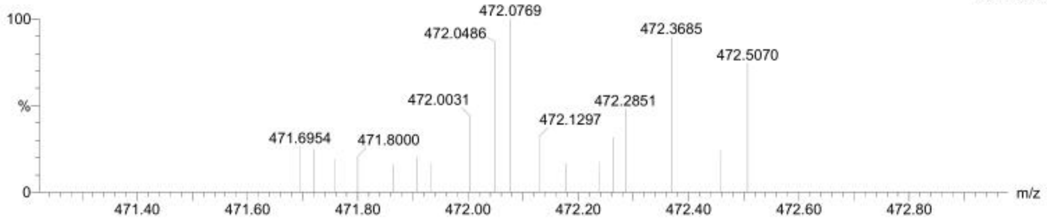

Minimum:				-1.5					
Maximum:		5.0	10.0	50.0					
Mass	Calc. Mass	mDa	PPM	DBE	i-FIT	Norm	Conf(%)	Formula	
472.0769	472.0773	-0.4	-0.8	17.5	80.2	n/a	n/a	C24 H19 N5 O Br	

HRMS spectrum of compound **6g**

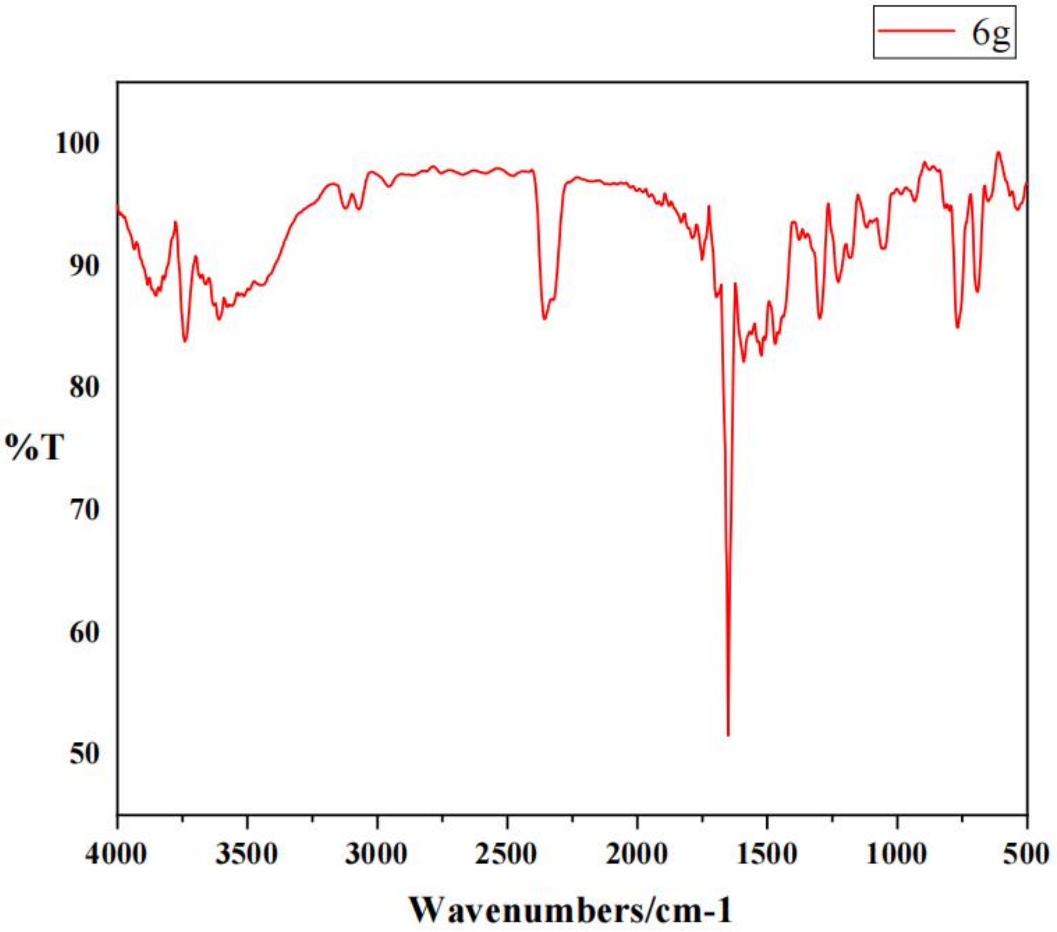

FT-IR spectrum of compound **6g**

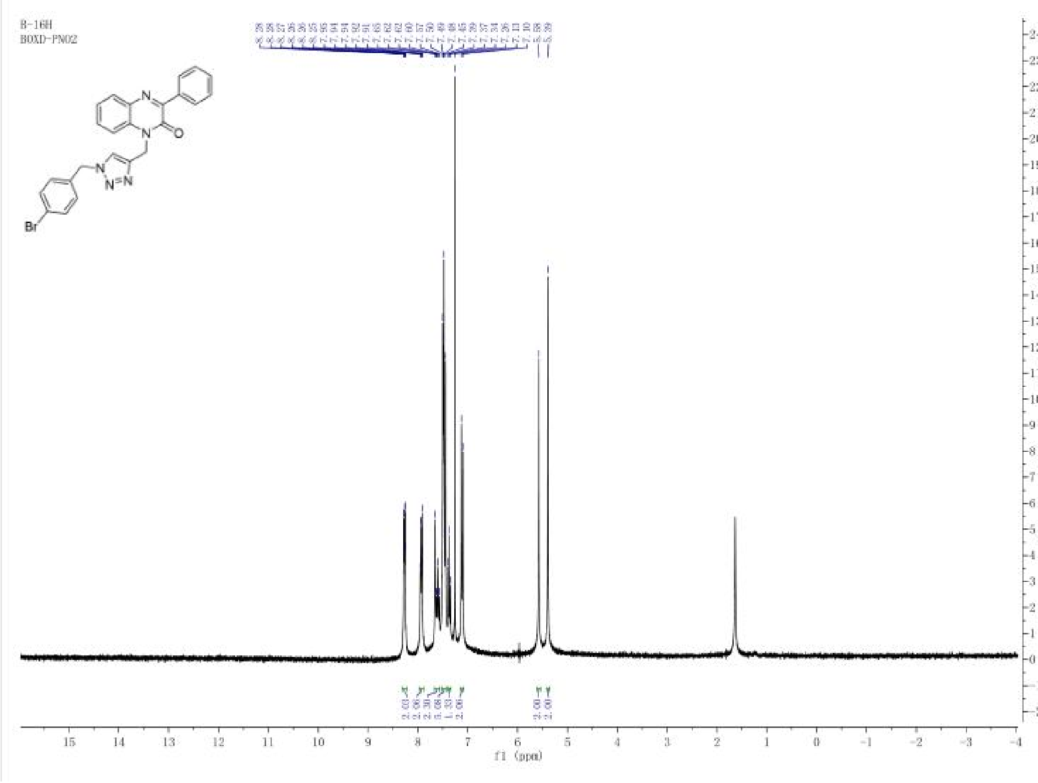

^{1}H-NMR (300 MHz, $CDCl_3$) spectrum of compound **6h**

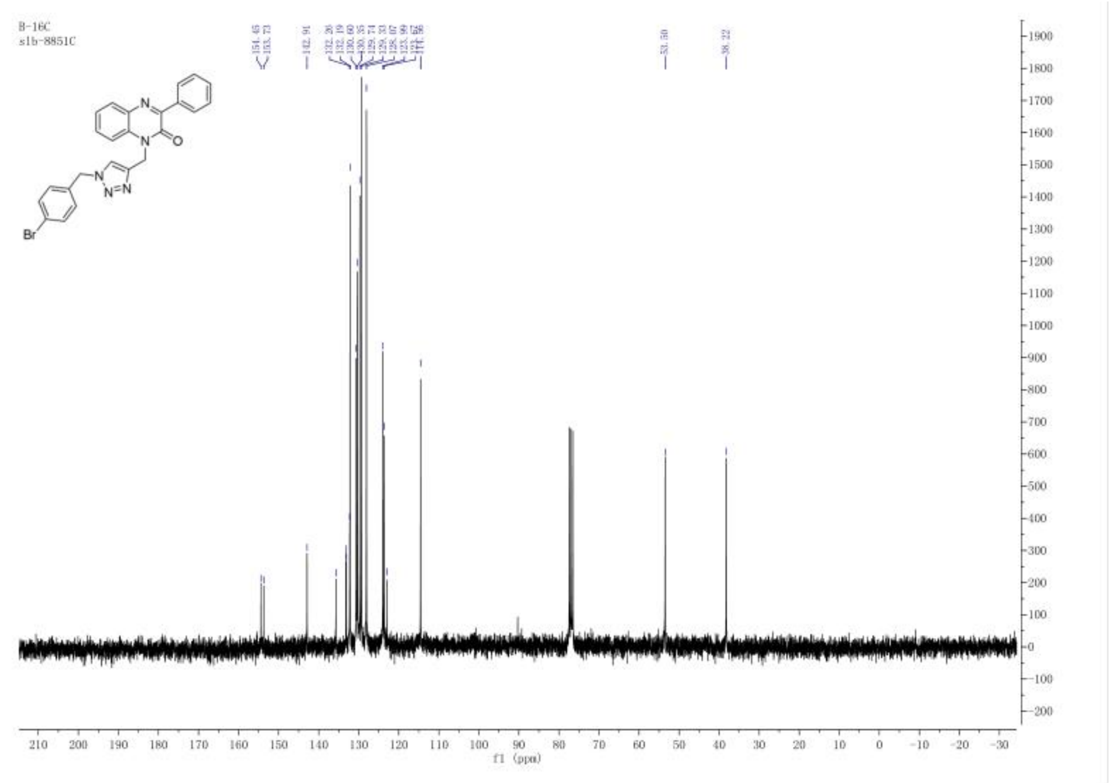

^{13}C NMR (75 MHz, $CDCl_3$) spectrum of compound **6h**

Elemental Composition Report **Page 1**

Single Mass Analysis
Tolerance = 10.0 PPM / DBE: min = -1.5, max = 50.0
Element prediction: Off
Number of isotope peaks used for i-FIT = 3

Monoisotopic Mass, Even Electron Ions
144 formula(e) evaluated with 1 results within limits (up to 50 closest results for each mass)
Elements Used:
C: 24-24 H: 0-80 N: 0-6 O: 0-20 Br: 1-1

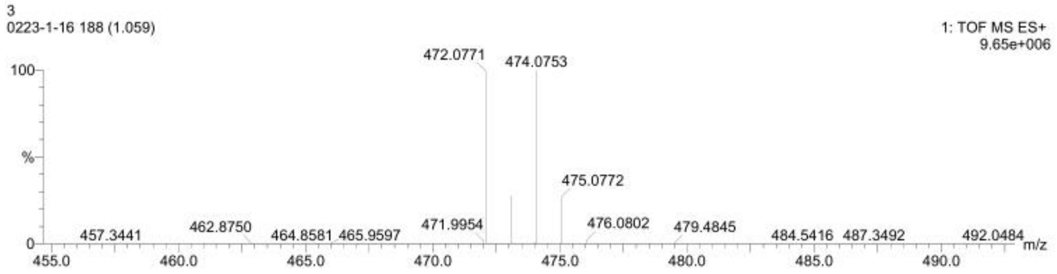

Minimum:				-1.5				
Maximum:		5.0	10.0	50.0				
Mass	Calc. Mass	mDa	PPM	DBE	i-FIT	Norm	Conf(%)	Formula
472.0771	472.0773	-0.2	-0.4	17.5	1092.9	n/a	n/a	C24 H19 N5 O Br

HRMS spectrum of compound **6h**

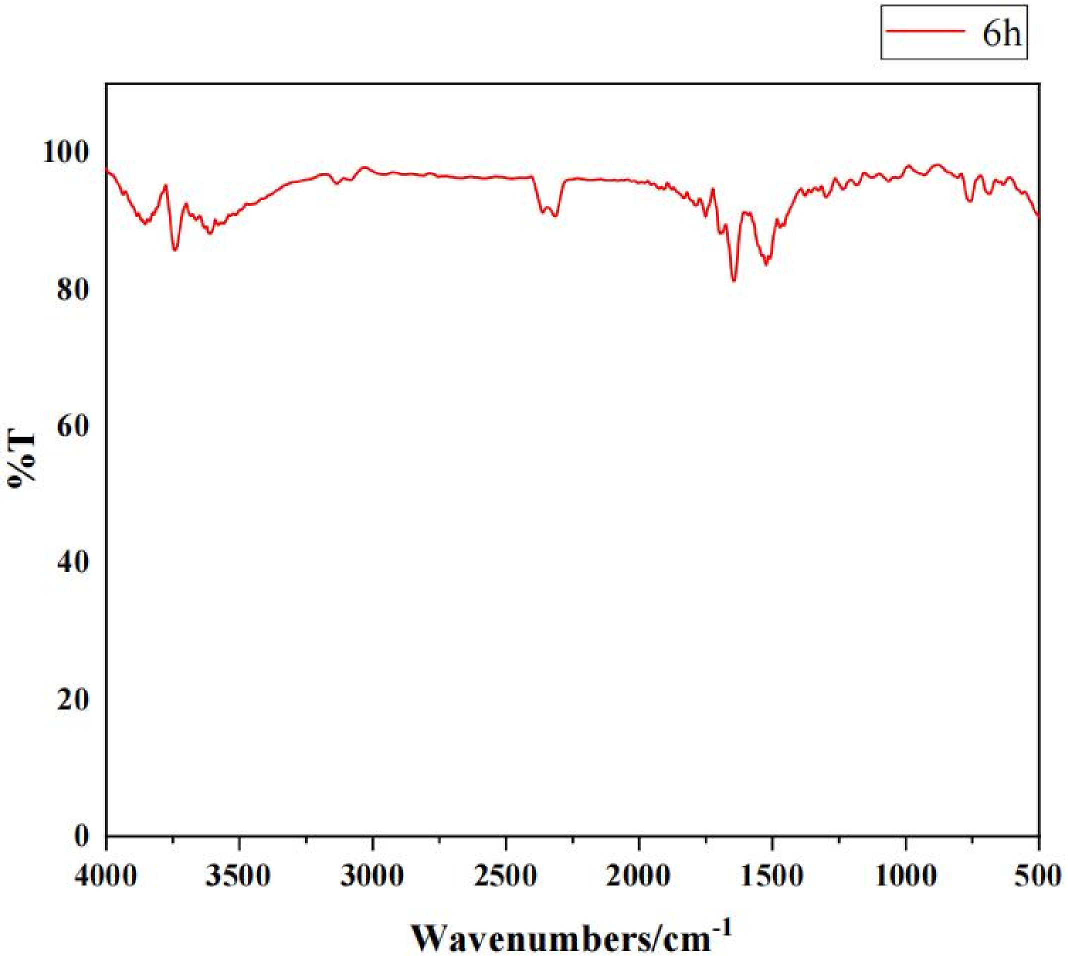

FT-IR spectrum of compound **6h**

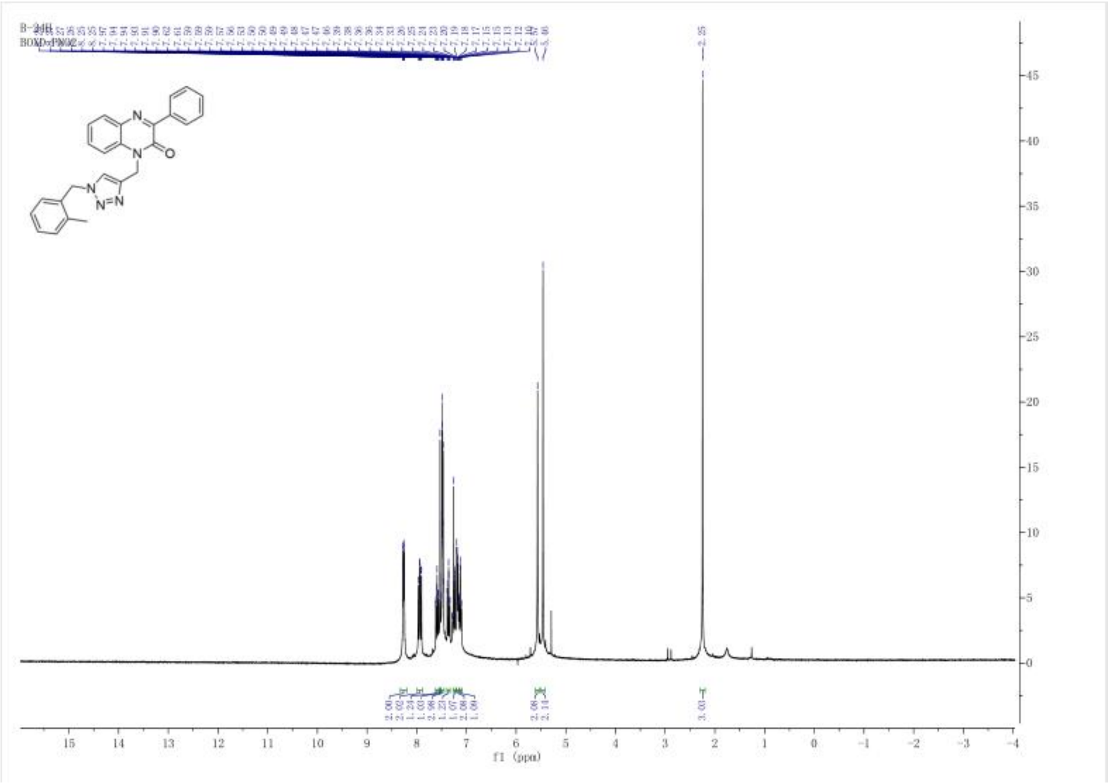

^{1}H-NMR (300 MHz, $CDCl_3$) spectrum of compound **6i**

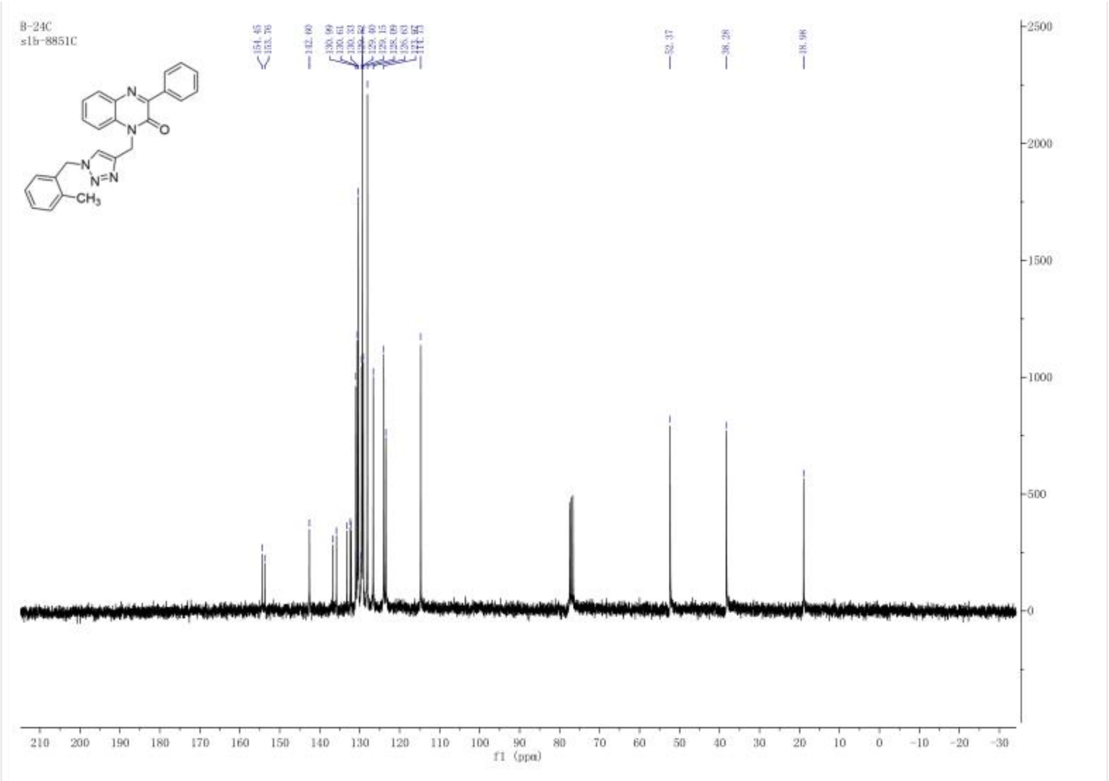

^{13}C NMR (75 MHz, $CDCl_3$) spectrum of compound **6i**

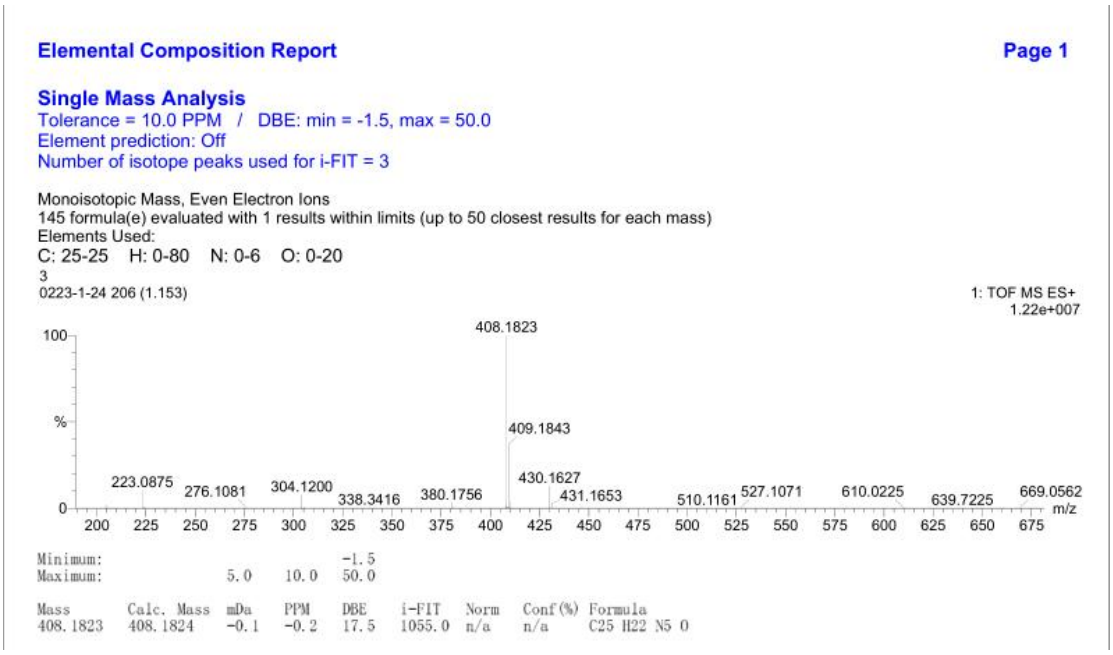

HRMS spectrum of compound **6i**

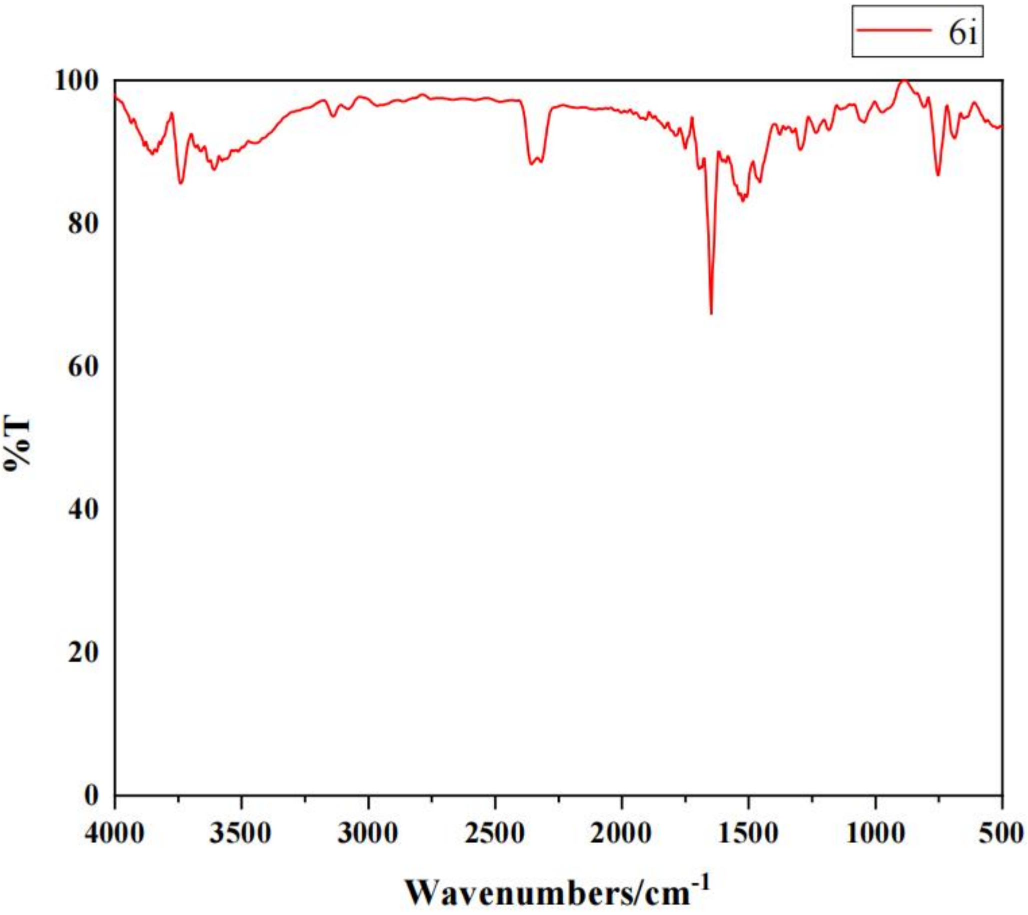

FT-IR spectrum of compound **6i**

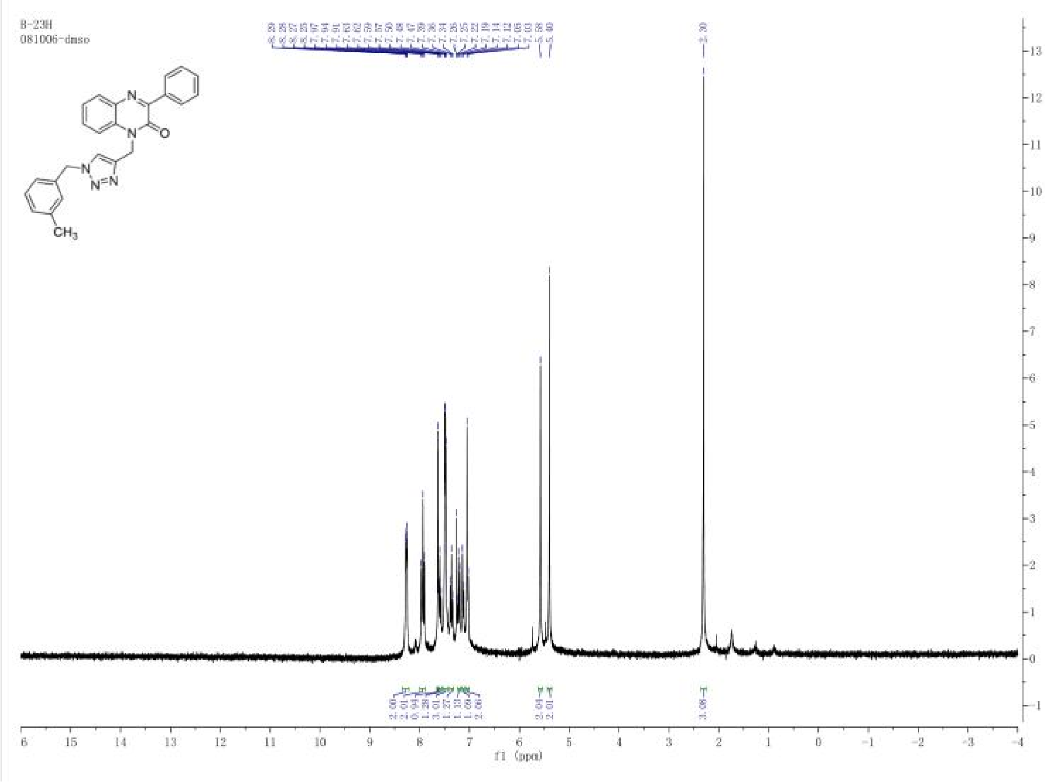

^{1}H-NMR (300 MHz, $CDCl_3$) spectrum of compound **6j**

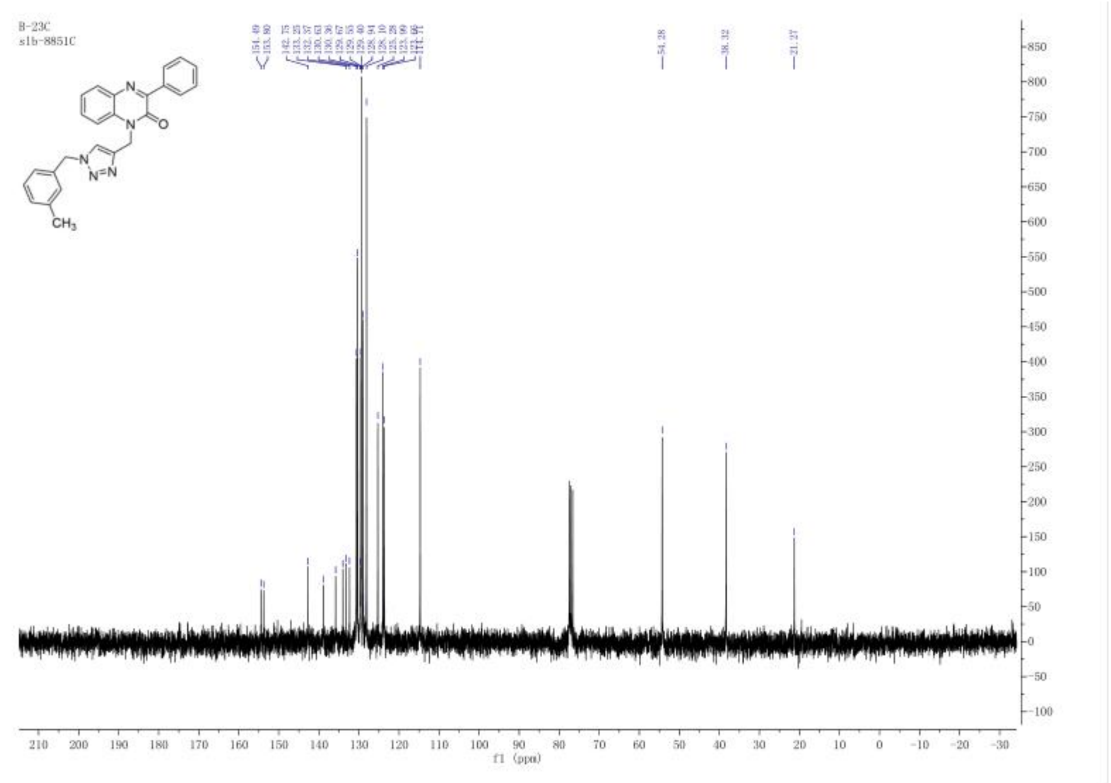

^{13}C NMR (75 MHz, $CDCl_3$) spectrum of compound **6j**

Elemental Composition Report **Page 1**

Single Mass Analysis
Tolerance = 10.0 PPM / DBE: min = -1.5, max = 50.0
Element prediction: Off
Number of isotope peaks used for i-FIT = 3

Monoisotopic Mass, Even Electron Ions
145 formula(e) evaluated with 1 results within limits (up to 50 closest results for each mass)
Elements Used:
C: 25-25 H: 0-80 N: 0-6 O: 0-20
3
0223-1-23 194 (1.090) 1: TOF MS ES+ 1.38e+007

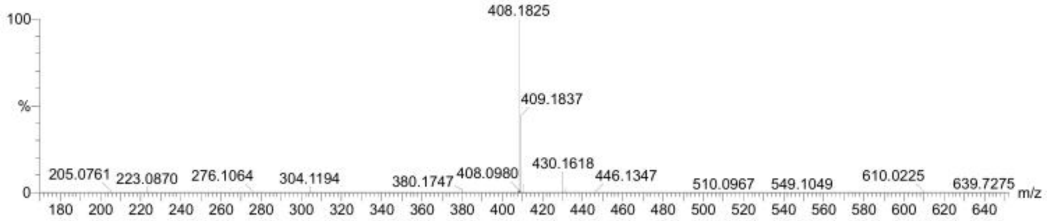

Minimum:				-1.5				
Maximum:		5.0	10.0	50.0				
Mass	Calc. Mass	mDa	PPM	DBE	i-FIT	Norm	Conf(%)	Formula
408.1825	408.1824	0.1	0.2	17.5	1150.0	n/a	n/a	C25 H22 N5 O

HRMS spectrum of compound **6j**

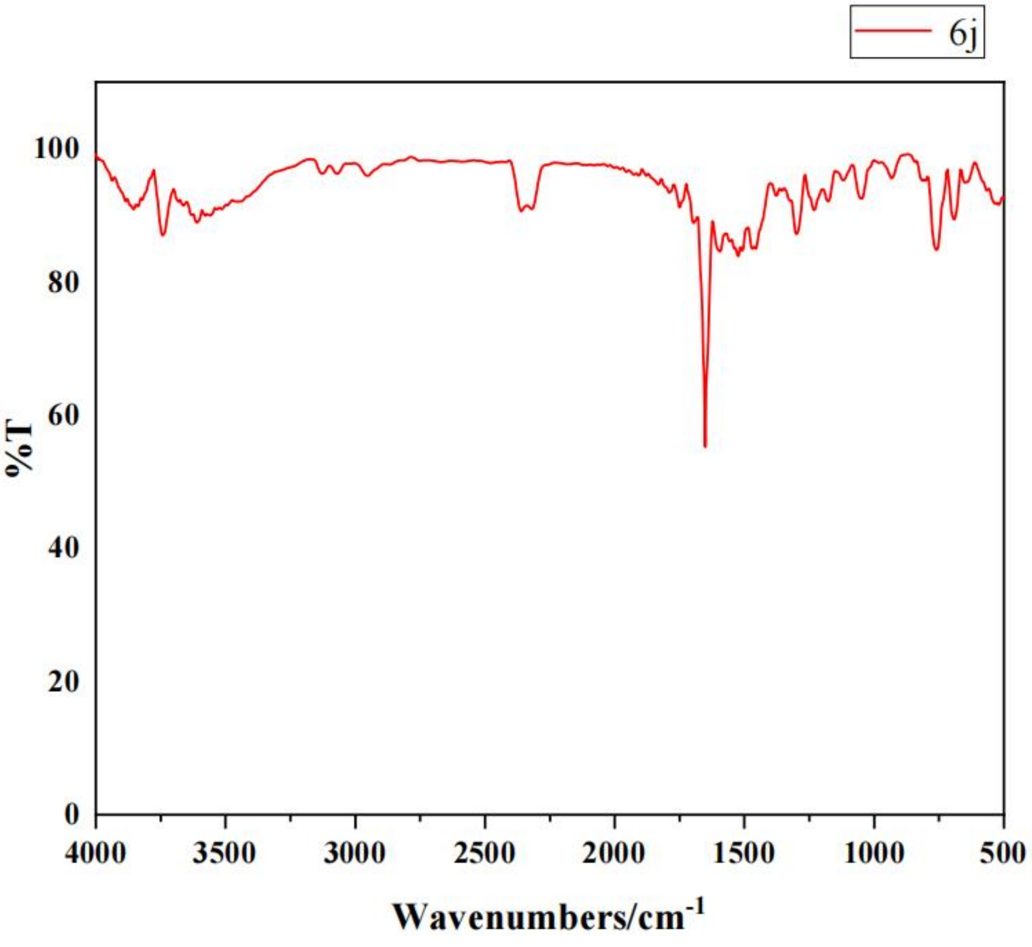

FT-IR spectrum of compound **6j**

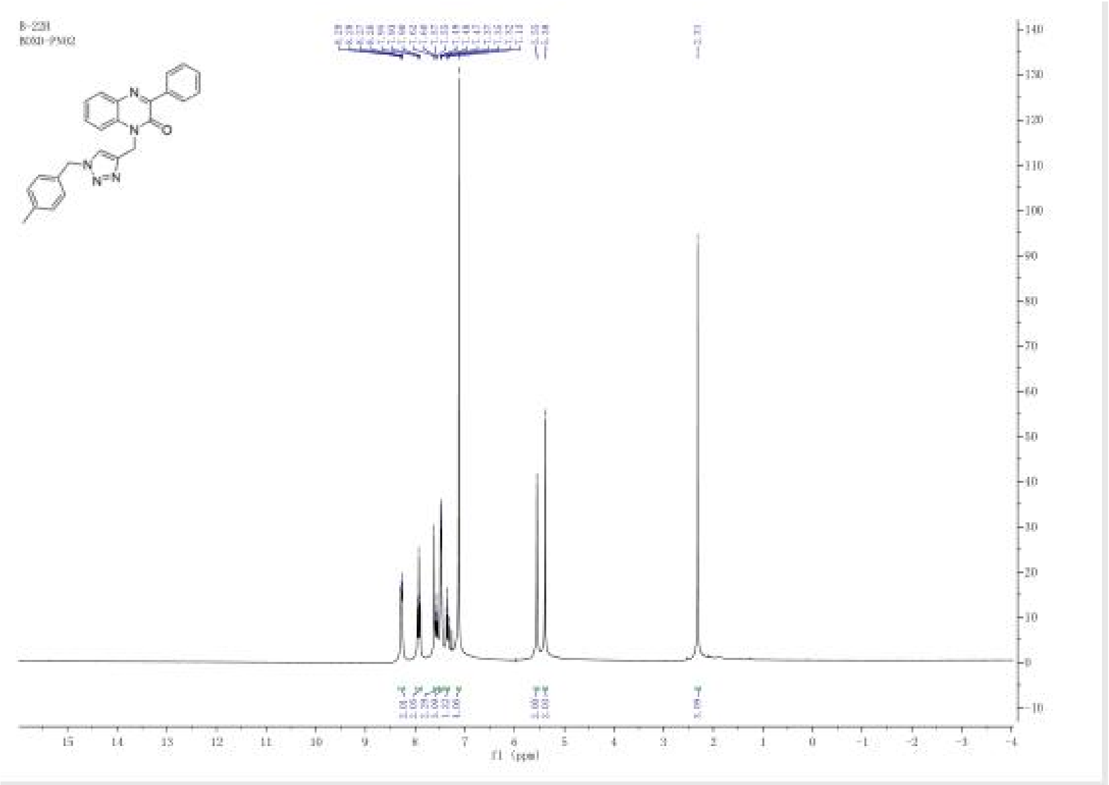

^{1}H-NMR (300 MHz, CDCl$_3$) spectrum of compound **6k**

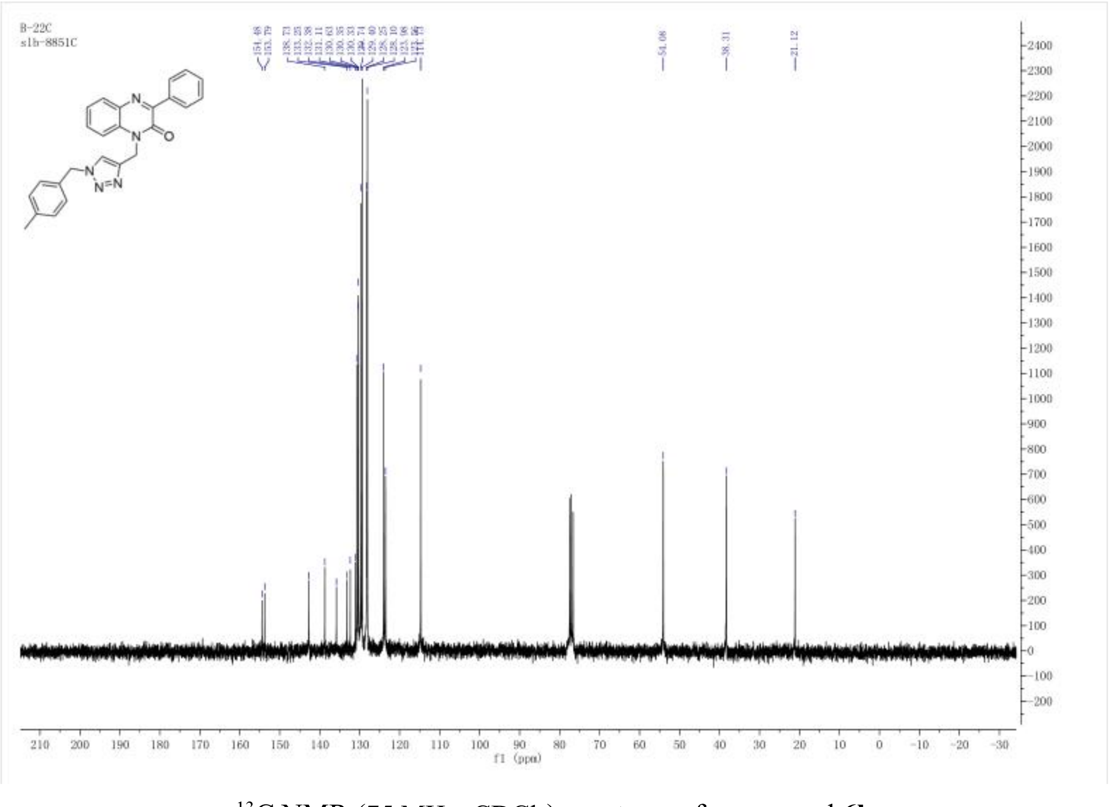

^{13}C NMR (75 MHz, CDCl$_3$) spectrum of compound **6k**

Elemental Composition Report **Page 1**

Single Mass Analysis
Tolerance = 10.0 PPM / DBE: min = -1.5, max = 50.0
Element prediction: Off
Number of isotope peaks used for i-FIT = 3

Monoisotopic Mass, Even Electron Ions
145 formula(e) evaluated with 1 results within limits (up to 50 closest results for each mass)
Elements Used:
C: 25-25 H: 0-80 N: 0-6 O: 0-20
3
0223-1-22 201 (1.127) 1: TOF MS ES+
7.29e+006

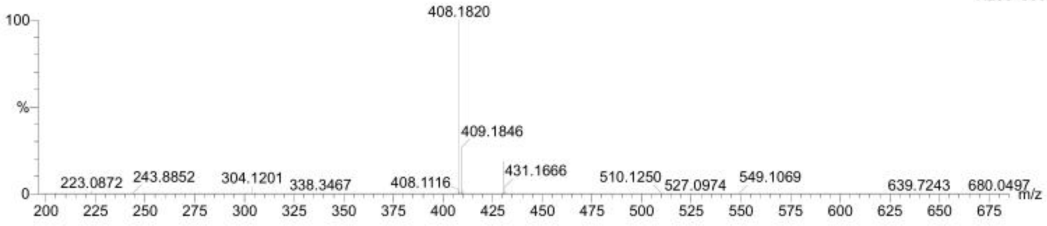

Minimum:				-1.5				
Maximum:		5.0	10.0	50.0				
Mass	Calc. Mass	mDa	PPM	DBE	i-FIT	Norm	Conf(%)	Formula
408.1820	408.1824	-0.4	-1.0	17.5	1140.8	n/a	n/a	C25 H22 N5 O

HRMS spectrum of compound **6k**

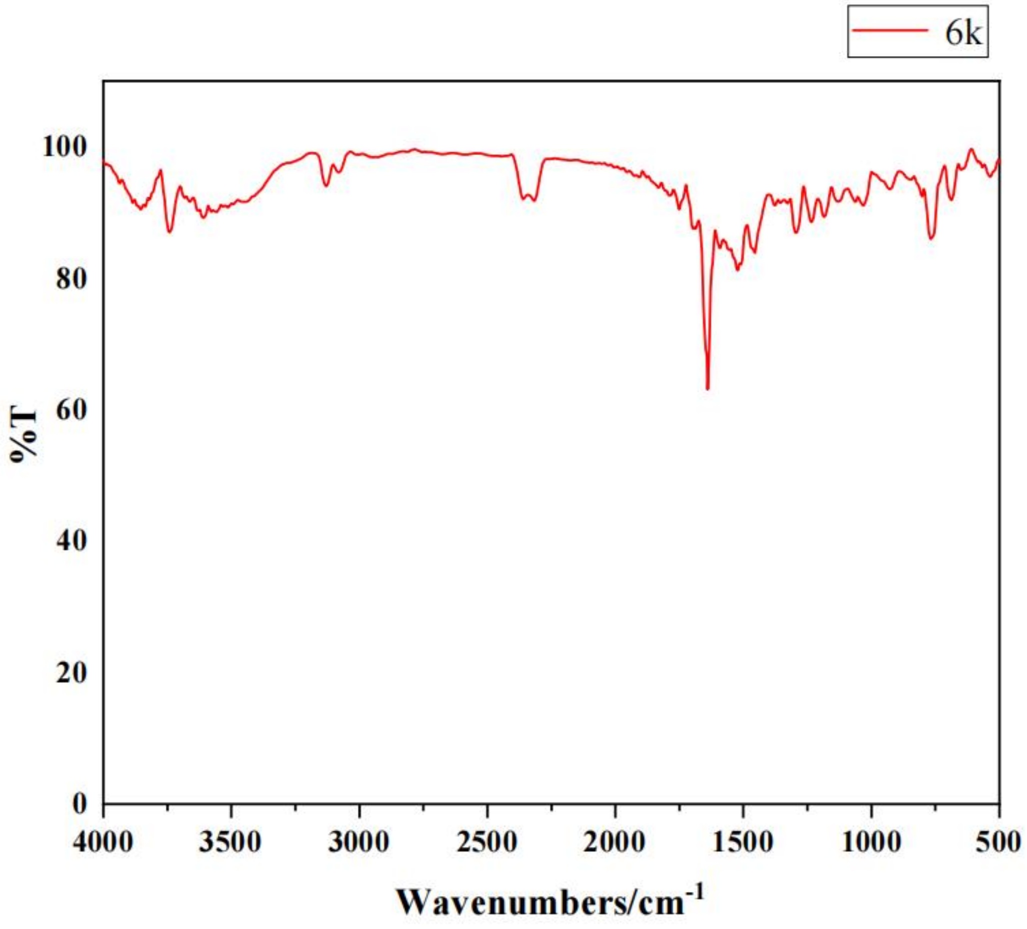

FT-IR spectrum of compound **6k**

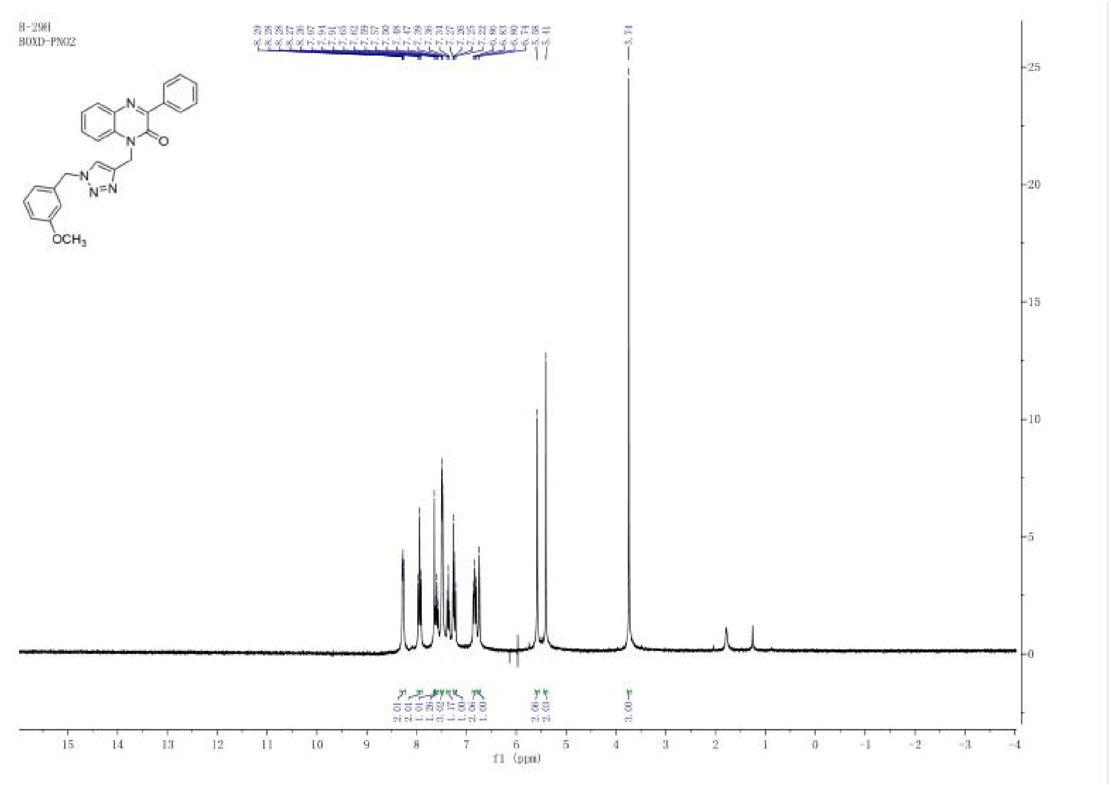

^{1}H-NMR (300 MHz, $CDCl_3$) spectrum of compound **6l**

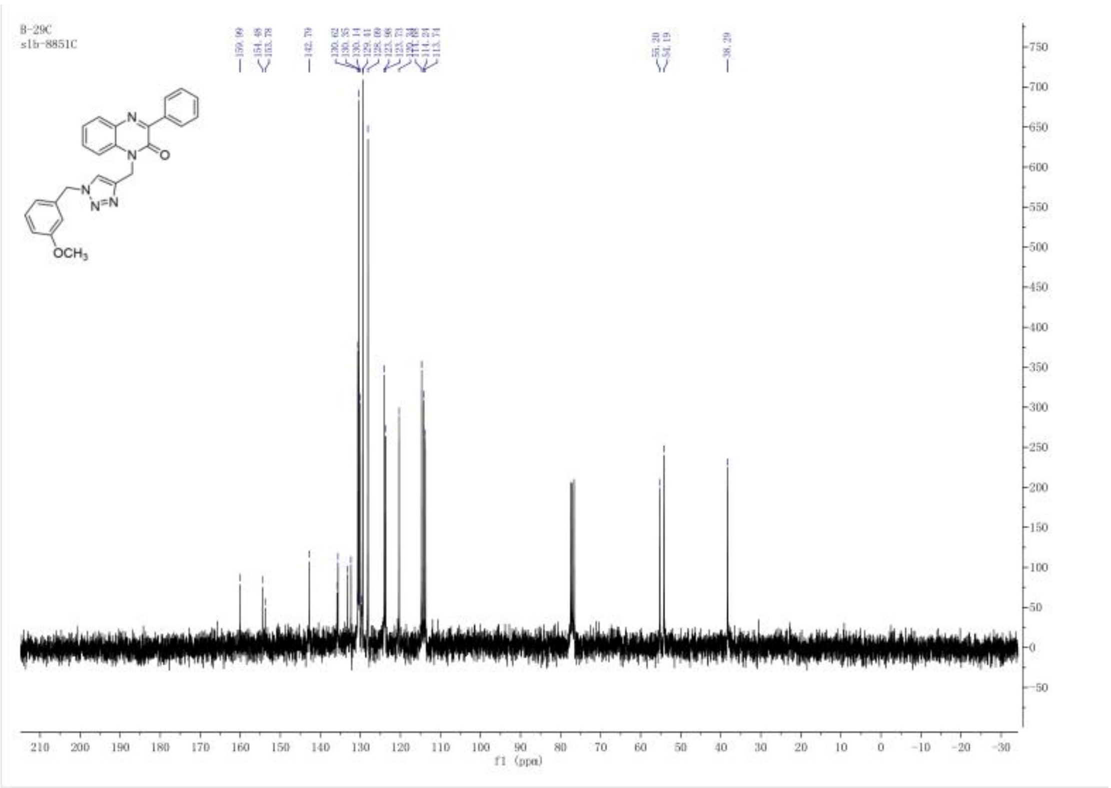

^{13}C NMR (75 MHz, $CDCl_3$) spectrum of compound **6l**

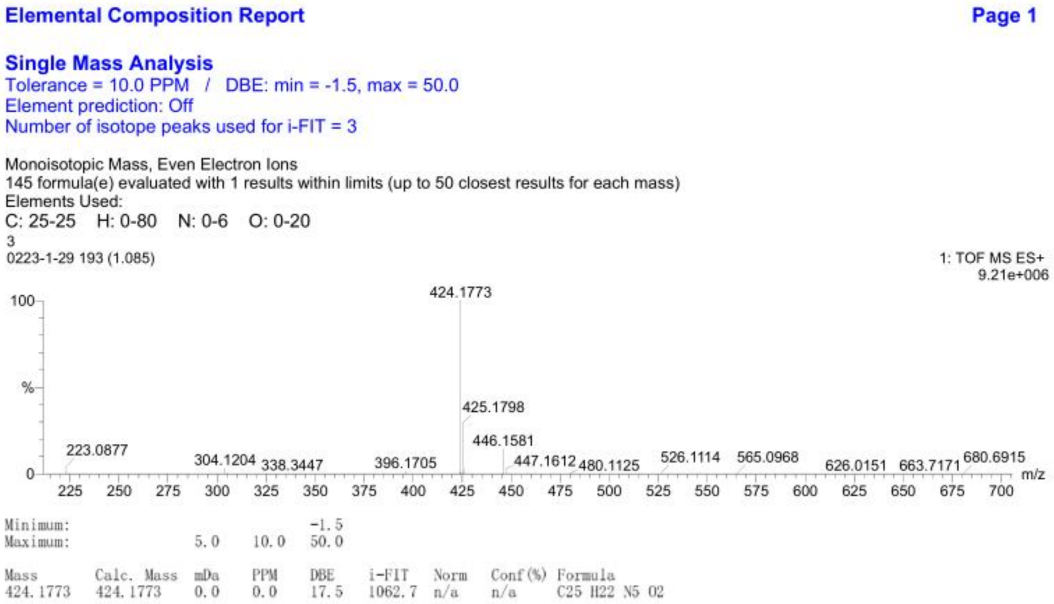

HRMS spectrum of compound **6l**

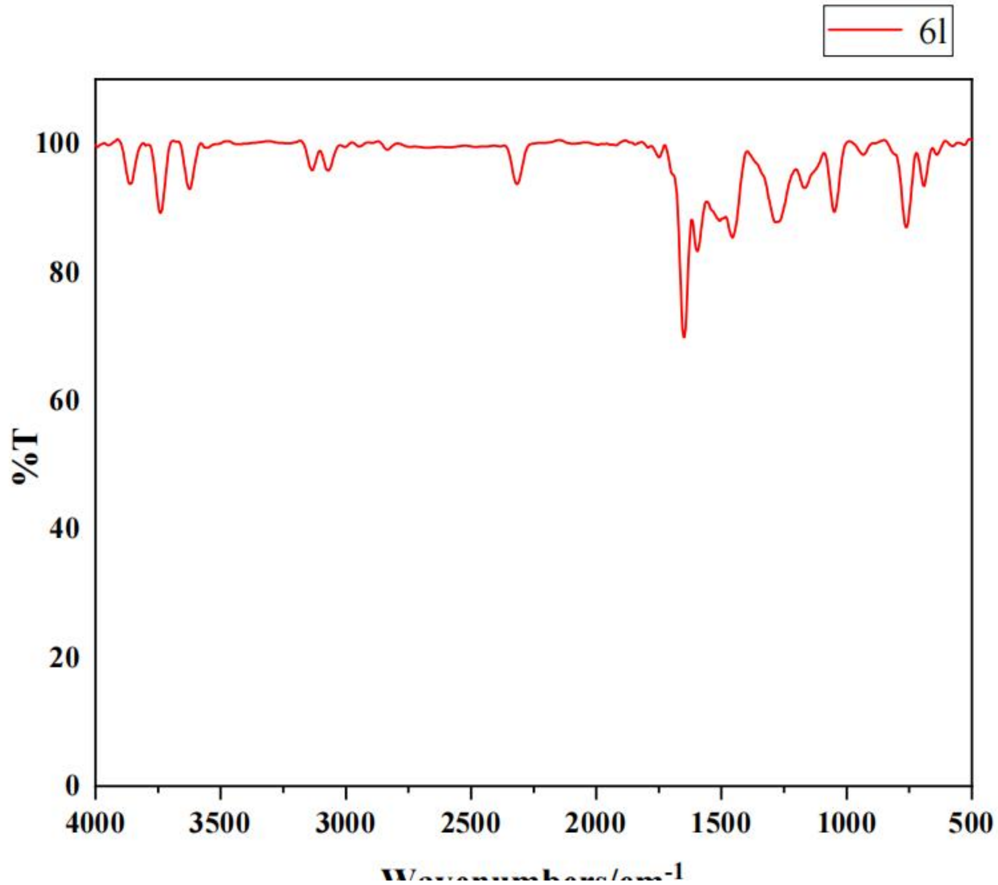

FT-IR spectrum of compound **6l**

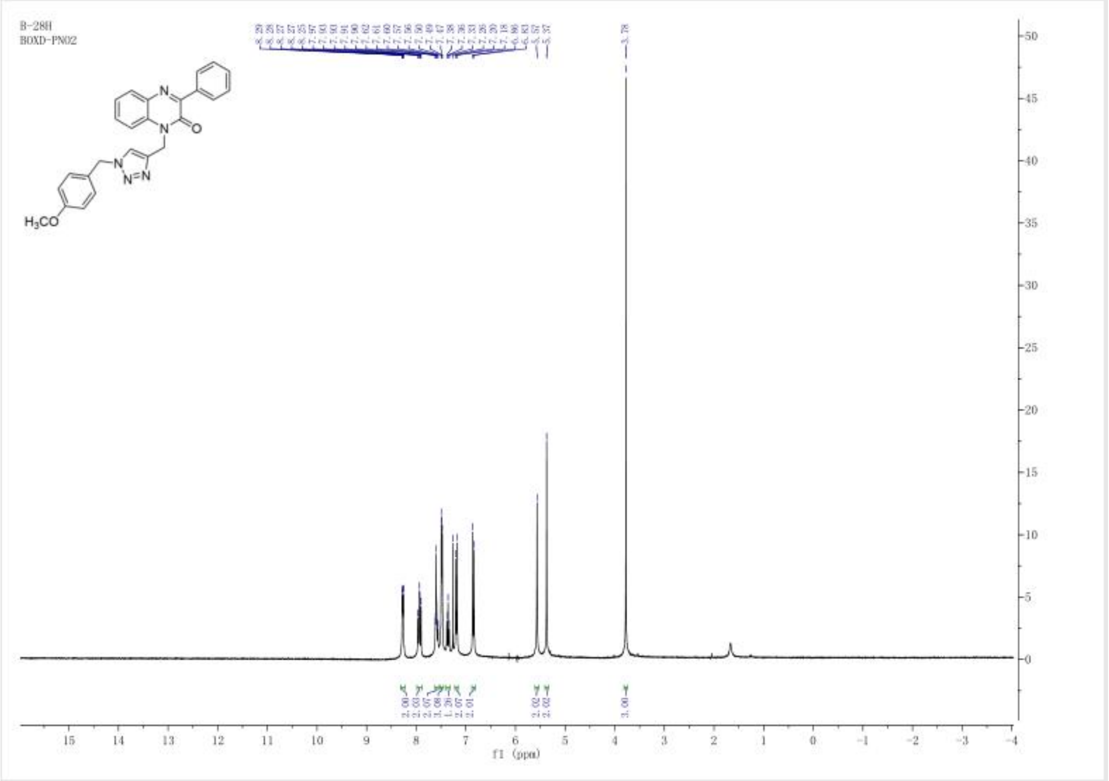

^{1}H-NMR (300 MHz, $CDCl_3$) spectrum of compound **6m**

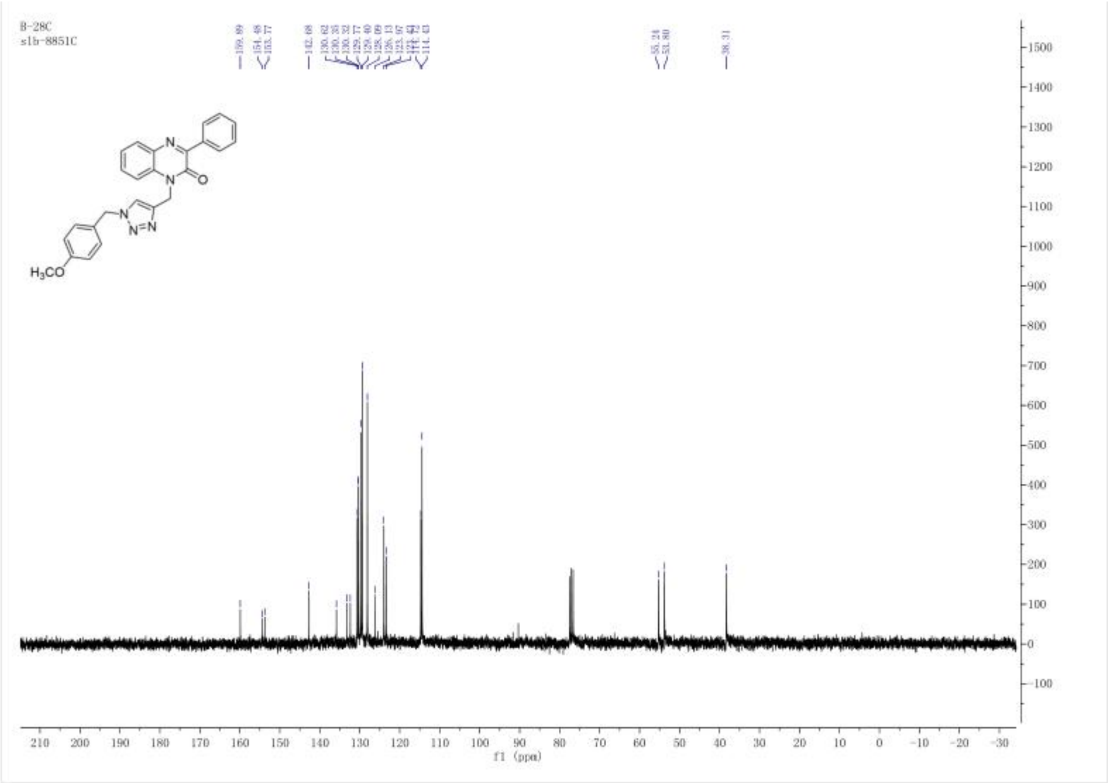

^{13}C NMR (75 MHz, $CDCl_3$) spectrum of compound **6m**

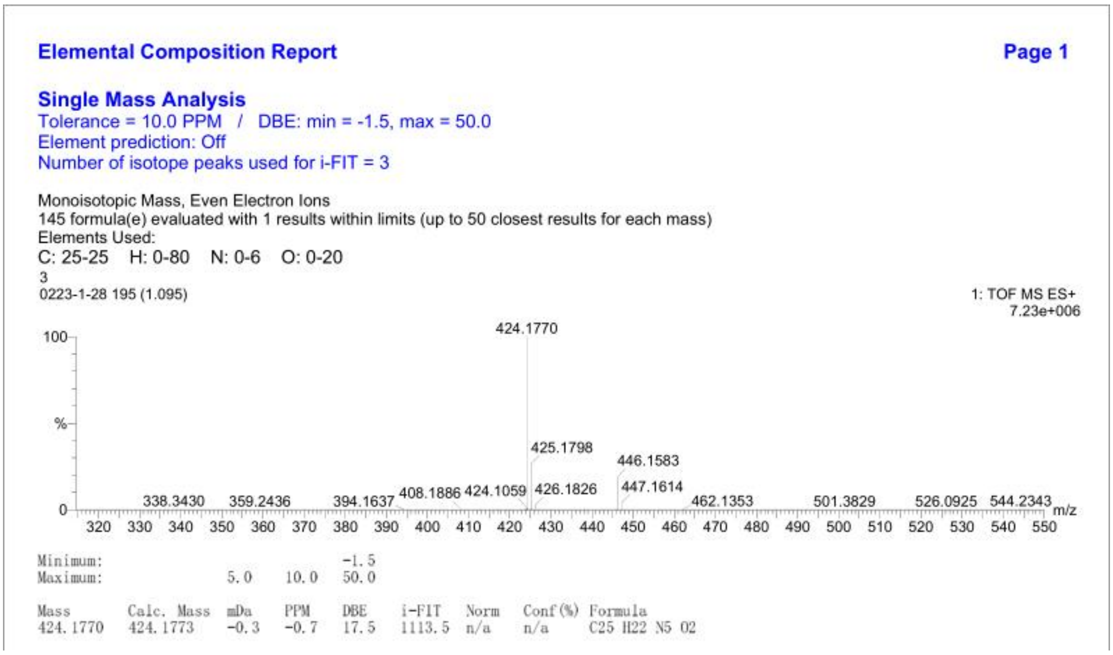

HRMS spectrum of compound **6m**

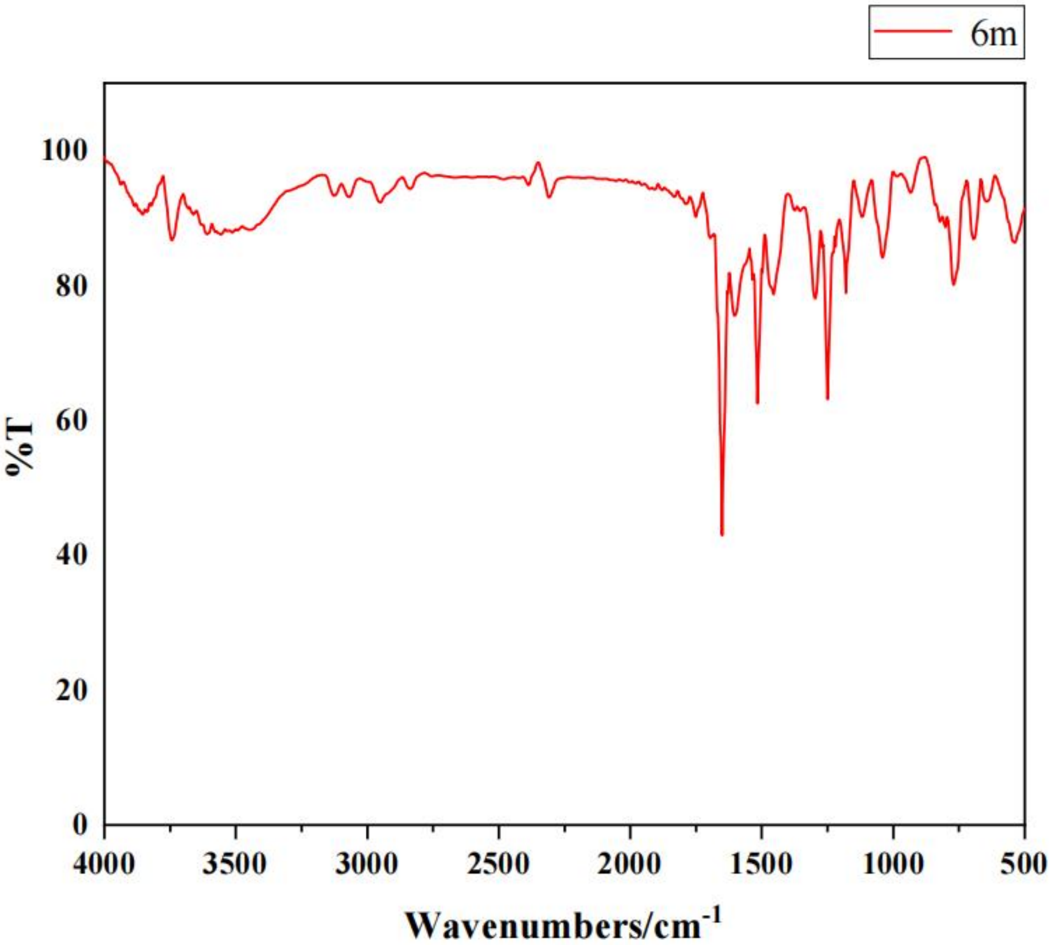

FT-IR spectrum of compound **6m**

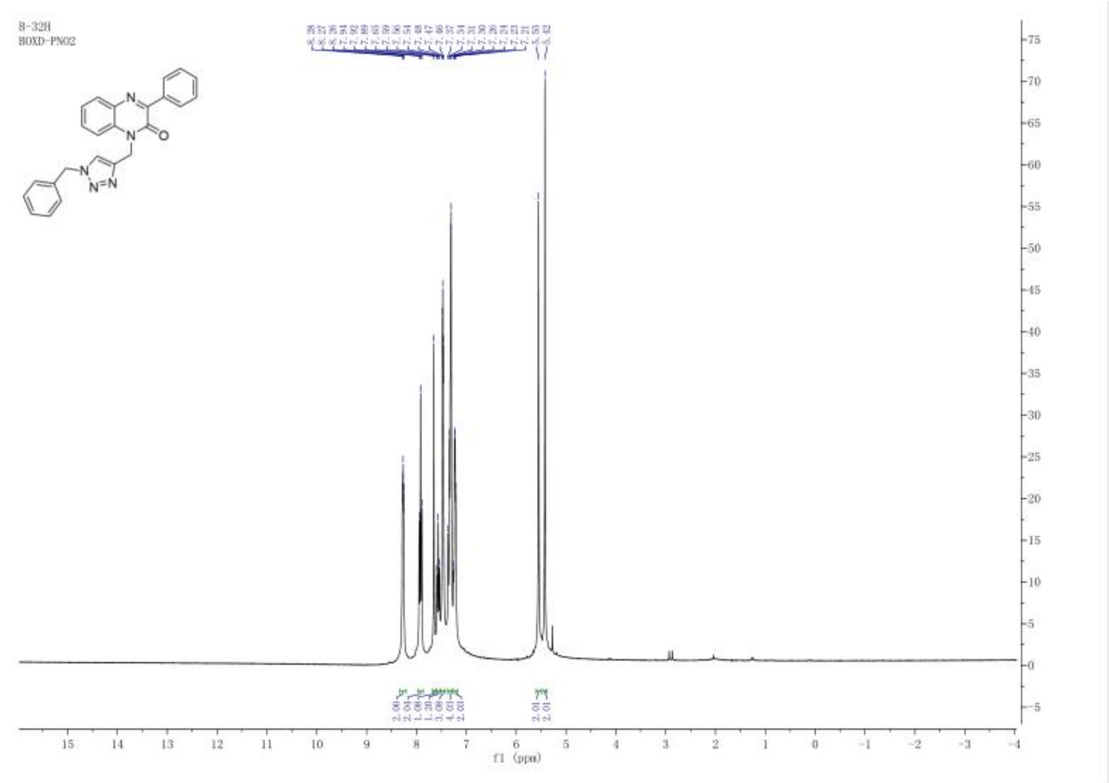

^{1}H-NMR (300 MHz, $CDCl_3$) spectrum of compound **6n**

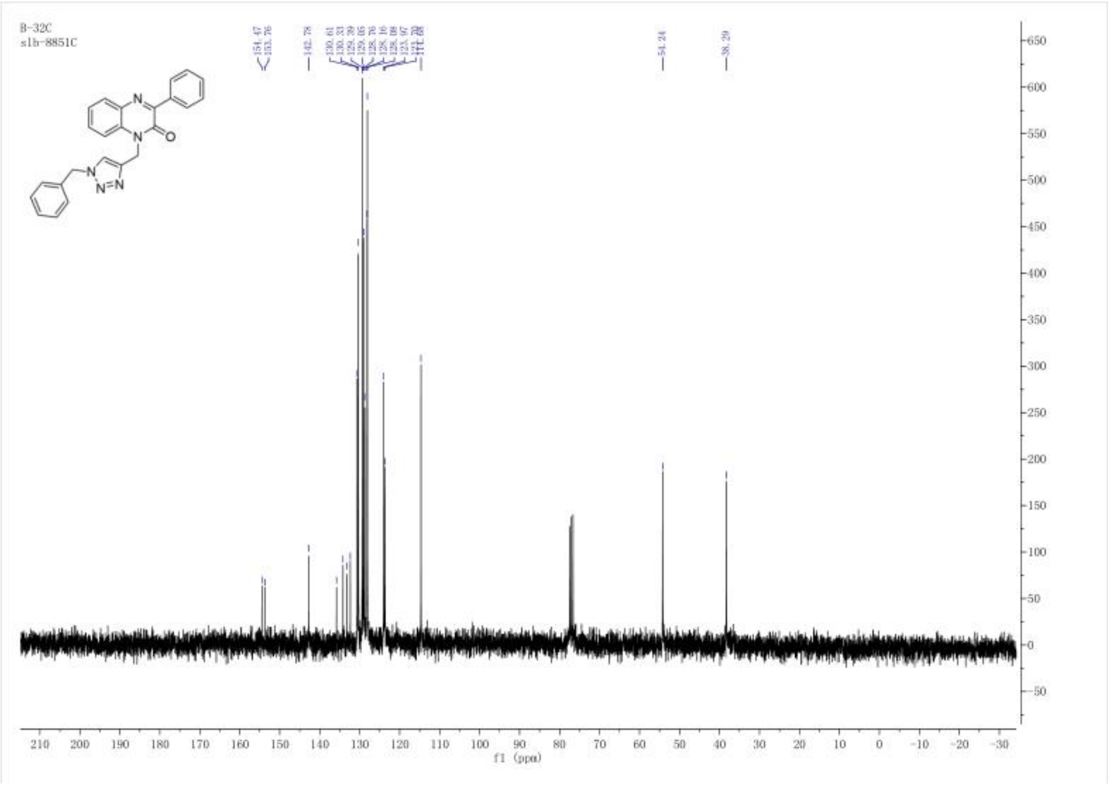

^{13}C NMR (75 MHz, $CDCl_3$) spectrum of compound **6n**

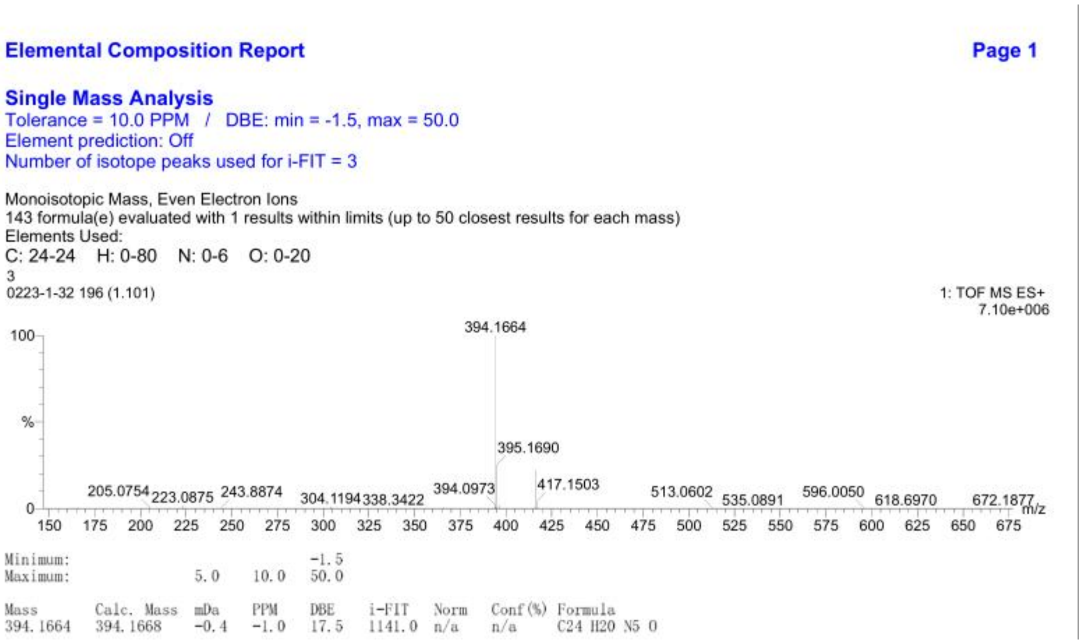

HRMS spectrum of compound **6n**

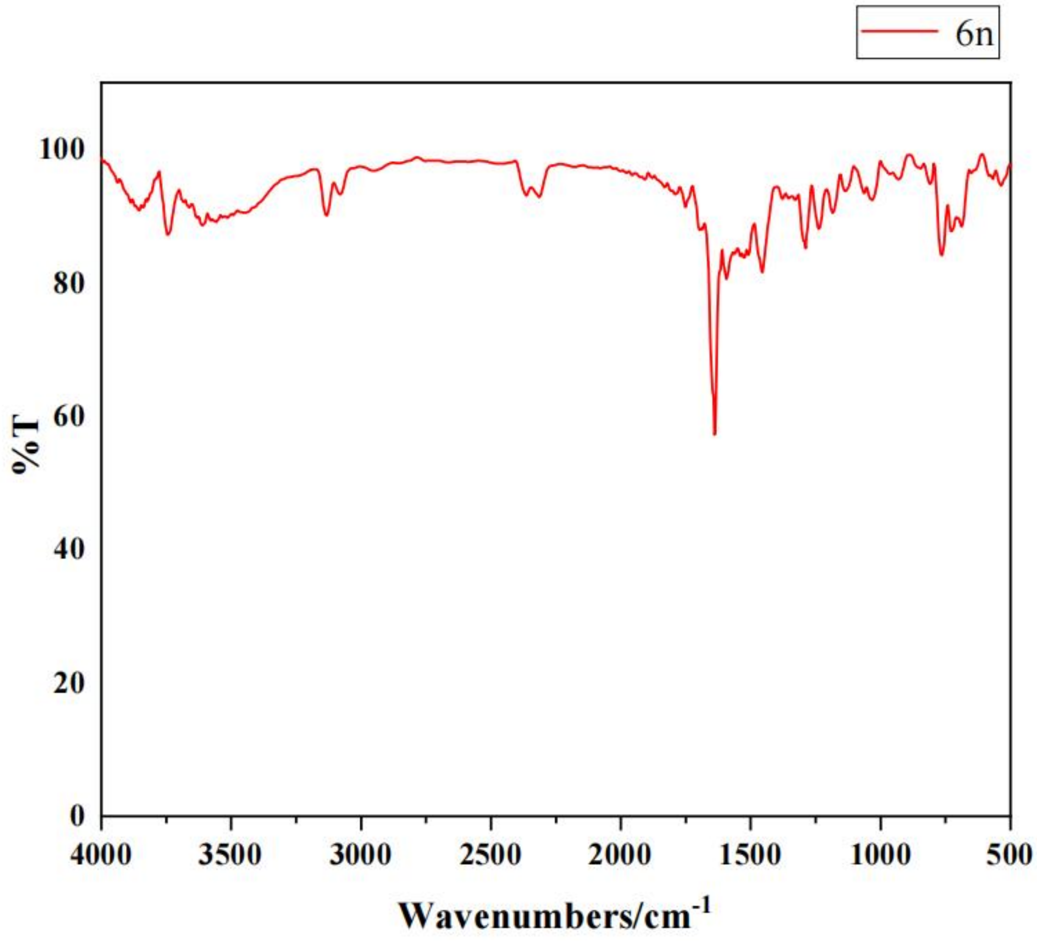

FT-IR spectrum of compound **6n**

The absorbance of compounds 5a-5p and 6a-6n(Unit:nM)

**5a**	**5b**	**5c**	**5d**	**5e**	**5f**	**5g**	**5h**	**5i**	**5j**
**0.044**	**0.047**	**0.045**	**0.056**	**0.058**	**0.059**	**0.071**	**0.090**	**0.076**	**0.054**
**5k**	**5l**	**5m**	**5n**	**5o**	**5p**	**6a**	**6b**	**6c**	**6d**
**0.048**	**0.076**	**0.068**	**0.076**	**0.087**	**0.056**	**0.046**	**0.048**	**0.045**	**0.050**
**6e**	**6f**	**6g**	**6h**	**6i**	**6j**	**6k**	**6l**	**6m**	**6n**
**0.046**	**0.048**	**0.046**	**0.051**	**0.045**	**0.062**	**0.050**	**0.043**	**0.053**	**0.057**
